# Supplementary material for: Light-driven decarboxylative deuteration enabled by a divergently engineered photodecarboxylase
Source: Nat Commun. 2021 Jun 25;12:3983. doi: 10.1038/s41467-021-24259-6 (PMC8233396; doi:10.1038/s41467-021-24259-6)
Supplement: Supplementary file 1 — Supplementary Information [file 41467_2021_24259_MOESM1_ESM.pdf]

Supplementary Information

Light-driven decarboxylative deuteration enabled by a divergently engineered photodecarboxylase

Jian Xu[1,2,4]*, Jiajie Fan[1,4], Yujiao Lou[1,4], Weihua Xu[1,4], Zhiguo Wang[3,4], Danyang Li[1], Haonan Zhou[1], Xianfu Lin[1] & Qi Wu[1]*

[1] Center of Chemistry for Frontier Technologies, Department of Chemistry, Zhejiang University, Hangzhou, 310027, P. R. China. *e-mail: wuqi1000@163.com or llc123@zju.edu.cn.

[2] College of Biotechnology and Bioengineering, Zhejiang University of Technology, Hangzhou, 310014, P. R. China. *e-mail: jianxu@zjut.edu.cn.

[3] Institute of Aging Research, School of Medicine, Hangzhou Normal University, Hangzhou, 311121, P. R. China.

[4] These authors contributed equally.

Supplementary Figures

Supplementary Fig. 1. *Dependence of cosolvent in the CvFAP catalyzed decarboxylative deuteration.* 3

Supplementary Fig. 2. *The effect of substrate concentration on the CvFAP catalyzed decarboxylative deuteration.* 4

Supplementary Fig. 3. *The progress curve of CvFAP catalyzed decarboxylative deuteration.* 5

Supplementary Fig. 4. *Effect of water content in the enzyme system on the D- incorporation.* 6

Supplementary Fig. 5. *Screening results of the FRISM library using the model decarboxylation of nonanoic acid.* 7

Supplementary Fig. 6. *Recovered times of D_2O in the CvFAP catalyzed decarboxylative deuteration.* 8

Supplementary Fig. 7. *SDS-PAGE of WT-CvFAP and mutants.* 9

Supplementary Fig. 8. *Derivatization of 5d to (+)-Igmesine-d_1 with reported methods.* 10

Supplementary Fig. 9-14. *Comparison of the distance between the carboxyl of substrates and the N5 atom of FAD in variants by MD simulation.* 11-16

Supplementary Fig. 15-25. *Chiral GC chromatogram of the enantioselective decarboxylative deuteration of 4a-4k.* 17-27

Supplementary Fig. 26. *NMR spectra of 2-(Hexadecyloxy)acetic acid (1I).* 28

Supplementary Fig. 27. *NMR spectra of 2-(Dodecyloxy)acetic acid (2K).* 29

Supplementary Fig. 28-36. *NMR spectra of 4A-4I.* 30-38

Supplementary Fig. 37-47. *NMR (or/and HRMS) spectra of 1a-1k.* 39-56

Supplementary Fig. 48-58. *NMR (or/and HRMS) spectra of 2a-2k.* 57-71

Supplementary Fig. 59-66. *HRMS spectra of 3a-3h.* 72-79

Supplementary Fig. 67-77. *NMR (or/and HRMS) spectra of 4a-4k.* 80-90

Supplementary Fig. 78-88. *NMR (or/and HRMS) spectra of 5a-5k.* 91-103

Supplementary Tables

Supplementary Table 1 *List of primers.* 104

Supplementary Table 2 *The ratio of M-1 peak to M peak in HRMS of various hydrocarbons with low boiling points.* 105

Supplementary Table 3 *Evaluation of the difference of D-incorporation determined by NMR and HRMS.* 106

Supplementary Table 4 *Protein sequence of CvFAP.* 107

Supplementary Table 5 *DNA sequence of CvFAP.* 108

Supplementary Table 6 *Characterization data of prepared substrates and products.* 109

Supplementary References

Supplementary References 117

Supplementary Figures

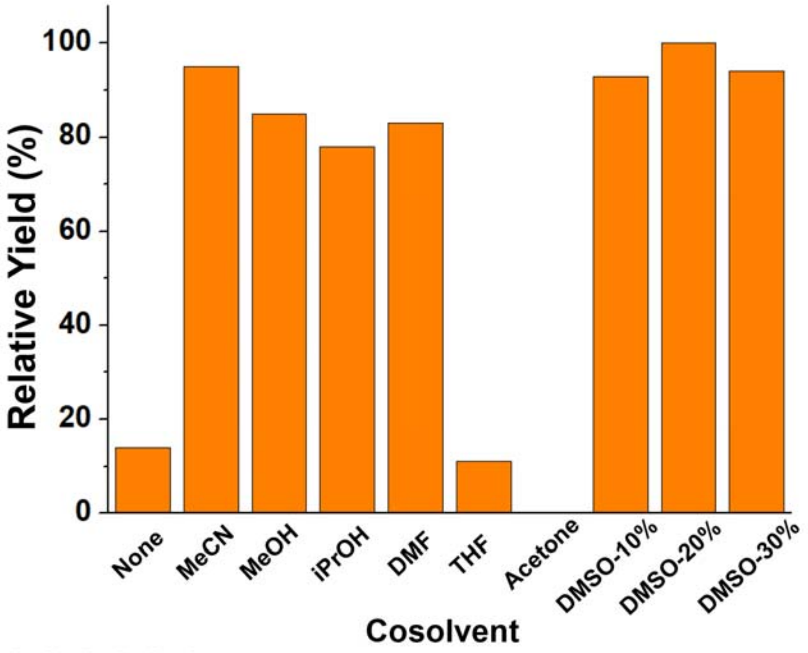

Supplementary Fig. 1. Dependence of cosolvent in the *Cv*FAP catalyzed decarboxylative deuteration. Reaction conditions: palmitic acid (0.40 mmol), crude enzyme powder (containing *Cv*FAP about 20 mg), D_2O (4 mL), cosolvent (1 mL), 450 nm LED, 20 ^oC, 2 h, yields are determined by GC.

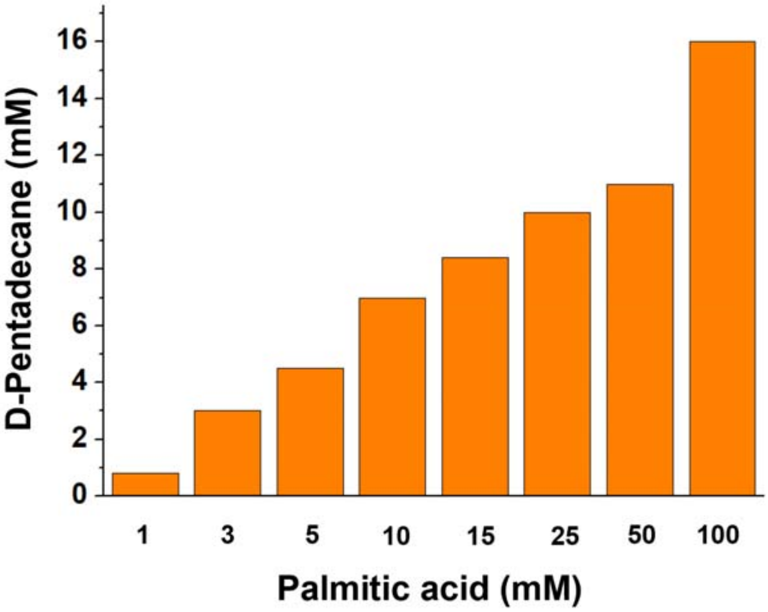

Supplementary Fig. 2. The effect of substrate concentration on the *Cv*FAP catalyzed decarboxylative deuteration. Reaction conditions: palmitic acid (1-100 mM), crude enzyme powder (containing *Cv*FAP about 20 mg), D_2O (4 mL), DMSO (1 mL), 450 nm LED, 20 ^oC, 2 h. Yields are determined by GC.

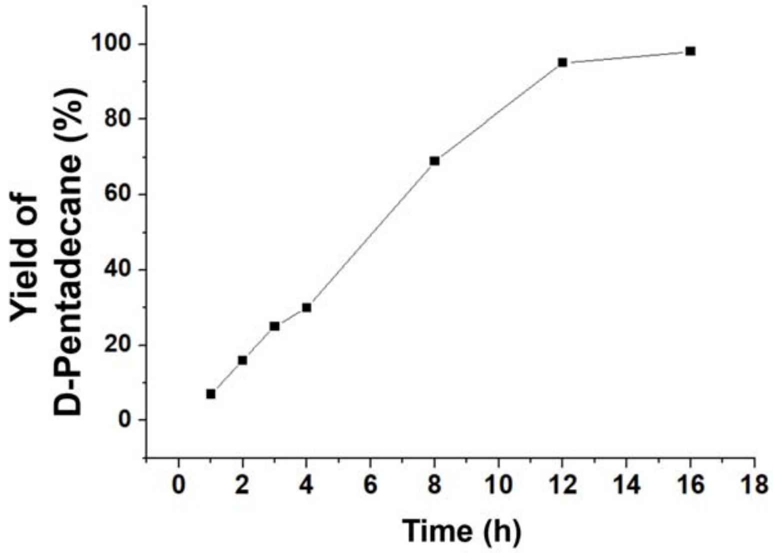

Supplementary Fig. 3. The progress curve of *Cv*FAP catalyzed decarboxylative deuteration. Reaction conditions: palmitic acid (100 mM), crude enzyme powder (containing *Cv*FAP about 20 mg), D_2O (4 mL), DMSO (1 mL), 450 nm LED, 20 °C, 1-16 h, yields are determined by GC.

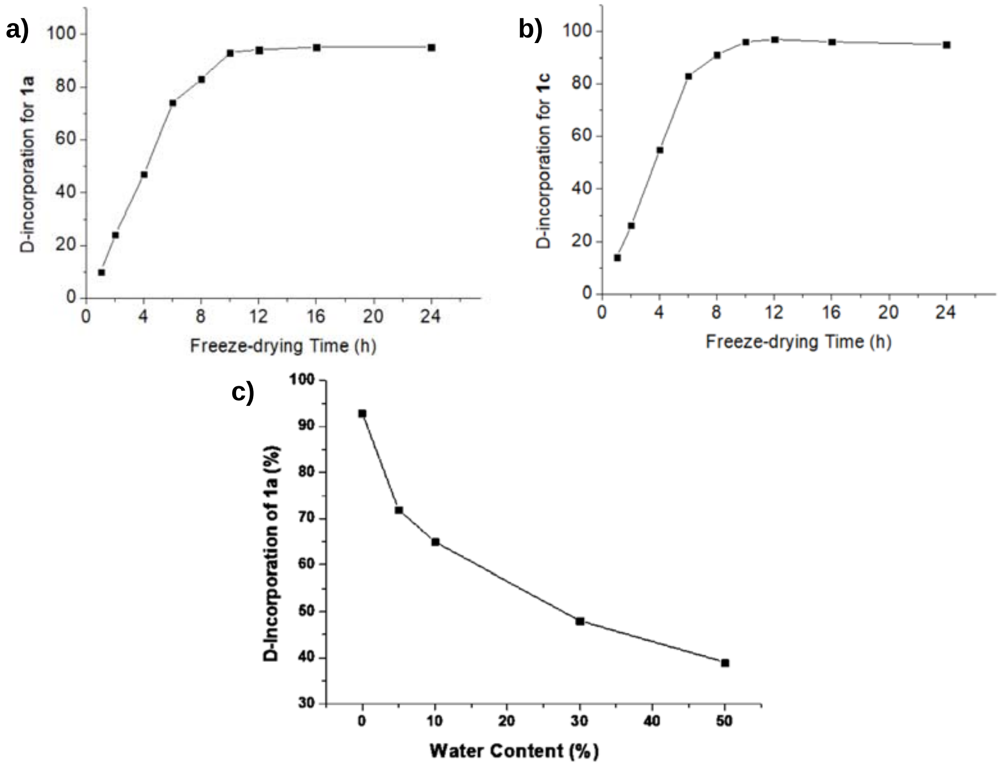

Supplementary Fig. 4. Effect of water content in the enzyme system on the D- incorporation. a) the D- incorporation of **1a** obtained from WT *Cv*FAP treated with different freeze-drying time. **b**) the D- incorporation of **1c** obtained from WT *Cv*FAP treated with different freeze-drying time. **c**) Effect of water content in WT *Cv*FAP on the D- incorporation of **1a**. Reaction conditions: substrate (0.40 mmol), crude enzyme powder (containing *Cv*FAP about 20 mg), D_2O (4 mL), DMSO (1 mL), 450 nm LED, 20 °C, 12 h, yields are determined by GC. D-inc. data are determined by ^1H NMR or HRMS.

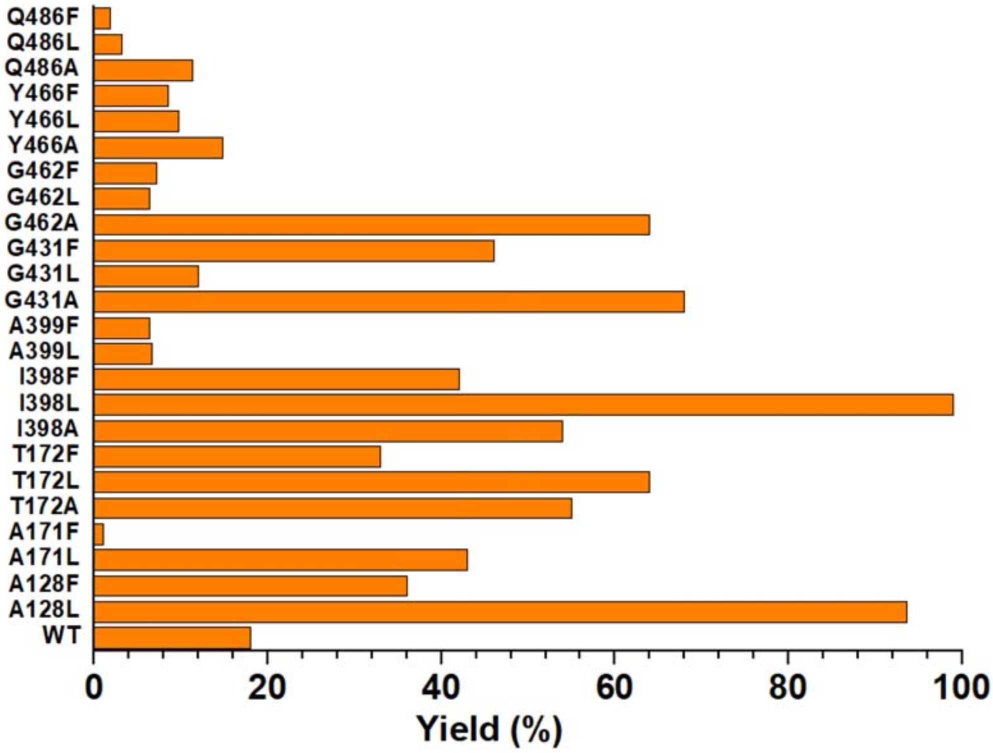

Supplementary Fig. 5. Screening results of the FRISM library using the model decarboxylation of nonanoic acid. Reaction conditions: nonanoic acid (0.02 mmol), 1 mL crude enzyme solutions of different mutants (1 g wet cell in 10 mL pH 8.5 phosphate buffer), DMSO (0.2 mL), 450 nm LED, 20 °C, 12 h, yields are determined by GC.

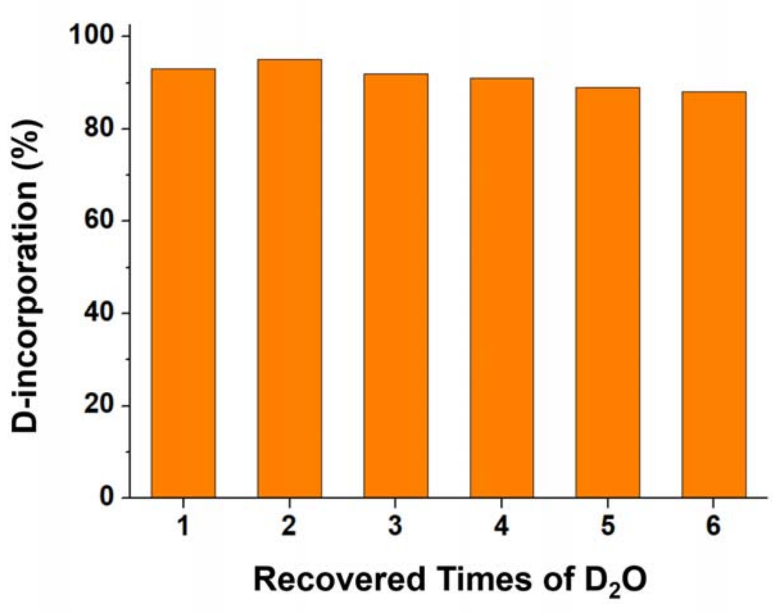

Supplementary Fig. 6. Recovered times of D_2O in the *Cv*FAP catalyzed decarboxylative deuteration. D_2O was recovered by vacuum distillation. Reaction conditions: palmitic acid (100 mM), crude enzyme powder (containing *Cv*FAP about 20 mg), D_2O (4 mL), DMSO (1 mL), 450 nm LED, 20 ^oC, 12 h, yield is determined by GC, D-inc. was determined by HRMS.

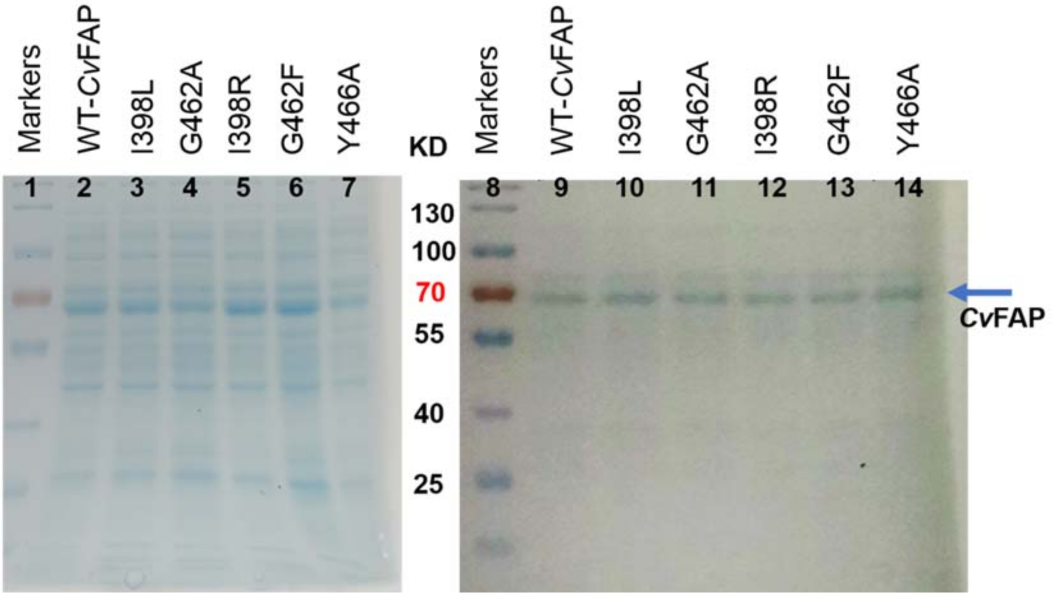

Supplementary Fig. 7 SDS-PAGE of WT-*Cv*FAP and mutants expressed in *E. coli*. Lane 1 and 8: protein markers. Lane 2-7: cell extract of WT *Cv*FAP, I398L, G462A, I398R, G462F, and Y466A. Lane 9-14: protein of WT *Cv*FAP, I398L, G462A, I398R, G462F, and Y466A after purification. Three experiment was repeated independently with similar results.

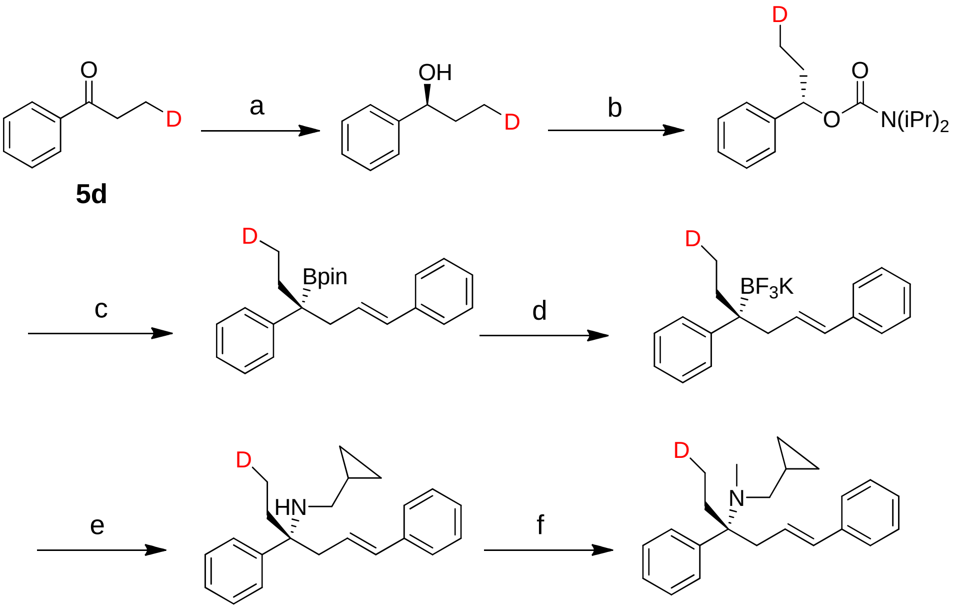

(+)-Igmesine-d_1

Supplementary Fig. 8. Derivatization of 5d to (+)-Igmesine-d_1 with reported methods.[1,2] Reagents and conditions: a) whole-cell culture medium of ketoreductase with glucose, 30 °C, over night; b) iPr_2NCOCl (1.05 equiv), NEt_3 (1.1 equiv), CH_2Cl_2, reflux, 24 h; c) *sec*-butyllithium (1.1 equiv), -78°C, 20 min; then cinnamyl boronic acid pinacol ester (1.2 equiv), -78°C, 1 h; then 1M $MgBr_2$ in MeOH (1.2 equiv), -78°C, 10 min, then RT, 16 h; then 1M aq KH_2PO_4; d) 4.5m aq KHF_2 (2.5 equiv), MeOH, RT, 30 min, evaporation; then 50% aq MeOH, 10 min and evaporation (5x); e) $SiCl_4$, (2 equiv), DCE, RT, 1 h; *c*$PrCH_2N_3$ (2 equiv), 80°C, 30 min; then 2M aq NaOH, RT, 1 h; f) 37% aq CH_2O, $NaHB(OAc)_3$, DCE, RT, 16 h.

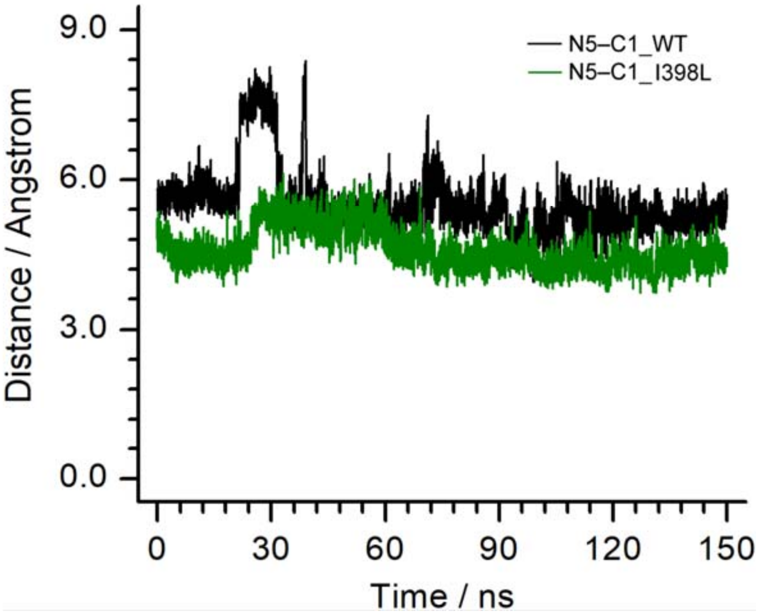

Supplementary Fig. 9. **Comparison of the distance between the carboxyl of nonanoic acid (2A) and the N5 atom of FAD in WT-*Cv*FAP and I398L by MD simulation.**

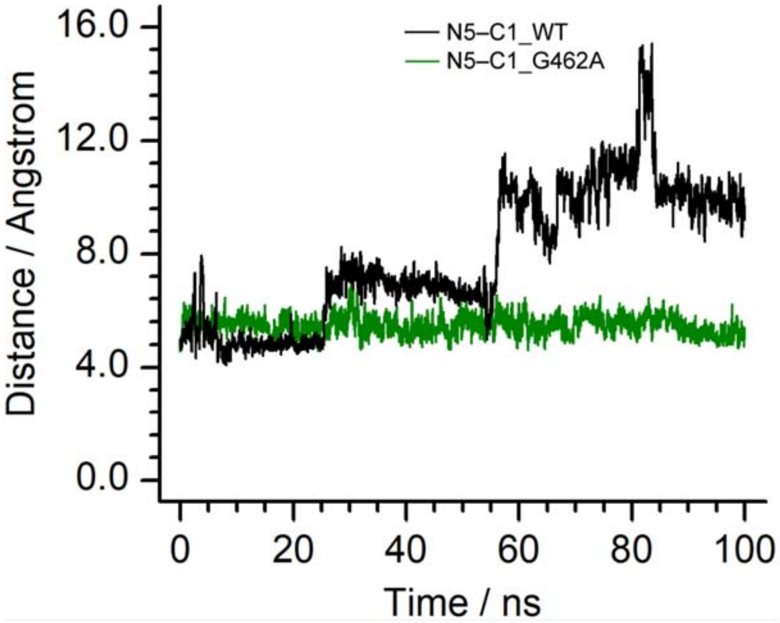

Supplementary Fig. 10. **Comparison of the distance between the carboxyl of propionic acid (3A) and the N5 atom of FAD in WT-*Cv*FAP and G462A by MD simulation.**

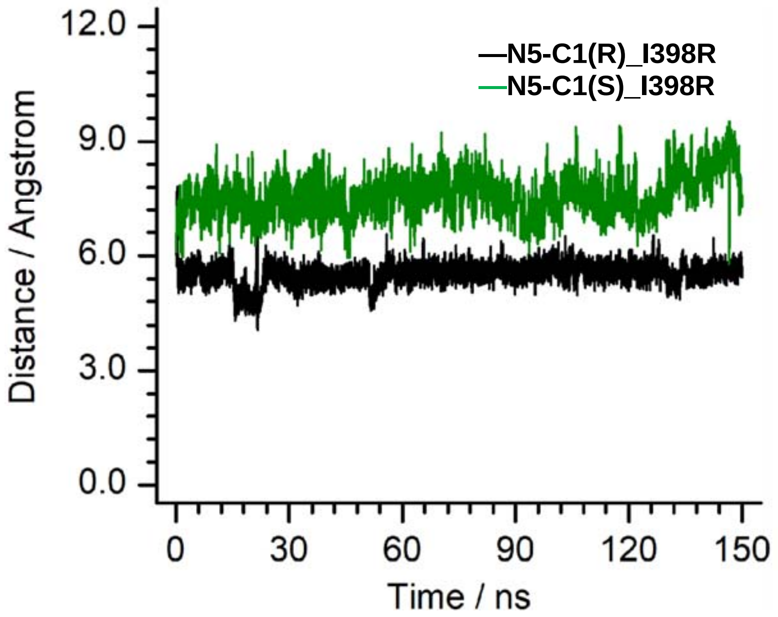

Supplementary Fig. 11. **Comparison of the distance between the carboxyl of (*R*)- and (*S*)- 2-(heptan-3-yloxy) acetic acid (4A) and the N5 atom of FAD in I398R by MD simulation.**

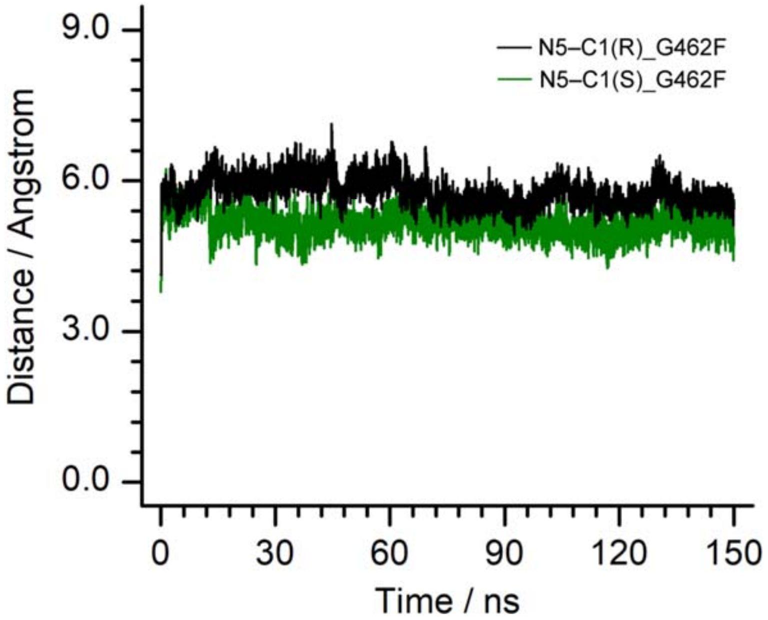

Supplementary Fig. 12. Comparison of the distance between the carboxyl of (*R*)- and (*S*)- 2-(heptan-3-yloxy) acetic acid (4A) and the N5 atom of FAD in G462F by MD simulation.

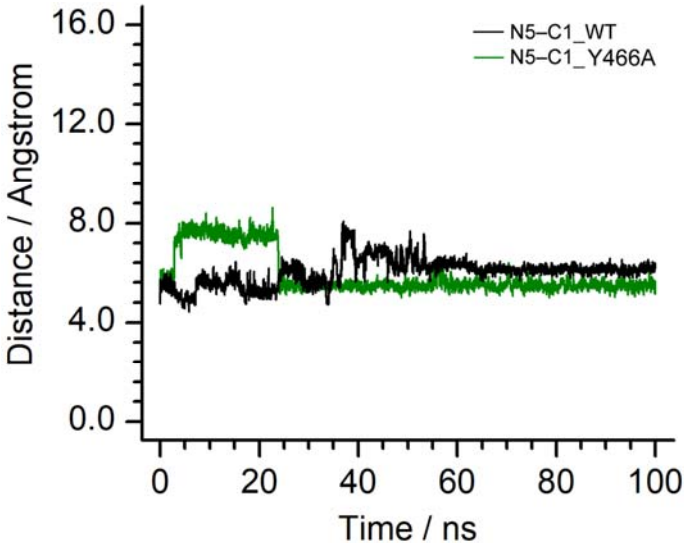

Supplementary Fig. 13. **Comparison of the distance between the carboxyl of (*S*)- 3-phenylbutanoic acid ((*S*)-5A) and the N5 atom of FAD in WT-*Cv*FAP and Y466A by MD simulation.**

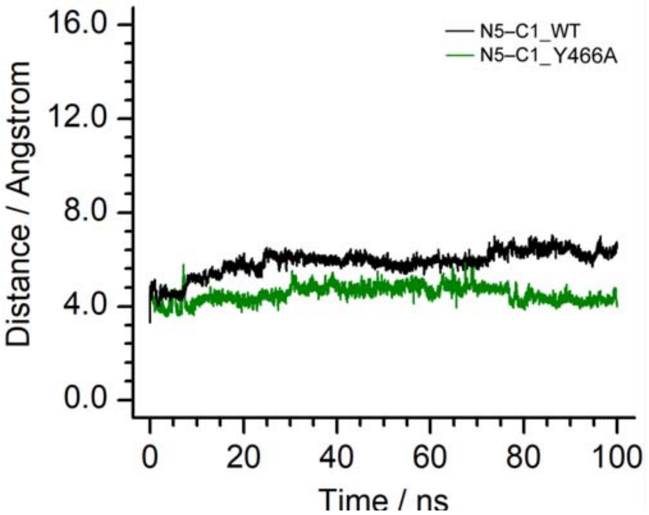

Supplementary Fig. 14. **Comparison of the distance between the carboxyl of (*R*)- 3-phenylbutanoic acid ((*R*)-5A)and the N5 atom of FAD in WT-*Cv*FAP and Y466A by MD simulation.**

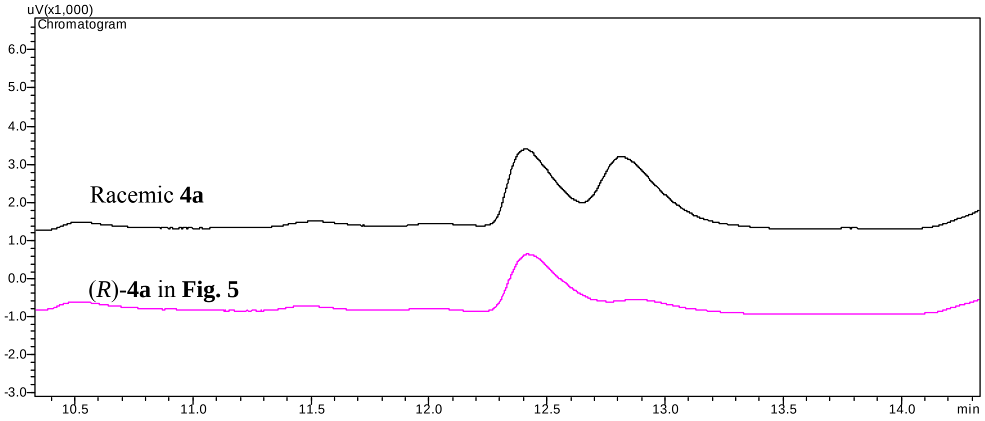

Supplementary Fig. 15. **Chiral GC chromatogram of the enantioselective decarboxylative deuteration of 4a.** Agilent CP-chirasil-Dex CB, T_R = 12.47 min, T_S = 12.87 min, Temperature conditions: initial temperature 40 ºC, holding 15 min, then 40 ºC/min to 200 ºC, holding 1 min.

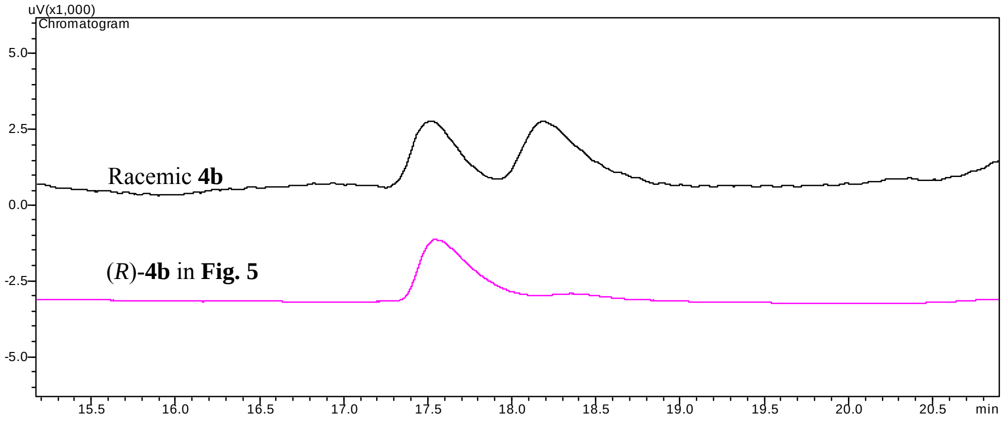

Supplementary Fig. 16. **Chiral GC chromatogram of the enantioselective decarboxylative deuteration of 4b.** Agilent CP-chirasil-Dex CB, T_R = 17.52 min, T_S = 18.19 min, Temperature conditions: initial temperature 40 ºC, holding 20 min, then 40 ºC/min to 200 ºC, holding 1 min

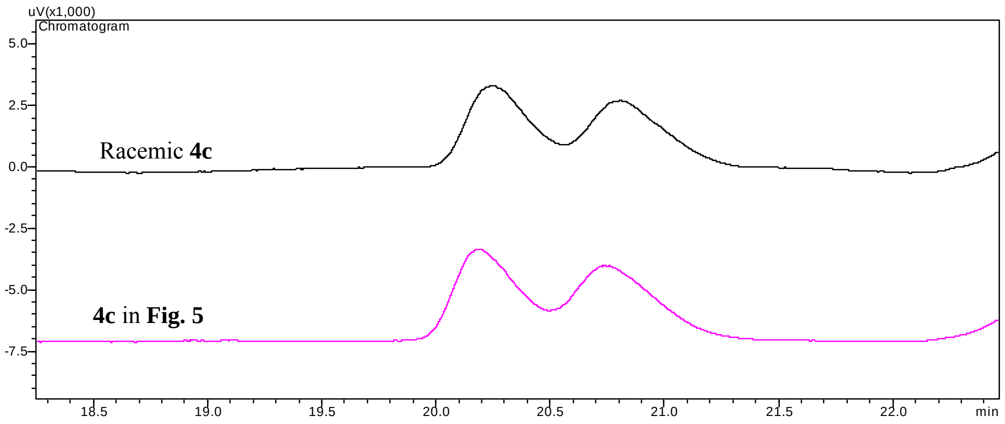

Supplementary Fig. 17. **Chiral GC chromatogram of the enantioselective decarboxylative deuteration of 4c.** Agilent CP-chirasil-Dex CB, T_R = 20.21 min, T_S = 20.75 min, Temperature conditions: initial temperature 50 ºC, holding 22 min, then 40 ºC/min to 200 ºC, holding 1 min.

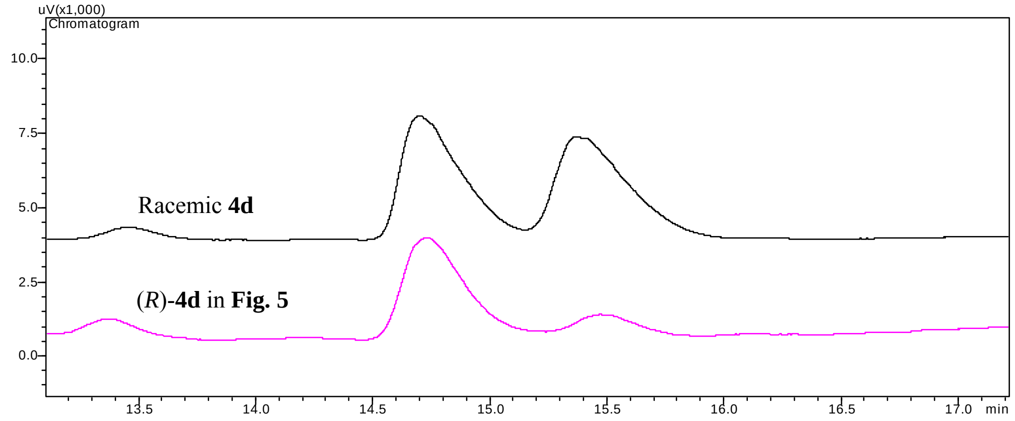

Supplementary Fig. 18. **Chiral GC chromatogram of the enantioselective decarboxylative deuteration of 4d.** Agilent CP-chirasil-Dex CB, T_R = 14.77 min, T_S = 15.46 min, Temperature conditions: initial temperature 40 °C, holding 20 min, then 40 °C/min to 200 °C, holding 1 min.

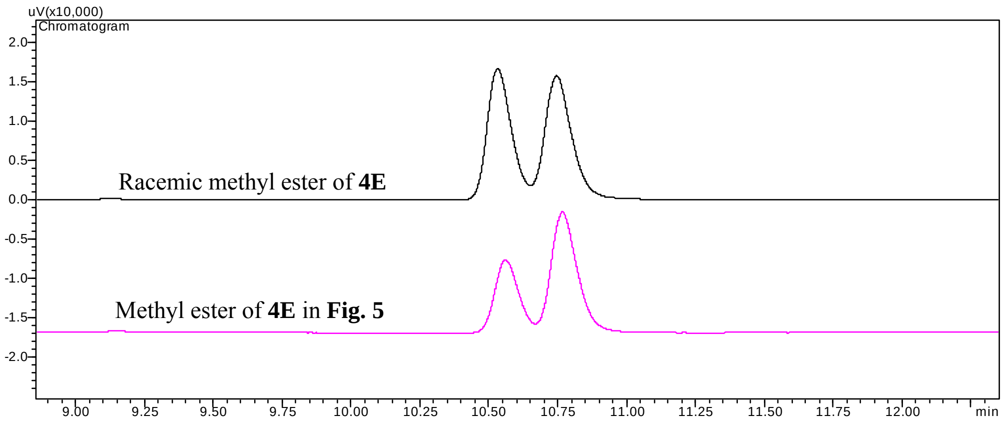

Supplementary Fig. 19. **Chiral GC chromatogram of the enantioselective decarboxylative deuteration of 4E.** Agilent CP-chirasil-Dex CB, T_R = 10.58 min, T_S = 10.79 min, Temperature conditions: initial temperature 100 ^oC, 2 ^oC/min to 130 ^oC, then 35 ^oC/min to 200 ^oC, holding 1 min. The e.r. value of **4E** was determined after the sulfuric acid-catalyzed derivatization with methyl alcohol, and the e.r. value of **4e** was calculated based on the conversion and e.r. of **4E**.

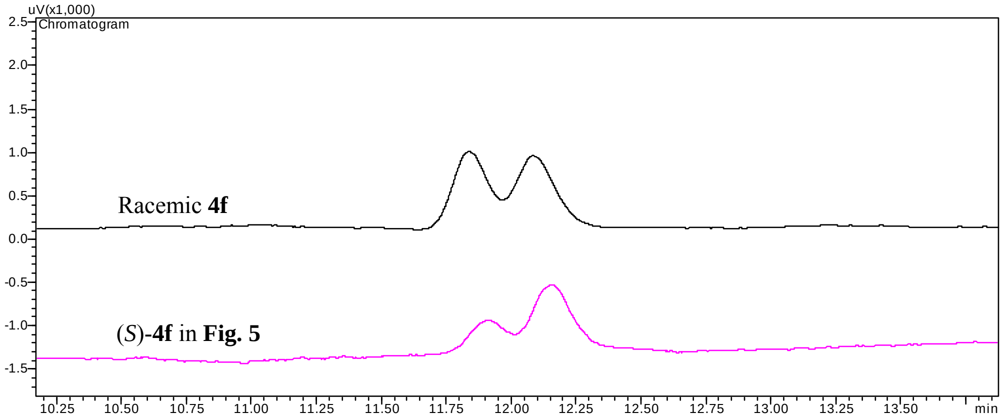

Supplementary Fig. 20. **Chiral GC chromatogram of the enantioselective decarboxylative deuteration of 4f.** Agilent CP-chirasil-Dex CB, T_R = 11.91 min, T_S = 12.16 min, Temperature conditions: initial temperature 40 °C, holding 7 min, 1 °C /min to 50 °C, then 50 °C /min to 200 °C, holding 1 min.

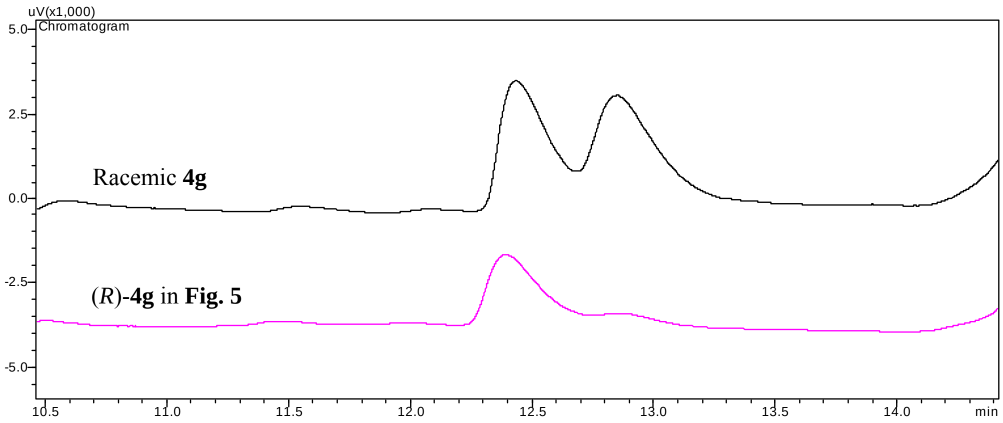

Supplementary Fig. 21. **Chiral GC chromatogram of the enantioselective decarboxylative deuteration of 4g.** Agilent CP-chirasil-Dex CB, T_R = 12.47 min, T_S = 12.87 min, Temperature conditions: initial temperature 40 ºC, holding 15 min, then 40 ºC /min to 200 ºC, holding 1 min.

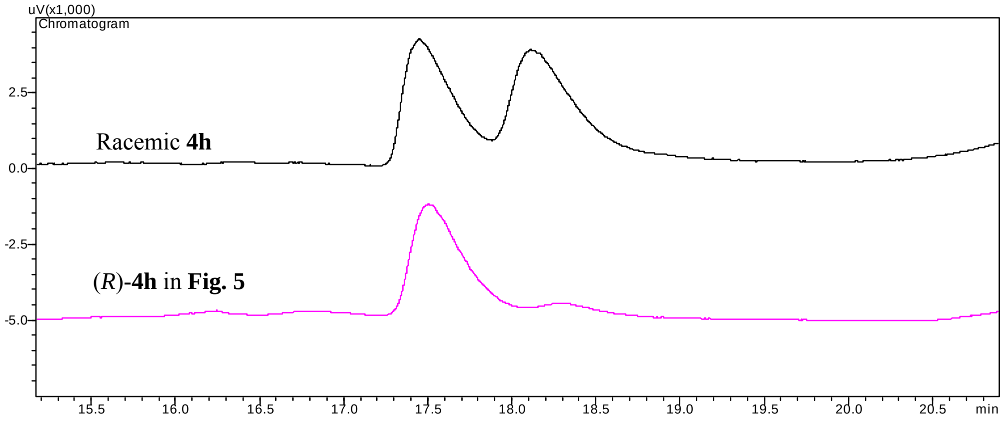

Supplementary Fig. 22. **Chiral GC chromatogram of the enantioselective decarboxylative deuteration of 4h.** Agilent CP-chirasil-Dex CB, T_R = 17.53 min, T_S = 18.20 min, Temperature conditions: initial temperature 40 ºC, holding 20 min, then 40 ºC /min to 200 ºC, holding 1 min.

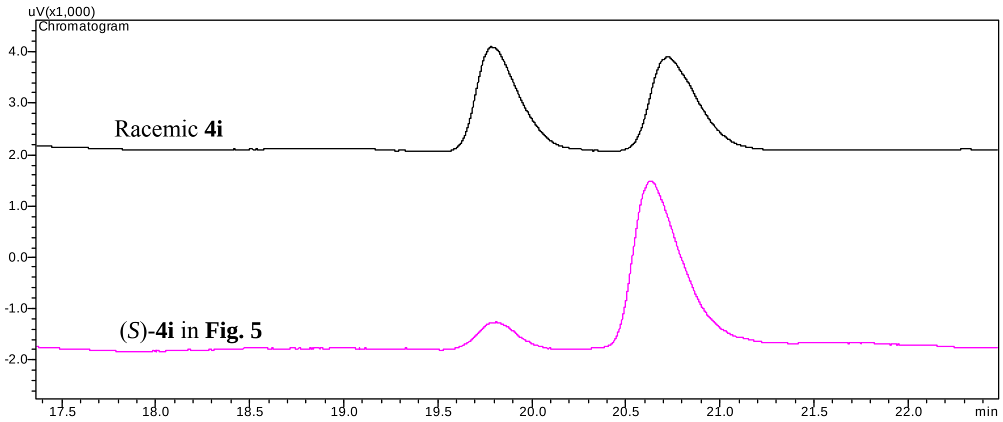

Supplementary Fig. 23. **Chiral GC chromatogram of the enantioselective decarboxylative deuteration of 4i.** Agilent CP-chirasil-Dex CB, T_R = 19.78 min, T_S = 20.73 min, Temperature conditions: initial temperature 50 ºC, holding 8 min, then 50 ºC /min to 200 ºC, holding 1 min.

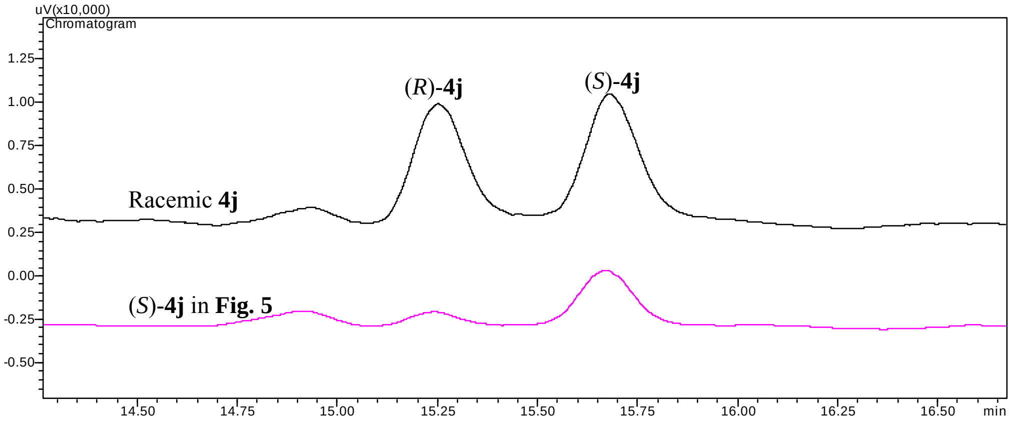

Supplementary Fig. 24. **Chiral GC chromatogram of the enantioselective decarboxylative deuteration of 4j.** Agilent CP-chirasil-Dex CB, T_R = 15.24 min, T_S = 15.67min, Temperature conditions: initial temperature 80 ºC, holding 5 min, 2 ºC /min to 110 ºC, then 45 ºC /min to 200 ºC, holding 1 min. The e.r. value of **4j** were determined after the derivatization with butyryl chloride.

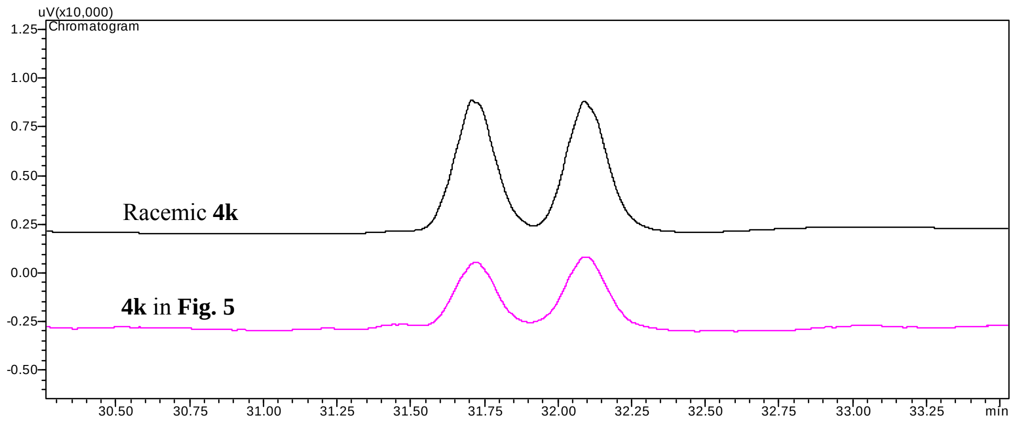

Supplementary Fig. 25. **Chiral GC chromatogram of the enantioselective decarboxylative deuteration of 4k.** Agilent CP-chirasil-Dex CB, T_R = 31.73 min, T_S = 32.11 min. Temperature conditions: initial temperature 80 ºC, holding 5 min, 5 ºC /min to 160 ºC, then 40 ºC /min to 200 ºC, holding 1 min. The e.r. value of **4k** were determined after the derivatization with butyryl chloride.

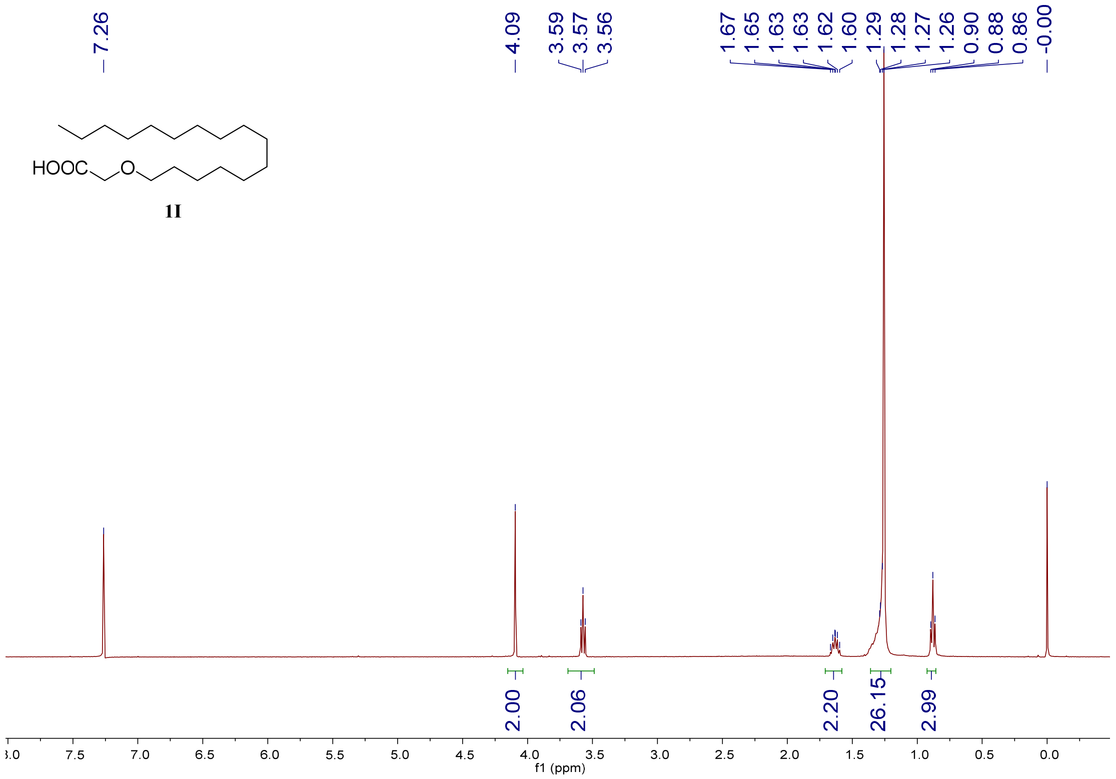

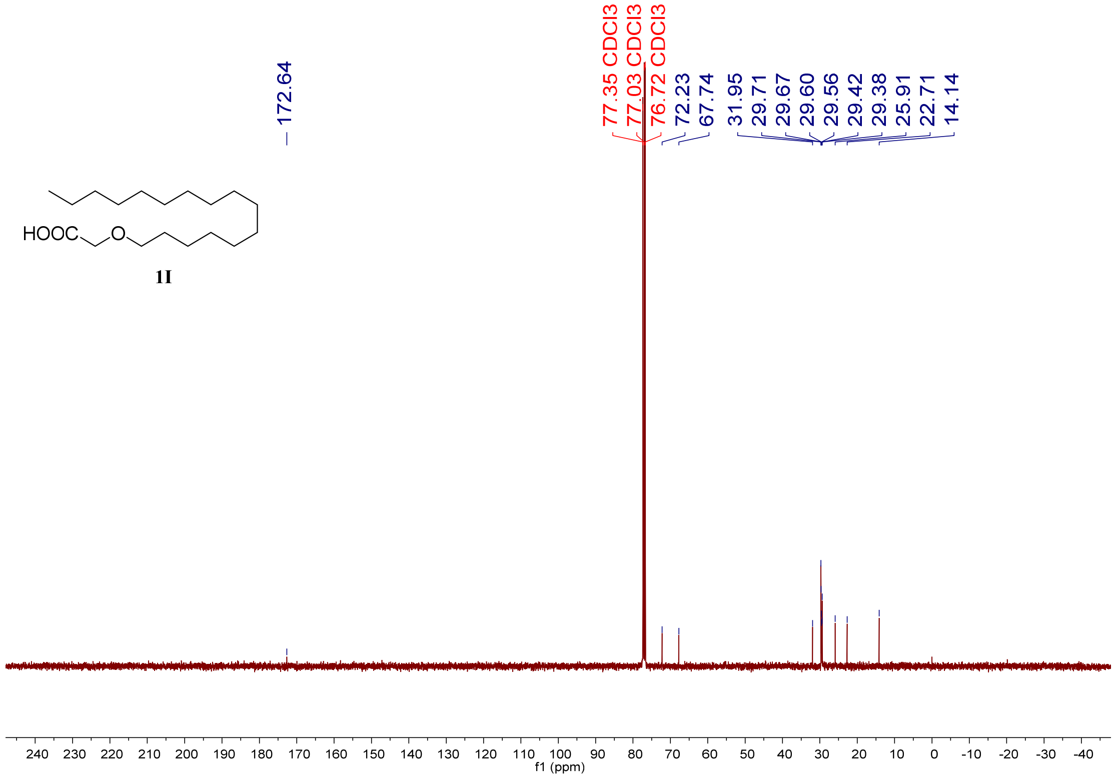

Supplementary Fig. 26. NMR spectra of 2-(Hexadecyloxy)acetic acid (1l).

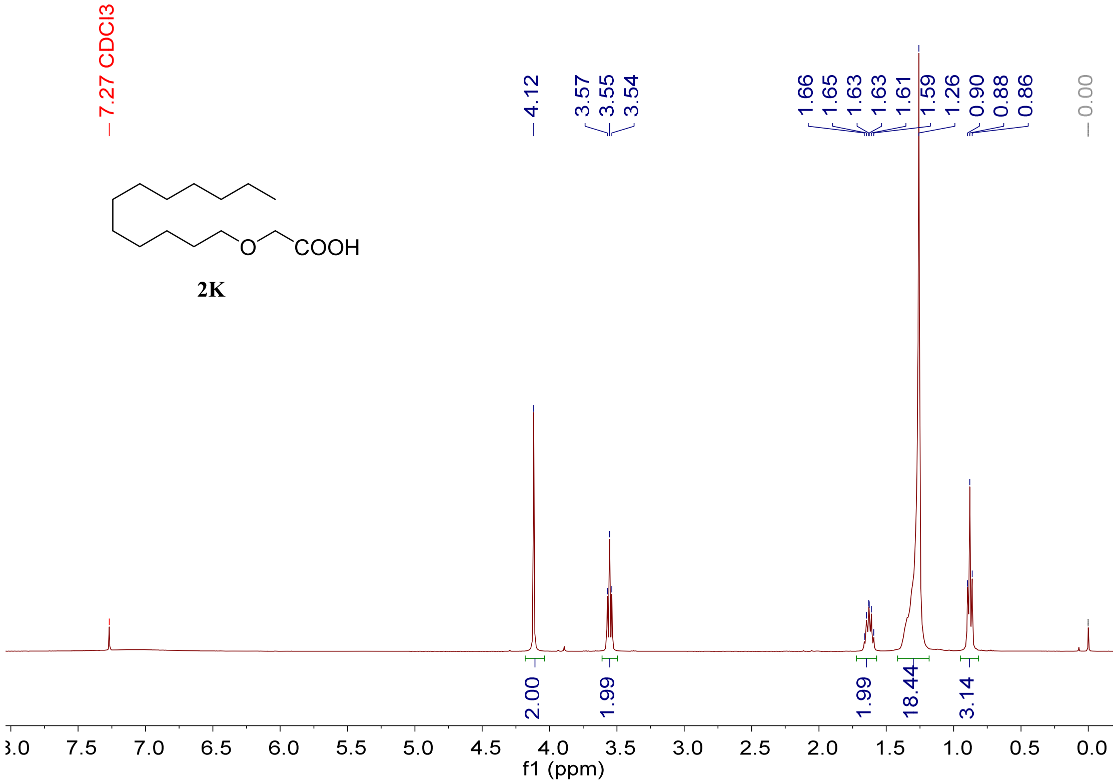

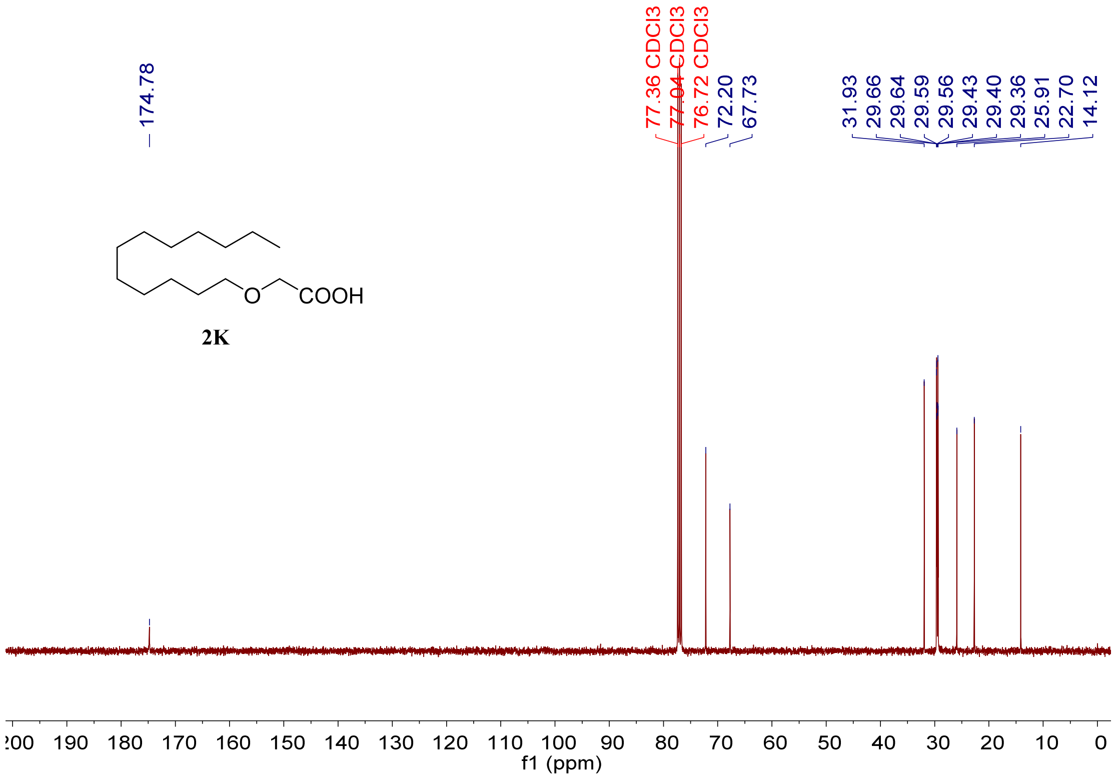

Supplementary Fig. 27. NMR spectra of 2-(Dodecyloxy)acetic acid (2K).

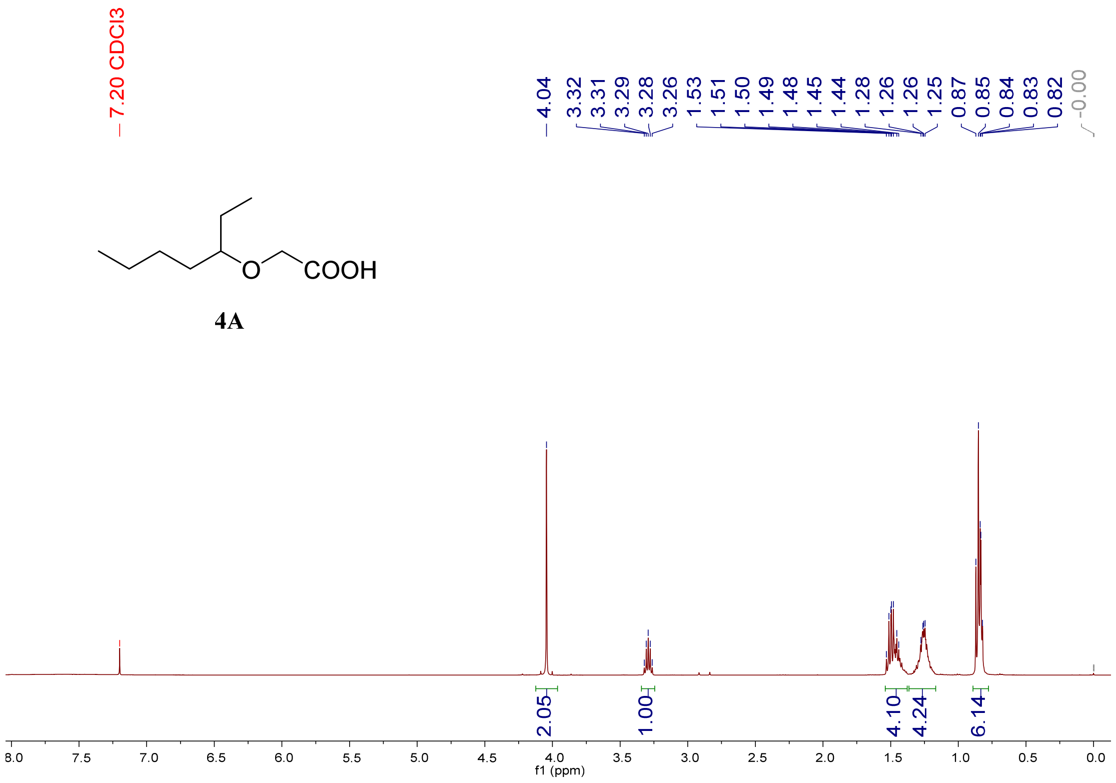

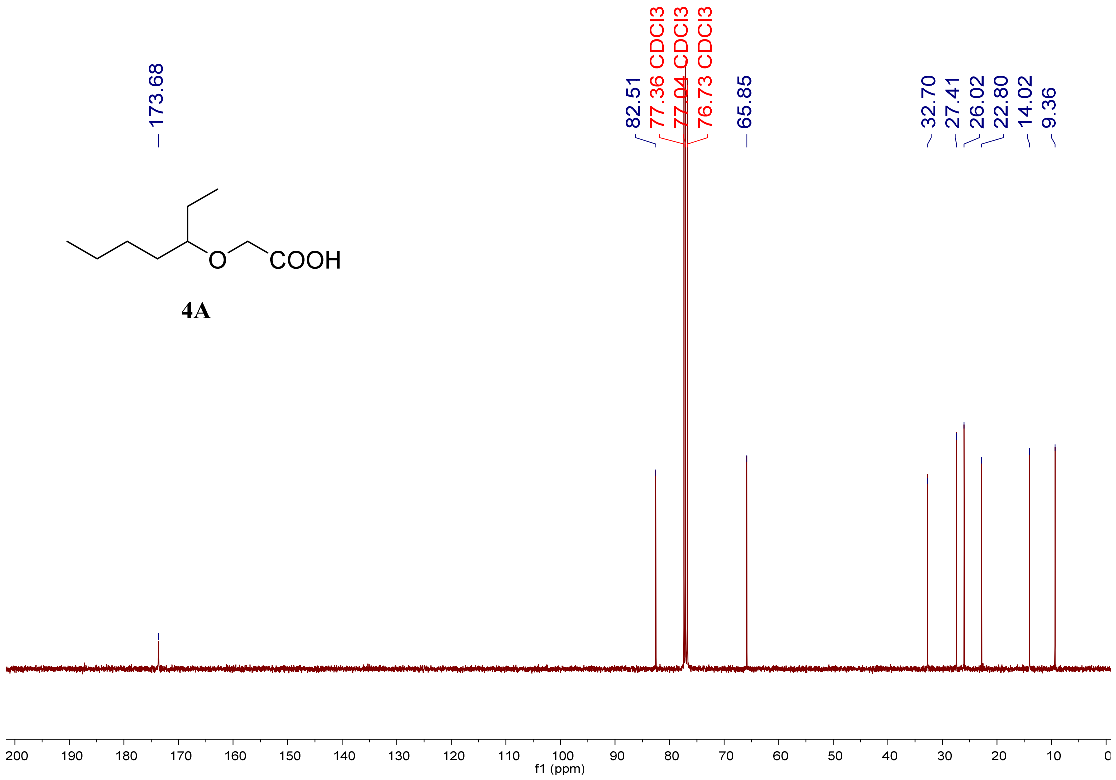

Supplementary Fig. 28. NMR spectra of 2-(Heptan-3-yloxy)acetic acid (4A).

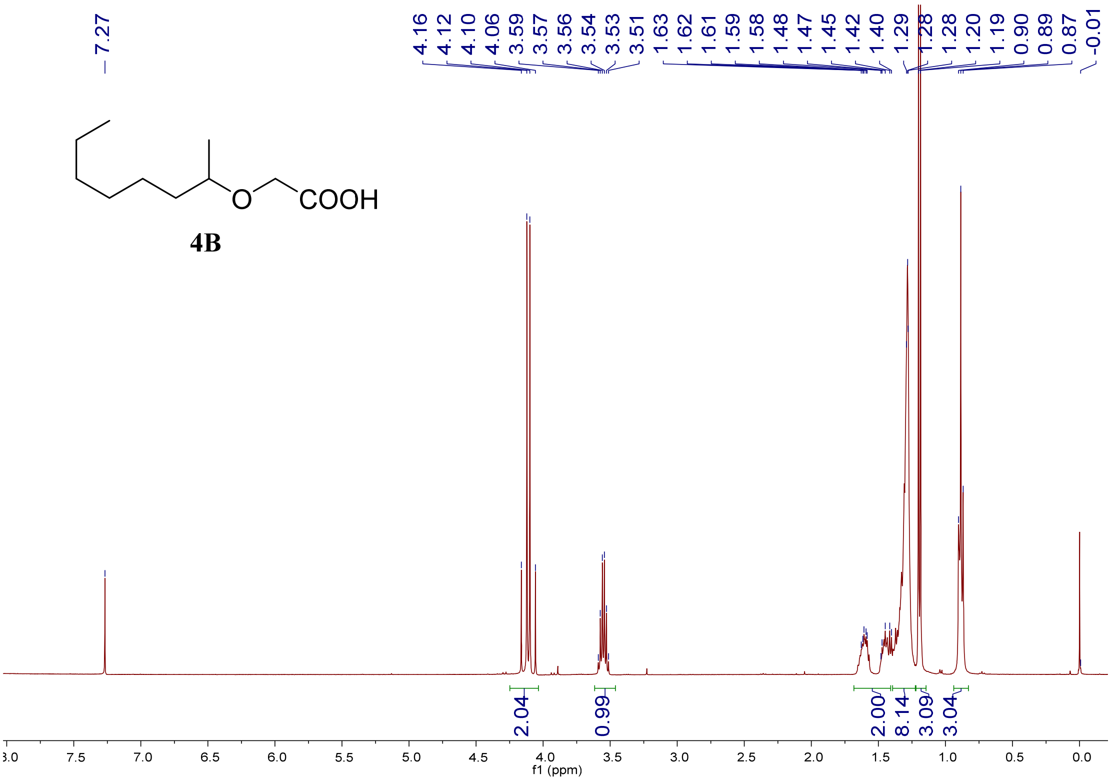

4B

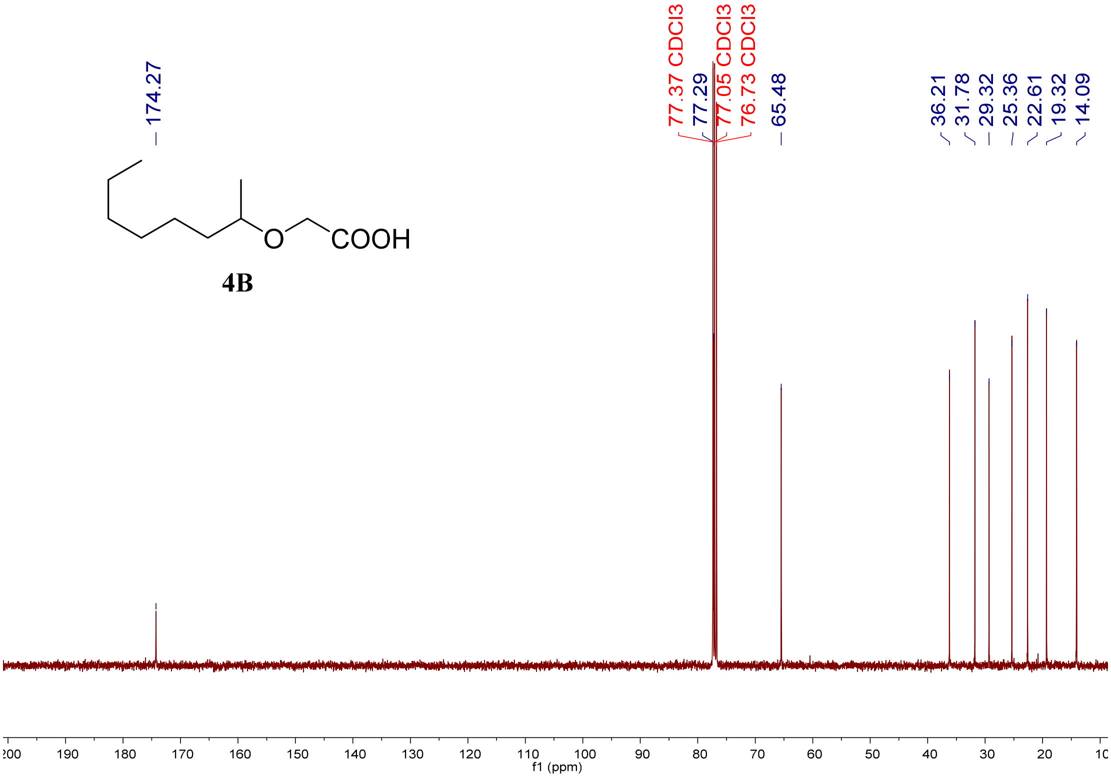

4B

Supplementary Fig. 29. NMR spectra of 2-(Octan-2-yloxy)acetic acid (4B).

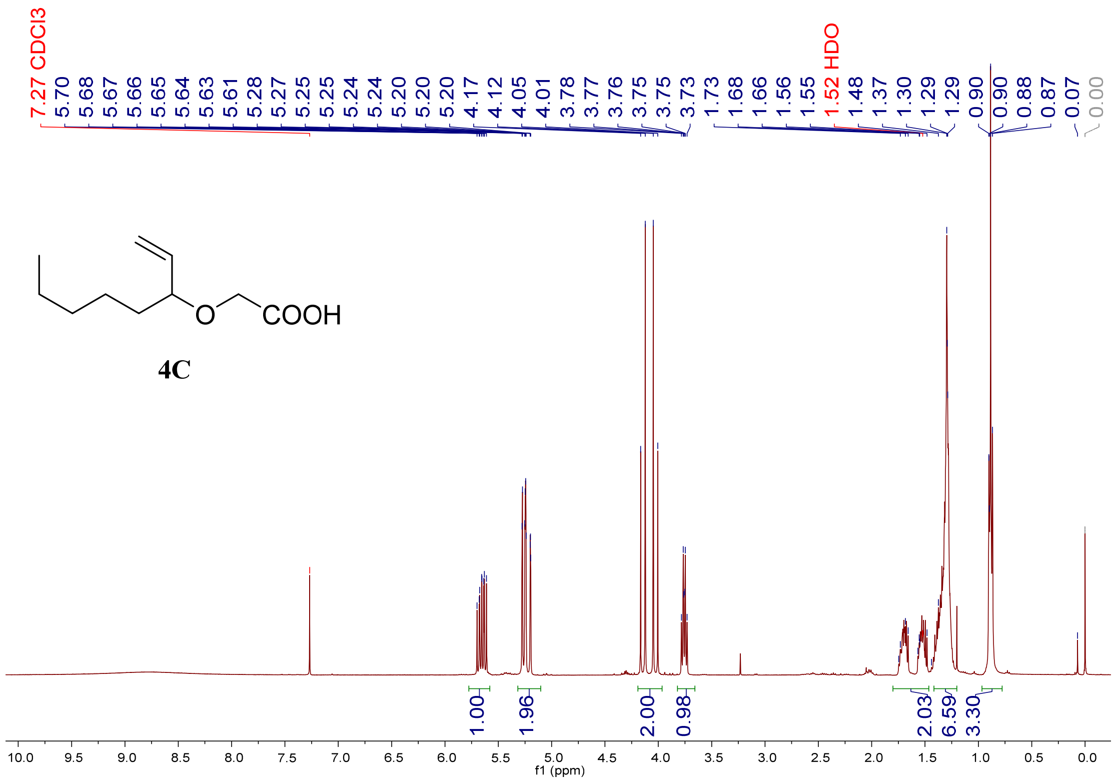

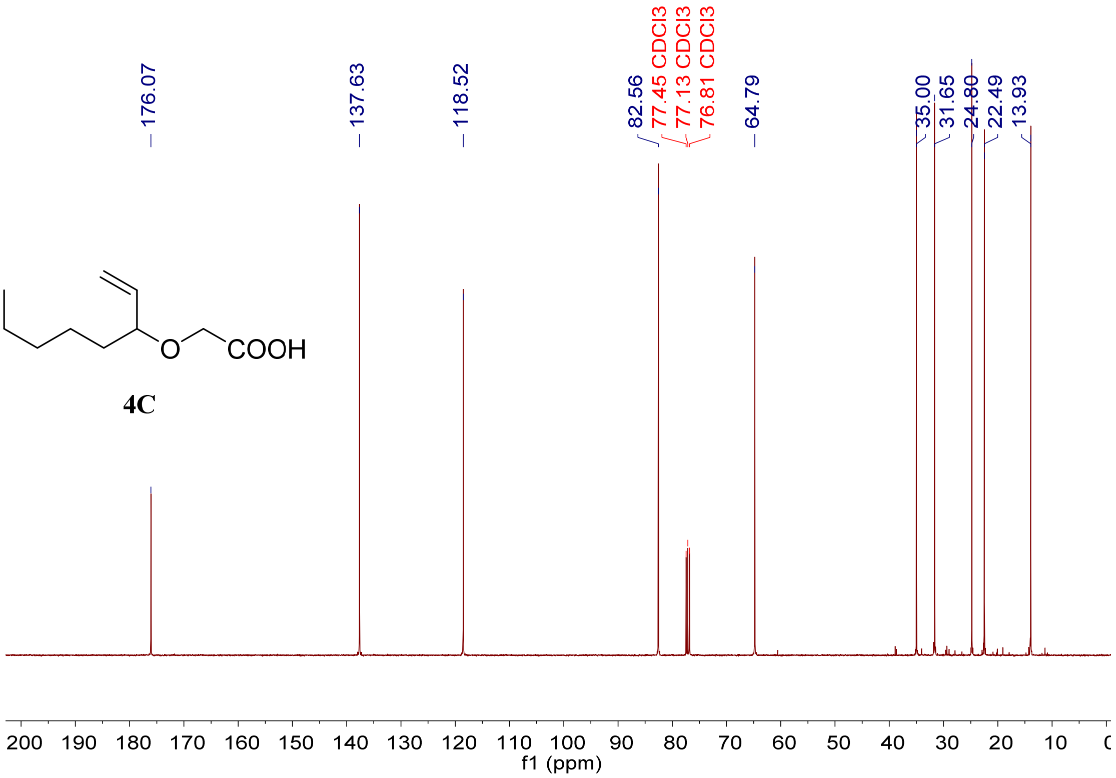

Supplementary Fig. 30. NMR spectra of 2-(Oct-1-en-3-yloxy)acetic acid (4C).

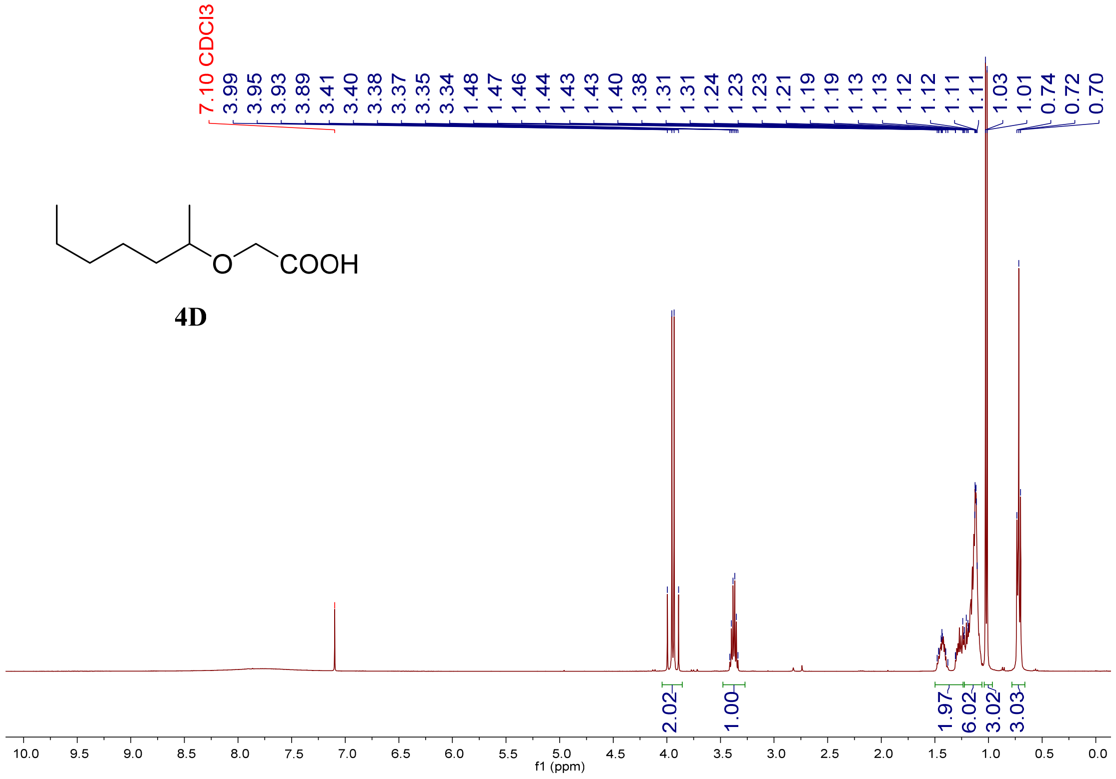

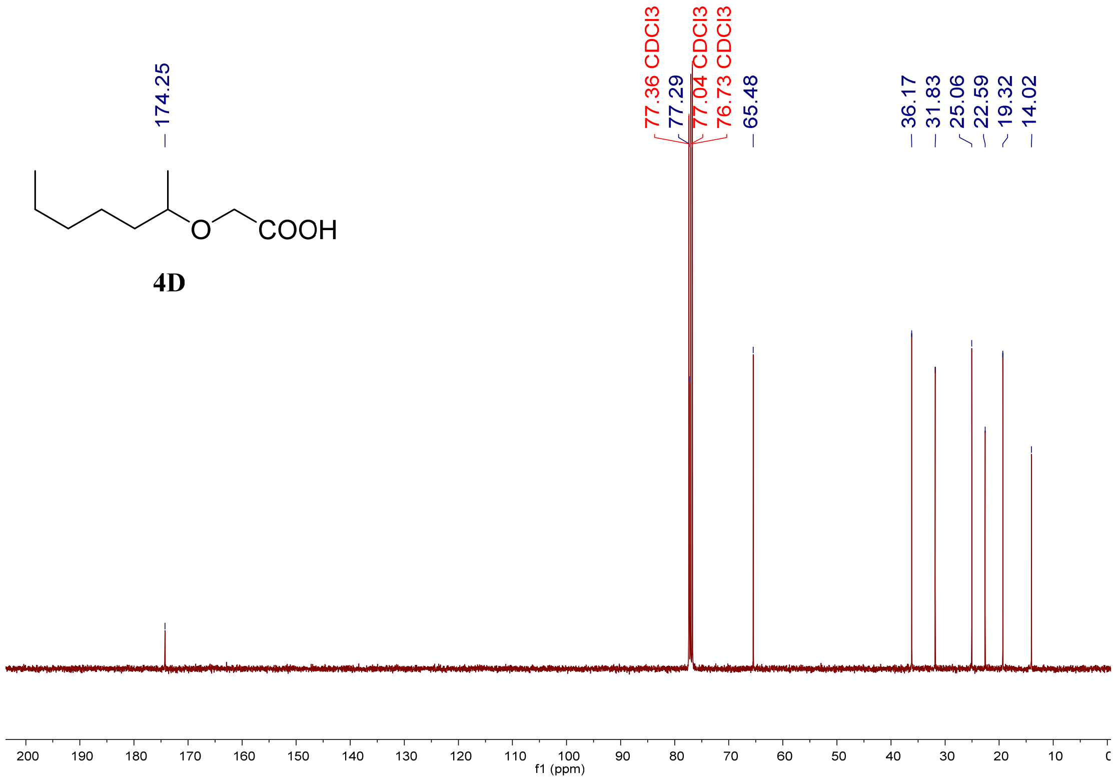

Supplementary Fig. 31. NMR spectra of 2-(Heptan-2-yloxy)acetic acid (4D).

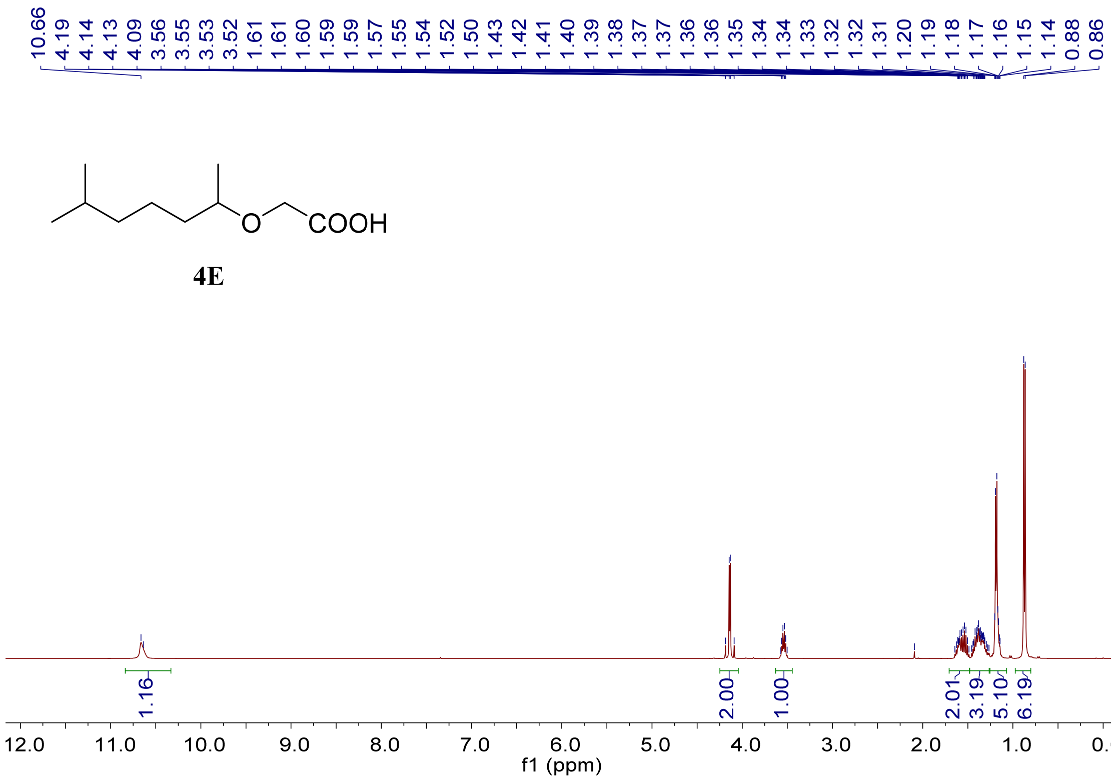

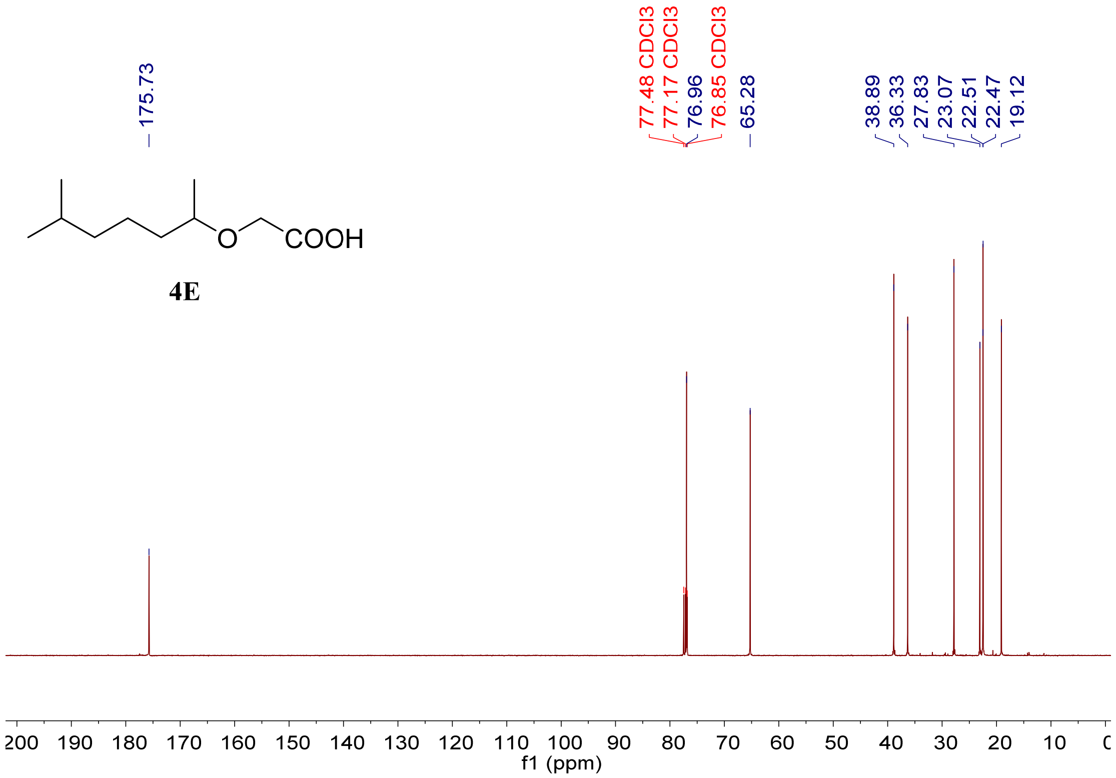

Supplementary Fig. 32. NMR spectra of 2-((6-Methylheptan-2-yl)oxy)acetic acid (4E).

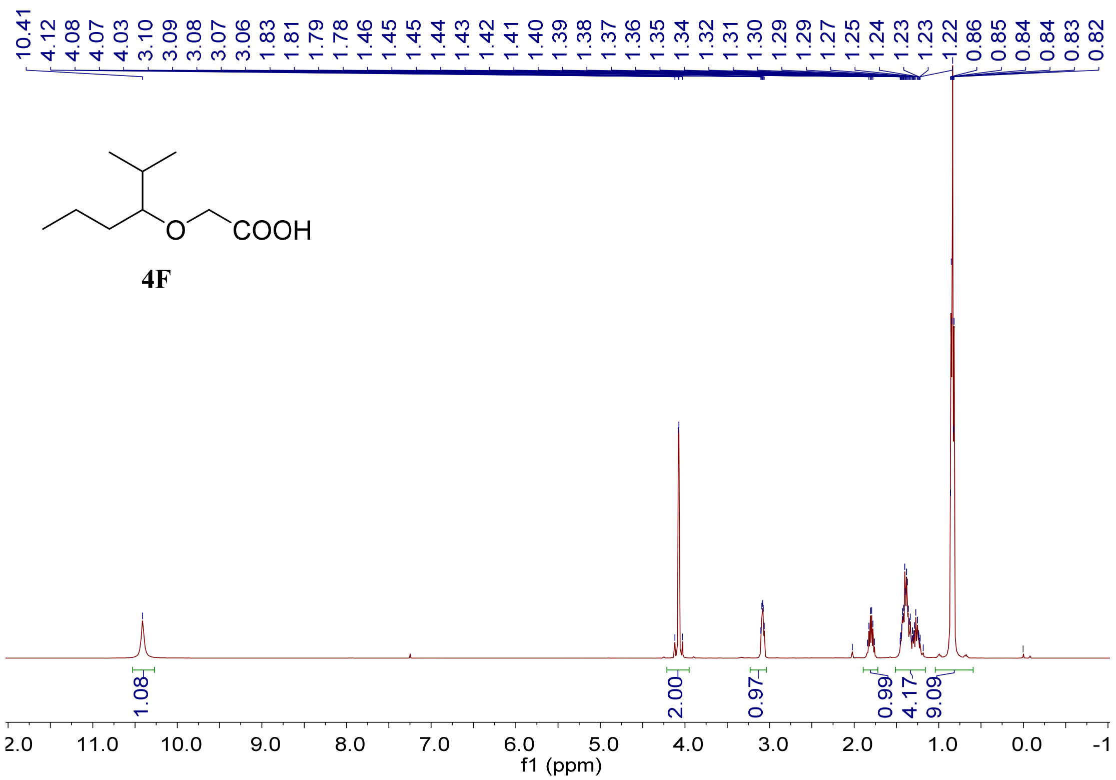

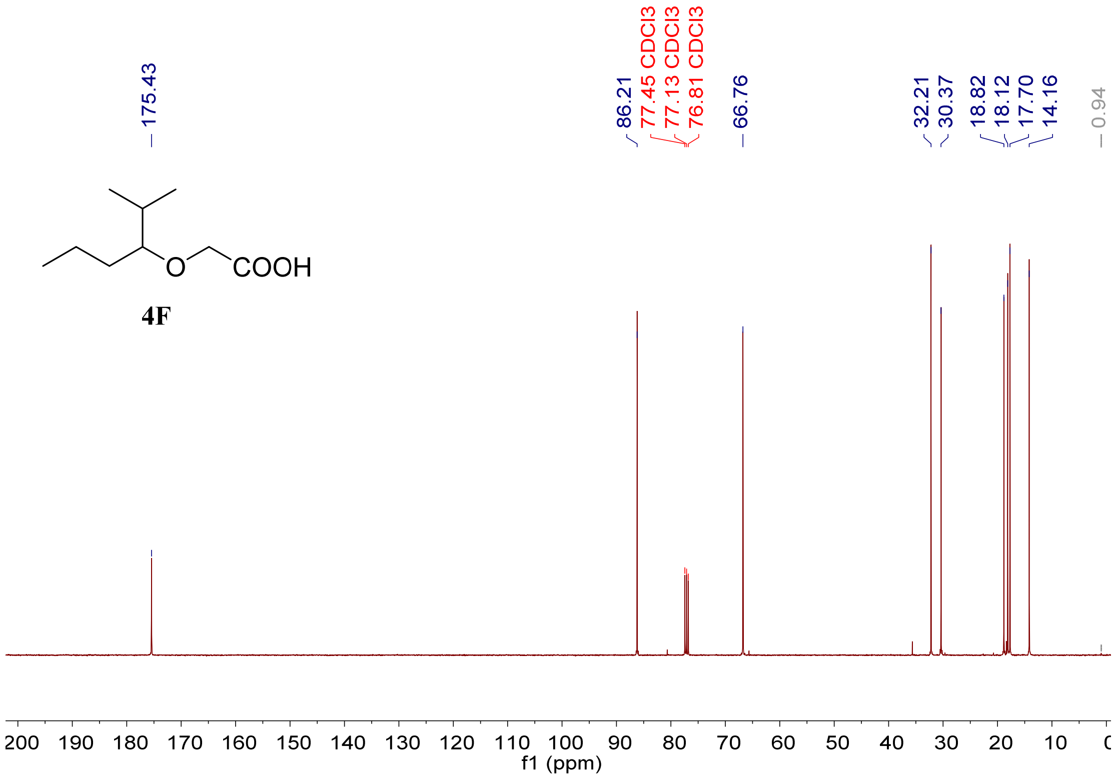

Supplementary Fig. 33. NMR spectra of 2-((2-Methylhexan-3-yl)oxy)acetic acid (4F).

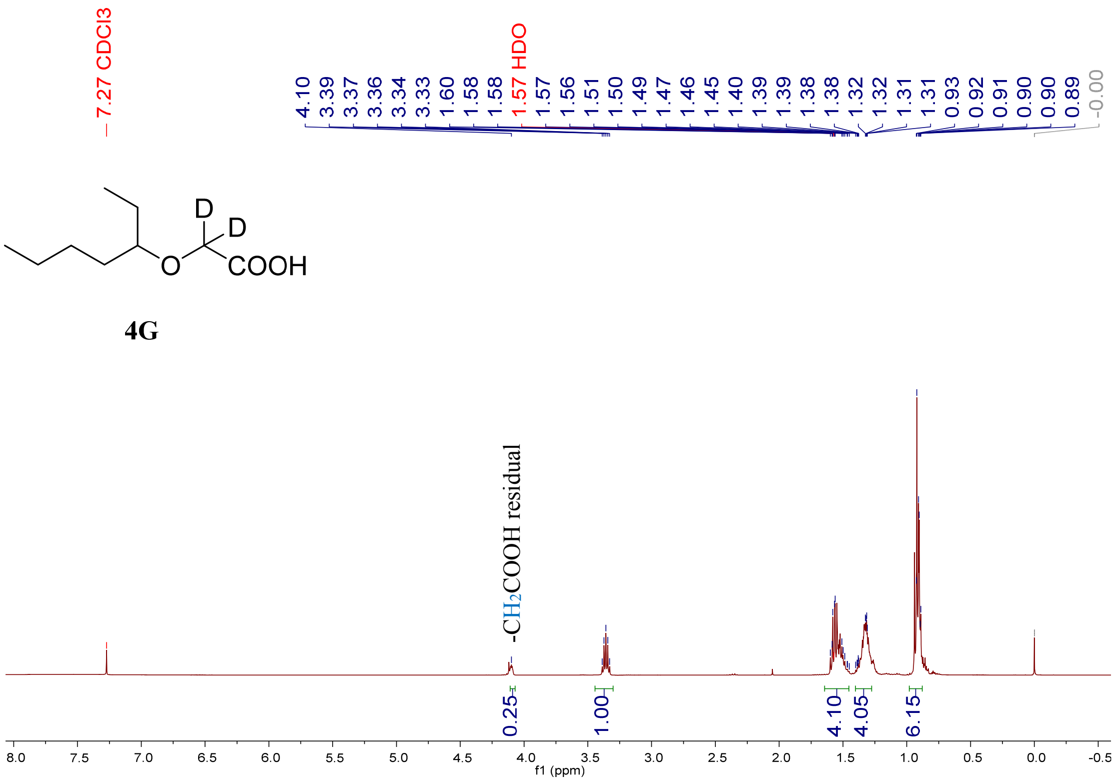

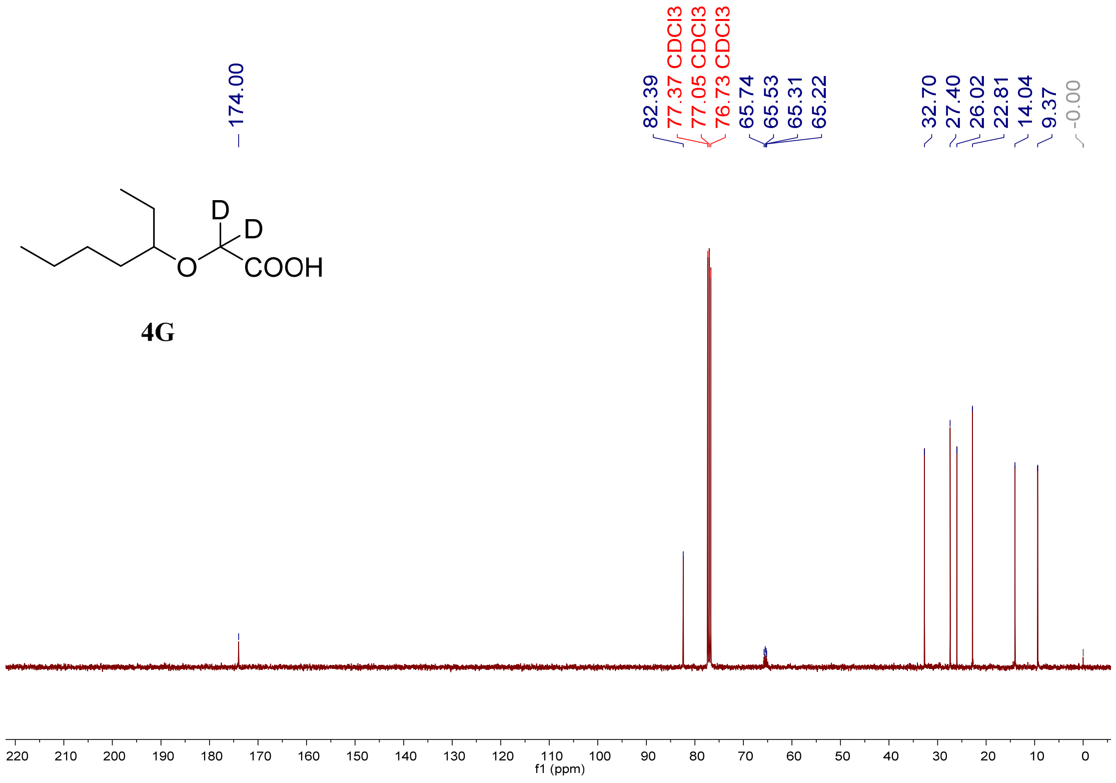

Supplementary Fig. 34. NMR spectra of 2-(Heptan-3-yloxy)acetic-2,2-d2 acid (4G).

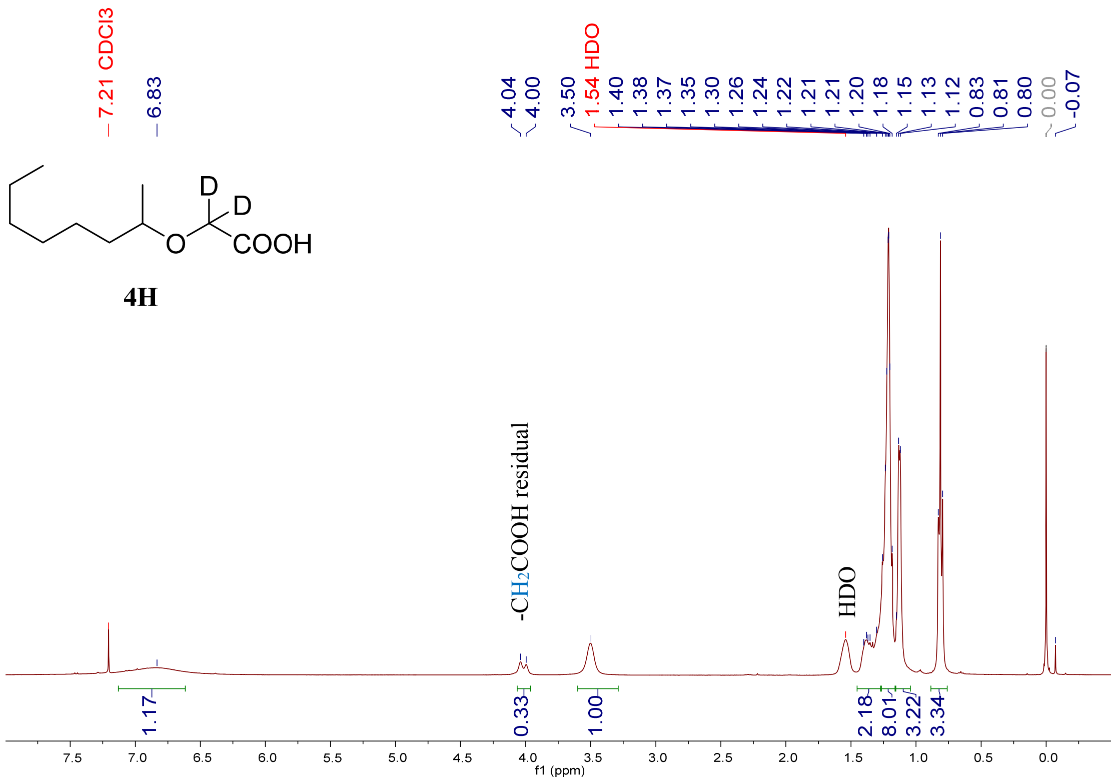

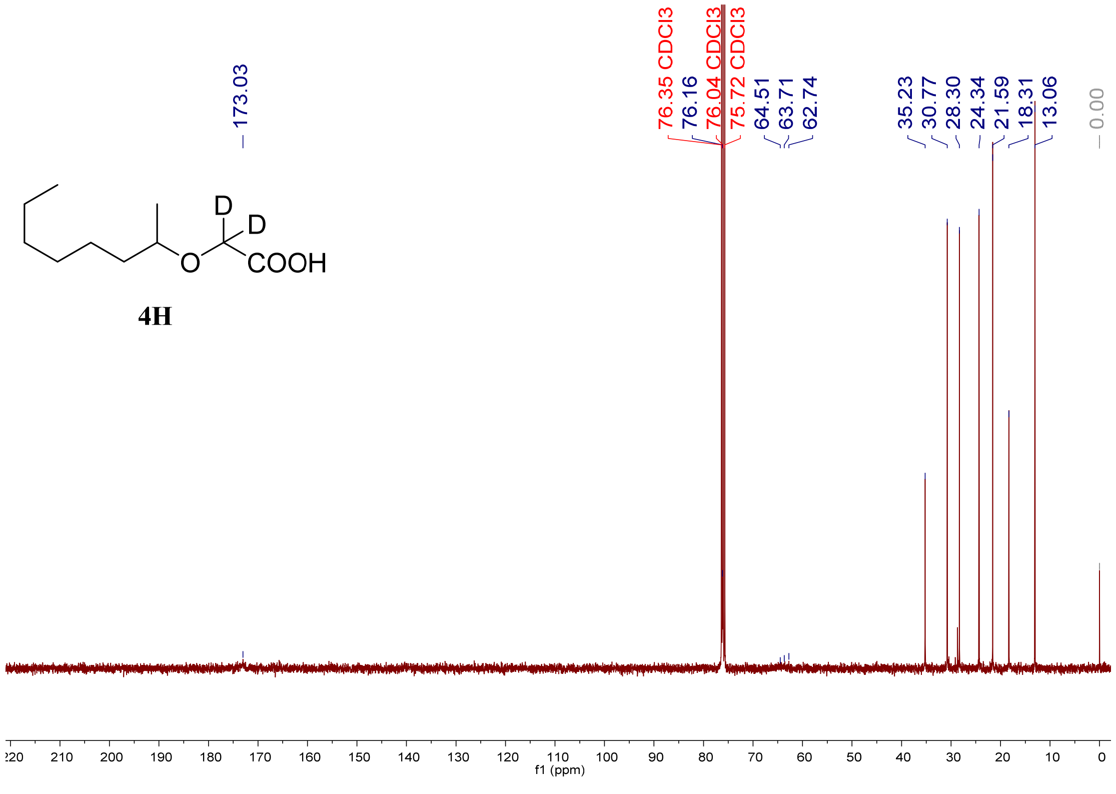

Supplementary Fig. 35. NMR spectra of 2-(Octan-2-yloxy)acetic-2,2-d2 acid (4H).

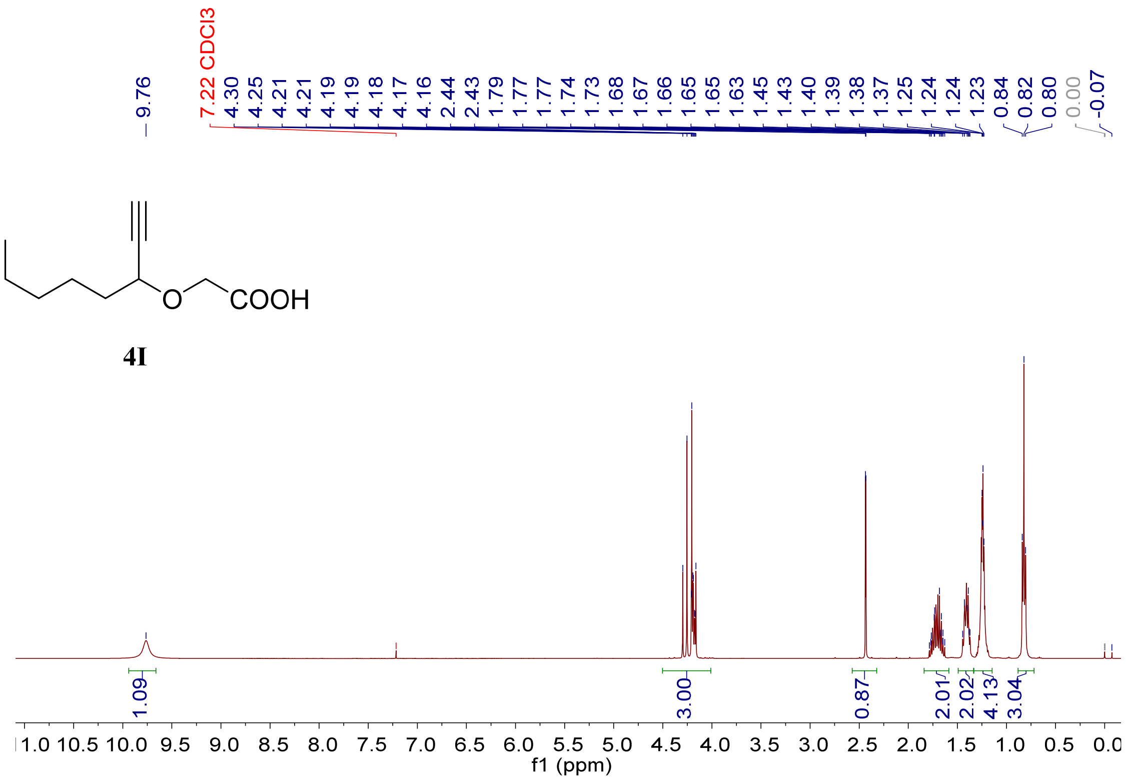

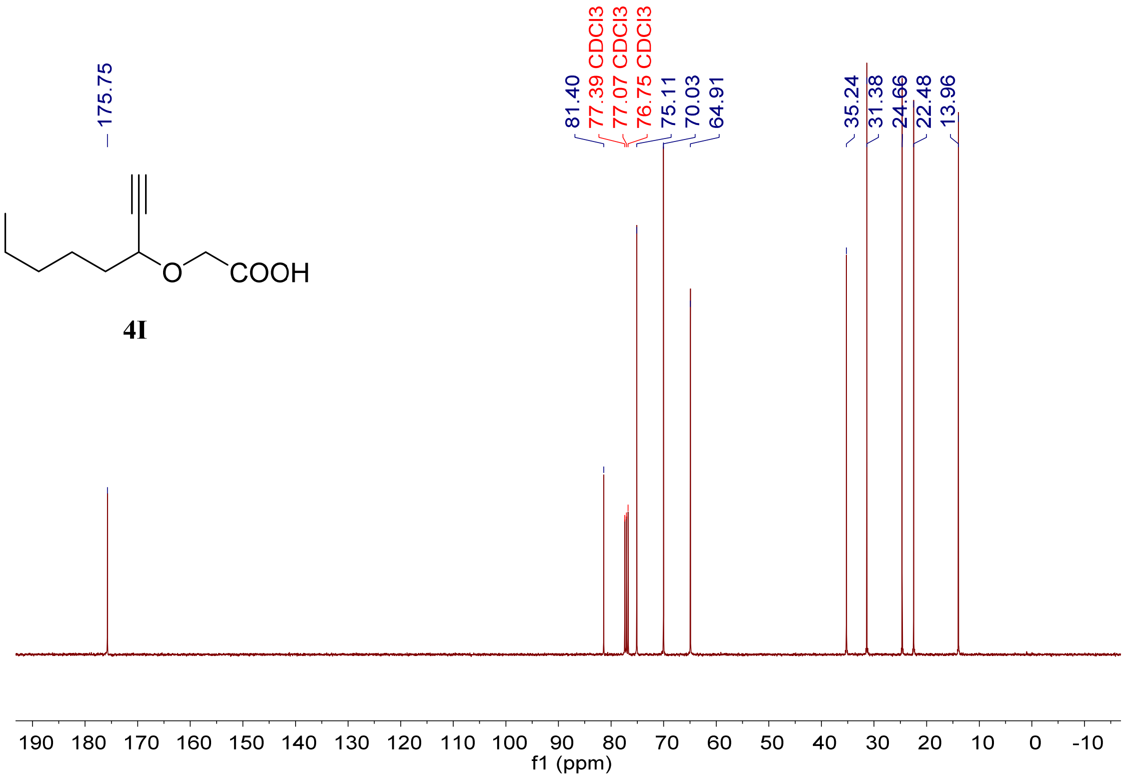

Supplementary Fig. 36. NMR spectra of 2-(Oct-1-yn-3-yloxy)acetic acid (4I).

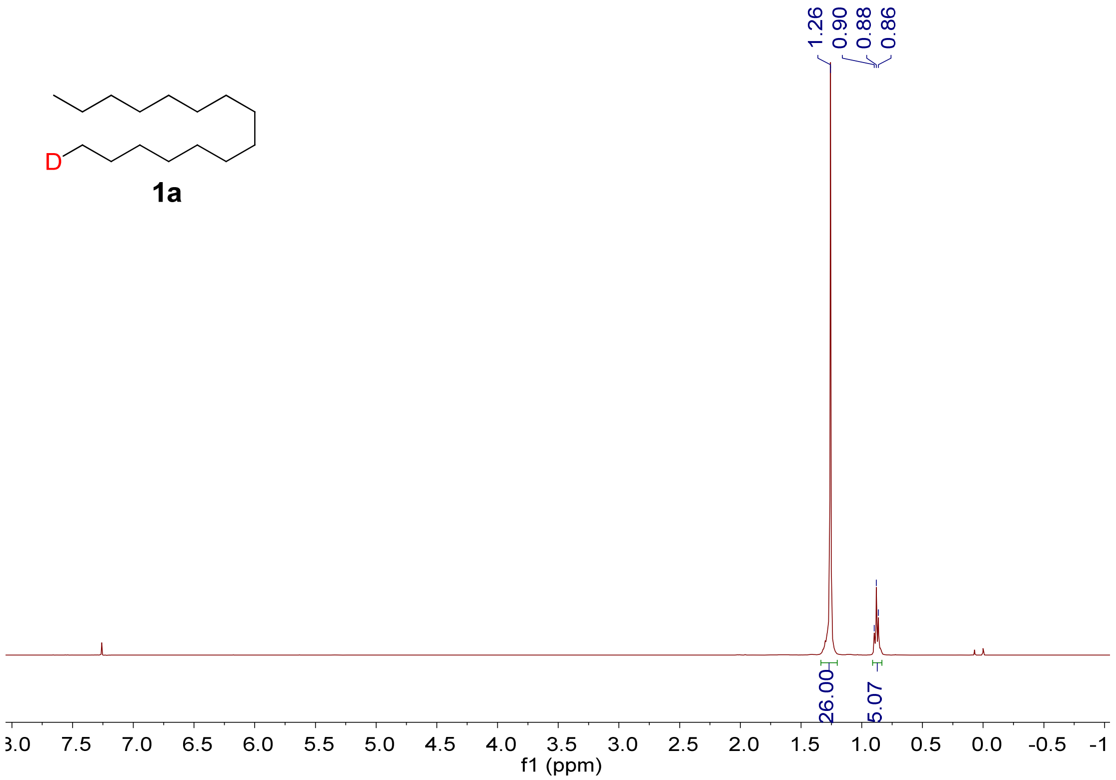
1.26
0.90
0.88
0.86
D
1a
26.00
5.07
f1 (ppm)

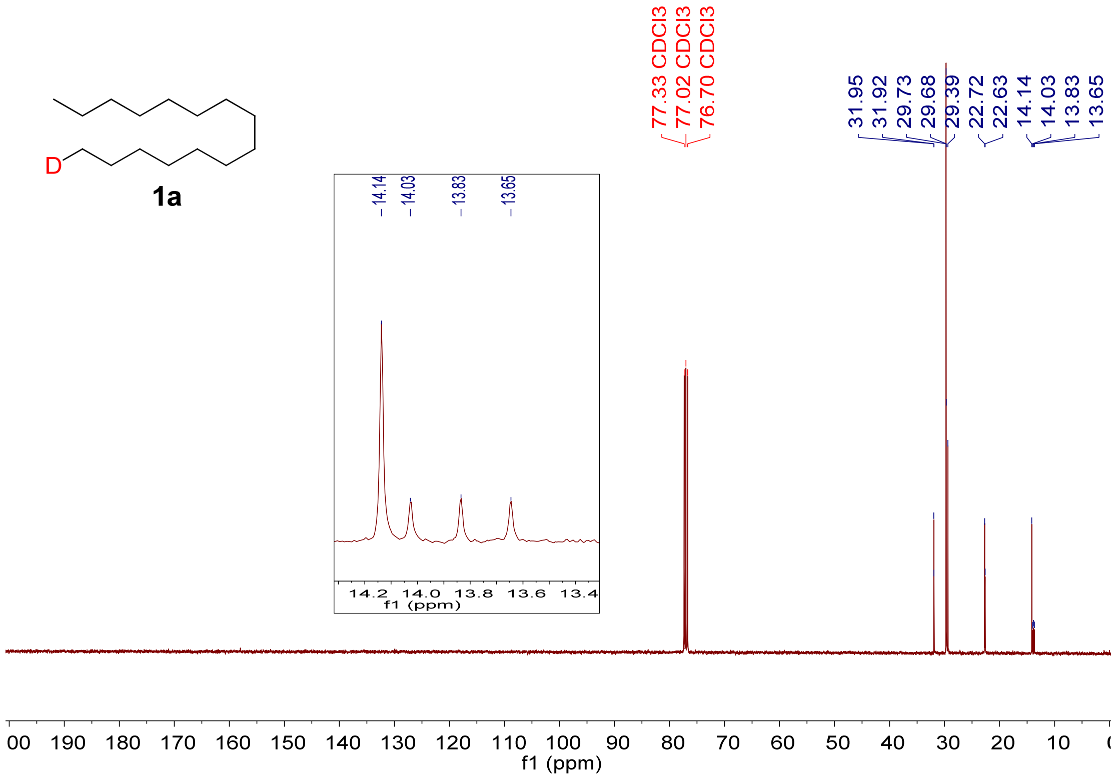
77.33 CDCl3
77.02 CDCl3
76.70 CDCl3
31.95
31.92
29.73
29.68
29.39
22.72
22.63
14.14
14.03
13.83
13.65
D
1a
f1 (ppm)

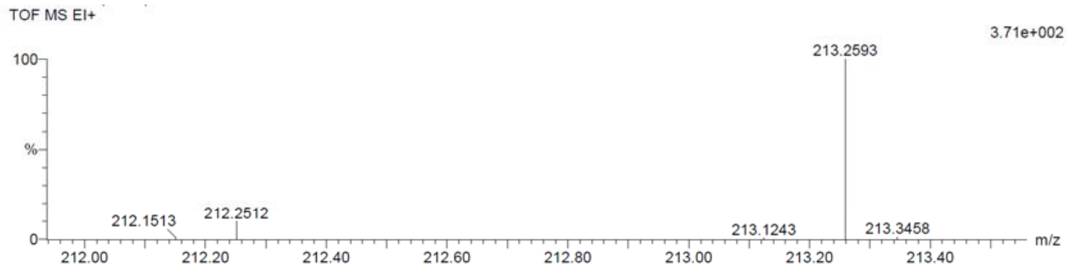

Minimum:	5.00				-1.5		
Maximum:	100.00		1.0	10.0	50.0		
Mass	RA	Calc. Mass	mDa	PPM	DBE	i-FIT	Formula
212.2512	9.84	212.2504	0.8	3.8	0.0	2773192.0	C15 1H32
213.2593	100.00	---					

Supplementary Fig. 37. NMR and HRMS spectra of Pentadecane-1-d (1a). The splitting of C-D coupling in ^{13}C-NMR was shown with an enlarged view.

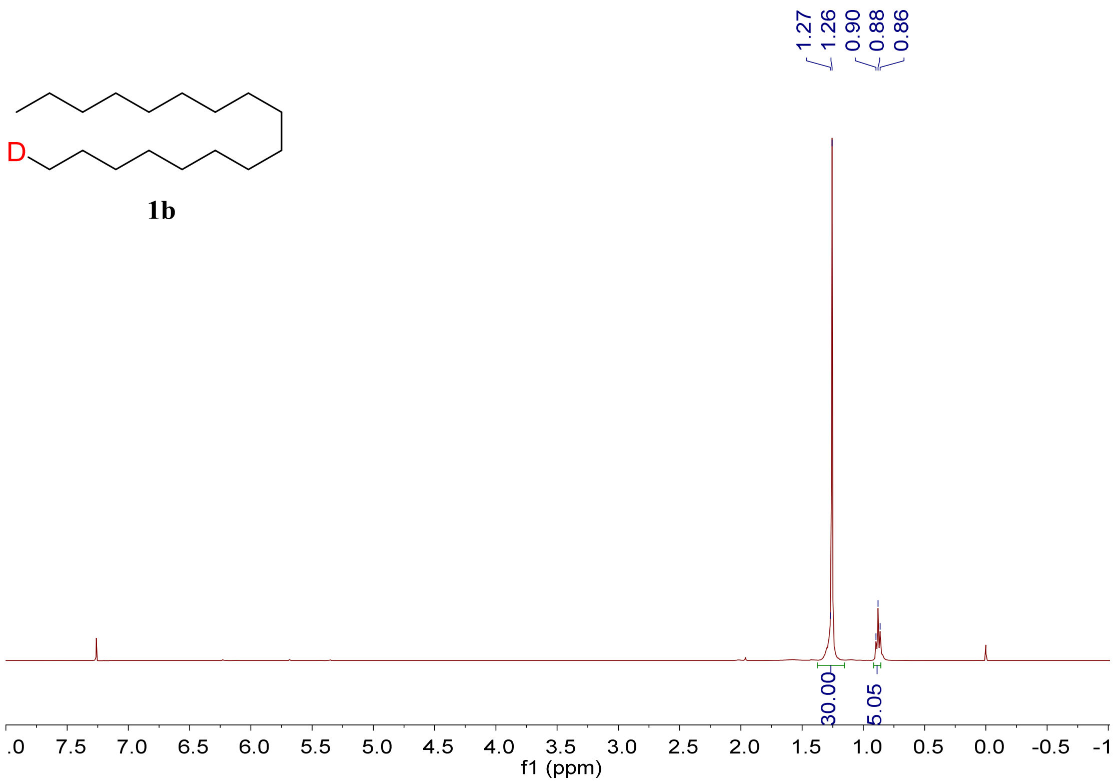

D
1b
1.27
1.26
0.90
0.88
0.86
30.00
5.05
f1 (ppm)

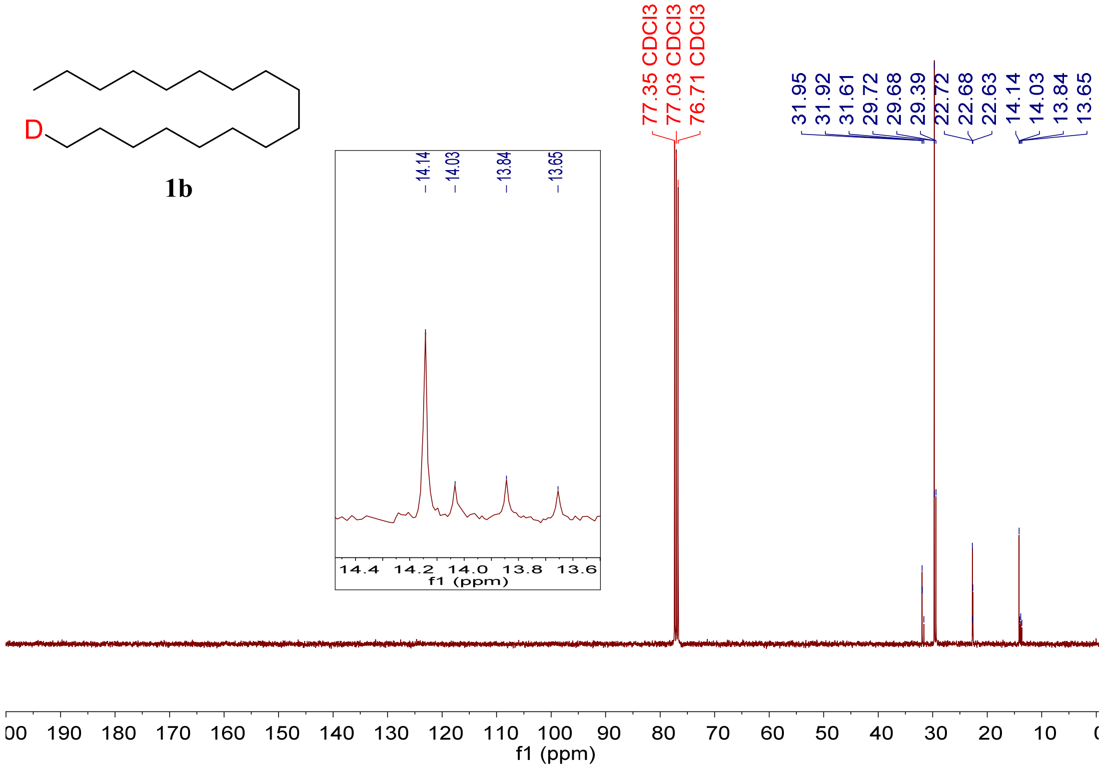

D
1b
77.35 CDCl3
77.03 CDCl3
76.71 CDCl3
31.95
31.92
31.61
29.72
29.68
29.39
22.72
22.68
22.63
14.14
14.03
13.84
13.65
f1 (ppm)

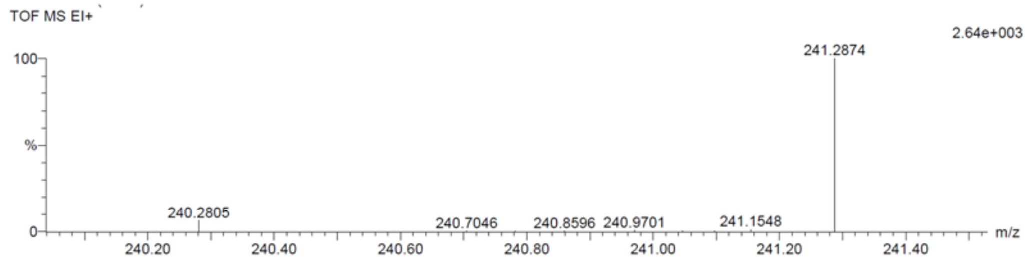

Minimum:	5.00				-1.5		
Maximum:	100.00		2.0	10.0	50.0		
Mass	RA	Calc. Mass	mDa	PPM	DBE	i-FIT	Formula
240.2805	6.22	240.2817	-1.2	-5.0	0.0	2774299.5	C17 1H36
		240.2802	0.3	1.2	0.5	2774320.5	C17 1H34 2H
241.2874	100.00	241.2880	-0.6	-2.5	0.0	5547276.5	C17 1H35 2H

Supplementary Fig. 38. NMR and HRMS spectra of Heptadecane-1-d (1b). The splitting of C-D coupling in ^{13}C-NMR was shown with an enlarged view.

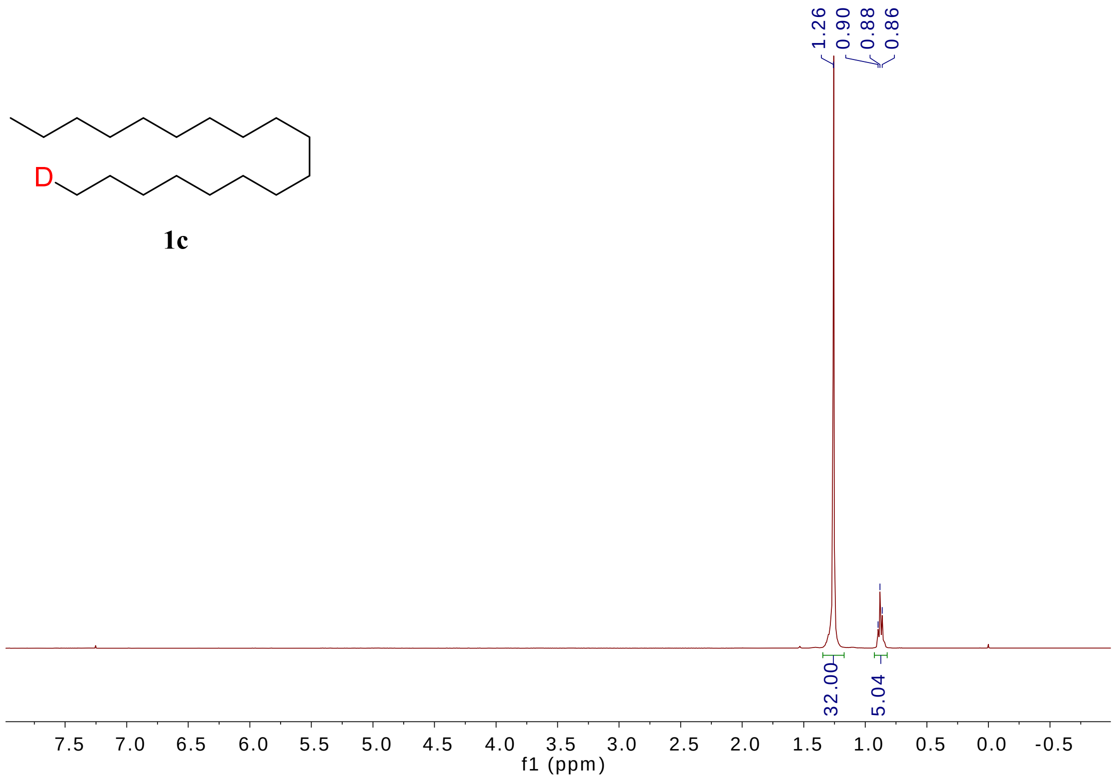
D
1c
1.26
0.90
0.88
0.86
32.00
5.04
7.5 7.0 6.5 6.0 5.5 5.0 4.5 4.0 3.5 3.0 2.5 2.0 1.5 1.0 0.5 0.0 -0.5
f1 (ppm)

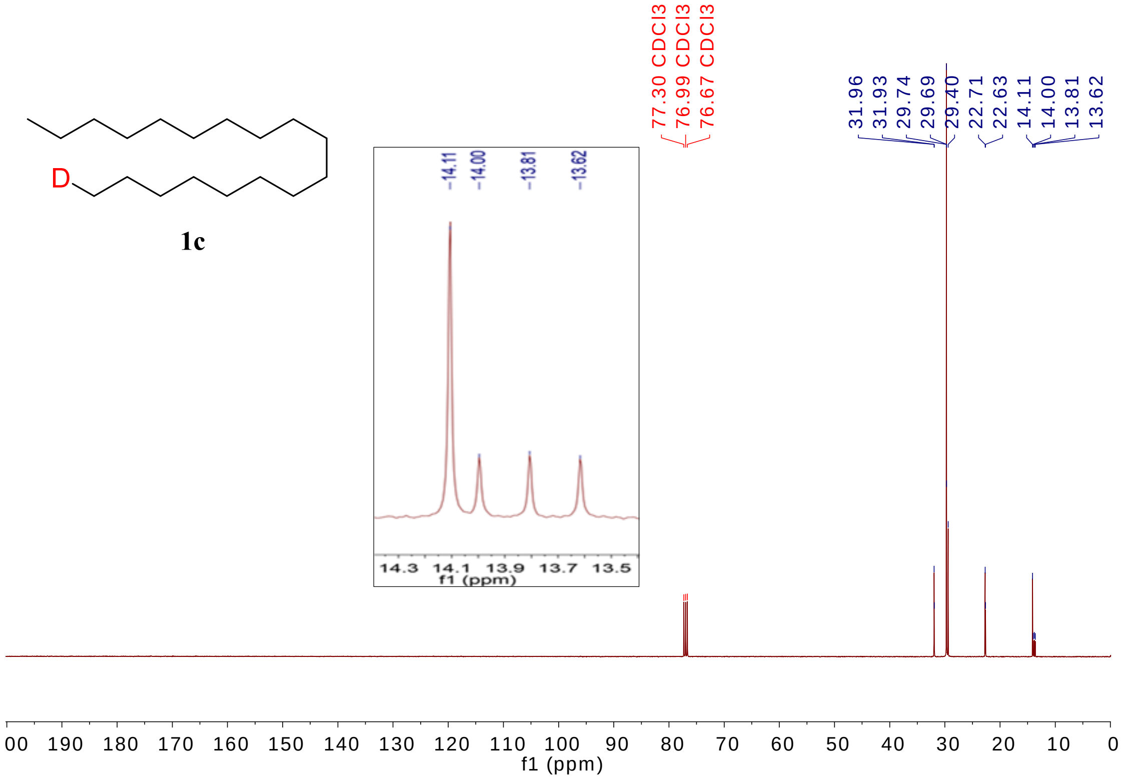
D
1c
77.30 CDCl3
76.99 CDCl3
76.67 CDCl3
31.96
31.93
29.74
29.69
29.40
22.71
22.63
14.11
14.00
13.81
13.62
14.3 14.1 13.9 13.7 13.5
f1 (ppm)
00 190 180 170 160 150 140 130 120 110 100 90 80 70 60 50 40 30 20 10 0
f1 (ppm)

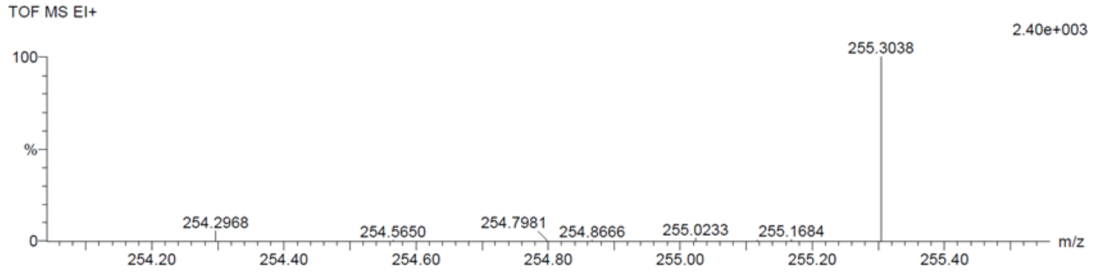

Minimum:	5.00				-1.5		
Maximum:	100.00		0.7	10.0	50.0		
Mass	RA	Calc. Mass	mDa	PPM	DBE	i-FIT	Formula
254.2968	5.11	254.2974	-0.6	-2.4	0.0	2774185.8	C18 1H38
255.3038	100.00	255.3036	0.2	0.8	0.0	5547164.0	C18 1H37 2H

Supplementary Fig. 39. NMR and HRMS spectra of Octadecane-1-d (1c). The splitting of C-D coupling in ^{13}C-NMR was shown with an enlarged view.

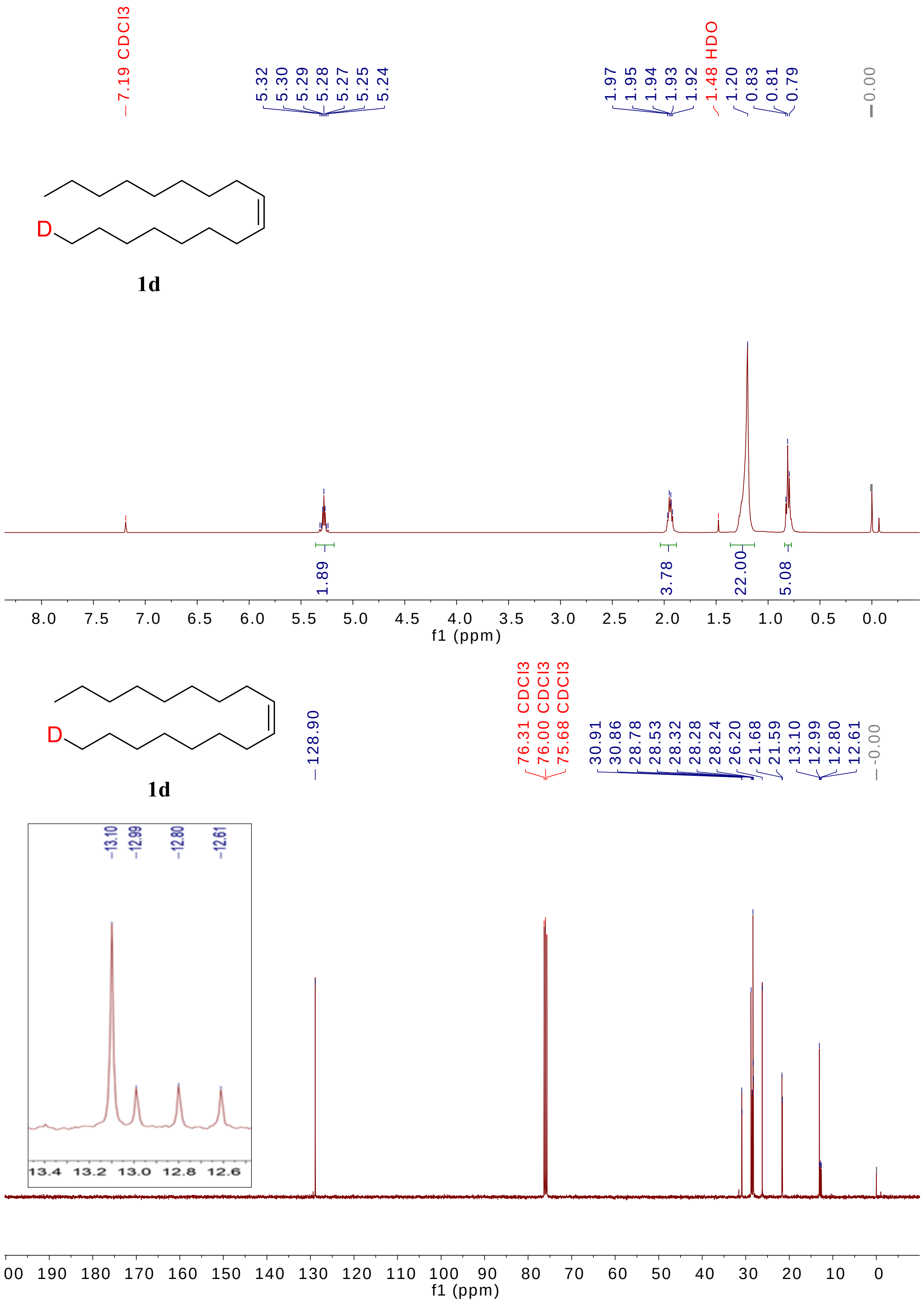

Supplementary Fig. 40. NMR spectra of (Z)-Heptadec-8-ene-1-d (1d). The splitting of C-D coupling in ^{13}C-NMR was shown with an enlarged view.

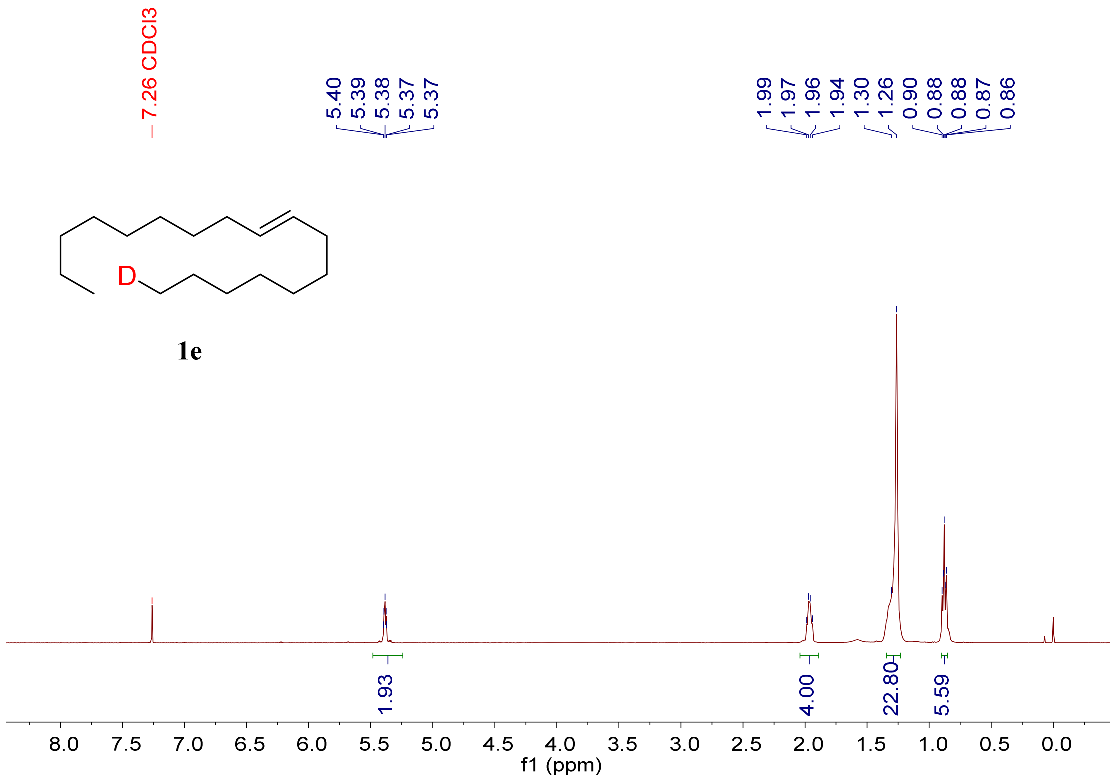

7.26 CDCl3
5.40
5.39
5.38
5.37
5.37
1.99
1.97
1.96
1.94
1.30
1.26
0.90
0.88
0.88
0.87
0.86
D
1e
1.93
4.00
22.80
5.59
8.0 7.5 7.0 6.5 6.0 5.5 5.0 4.5 4.0 3.5 3.0 2.5 2.0 1.5 1.0 0.5 0.0
f1 (ppm)

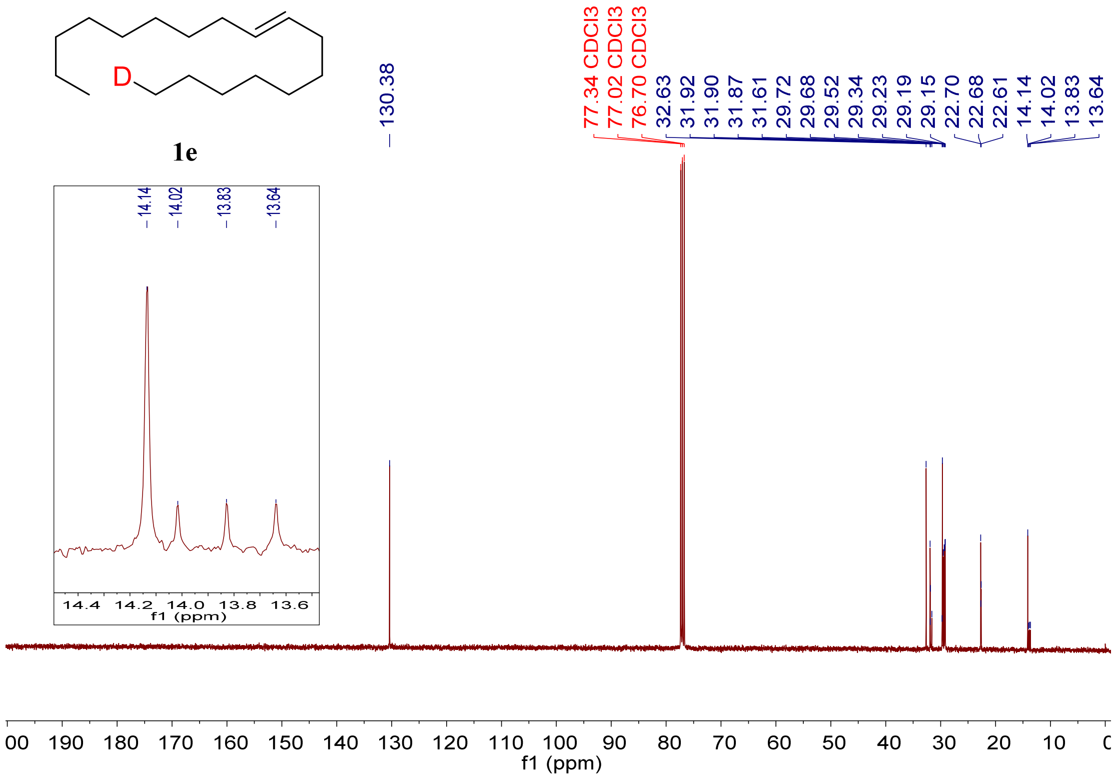

D
1e
130.38
77.34 CDCl3
77.02 CDCl3
76.70 CDCl3
32.63
31.92
31.90
31.87
31.61
29.72
29.68
29.52
29.34
29.23
29.19
29.15
22.70
22.68
22.61
14.14
14.02
13.83
13.64
14.4 14.2 14.0 13.8 13.6
f1 (ppm)
00 190 180 170 160 150 140 130 120 110 100 90 80 70 60 50 40 30 20 10 0
f1 (ppm)

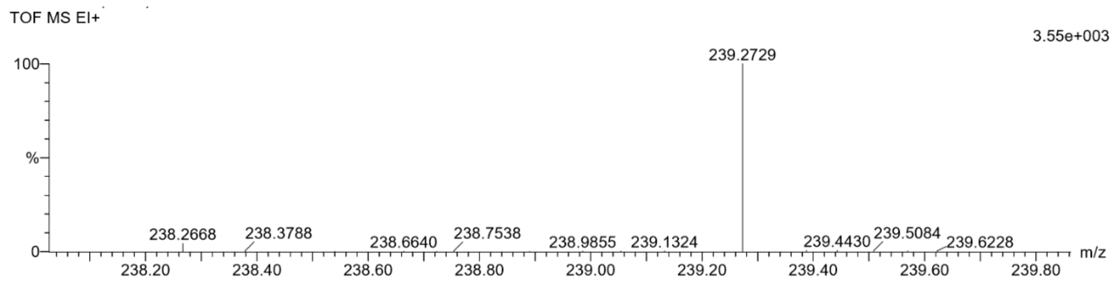

Minimum:	2.00				-1.5		
Maximum:	100.00		0.9	10.0	50.0		
Mass	RA	Calc. Mass	mDa	PPM	DBE	i-FIT	Formula
238.2668	4.31	238.2661	0.7	2.9	1.0	2774757.0	C17 1H34
239.2729	100.00	239.2723	0.6	2.5	1.0	5547731.5	C17 1H33 2H

Supplementary Fig. 41. NMR and HRMS spectra of (E)-Heptadec-8-ene-1-d (1e). The splitting of C-D coupling in ^{13}C-NMR was shown with an enlarged view.

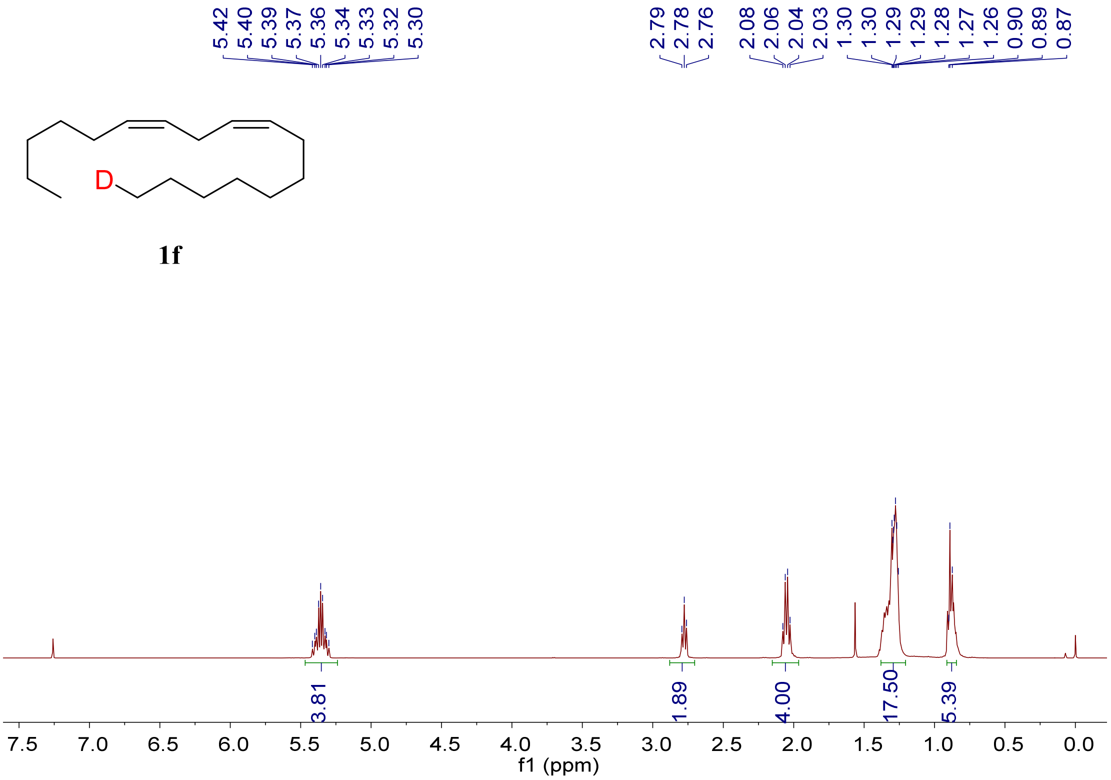
5.42 5.40 5.39 5.37 5.36 5.34 5.33 5.32 5.30
2.79 2.78 2.76
2.08 2.06 2.04 2.03 1.30 1.30 1.29 1.29 1.28 1.27 1.26 0.90 0.89 0.87
D
1f
3.81
1.89
4.00
17.50
5.39
7.5 7.0 6.5 6.0 5.5 5.0 4.5 4.0 3.5 3.0 2.5 2.0 1.5 1.0 0.5 0.0
f1 (ppm)

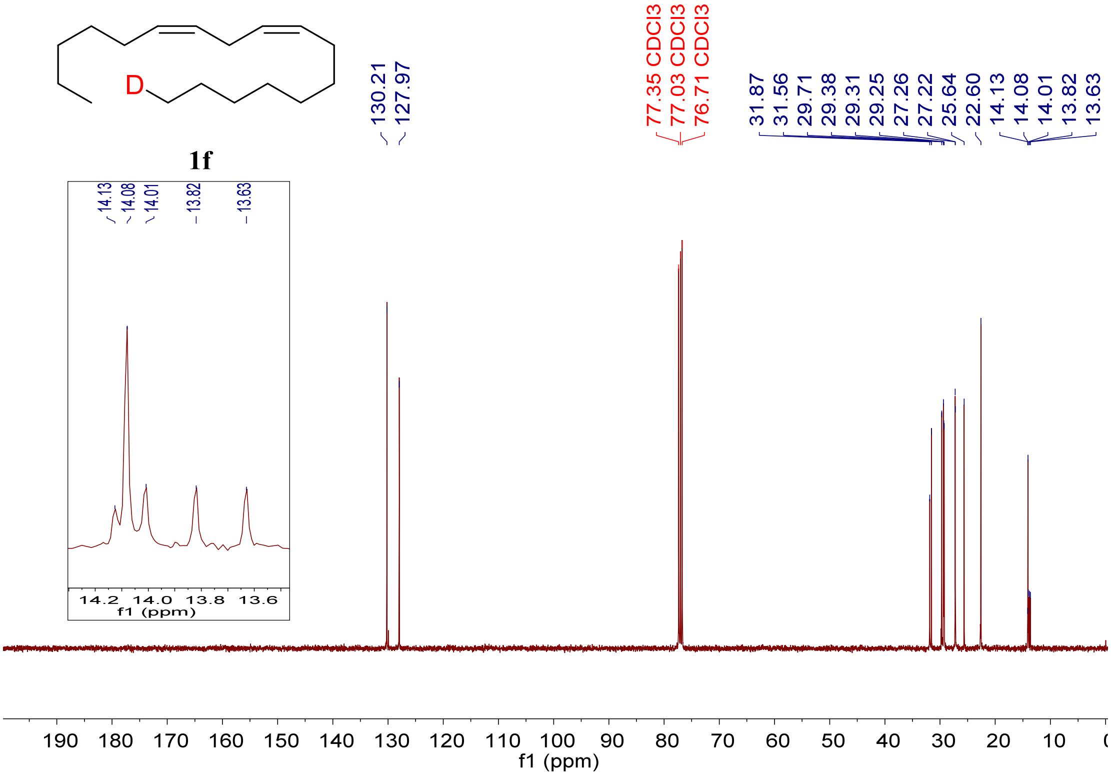
D
1f
130.21
127.97
77.35 CDCl3
77.03 CDCl3
76.71 CDCl3
31.87 31.56 29.71 29.38 29.31 29.25 27.26 27.22 25.64 22.60 14.13 14.08 14.01 13.82 13.63
14.13 14.08 14.01 13.82 13.63
14.2 14.0 13.8 13.6
f1 (ppm)
190 180 170 160 150 140 130 120 110 100 90 80 70 60 50 40 30 20 10
f1 (ppm)

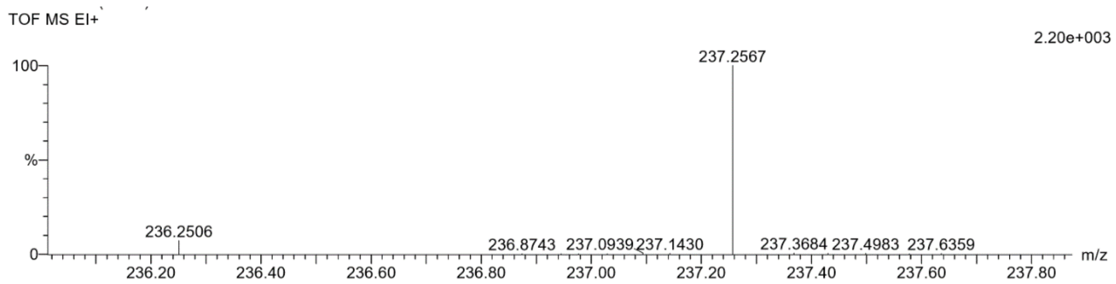

Minimum:	2.00				-1.5		
Maximum:	100.00		1.0	10.0	50.0		
Mass	RA	Calc. Mass	mDa	PPM	DBE	i-FIT	Formula
236.2506	7.10	236.2504	0.2	0.8	2.0	2774082.0	C17 1H32
237.2567	100.00	237.2567	0.0	0.0	2.0	5547058.0	C17 1H31 2H

Supplementary Fig. 42. NMR and HRMS spectra of (6Z,9Z)-Heptadeca-6,9-diene-17-d (1f). The splitting of C-D coupling in ^{13}C-NMR was shown with an enlarged view.

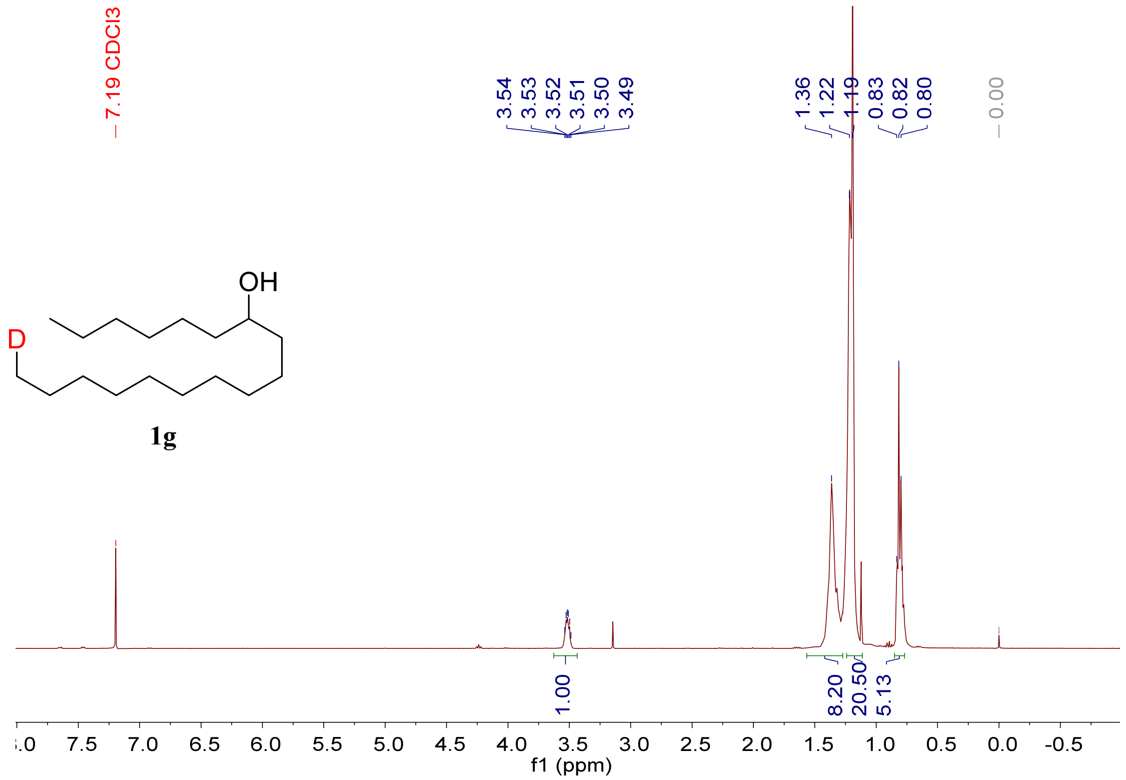

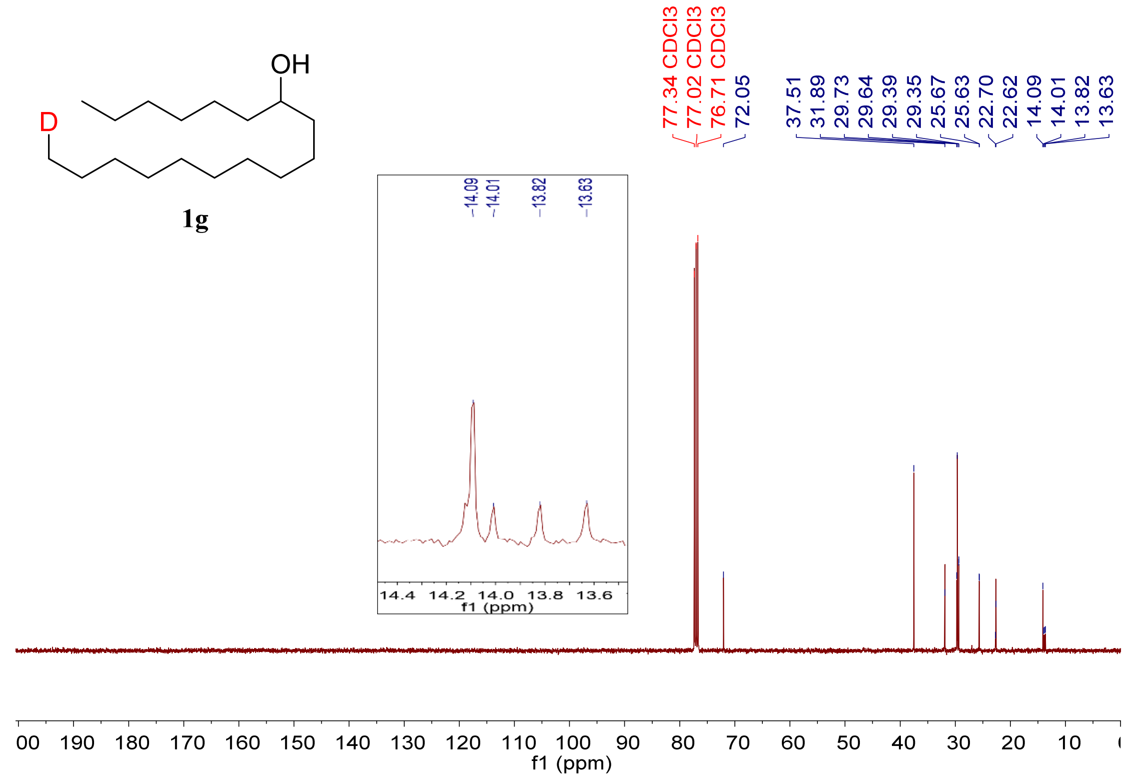

Supplementary Fig. 43. NMR spectra of Heptadecan-17-d-7-ol (1g). The splitting of C-D coupling in ^{13}C-NMR was shown with an enlarged view.

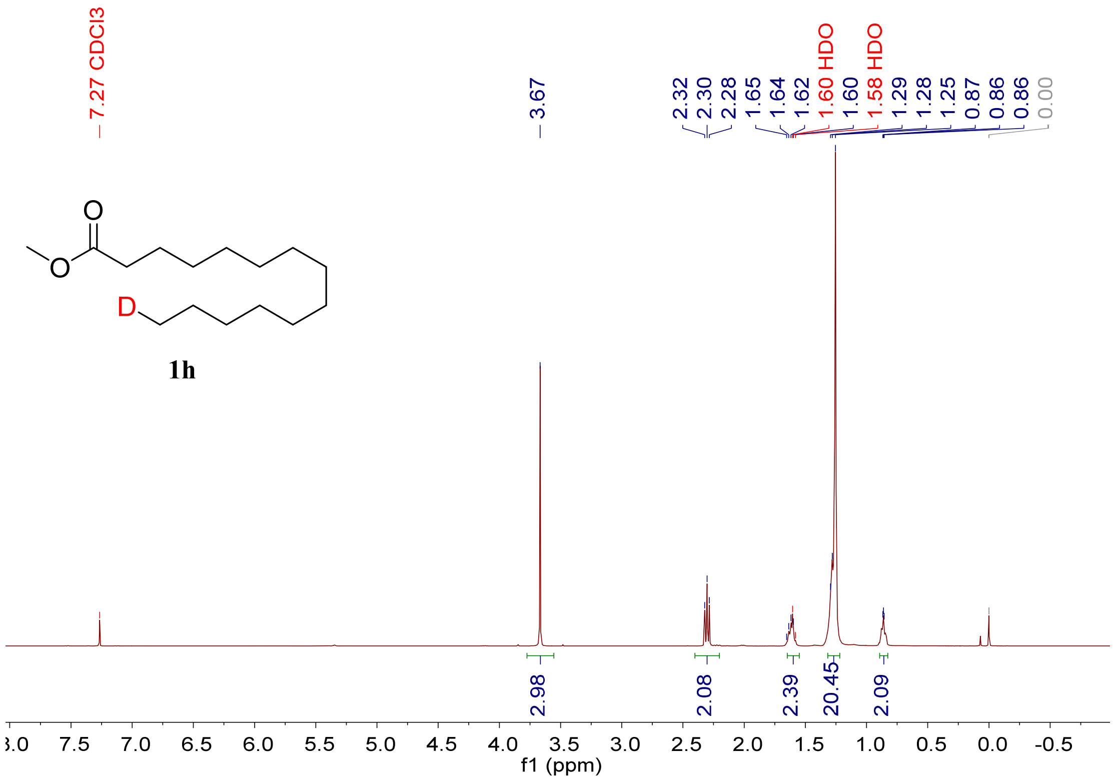

7.27 CDCl3
3.67
2.32
2.30
2.28
1.65
1.64
1.62
1.60 HDO
1.60
1.58 HDO
1.29
1.28
1.25
0.87
0.86
0.86
0.00
O
O
D
1h
2.98
2.08
2.39
20.45
2.09
f1 (ppm)

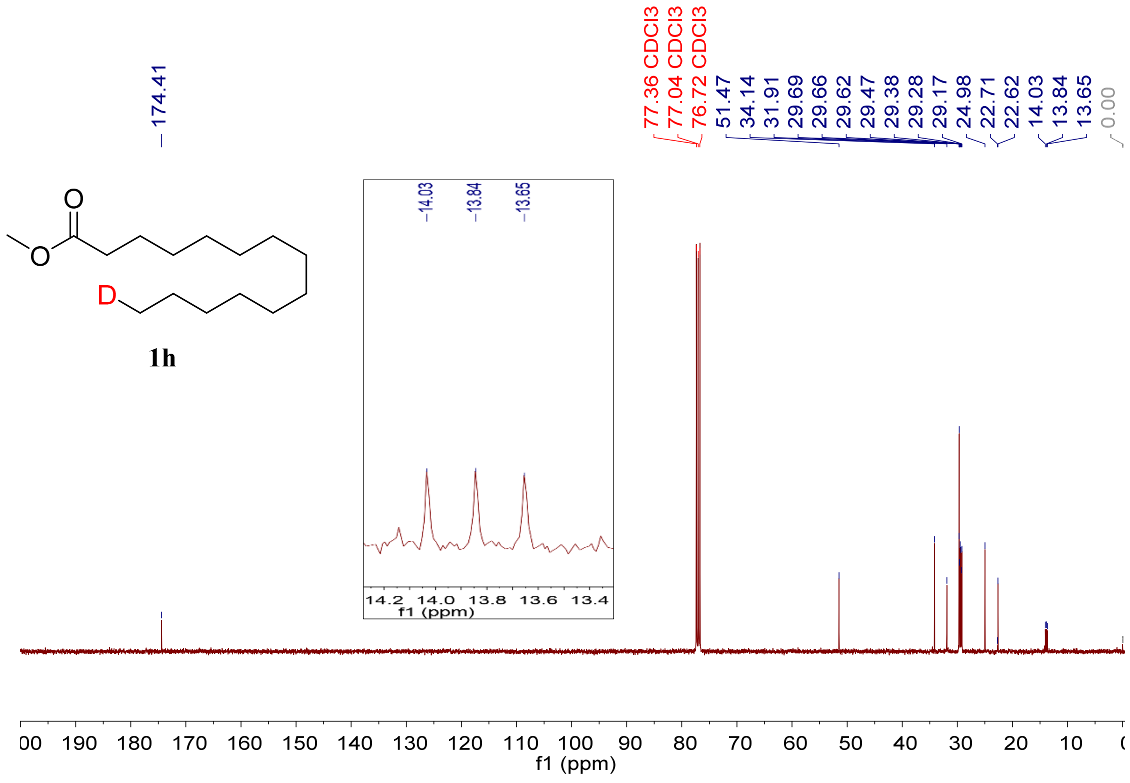

174.41
77.36 CDCl3
77.04 CDCl3
76.72 CDCl3
51.47
34.14
31.91
29.69
29.66
29.62
29.47
29.38
29.28
29.17
24.98
22.71
22.62
14.03
13.84
13.65
0.00
O
O
D
1h
f1 (ppm)

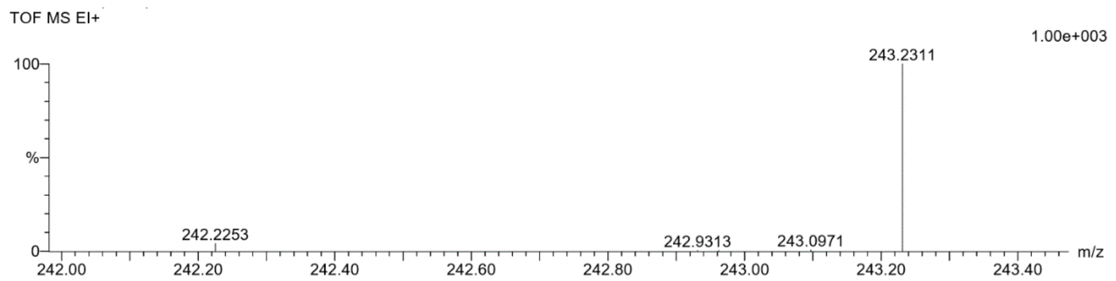

Minimum:	2.00				-1.5		
Maximum:	100.00		1.0	10.0	50.0		
Mass	RA	Calc. Mass	mDa	PPM	DBE	i-FIT	Formula
242.2253	3.95	242.2246	0.7	2.9	1.0	2773505.5	C15 1H30 O2
243.2311	100.00	243.2309	0.2	0.8	1.0	5546474.0	C15 1H29 2H O2

Supplementary Fig. 44. NMR and HRMS spectra of Methyl tetradecanoate-14-d (1h). The splitting of C-D coupling in ^{13}C-NMR was shown with an enlarged view.

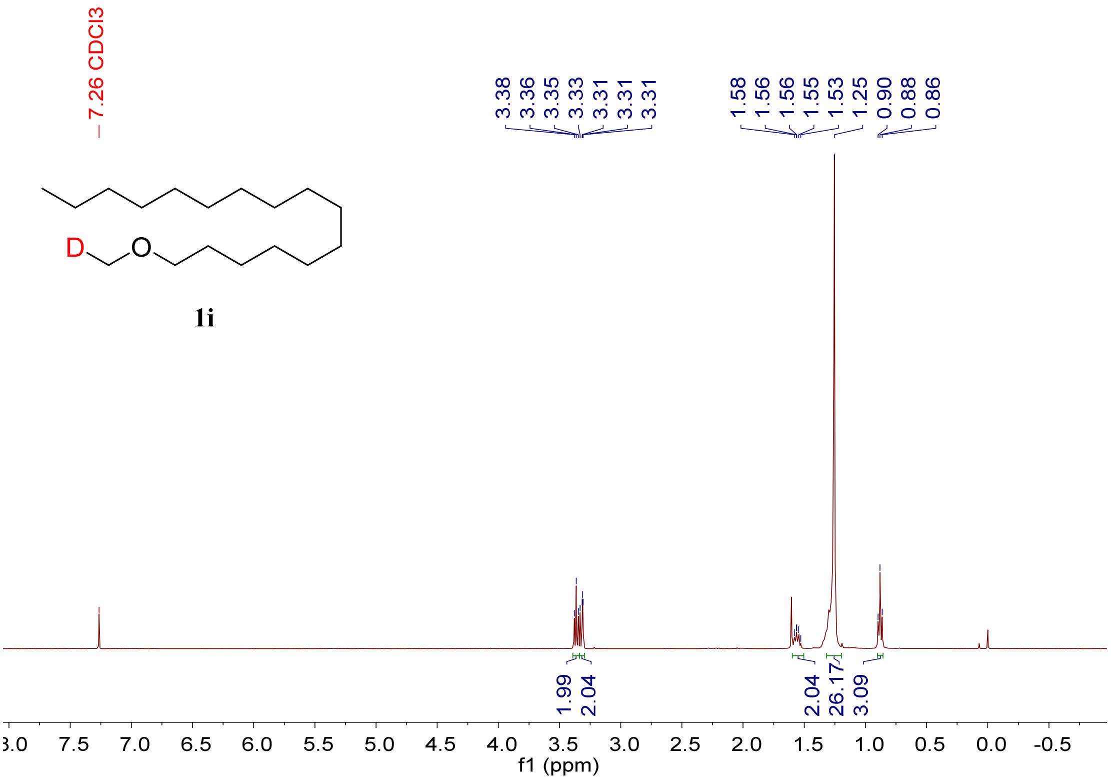

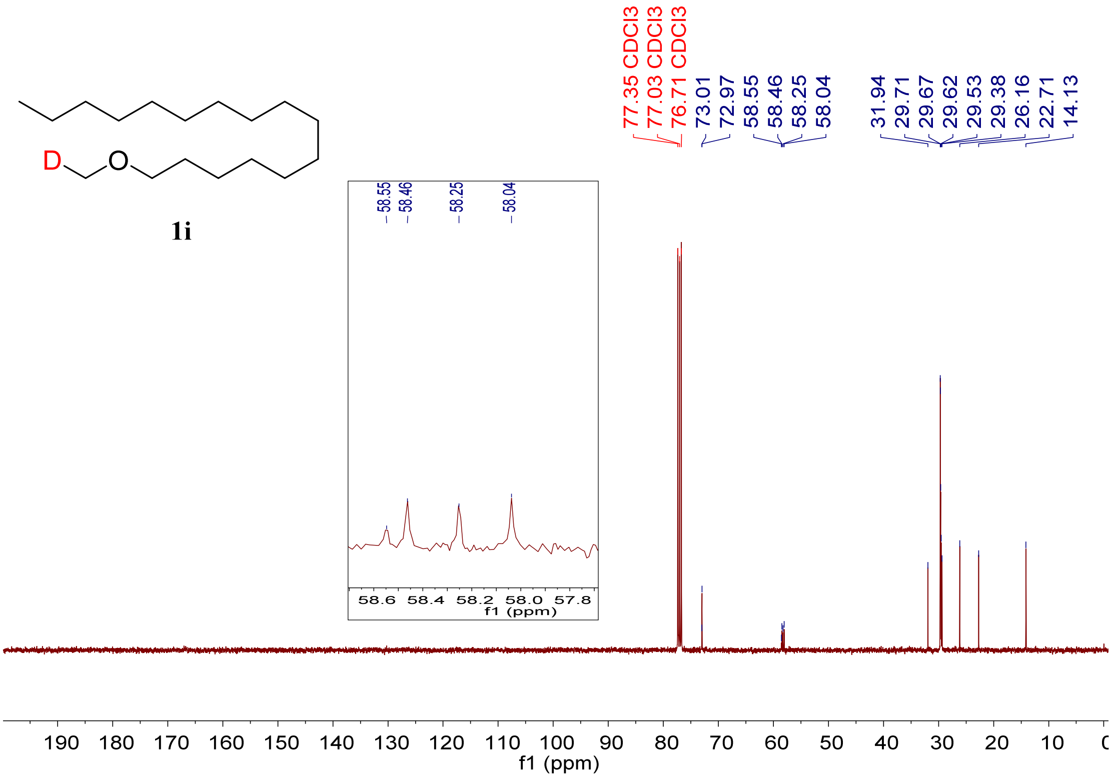

Supplementary Fig. 45. NMR spectra of 1-(Methoxy-d)hexadecane (1i). The splitting of C-D coupling in ^{13}C-NMR was shown with an enlarged view.

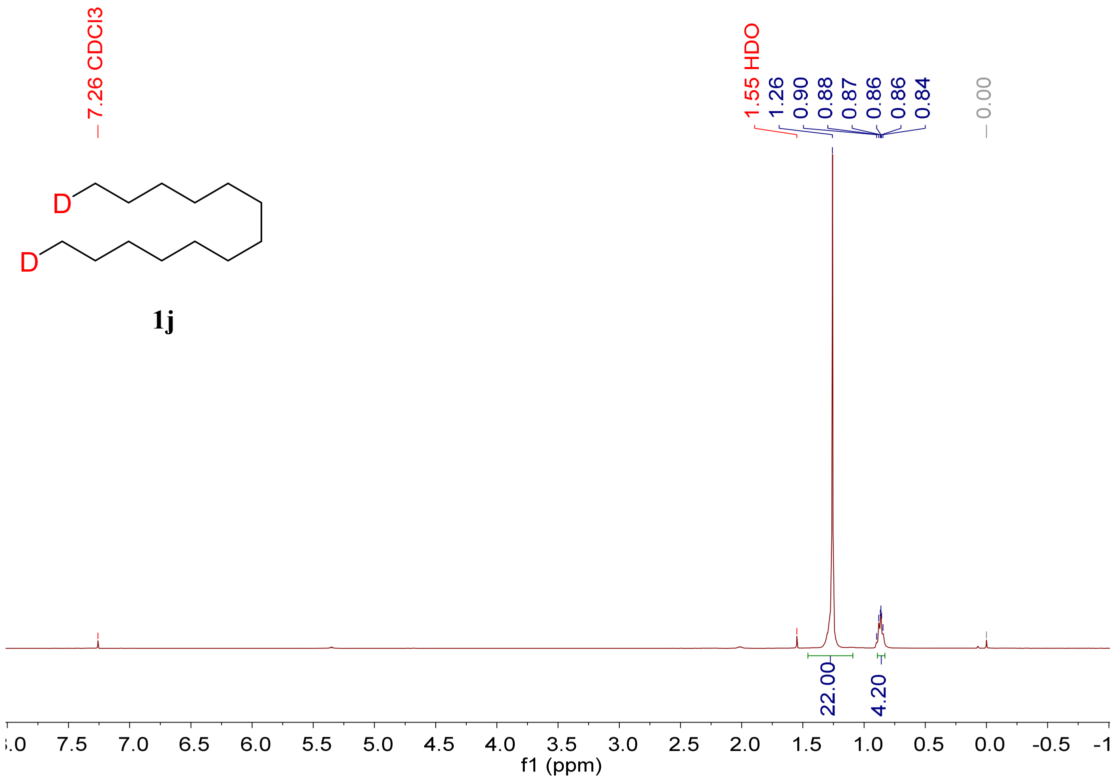
7.26 CDCl3
1.55 HDO
1.26
0.90
0.88
0.87
0.86
0.86
0.84
0.00
D
D
22.00
4.20
f1 (ppm)

1j

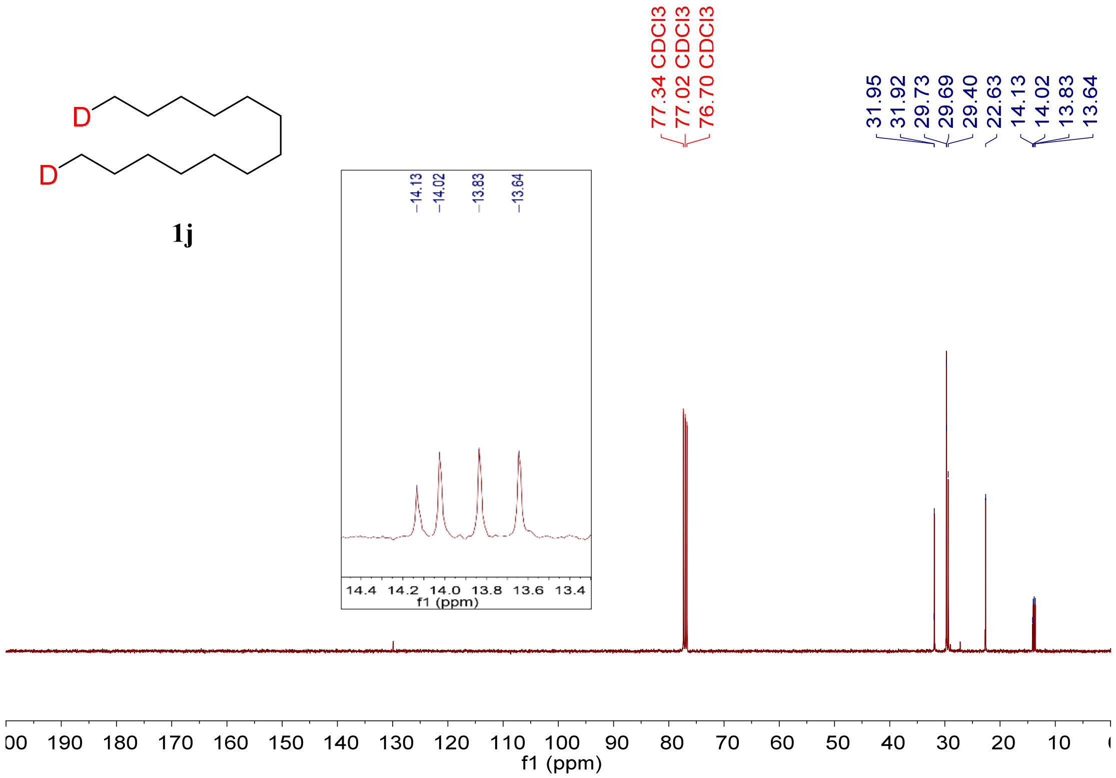
77.34 CDCl3
77.02 CDCl3
76.70 CDCl3
31.95
31.92
29.73
29.69
29.40
22.63
14.13
14.02
13.83
13.64
D
D
f1 (ppm)

1j

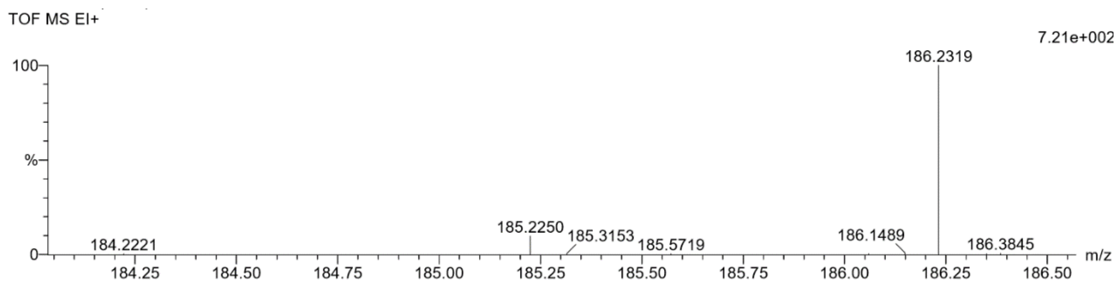

Minimum:	5.00				-1.5		
Maximum:	100.00		1.0	10.0	50.0		
Mass	RA	Calc. Mass	mDa	PPM	DBE	i-FIT	Formula
185.2250	9.69	185.2254	-0.4	-2.2	0.0	2773370.5	C13 1H27 2H
186.2319	100.00	186.2317	0.2	1.1	0.0	2773205.0	C13 1H26 2H2

Supplementary Fig. 46. NMR and HRMS spectra of Tridecane-1,13-d2 (1j). The splitting of C-D coupling in ^{13}C-NMR was shown with an enlarged view.

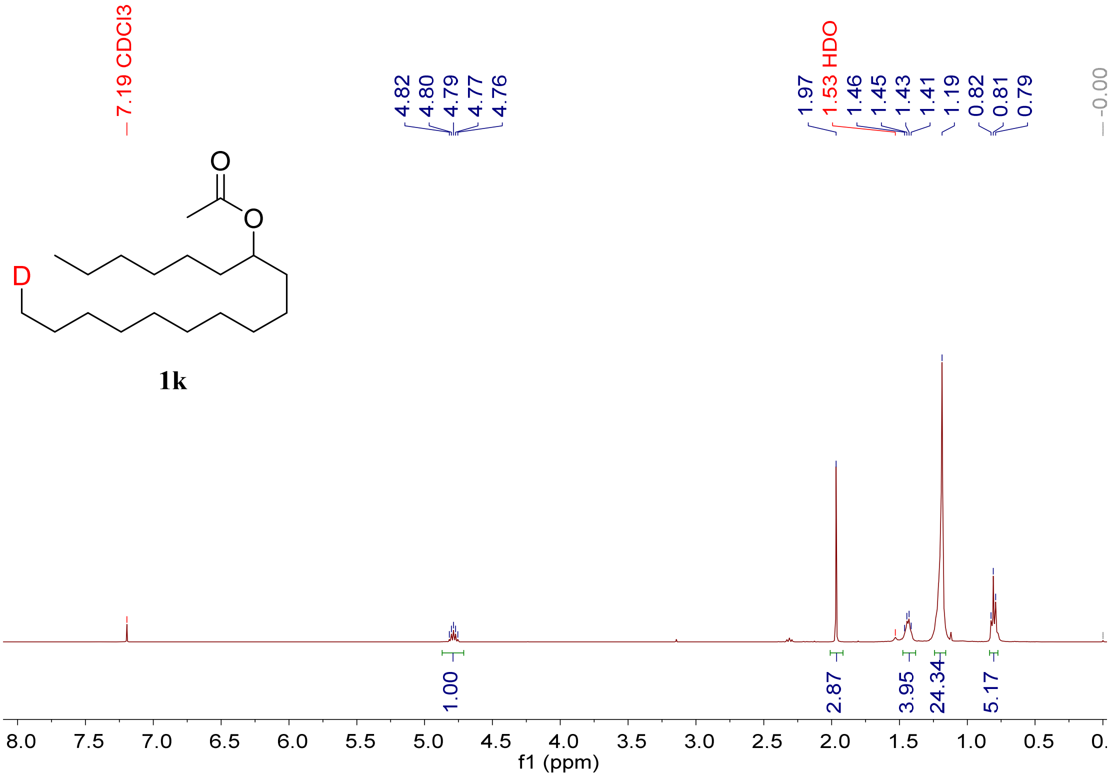

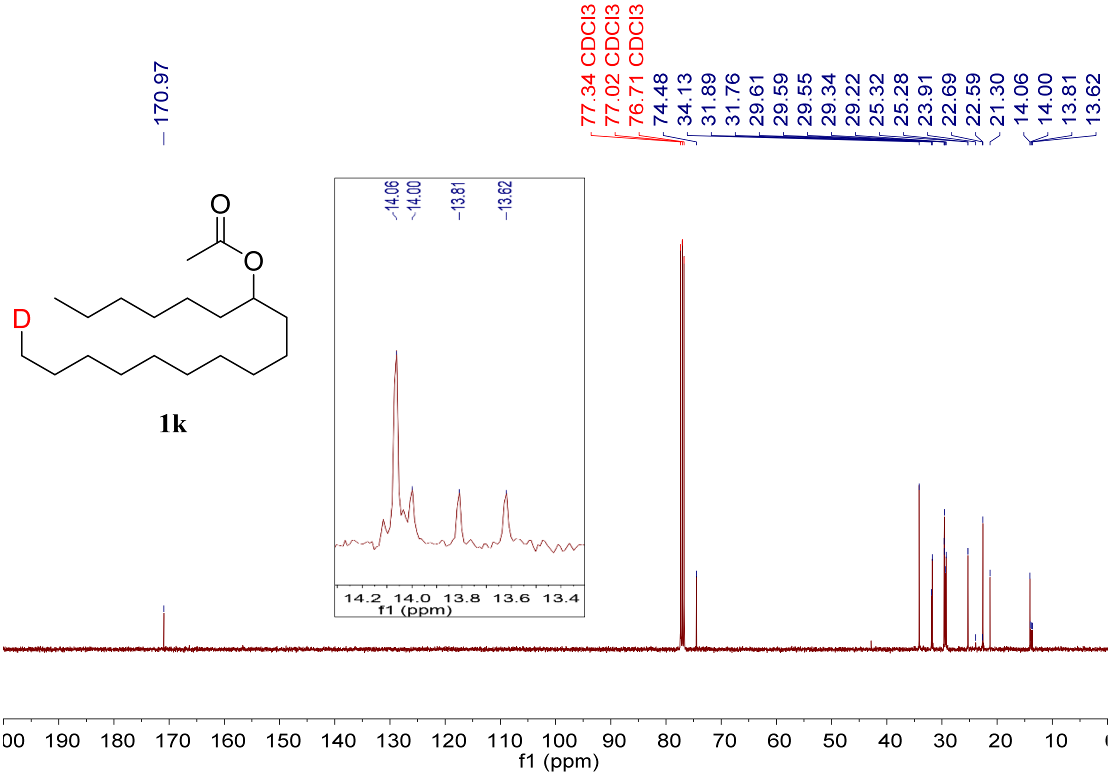

Supplementary Fig. 47. NMR spectra of Heptadecan-7-yl-17-d acetate (1k). The splitting of C-D coupling in ^{13}C-NMR was shown with an enlarged view.

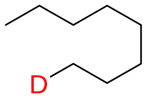

2a

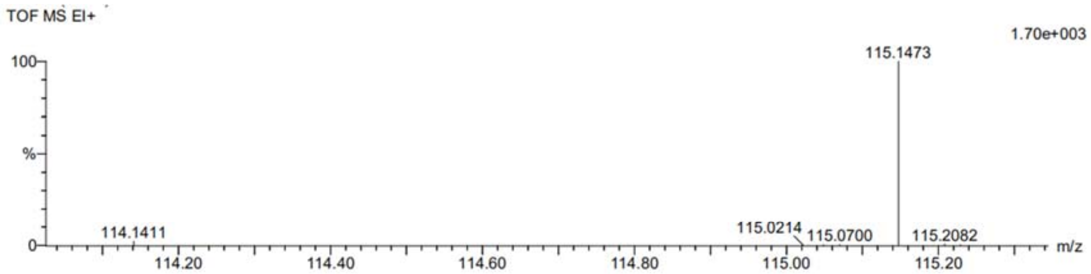

Minimum:	1.00				-1.5		
Maximum:	100.00		1.0	10.0	50.0		
Mass	RA	Calc. Mass	mDa	PPM	DBE	i-FIT	Formula
114.1411	2.03	114.1409	0.2	1.8	0.0	2773860.8	C8 1H18
115.1473	100.00	115.1471	0.2	1.7	0.0	2773621.0	C8 1H17 2H

Supplementary Fig. 48. HRMS spectrum of Octane-1-d (2a).

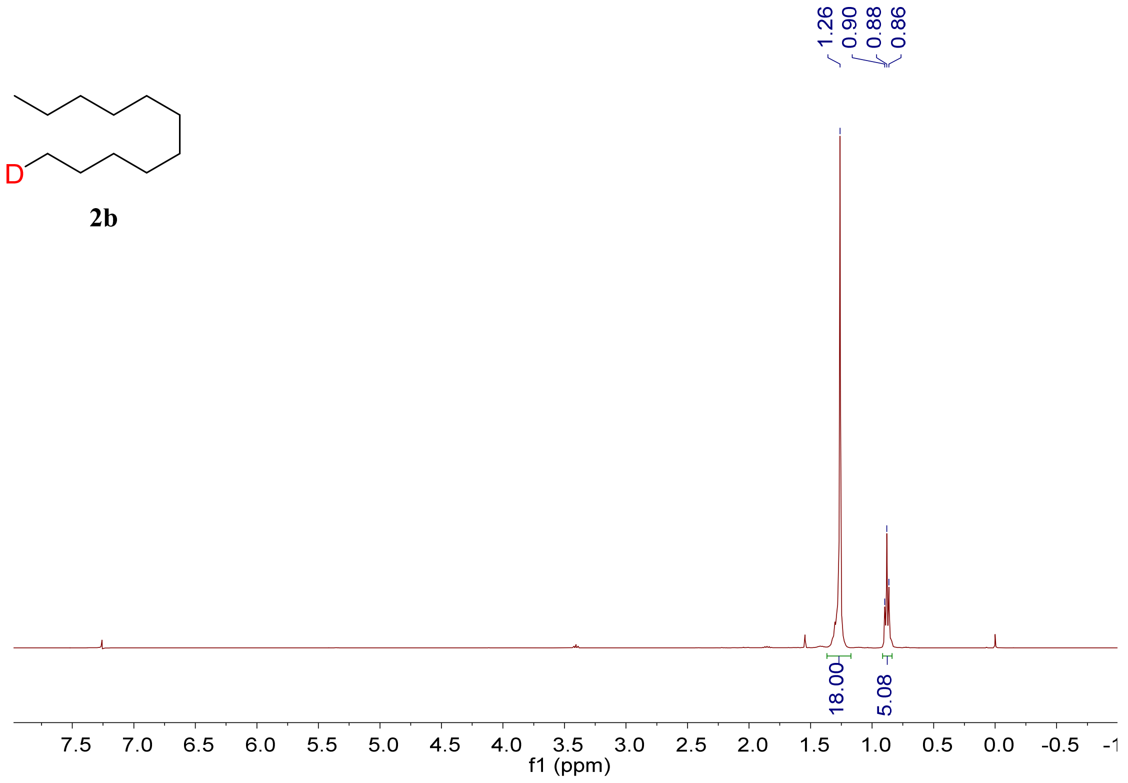
2b
1.26
0.90
0.88
0.86
18.00
5.08
7.5 7.0 6.5 6.0 5.5 5.0 4.5 4.0 3.5 3.0 2.5 2.0 1.5 1.0 0.5 0.0 -0.5
f1 (ppm)

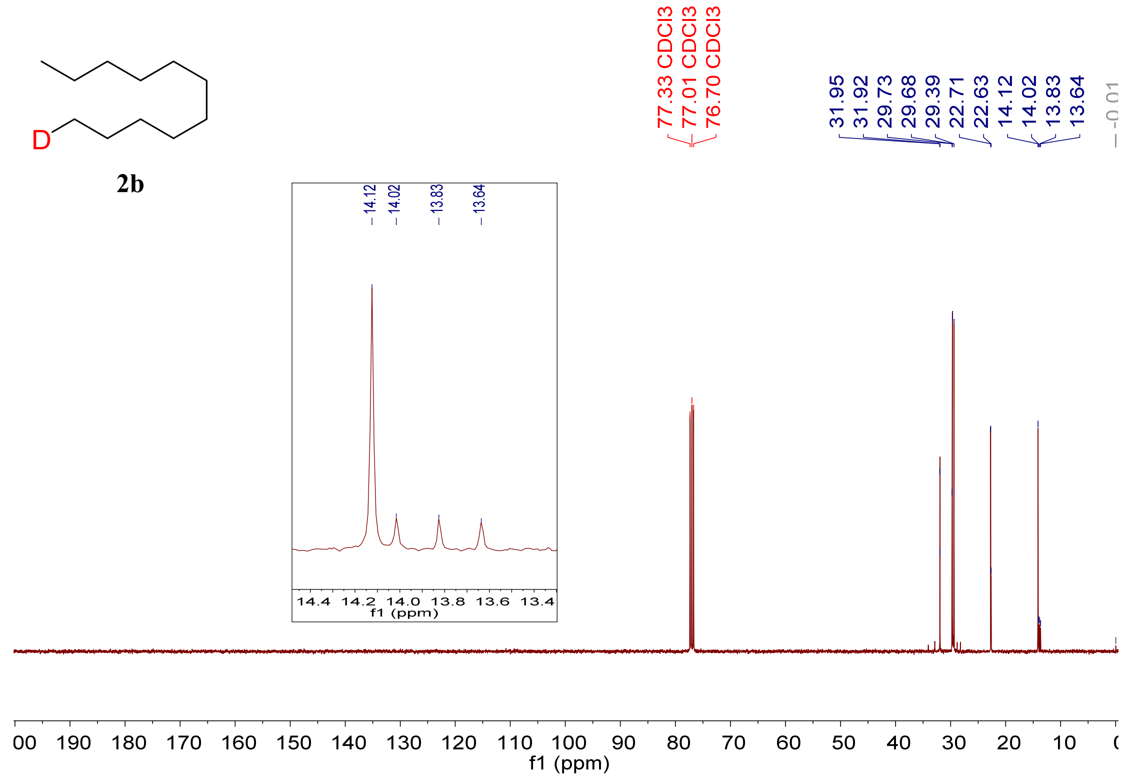
2b
77.33 CDCl3
77.01 CDCl3
76.70 CDCl3
31.95
31.92
29.73
29.68
29.39
22.71
22.63
14.12
14.02
13.83
13.64
14.12
14.02
13.83
13.64
14.4 14.2 14.0 13.8 13.6 13.4
f1 (ppm)
190 180 170 160 150 140 130 120 110 100 90 80 70 60 50 40 30 20 10
f1 (ppm)

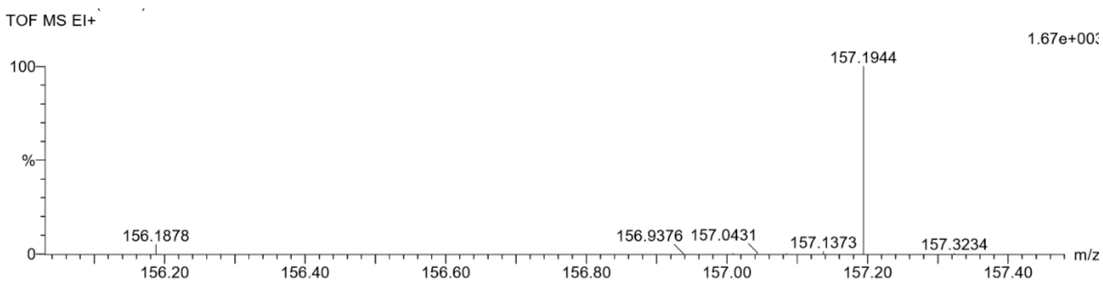

Minimum:	2.00				-1.5		
Maximum:	100.00		1.0	10.0	50.0		
Mass	RA	Calc. Mass	mDa	PPM	DBE	i-FIT	Formula
156.1878	4.97	156.1878	0.0	0.0	0.0	2773839.5	C11 1H24
157.1944	100.00	157.1941	0.3	1.9	0.0	5546720.5	C11 1H23 2H

Supplementary Fig. 49. NMR and HRMS spectra of Undecane-1-d (2b). The splitting of C-D coupling in ^{13}C-NMR was shown with an enlarged view.

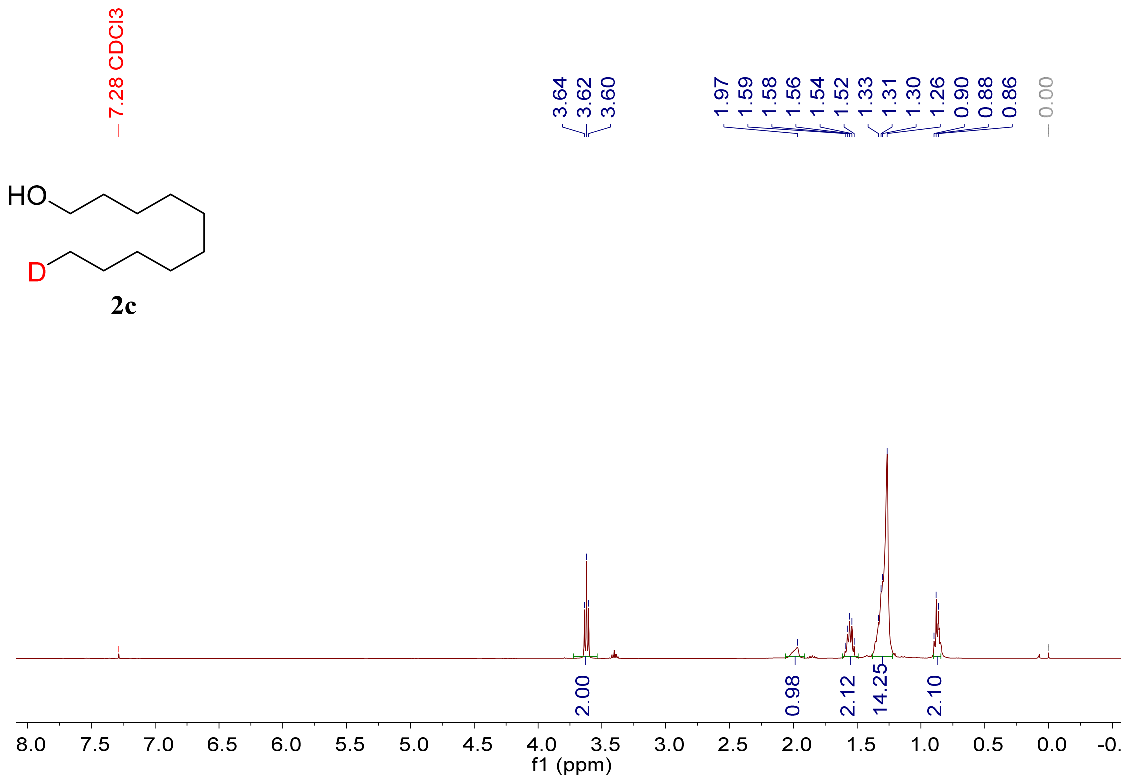

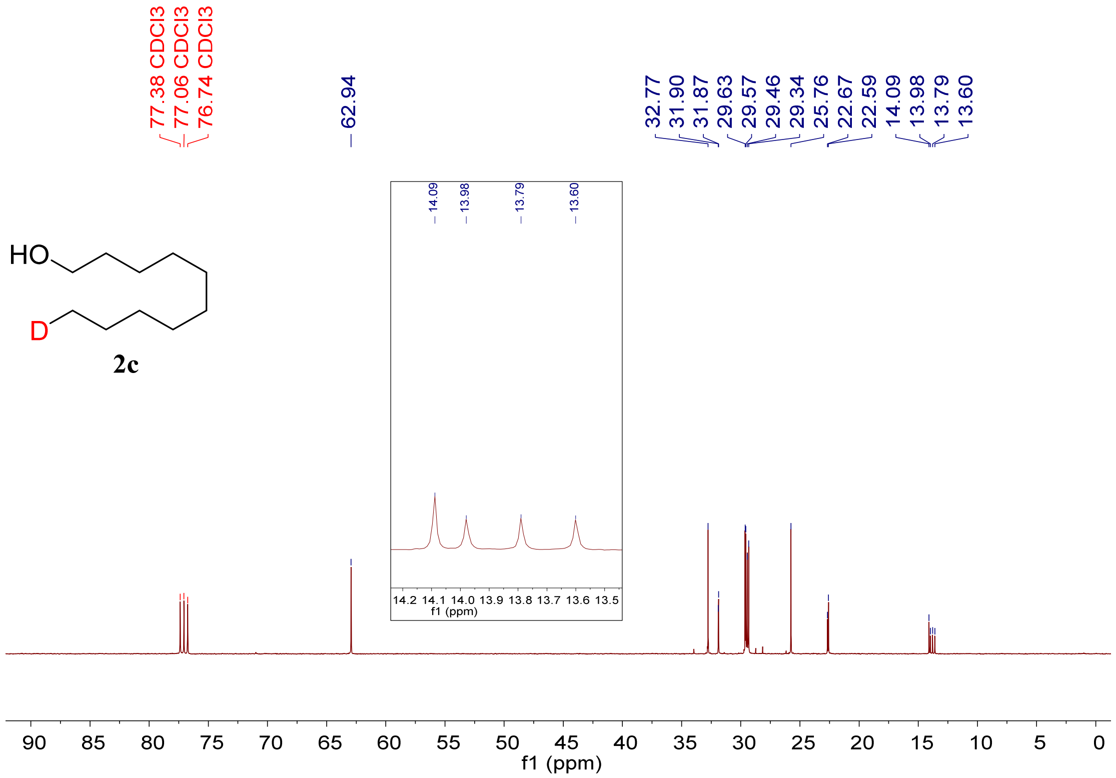

Supplementary Fig. 50. NMR spectra of Decan-10-d-1-ol (2c). The splitting of C-D coupling in ^{13}C-NMR was shown with an enlarged view.

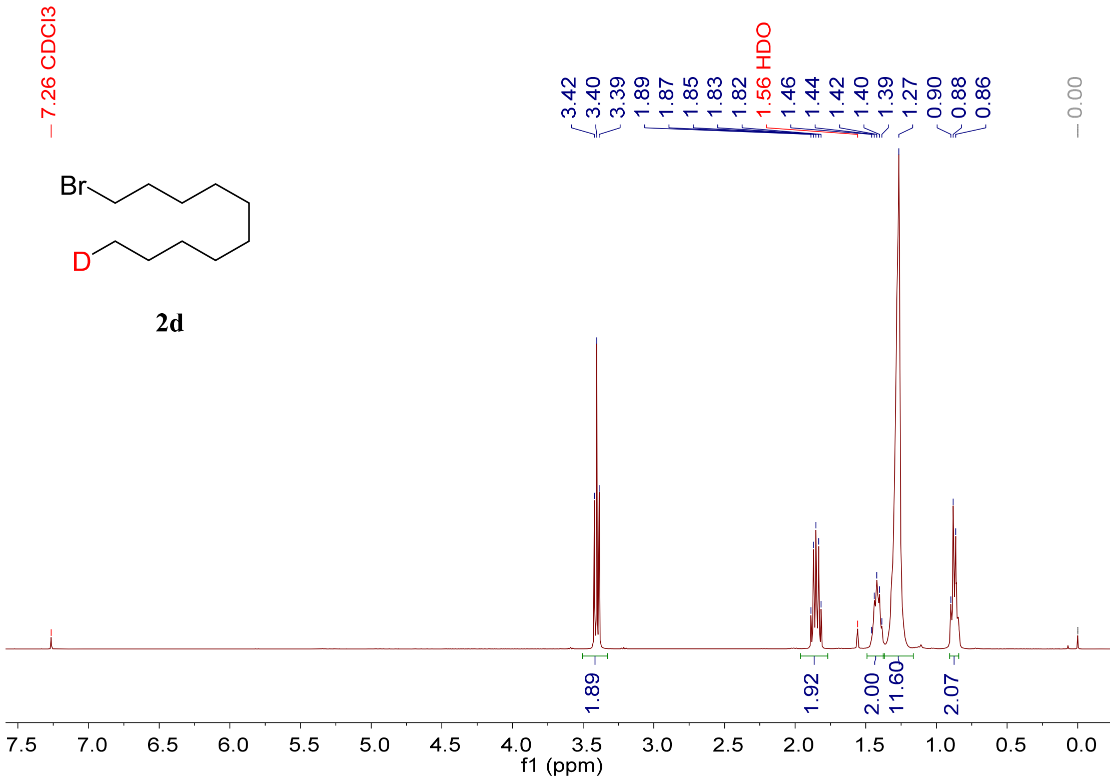
7.26 CDCl3
3.42
3.40
3.39
1.89
1.87
1.85
1.83
1.82
1.56 HDO
1.46
1.44
1.42
1.40
1.39
1.27
0.90
0.88
0.86
0.00
Br
D
2d
1.89
1.92
2.00
11.60
2.07
7.5 7.0 6.5 6.0 5.5 5.0 4.5 4.0 3.5 3.0 2.5 2.0 1.5 1.0 0.5 0.0
f1 (ppm)

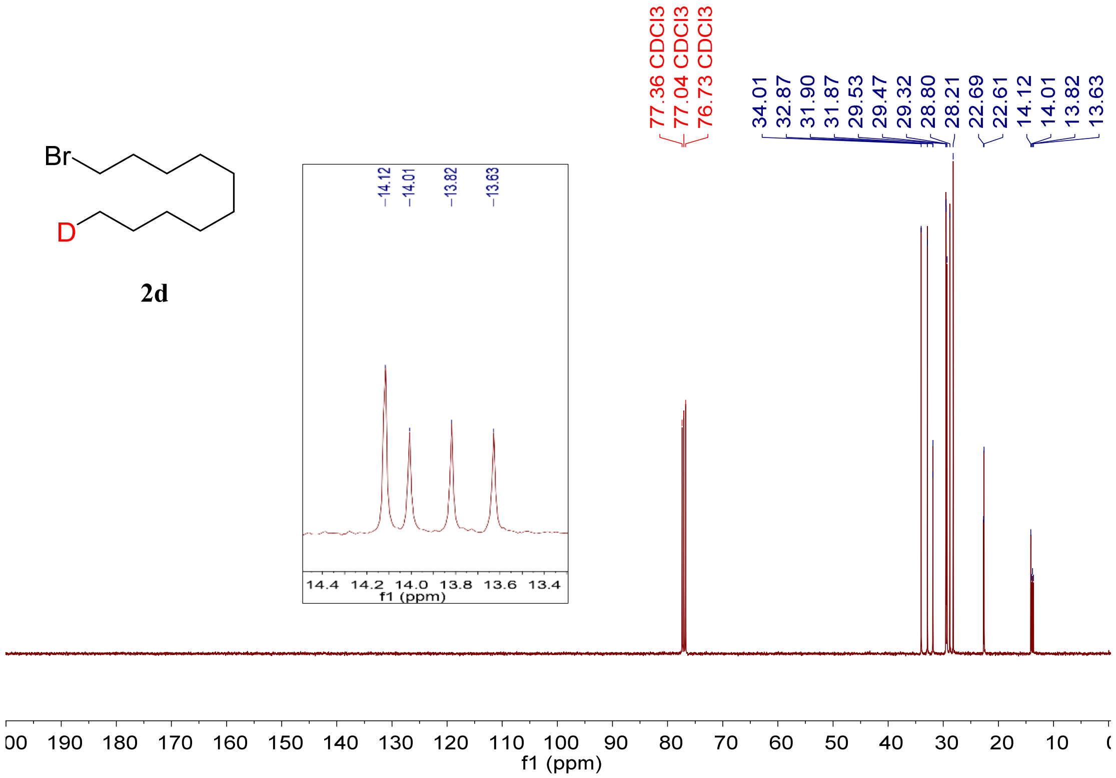
77.36 CDCl3
77.04 CDCl3
76.73 CDCl3
34.01
32.87
31.90
31.87
29.53
29.47
29.32
28.80
28.21
22.69
22.61
14.12
14.01
13.82
13.63
Br
D
2d
14.12
14.01
13.82
13.63
14.4 14.2 14.0 13.8 13.6 13.4
f1 (ppm)
190 180 170 160 150 140 130 120 110 100 90 80 70 60 50 40 30 20 10
f1 (ppm)

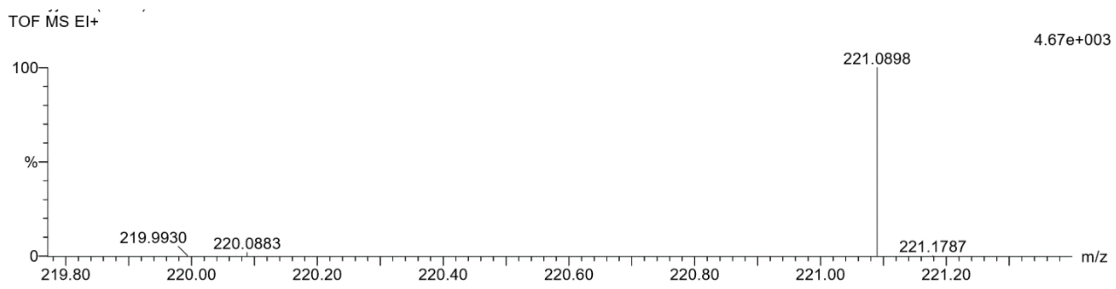

	Mass	RA	Calc. Mass	mDa	PPM	DBE	i-FIT	Formula
Minimum:		5.00				-1.5		
Maximum:		100.00		1.0	10.0	50.0		
	221.0898	100.00	---					

Supplementary Fig. 51. NMR and HRMS spectra of 1-Bromodecane-10-d (2d). The splitting of C-D coupling in ^{13}C-NMR was shown with an enlarged view.

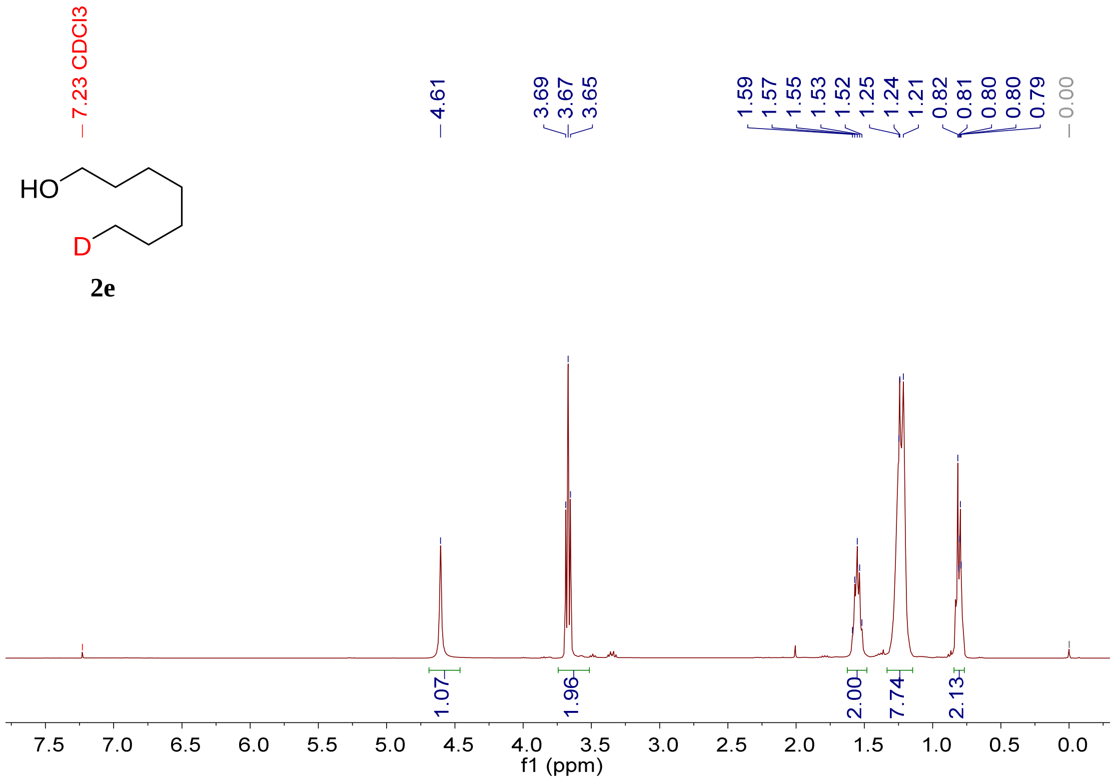

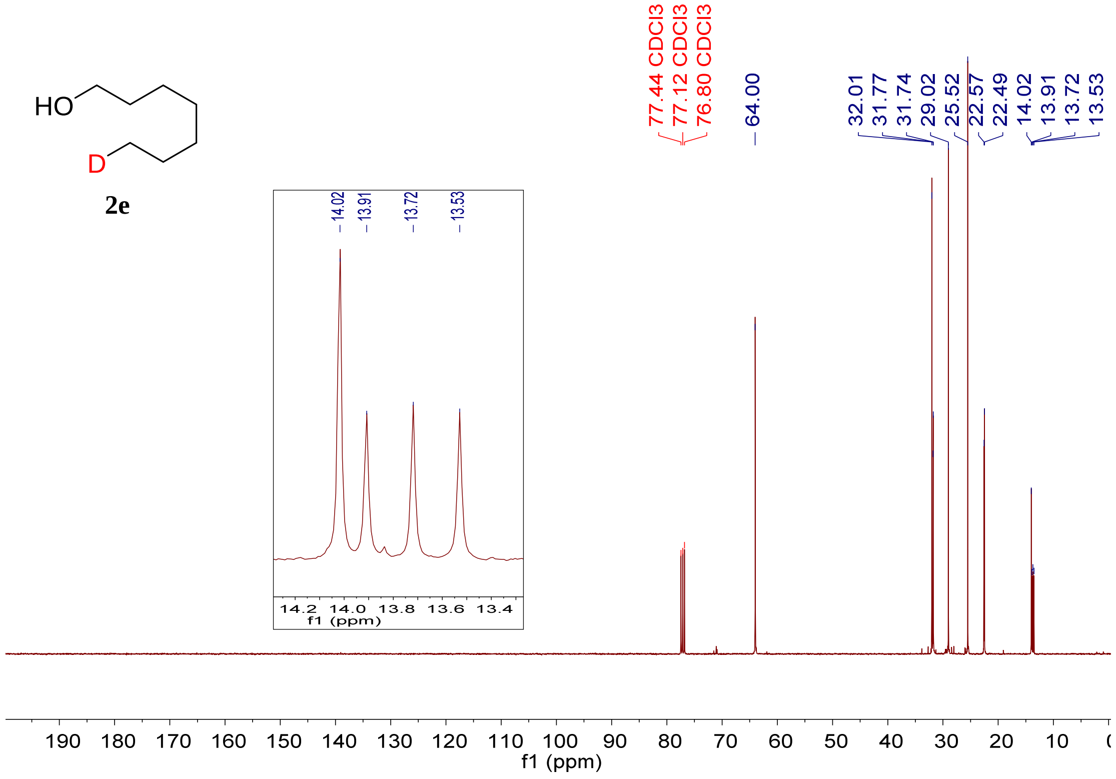

Supplementary Fig. 52. NMR spectra of Heptan-7-d-1-ol (2e). The splitting of C-D coupling in ^{13}C-NMR was shown with an enlarged view.

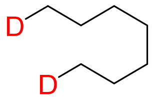

2f

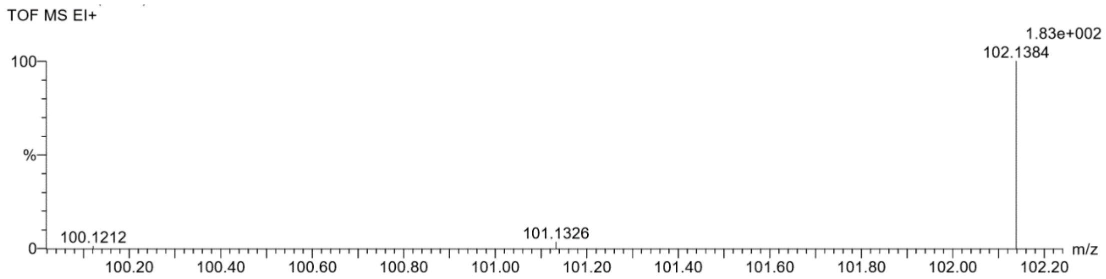

Minimum:	2.00				-1.5		
Maximum:	100.00		1.0	10.0	50.0		
Mass	RA	Calc. Mass	mDa	PPM	DBE	i-FIT	Formula
101.1326	3.32	101.1330	-0.4	-4.0	-0.5	2773104.3	C7 1H17
102.1384	100.00	102.1393	-0.9	-8.8	-0.5	2773024.5	C7 1H16 2H

Supplementary Fig. 53. HRMS spectrum of Heptane-1,7-d2 (2f).

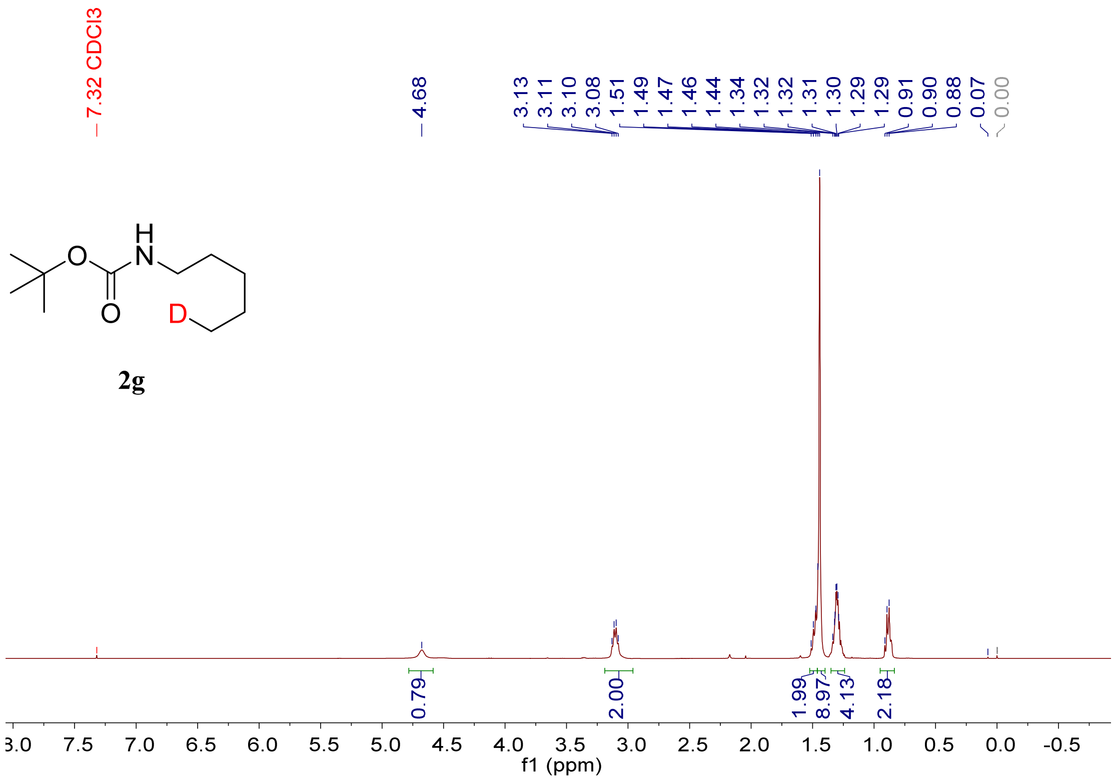

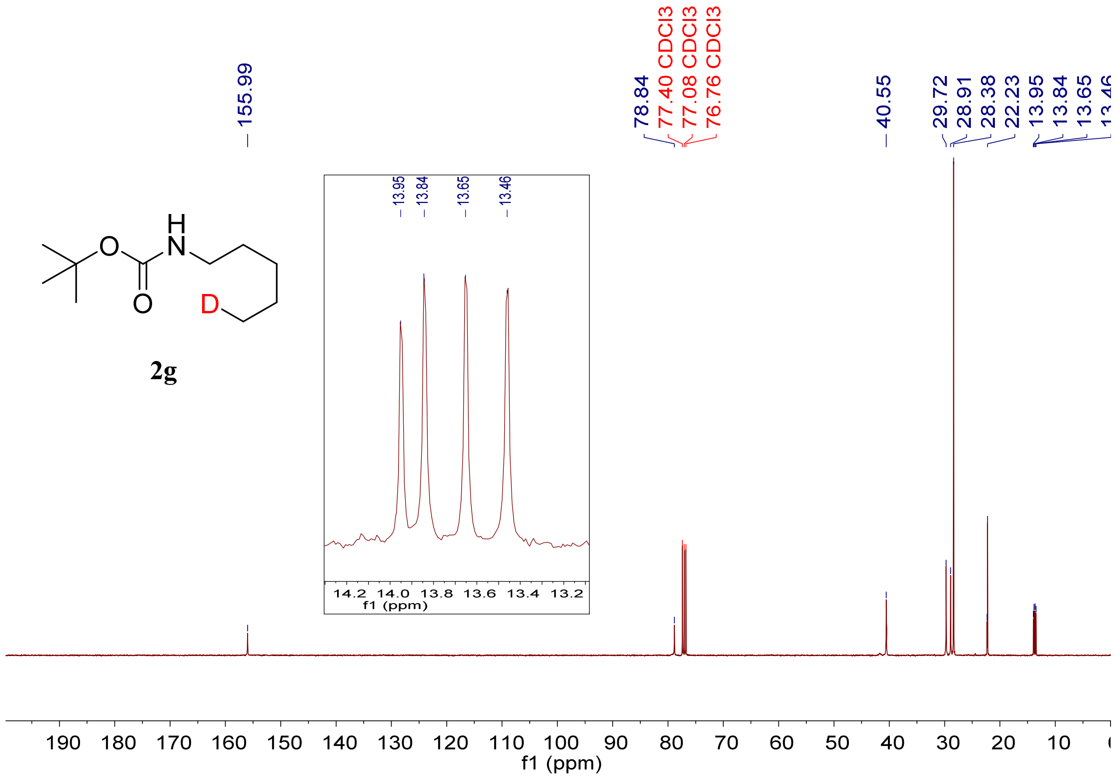

Supplementary Fig. 54. NMR spectra of *tert*-Butyl (pentyl-5-d)carbamate (2g). The splitting of C-D coupling in ^{13}C-NMR was shown with an enlarged view.

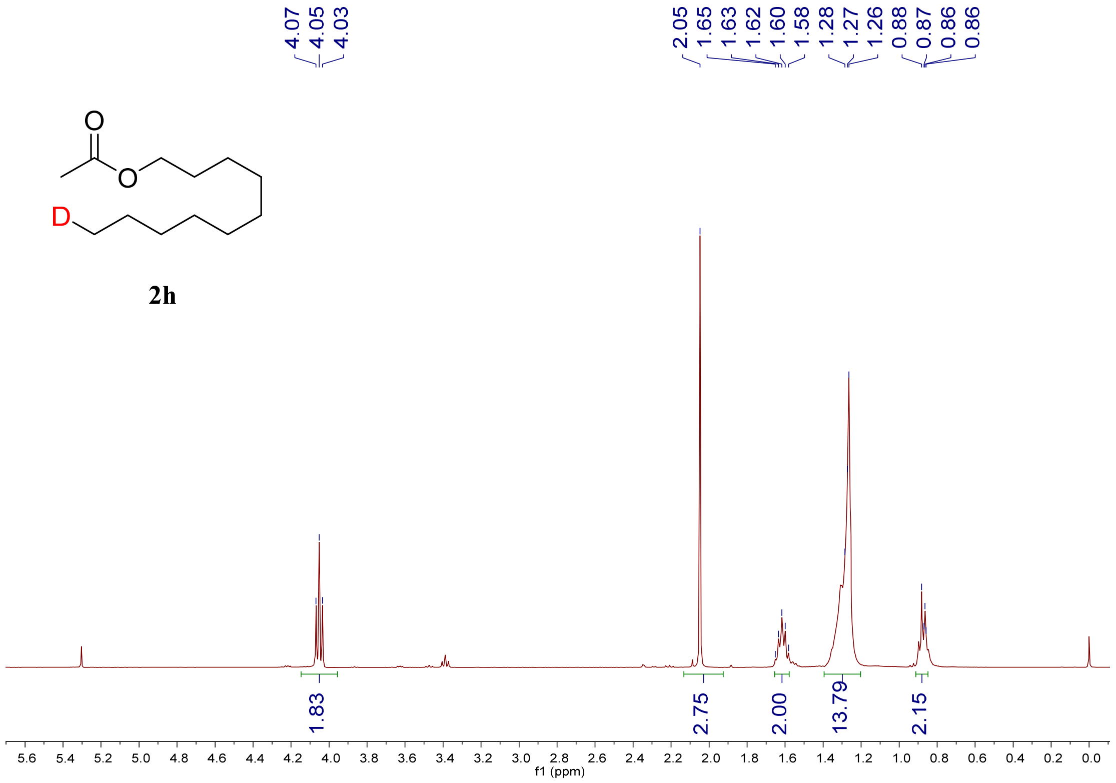

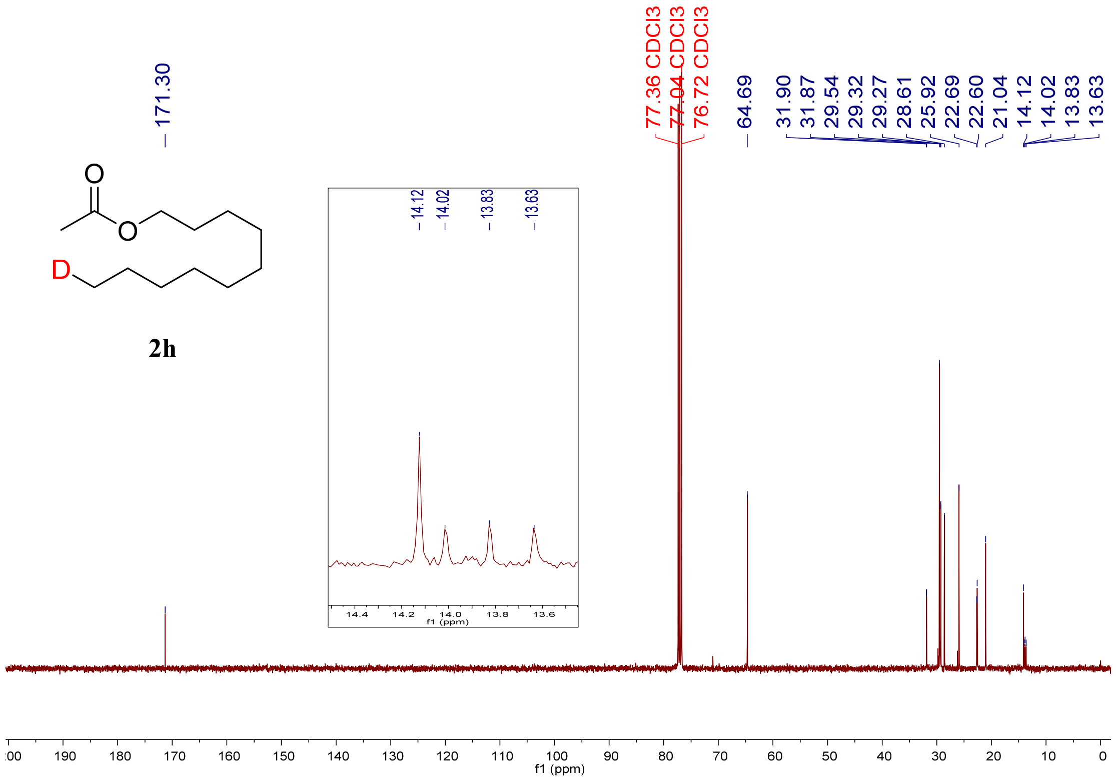

Supplementary Fig. 55. NMR spectra of Decyl-10-d acetate (2h). The splitting of C-D coupling in ^{13}C-NMR was shown with an enlarged view.

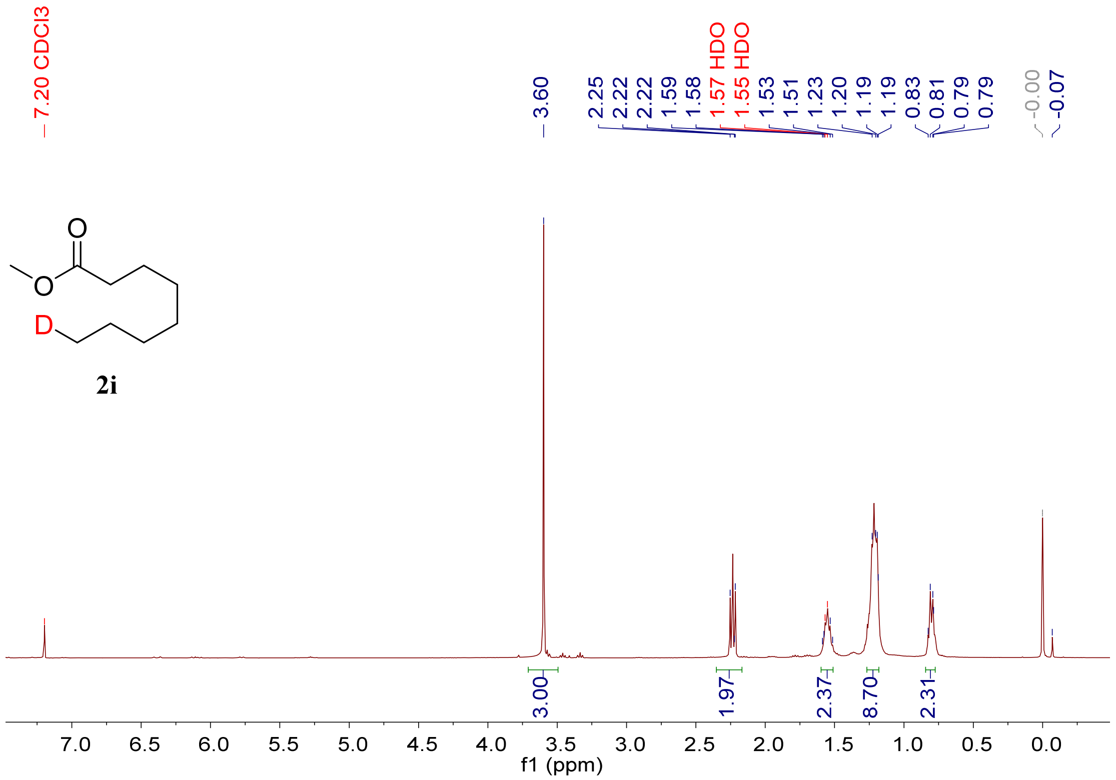

7.20 CDCl3
3.60
2.25
2.22
2.22
1.59
1.58
1.57 HDO
1.55 HDO
1.53
1.51
1.23
1.20
1.19
1.19
0.83
0.81
0.79
0.79
-0.00
-0.07
D
O
O
2i
3.00
1.97
2.37
8.70
2.31
f1 (ppm)

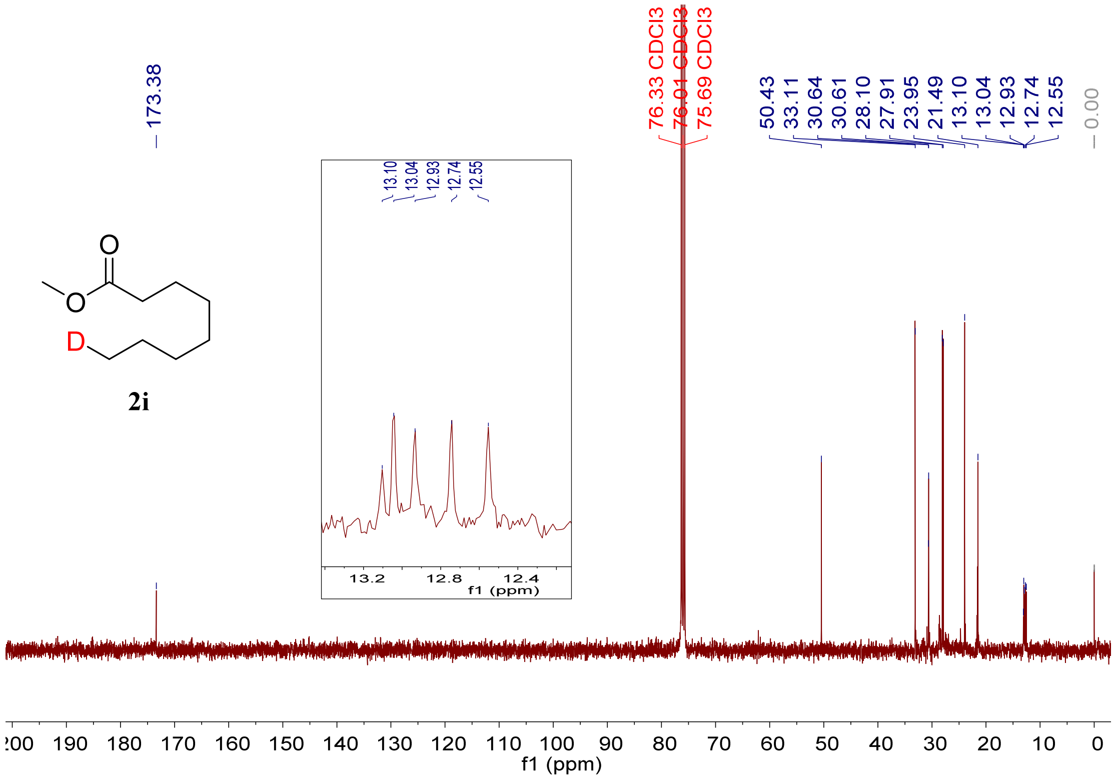

173.38
76.33 CDCl3
76.01 CDCl3
75.69 CDCl3
50.43
33.11
30.64
30.61
28.10
27.91
23.95
21.49
13.10
13.04
12.93
12.74
12.55
0.00
D
O
O
2i
f1 (ppm)

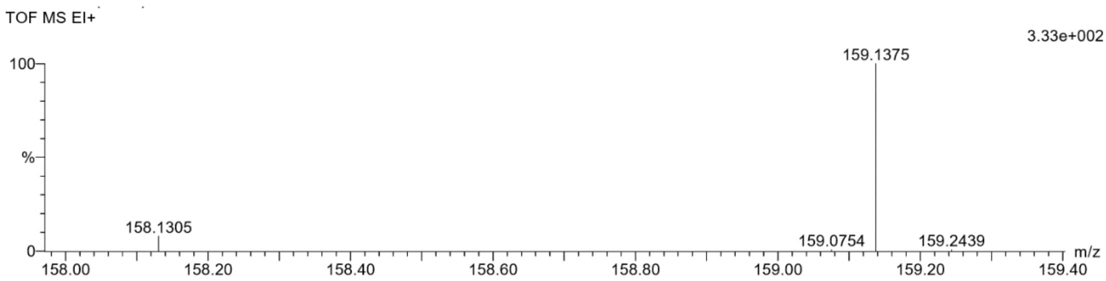

Minimum:	1.00				-1.5		
Maximum:	100.00		1.0	10.0	50.0		
Mass	RA	Calc. Mass	mDa	PPM	DBE	i-FIT	Formula
158.1305	7.90	158.1307	-0.2	-1.3	1.0	2773177.0	C9 1H18 O2
159.1375	100.00	159.1370	0.5	3.1	1.0	5546135.0	C9 1H17 2H O2

Supplementary Fig. 56. NMR and HRMS spectra of Methyl octanoate-8-d (2i). The splitting of C-D coupling in ^{13}C-NMR was shown with an enlarged view.

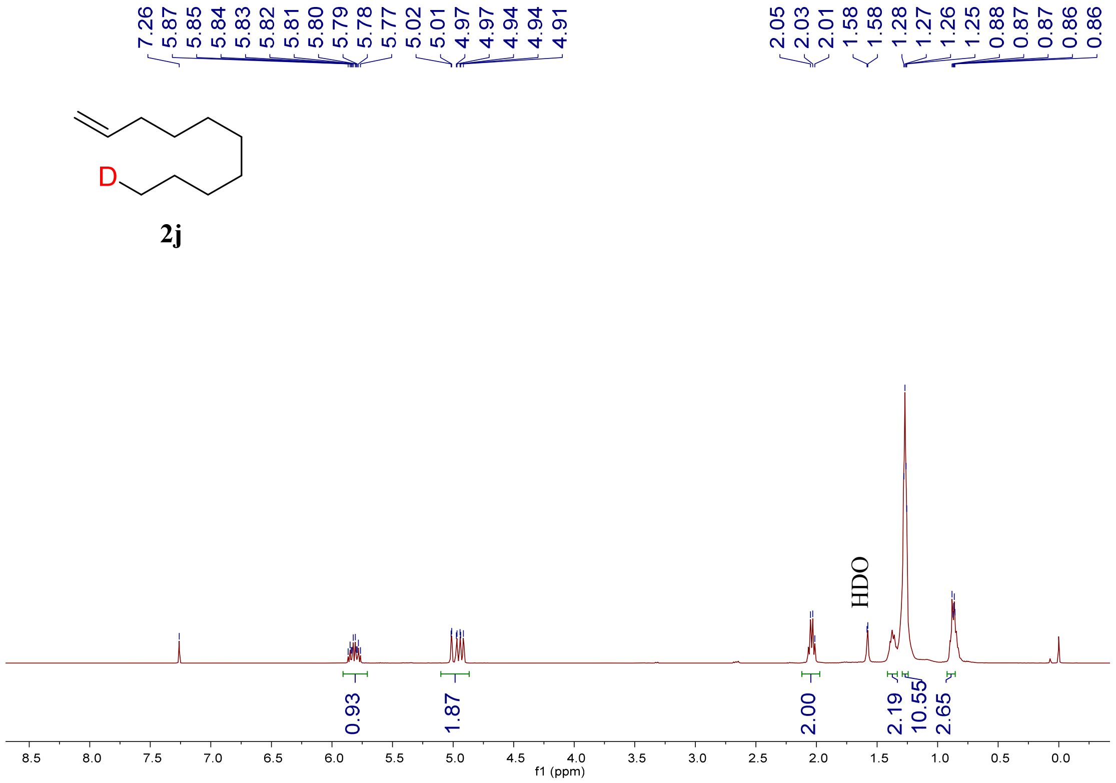

7.26
5.87
5.85
5.84
5.83
5.82
5.81
5.80
5.79
5.78
5.77
5.02
5.01
4.97
4.97
4.94
4.94
4.91
2.05
2.03
2.01
1.58
1.58
1.28
1.27
1.26
1.25
0.88
0.87
0.87
0.86
0.86
D
2j
HDO
0.93
1.87
2.00
2.19
10.55
2.65
f1 (ppm)

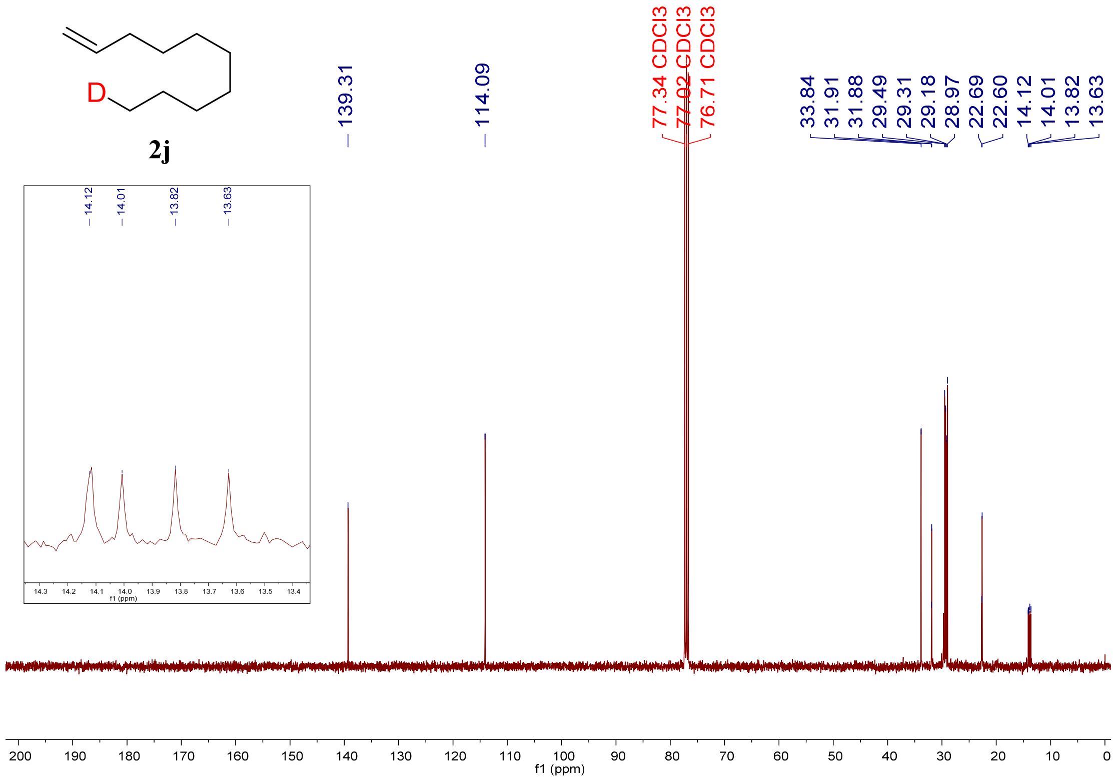

D
2j
139.31
114.09
77.34 CDCl3
77.02 CDCl3
76.71 CDCl3
33.84
31.91
31.88
29.49
29.31
29.18
28.97
22.69
22.60
14.12
14.01
13.82
13.63
f1 (ppm)

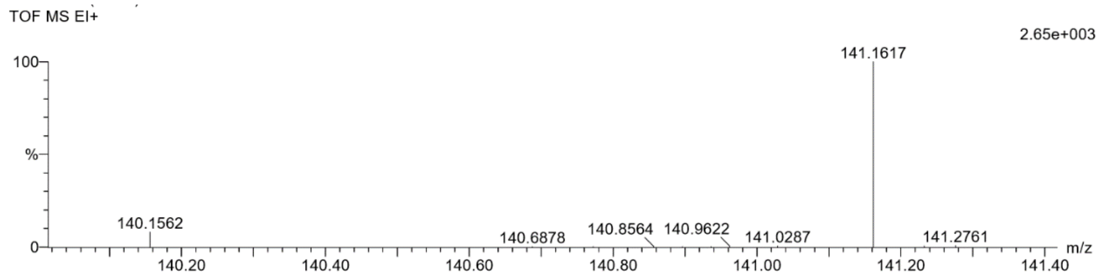

Minimum:	5.00				-1.5		
Maximum:	100.00		2.0	10.0	50.0		
Mass	RA	Calc. Mass	mDa	PPM	DBE	i-FIT	Formula
140.1562	8.05	140.1565	-0.3	-2.1	1.0	2774313.3	C10 1H20
		140.1550	1.2	8.6	1.5	2774341.3	C10 1H18 2H
141.1617	100.00	141.1612	0.5	3.5	1.5	2773952.0	C10 1H17 2H2
		141.1628	-1.1	-7.8	1.0	5547170.0	C10 1H19 2H

Supplementary Fig. 57. NMR and HRMS spectra of Dec-1-ene-10-d (2j). The splitting of C-D coupling in ^{13}C-NMR was shown with an enlarged view.

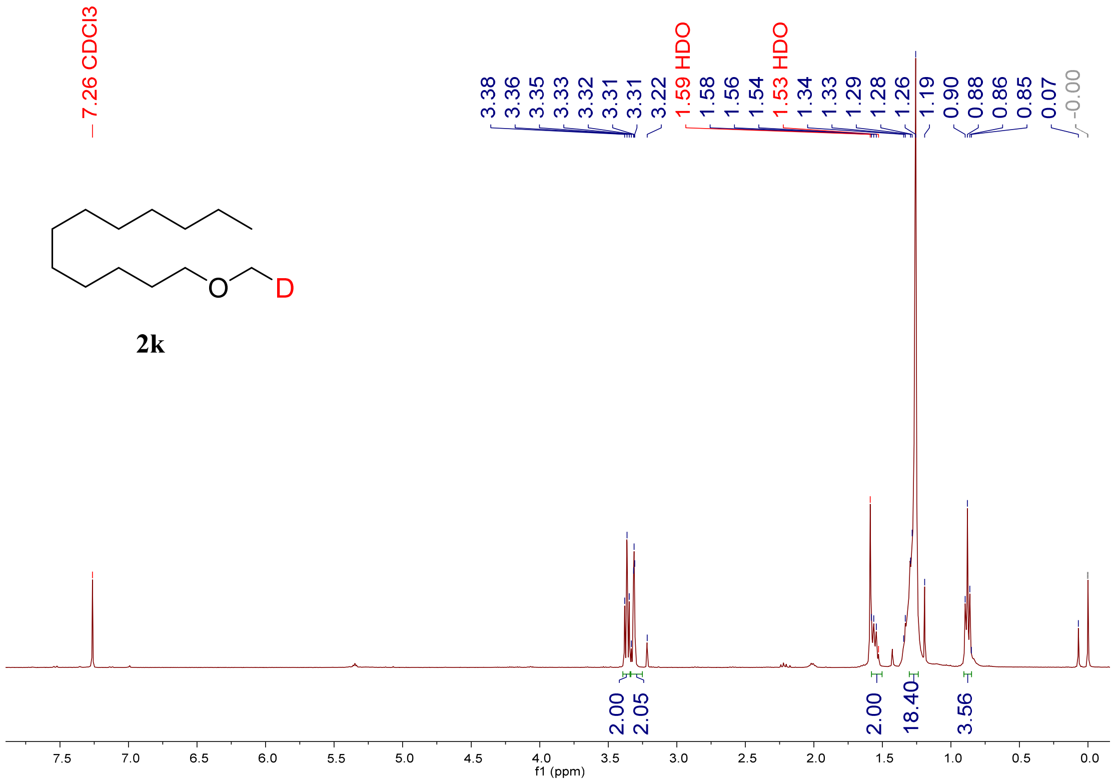

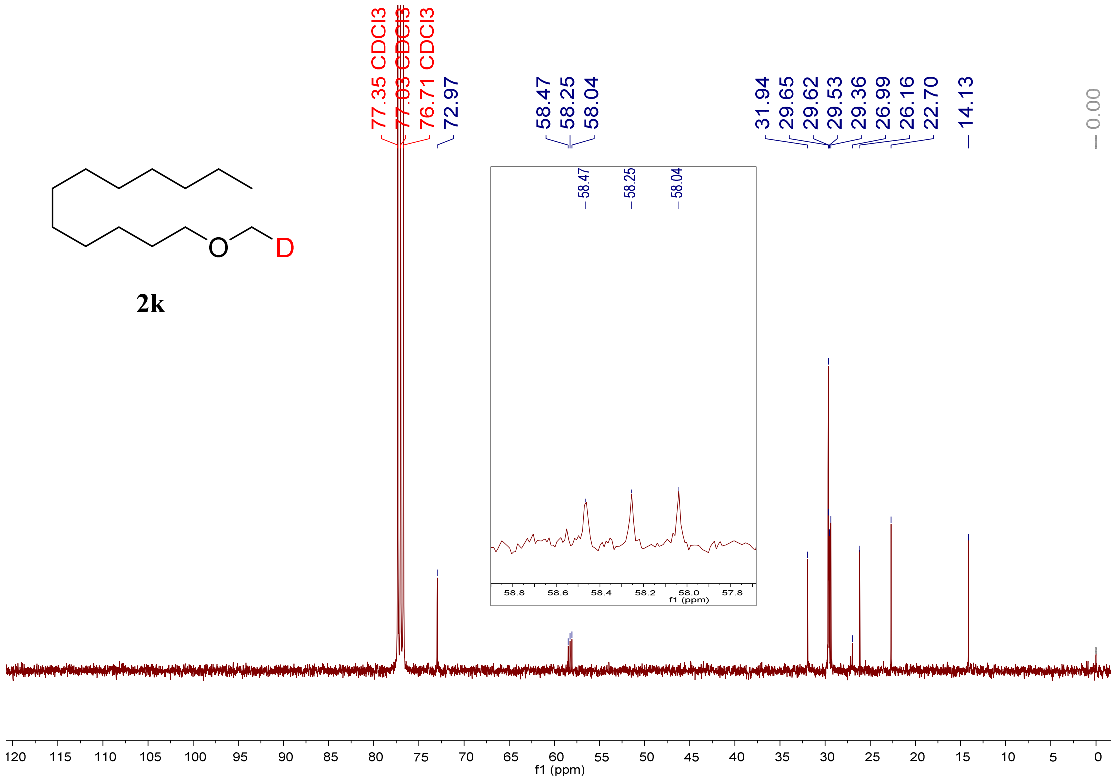

Supplementary Fig. 58. NMR spectra of 1-(Methoxy-d)dodecane (2k). The splitting of C-D coupling in ^{13}C-NMR was shown with an enlarged view.

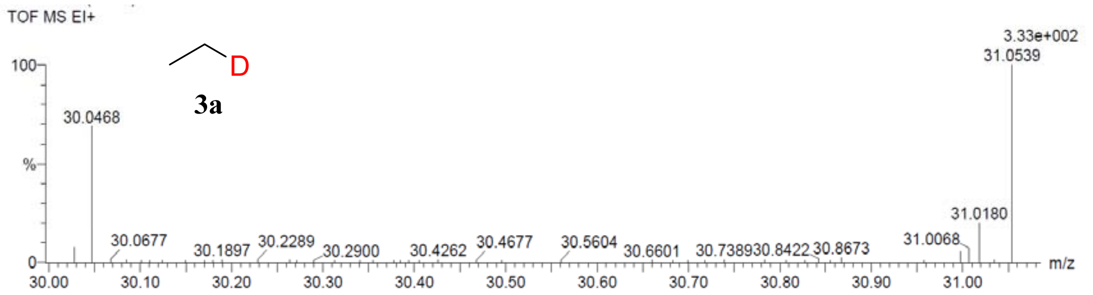

Minimum:	40.00				-1.5		
Maximum:	100.00		0.8	10.0	50.0		

Mass	RA	Calc. Mass	mDa	PPM	DBE	i-FIT	Formula
30.0468	68.86	30.0470	-0.2	-6.7	0.0	2773186.0	C2 1H6
31.0539	100.00	31.0532	0.7	22.5	0.0	2773024.8	C2 1H5 2H

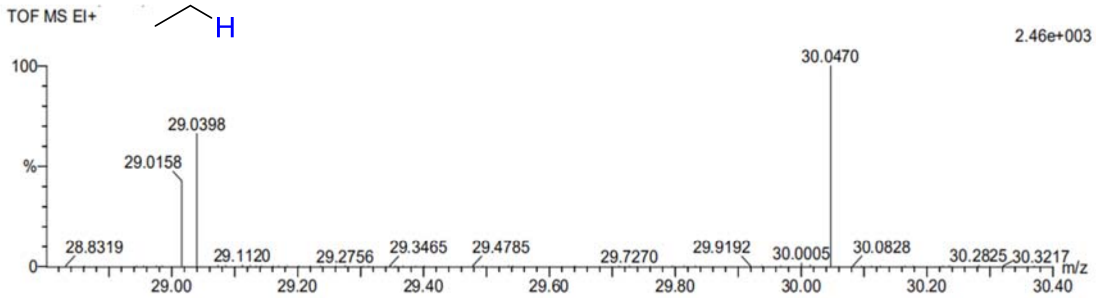

Minimum:	10.00				-1.5		
Maximum:	100.00		10.0	10.0	50.0		

Mass	RA	Calc. Mass	mDa	PPM	DBE	i-FIT	Formula
29.0158	42.54	---					
29.0398	66.37	29.0391	0.7	24.1	0.5	2774353.5	C2 H5
30.0470	100.00	30.0470	0.0	0.0	0.0	5546700.0	C2 H6

Supplementary Fig. 59. HRMS spectra of Ethane-d (3a) and Ethane without deuterium.

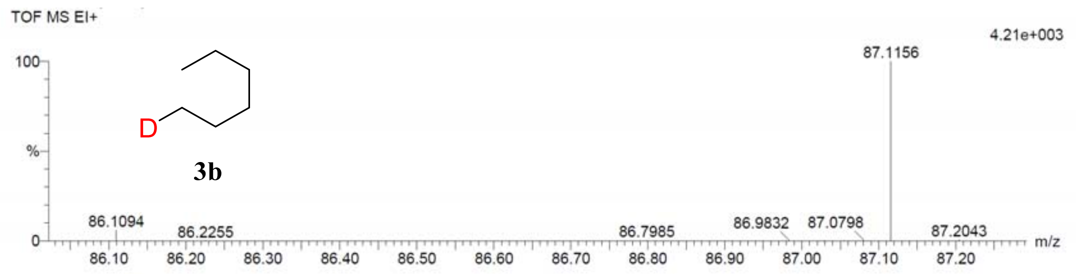

Minimum:	5.00				-1.5		
Maximum:	100.00		1.0	10.0	50.0		
Mass	RA	Calc. Mass	mDa	PPM	DBE	i-FIT	Formula
86.1094	5.82	86.1096	-0.2	-2.3	0.0	2775110.8	C6 1H14
87.1156	100.00	87.1158	-0.2	-2.3	0.0	2774638.3	C6 1H13 2H

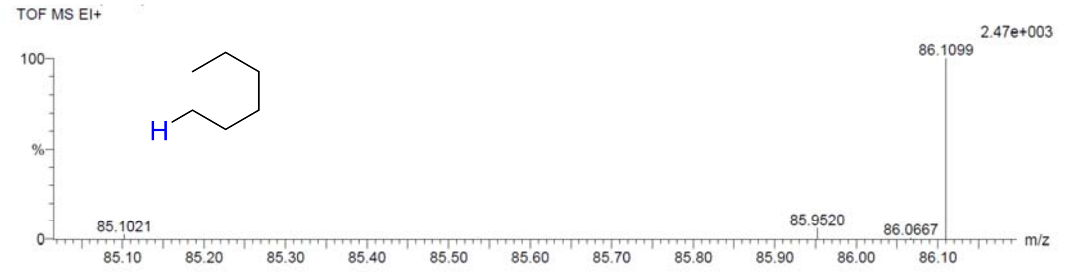

Minimum:	1.00				-1.5		
Maximum:	100.00		1.0	10.0	50.0		
Mass	RA	Calc. Mass	mDa	PPM	DBE	i-FIT	Formula
85.1021	2.33	85.1017	0.4	4.7	0.5	2774266.3	C6 H13
85.9520	5.85	---					
86.1099	100.00	86.1096	0.3	3.5	0.0	5547151.5	C6 H14

Supplementary Fig. 60. HRMS spectra of Hexane-1-d (3b) and Hexane without deuterium.

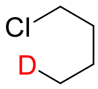

3c

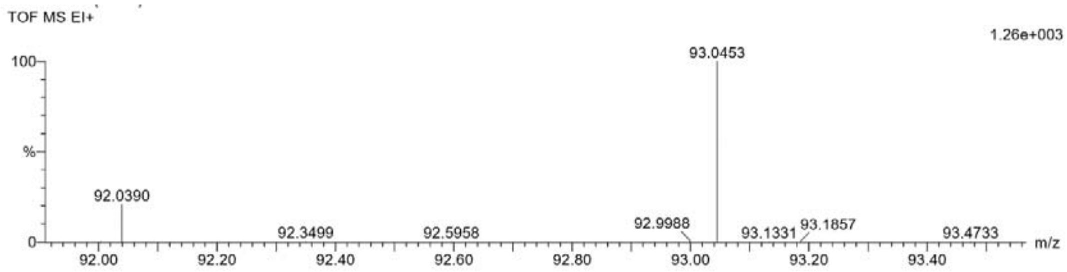

Minimum:	2.00				-1.5		
Maximum:	100.00		1.0	10.0	50.0		
Mass	RA	Calc. Mass	mDa	PPM	DBE	i-FIT	Formula
92.0390	20.77	92.0393	-0.3	-3.3	0.0	2773768.5	C4 1H9 Cl
93.0453	100.00	93.0456	-0.3	-3.2	0.0	5546658.5	C4 1H8 2H Cl

Supplementary Fig. 61. HRMS spectrum of 1-Chlorobutane-4-d (3c).

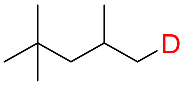

3d

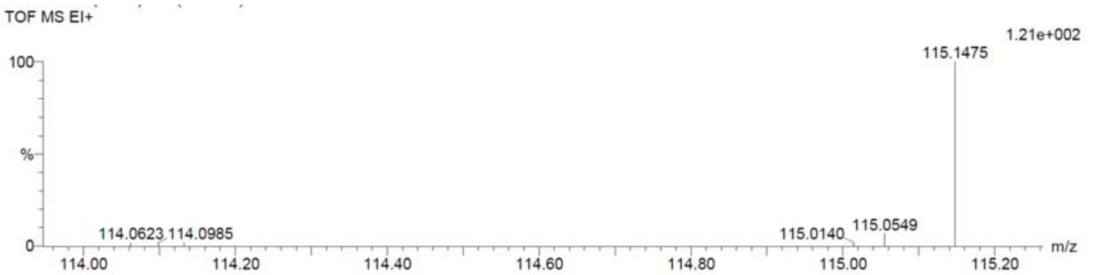

Minimum:	2.00			-1.5
Maximum:	100.00	1.0	10.0	50.0

Mass	RA	Calc. Mass	mDa	PPM	DBE	i-FIT	Formula
115.0549	6.67	115.0548	0.1	0.9	6.5	5546025.5	C9 1H7
115.1475	100.00	115.1471	0.4	3.5	0.0	2773019.0	C8 1H17 2H

Supplementary Fig. 62. HRMS spectrum of 2, 2, 4-Trimethylpentane-5-d (3d).

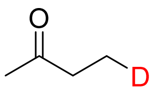

3e

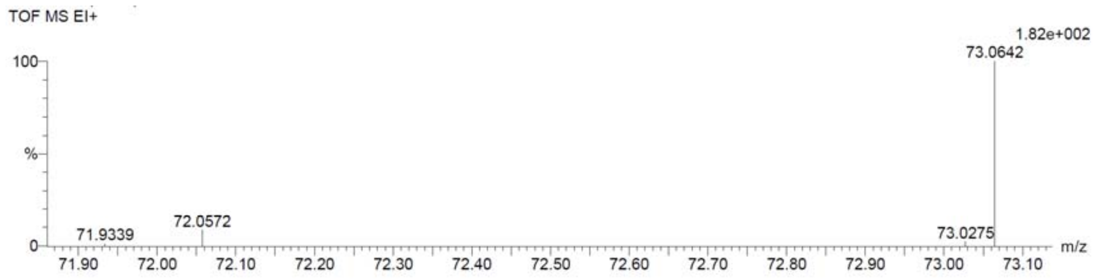

Minimum:	5.00				-1.5		
Maximum:	100.00		1.0	10.0	50.0		

Mass	RA	Calc. Mass	mDa	PPM	DBE	i-FIT	Formula
72.0572	8.36	72.0575	-0.3	-4.2	1.0	2773104.0	C4 1H8 O
73.0642	100.00	73.0638	0.4	5.5	1.0	2773044.0	C4 1H7 2H O

Supplementary Fig. 63. HRMS spectrum of Butan-2-one-4-d (3e).

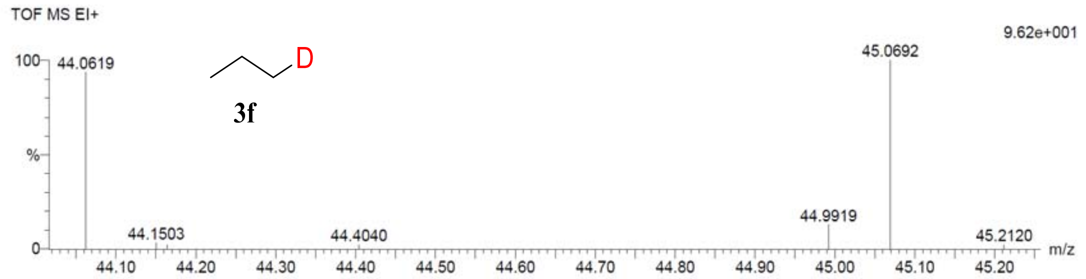

Minimum:	20.00				-1.5		
Maximum:	100.00		0.8	10.0	50.0		

Mass	RA	Calc. Mass	mDa	PPM	DBE	i-FIT	Formula
44.0619	93.69	44.0626	-0.7	-15.9	0.0	2773059.3	C3 1H8
45.0692	100.00	45.0689	0.3	6.7	0.0	2773013.8	C3 1H7 2H

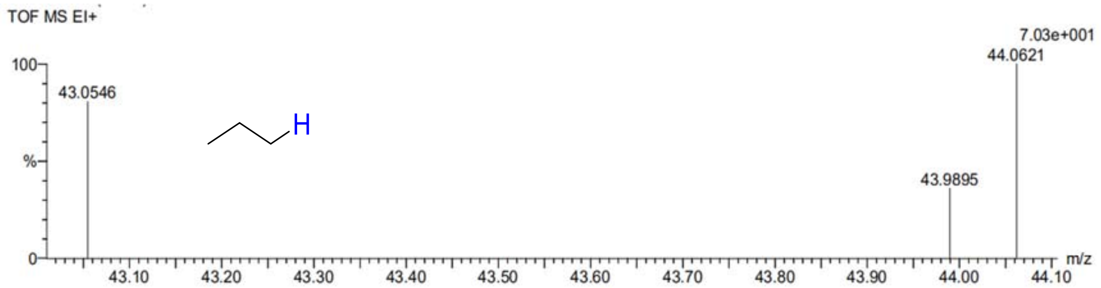

Minimum:	5.00				-1.5		
Maximum:	100.00		2.0	10.0	50.0		

Mass	RA	Calc. Mass	mDa	PPM	DBE	i-FIT	Formula
43.0546	80.68	43.0532	1.4	32.5	1.0	42.7	C3 1H5 2H
		43.0548	-0.2	-4.6	0.5	2773047.8	C3 1H7
43.9895	36.02	43.9898	-0.3	-6.8	2.0	5546025.5	C O2
44.0621	100.00	44.0611	1.0	22.7	0.5	2773014.0	C3 1H6 2H
		44.0626	-0.5	-11.3	0.0	5546026.5	C3 1H8

Supplementary Fig. 64. HRMS spectra of Propane-1-d (3f) and Propane without deuterium.

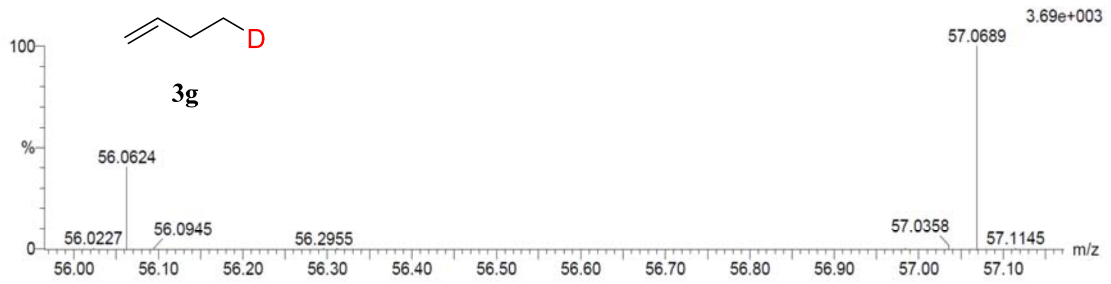

Minimum:	20.00				-1.5		
Maximum:	100.00		1.0	10.0	50.0		

Mass	RA	Calc. Mass	mDa	PPM	DBE	i-FIT	Formula
56.0624	40.37	56.0626	-0.2	-3.6	1.0	2774814.3	C4 1H8
57.0689	100.00	57.0689	0.0	0.0	1.0	2773983.3	C4 1H7 2H

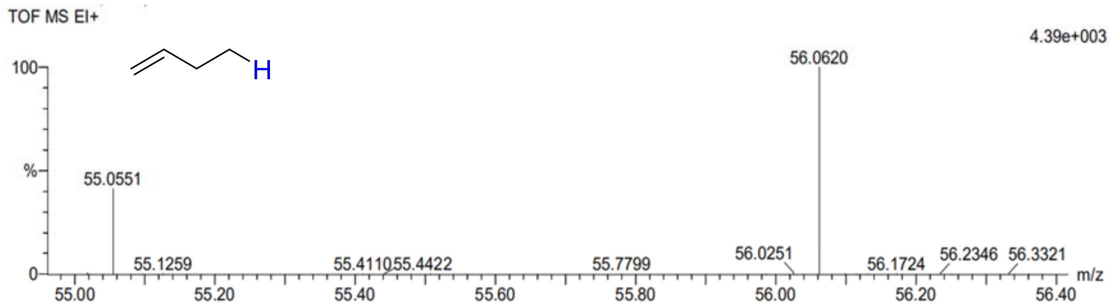

Minimum:	5.00				-1.5		
Maximum:	100.00		2.0	10.0	50.0		

Mass	RA	Calc. Mass	mDa	PPM	DBE	i-FIT	Formula
55.0551	41.23	55.0548	0.3	5.4	1.5	2775181.0	C4 H7
56.0620	100.00	56.0626	-0.6	-10.7	1.0	5547987.0	C4 H8

Supplementary Fig. 65. HRMS spectra of But-1-ene-4-d (3g) and Butene without deuterium.

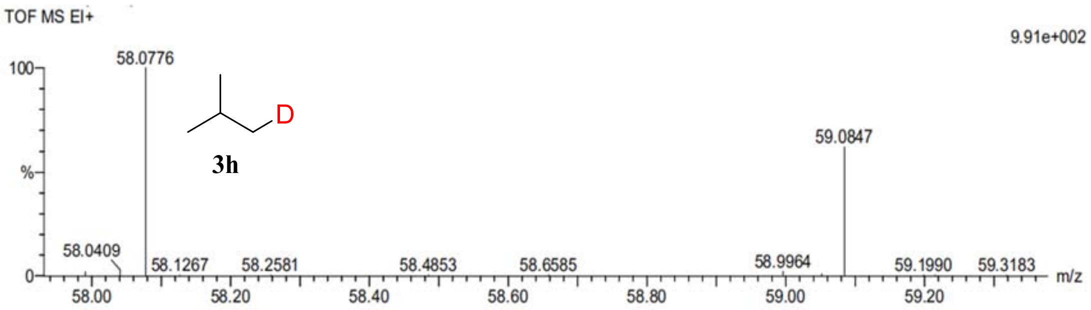

Minimum:	20.00				-1.5		
Maximum:	100.00		1.0	10.0	50.0		
Mass	RA	Calc. Mass	mDa	PPM	DBE	i-FIT	Formula
58.0776	100.00	58.0767	0.9	15.5	0.5	327.7	C4 1H8 2H
		58.0783	-0.7	-12.1	0.0	2773280.3	C4 1H10
59.0847	62.11	59.0845	0.2	3.4	0.0	2773057.8	C4 1H9 2H

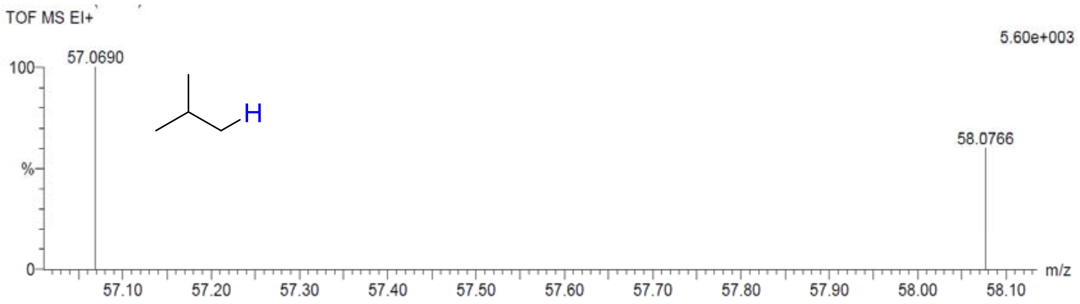

Minimum:	1.00				-1.5		
Maximum:	100.00		10.0	10.0	50.0		
Mass	RA	Calc. Mass	mDa	PPM	DBE	i-FIT	Formula
57.0690	100.00	57.0704	-1.4	-24.5	0.5	2774477.5	C4 H9
58.0766	60.05	58.0783	-1.7	-29.3	0.0	5547487.5	C4 H10

Supplementary Fig. 66. HRMS spectra of Isobutane-1-d (3h) and Isobutane without deuterium.

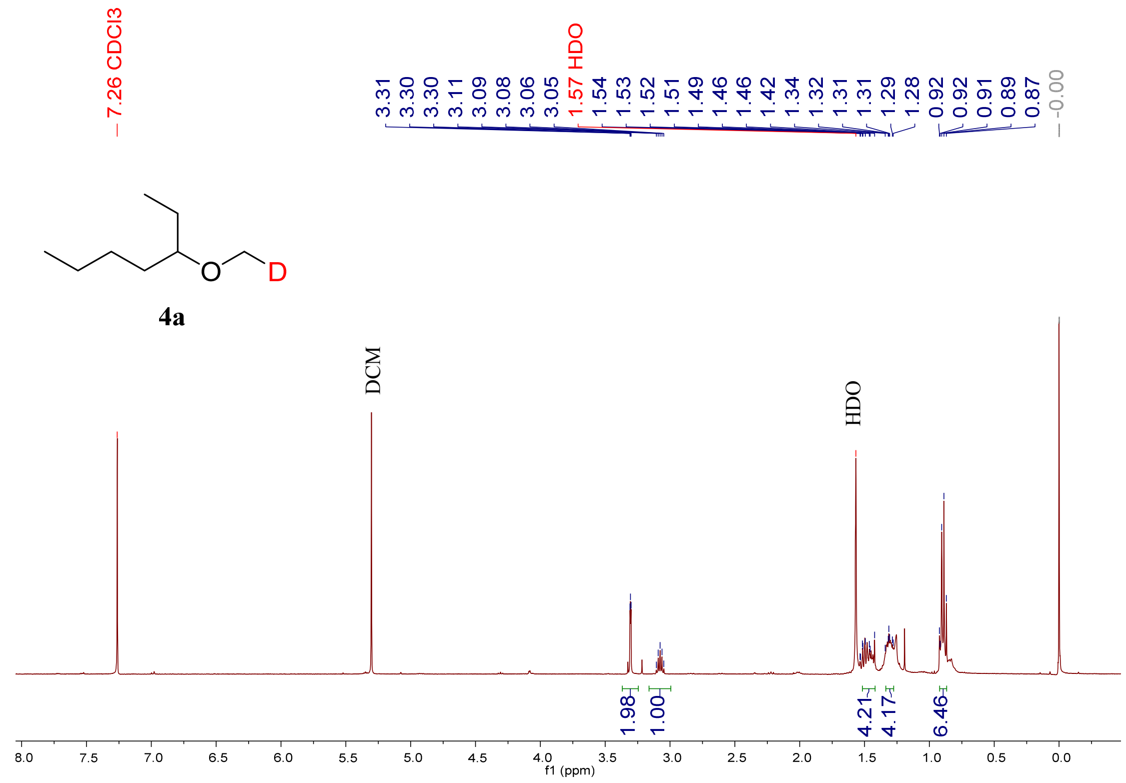

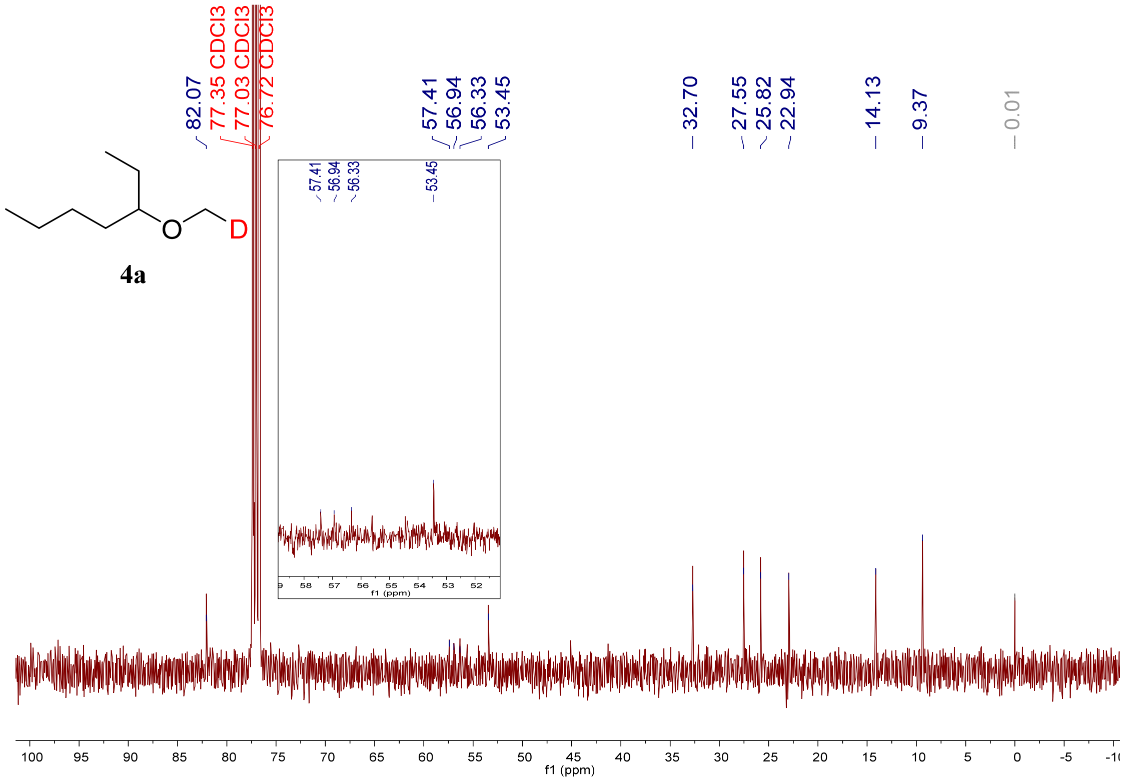

Supplementary Fig. 67. NMR spectra of 3-(Methoxy-d)heptane (4a). The splitting of C-D coupling in ^{13}C-NMR was shown with an enlarged view.

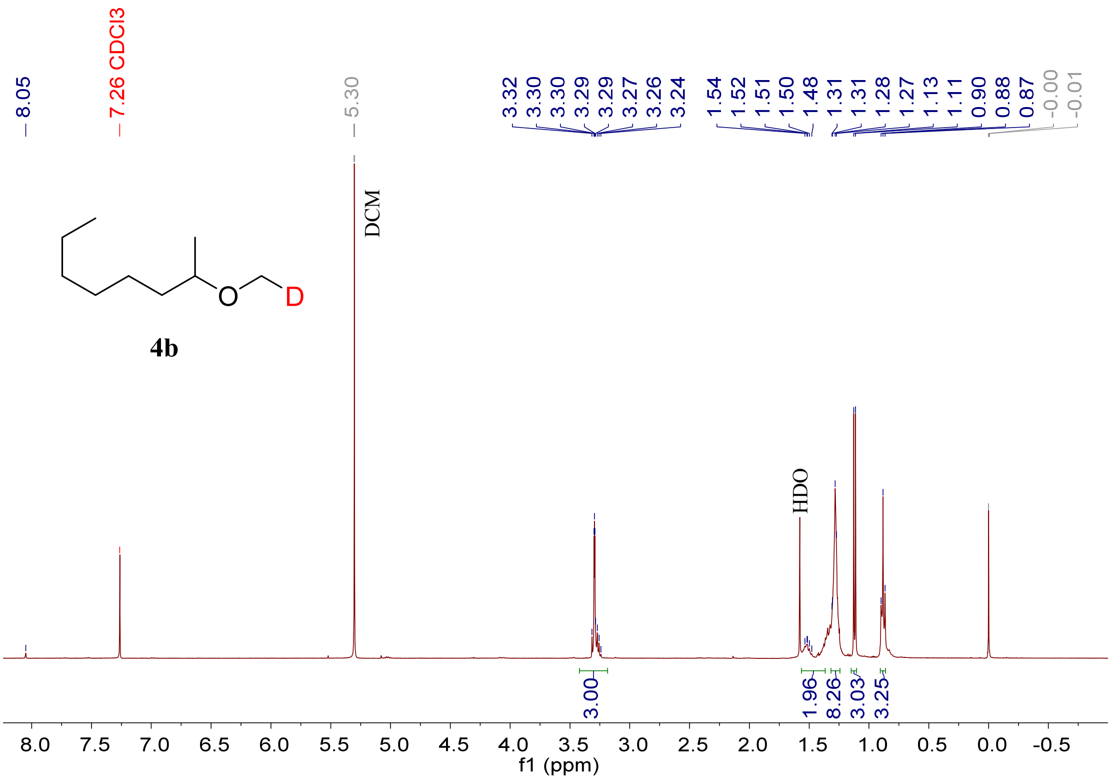

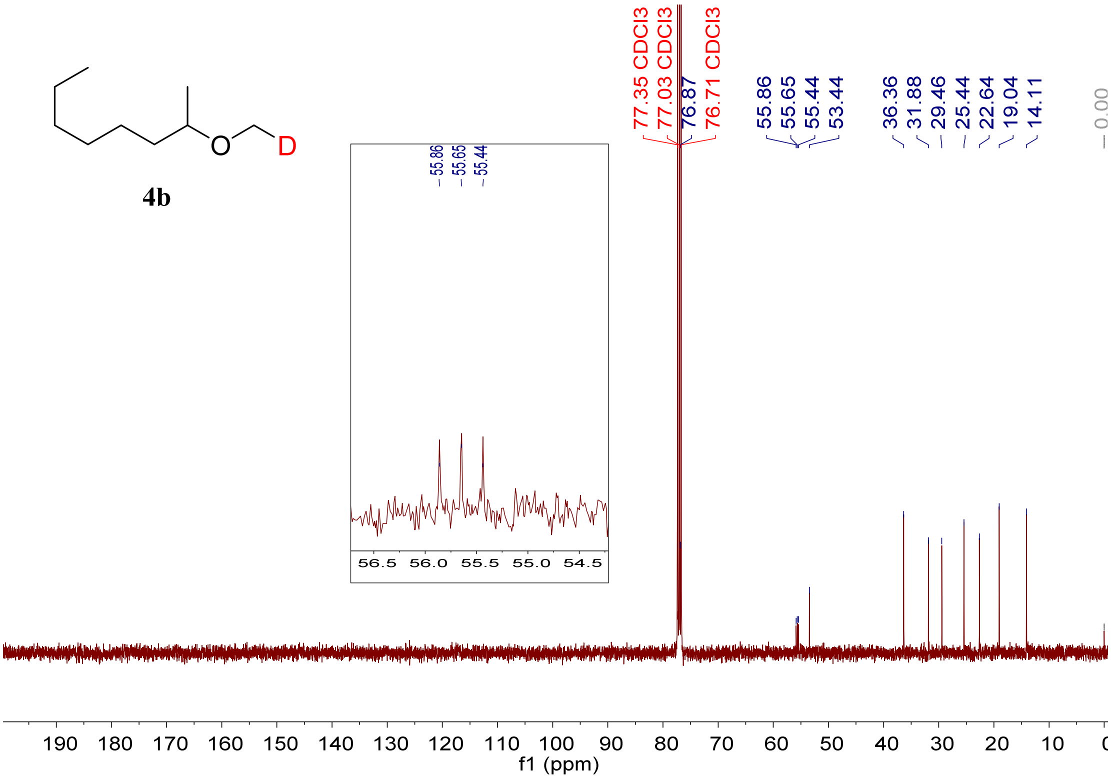

Supplementary Fig. 68. NMR spectra of 2-(Methoxy-d)-octane (4b). The splitting of C-D coupling in ^{13}C-NMR was shown with an enlarged view.

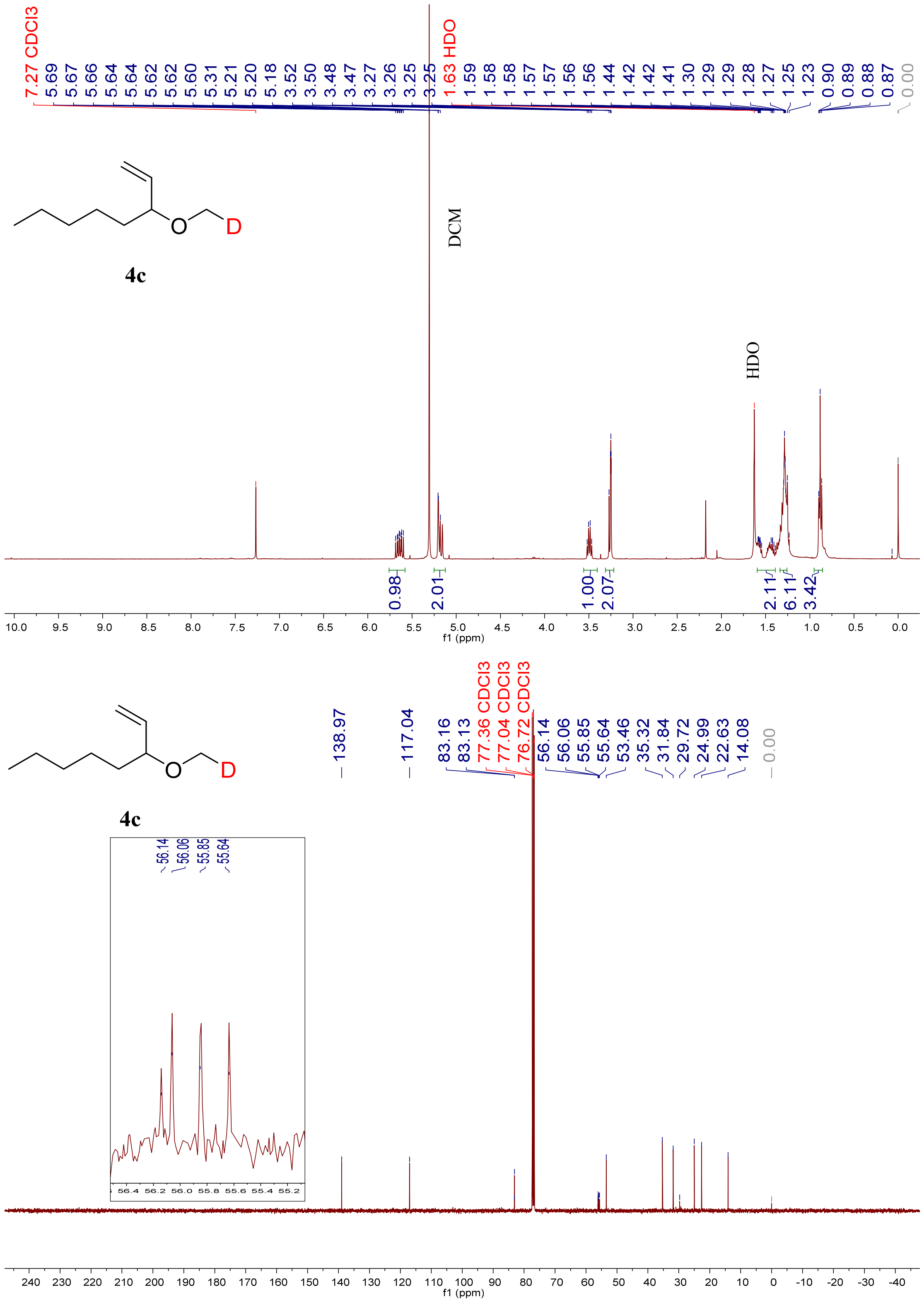

Supplementary Fig. 69. NMR spectra of 3-(Methoxy-d)oct-1-ene (4c). The splitting of C-D coupling in ^{13}C-NMR was shown with an enlarged view.

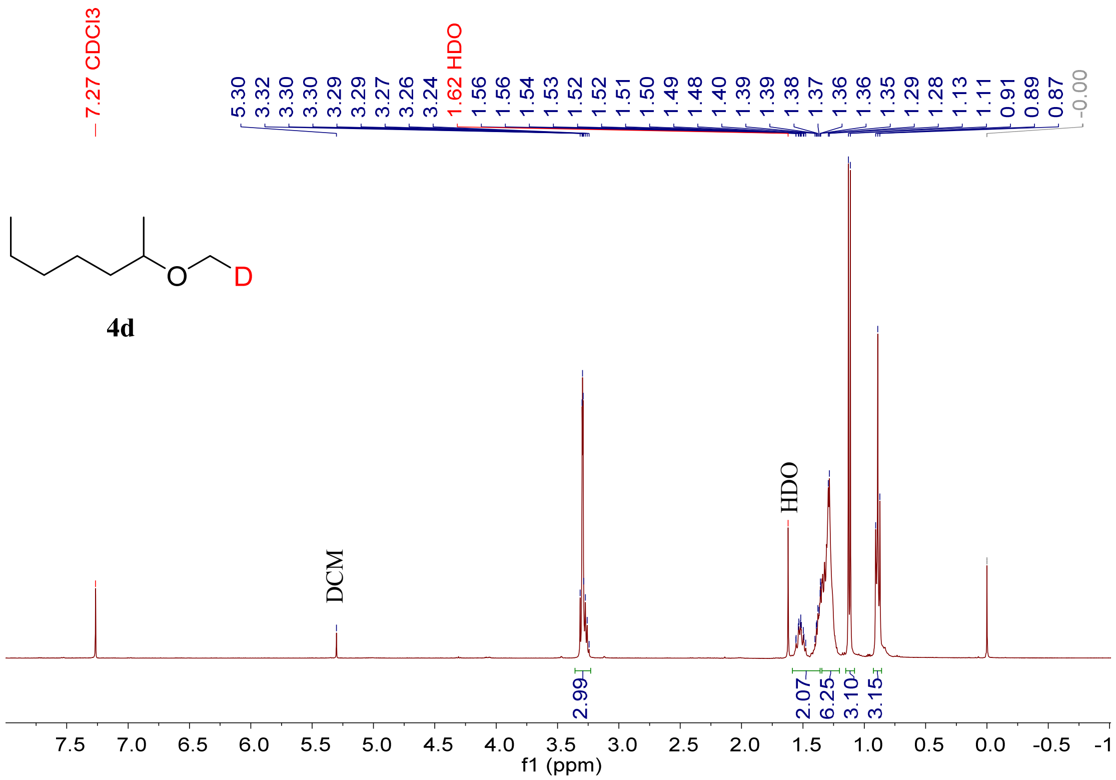

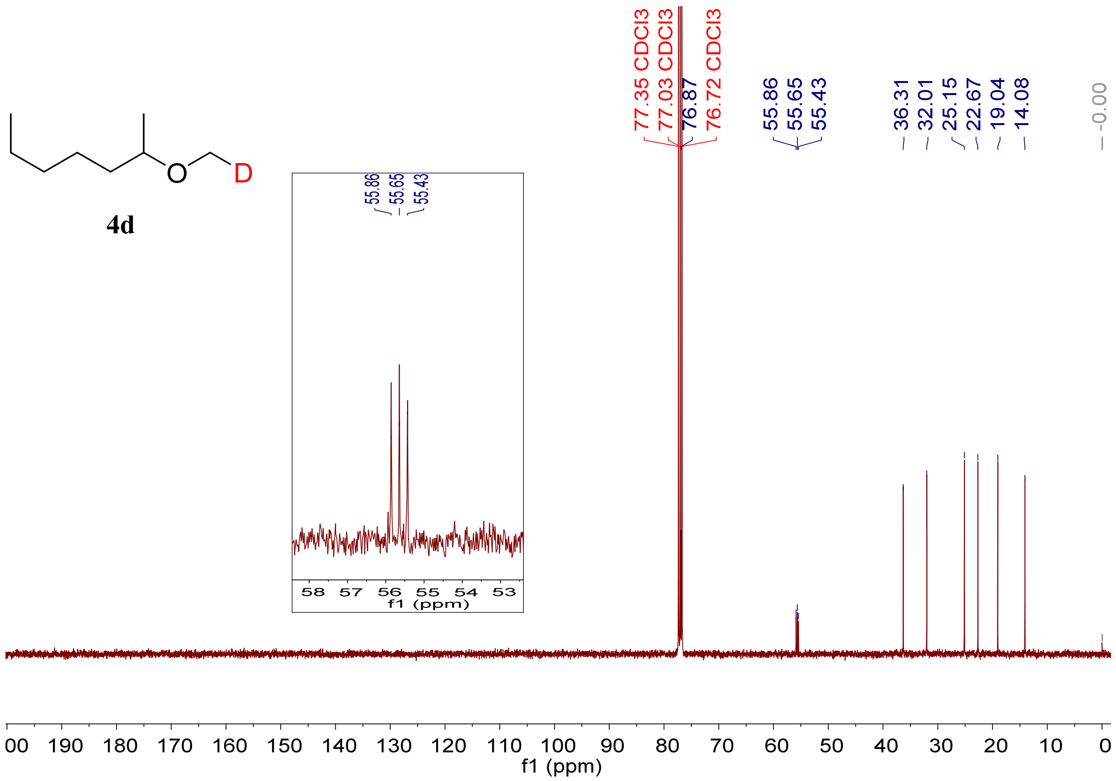

Supplementary Fig. 70. NMR spectra of 2-(Methoxy-d)heptane (4d). The splitting of C-D coupling in ^{13}C-NMR was shown with an enlarged view.

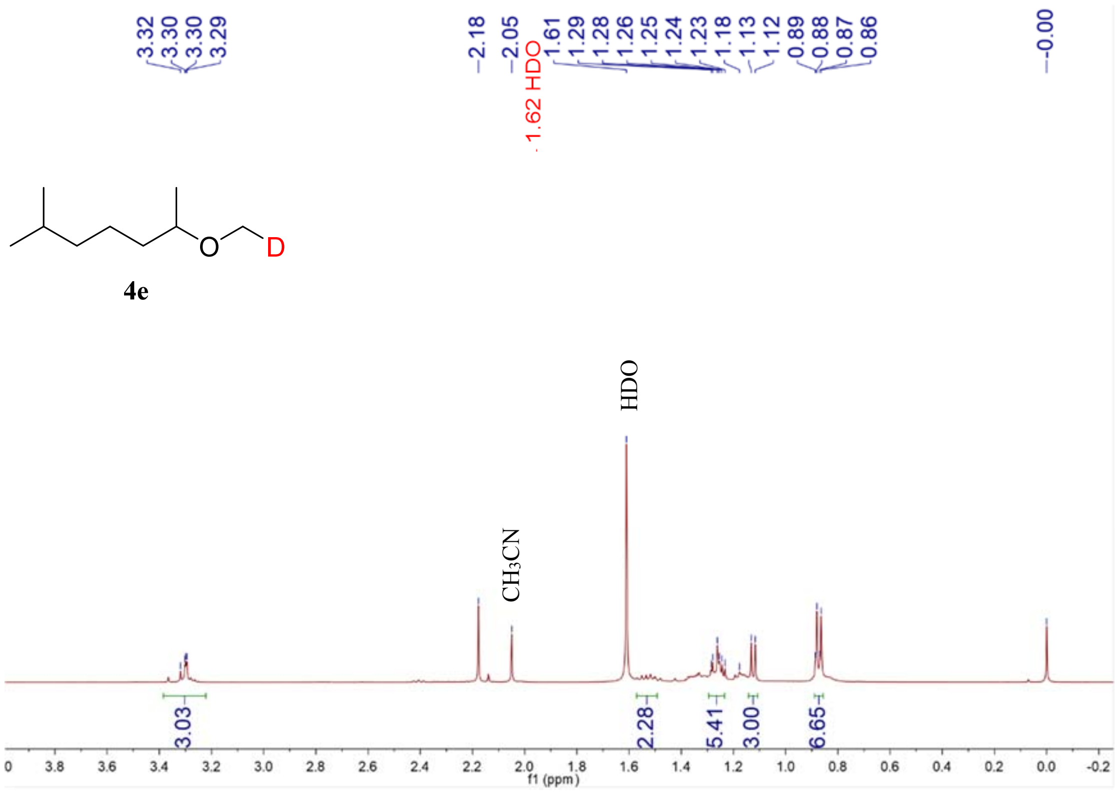

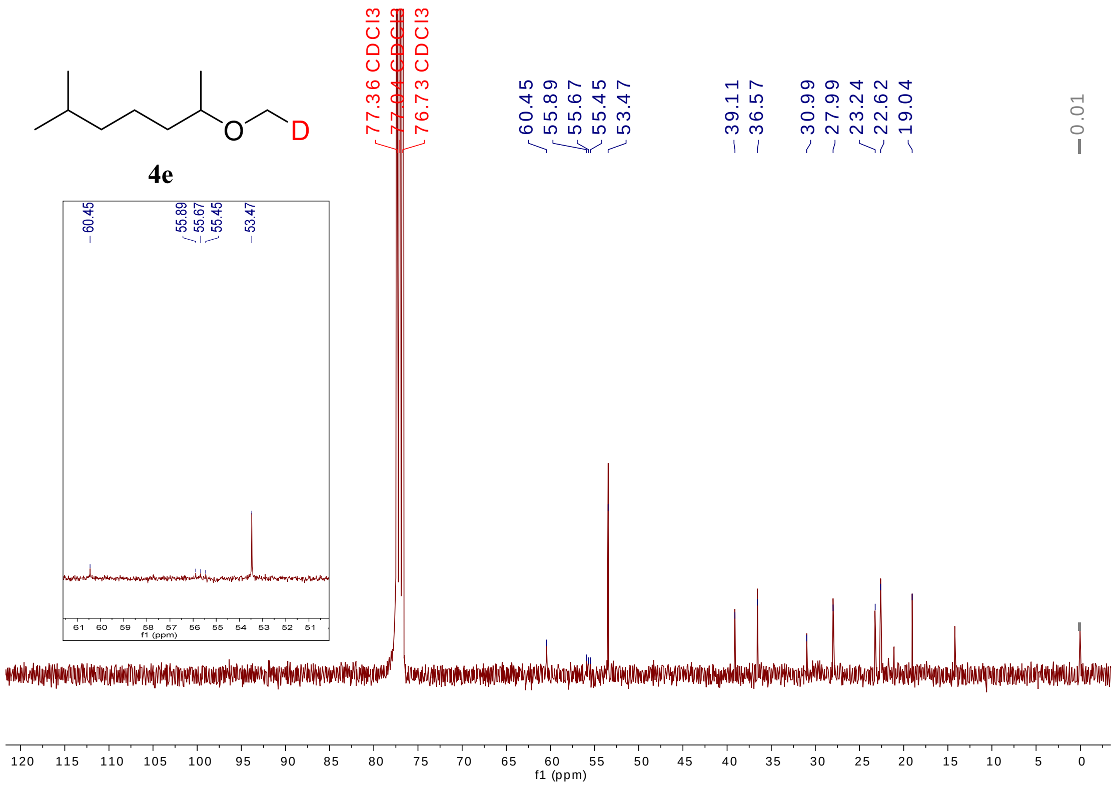

Supplementary Fig. 71. NMR spectra of 2-(Methoxy-d)-6-methylheptane (4e). The splitting of C-D coupling in ^{13}C-NMR was shown with an enlarged view.

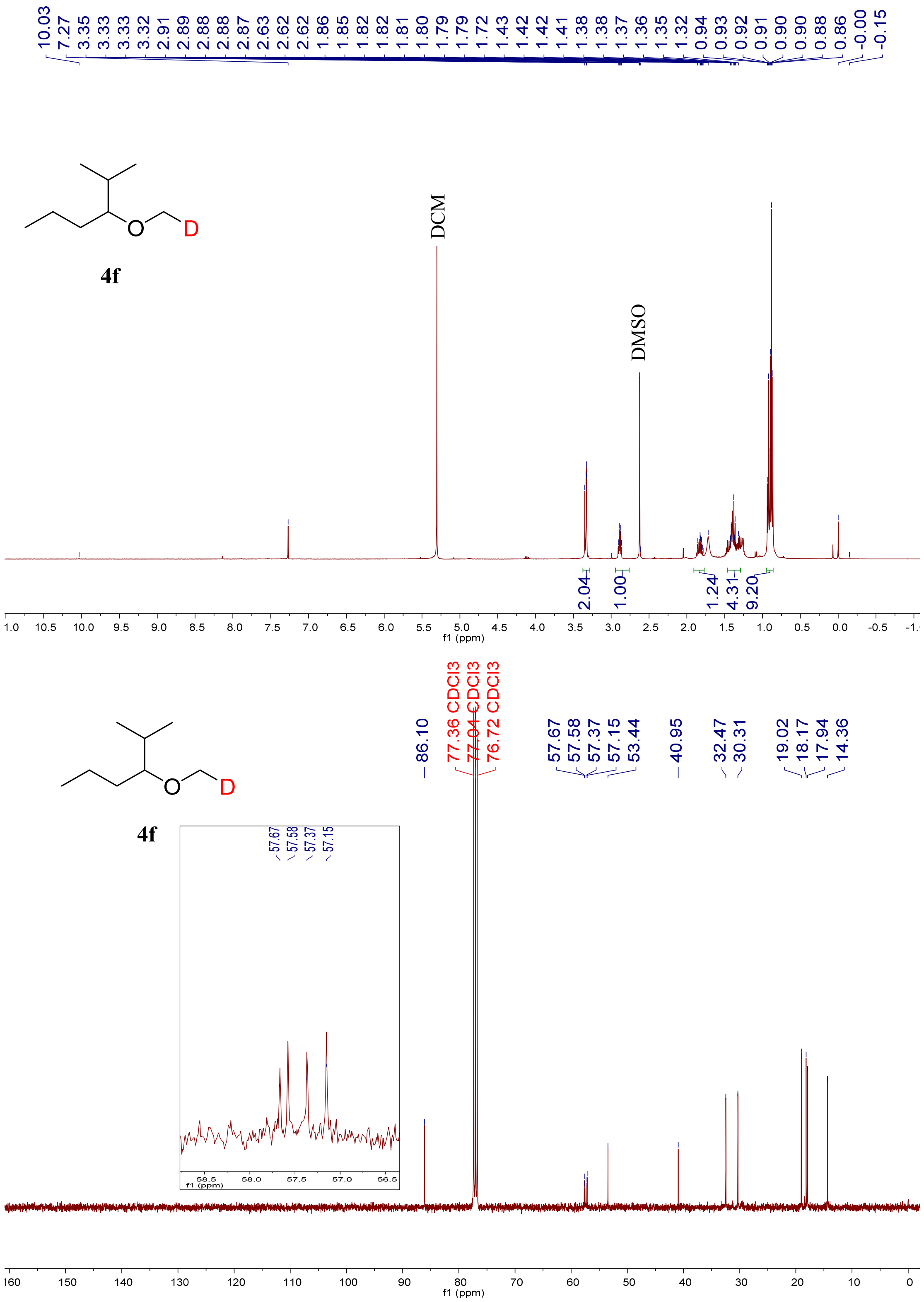

Supplementary Fig. 72. NMR spectra of 3-(Methoxy-d)-2-methylhexane (4f). The splitting of C-D coupling in ^{13}C-NMR was shown with an enlarged view.

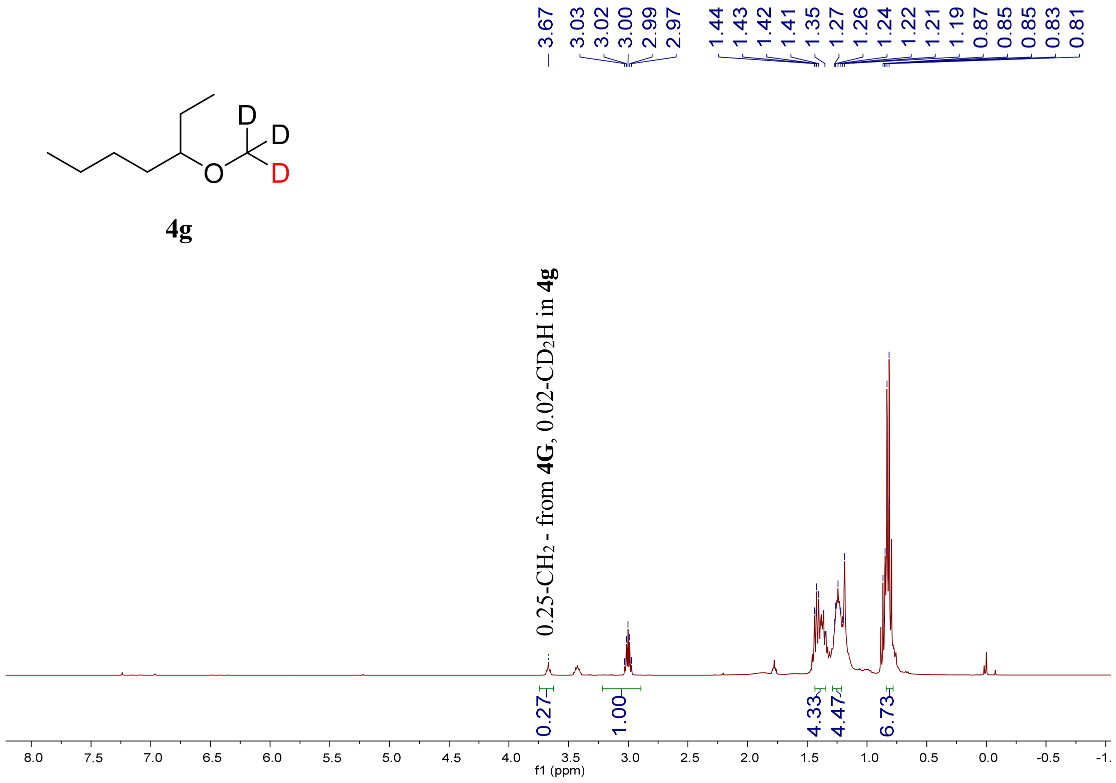

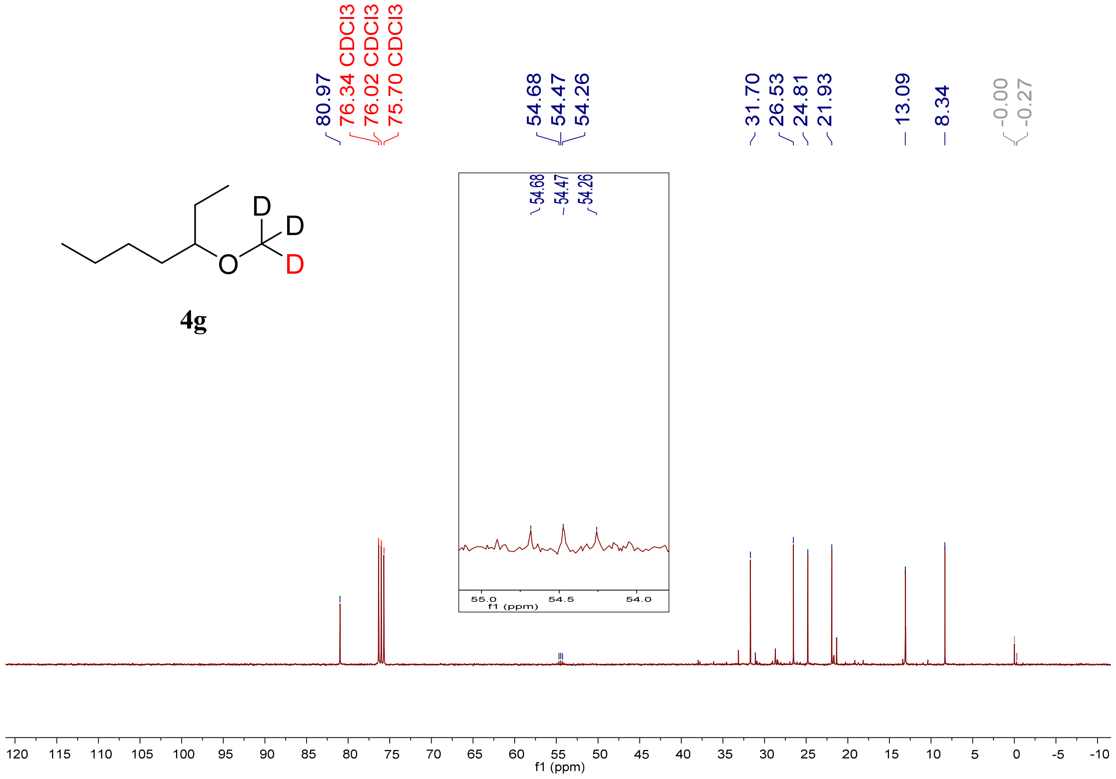

Supplementary Fig. 73. NMR spectra of 3-(Methoxy-d3)heptane (4g). The splitting of C-D coupling in ^{13}C-NMR was shown with an enlarged view.

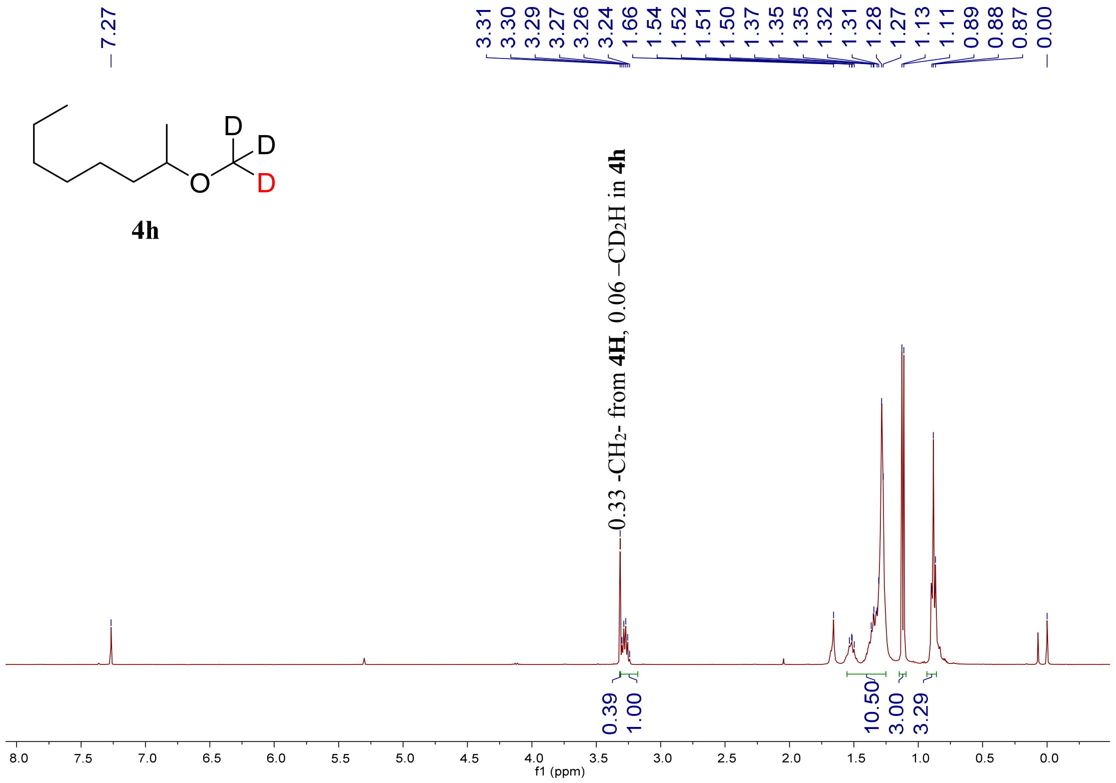

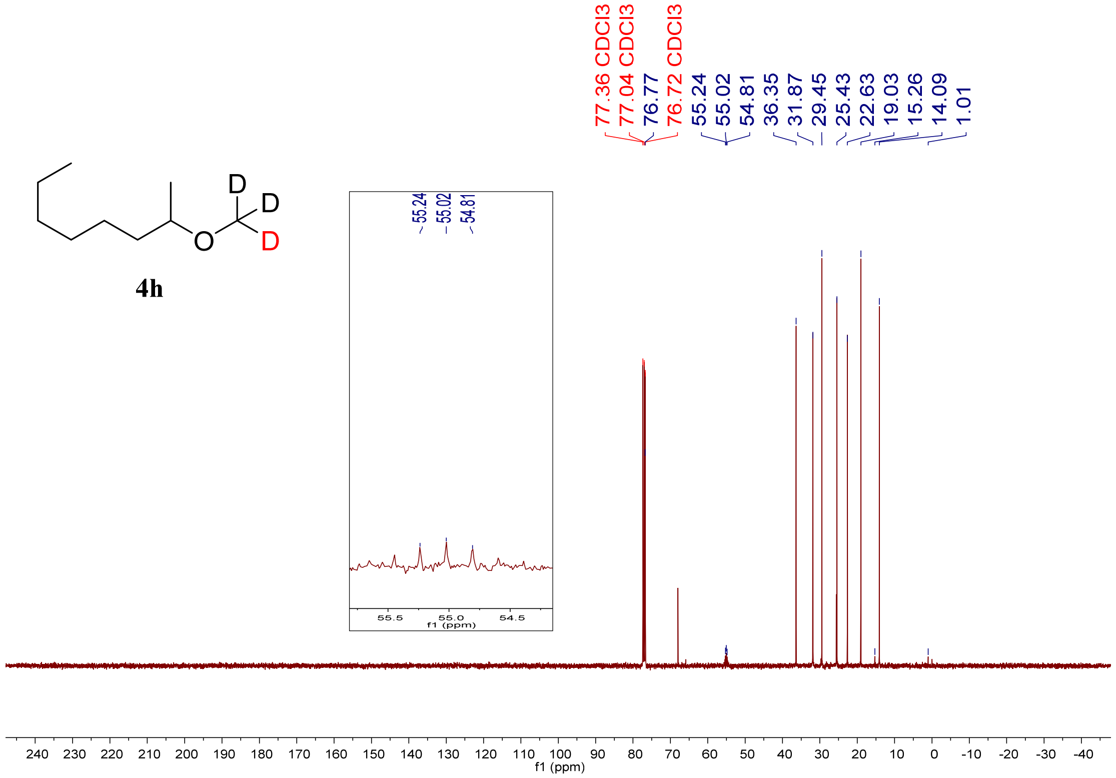

Supplementary Fig. 74. NMR spectra of 2-(Methoxy-d3)octane (4h). The splitting of C-D coupling in ^{13}C-NMR was shown with an enlarged view.

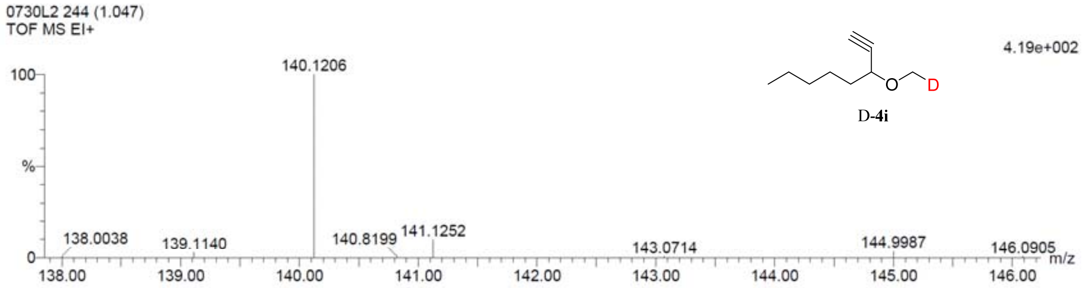

Minimum:	1.00				-1.5		
Maximum:	100.00		2.0	10.0	50.0		
Mass	RA	Calc. Mass	mDa	PPM	DBE	i-FIT	Formula
139.1140	2.66	139.1123	1.7	12.2	2.5	229.9	C9 1H15 O
140.1206	100.00	140.1201	0.5	3.6	2.0	2773014.3	C9 1H16 O
141.1252	9.68	141.1264	-1.2	-8.5	2.0	5546026.5	C9 1H15 2H O
144.9987	3.39	---					

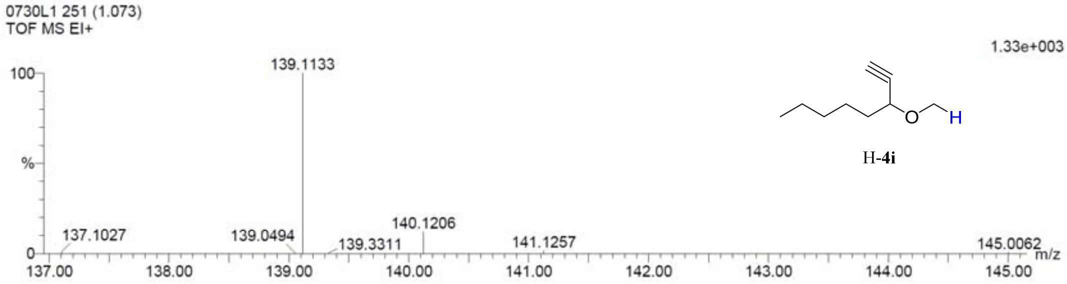

Minimum:	3.00				-1.5		
Maximum:	100.00		2.0	10.0	50.0		
Mass	RA	Calc. Mass	mDa	PPM	DBE	i-FIT	Formula
139.1133	100.00	139.1123	1.0	7.2	2.5	3.9	C9 H15 O
140.1206	11.78	140.1201	0.5	3.6	2.0	2773012.5	C9 H16 O

Supplementary Fig. 75. HRMS spectra of 3-(Methoxy-d)oct-1-yne (4i) and the corresponding 3-(Methoxy)oct-1-yne without deuterium. The D-incorporation of **4i** was determined by the comparation of HRMS between the H- and D-**4i**.

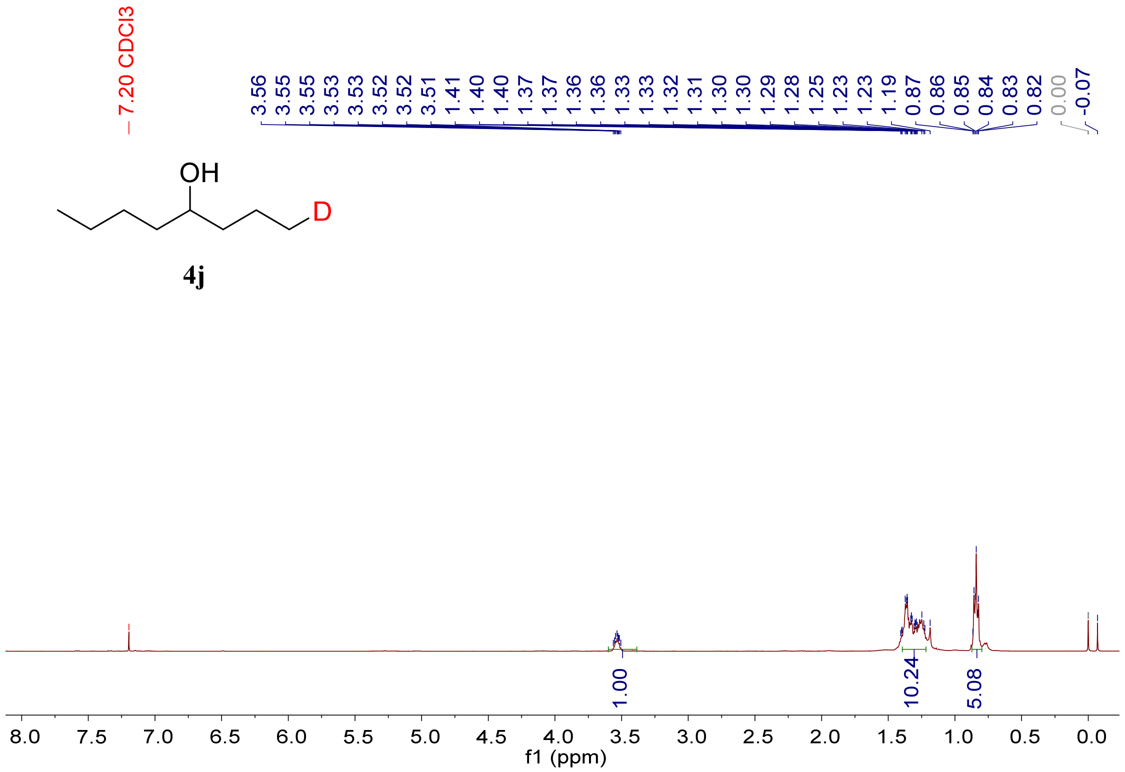

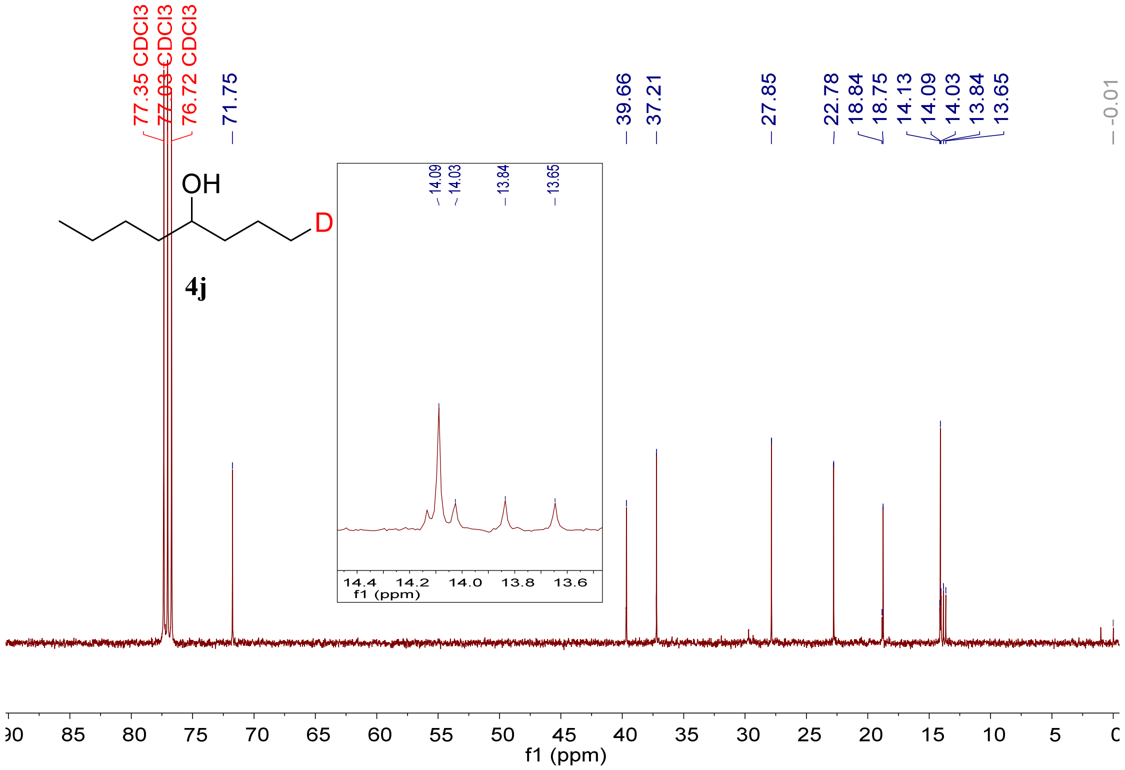

Supplementary Fig. 76. NMR spectra of Octan-8-d-4-ol (4j). The splitting of C-D coupling in ^{13}C-NMR was shown with an enlarged view.

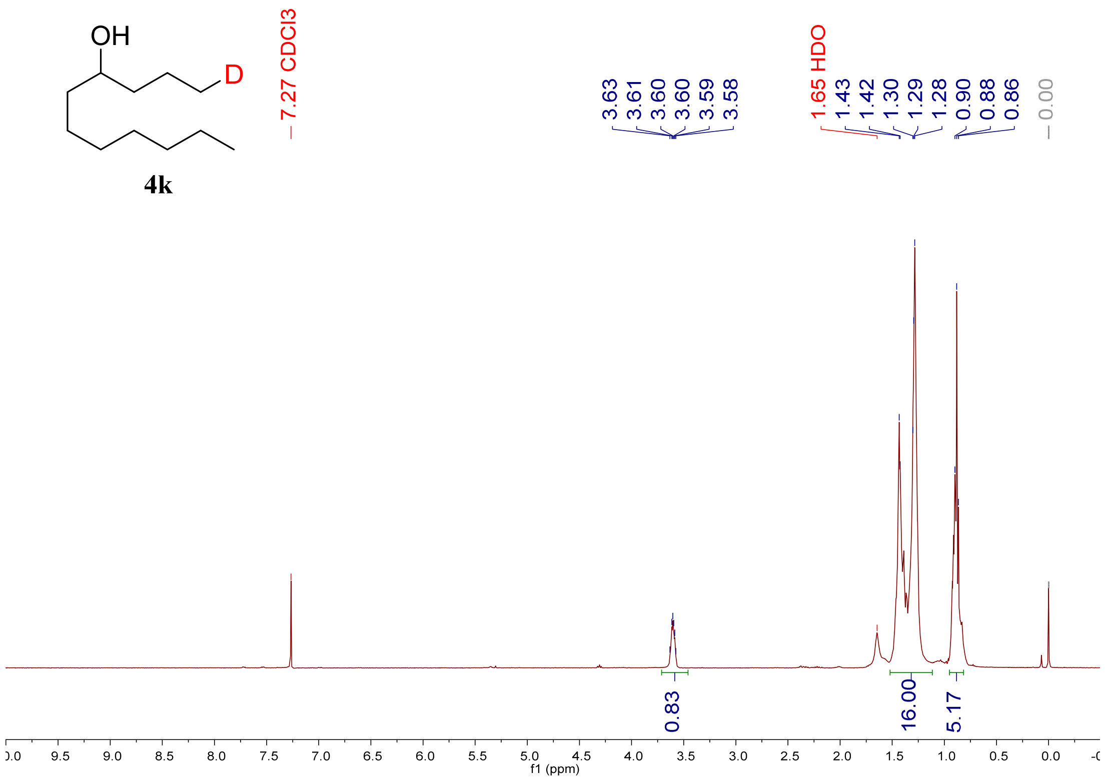

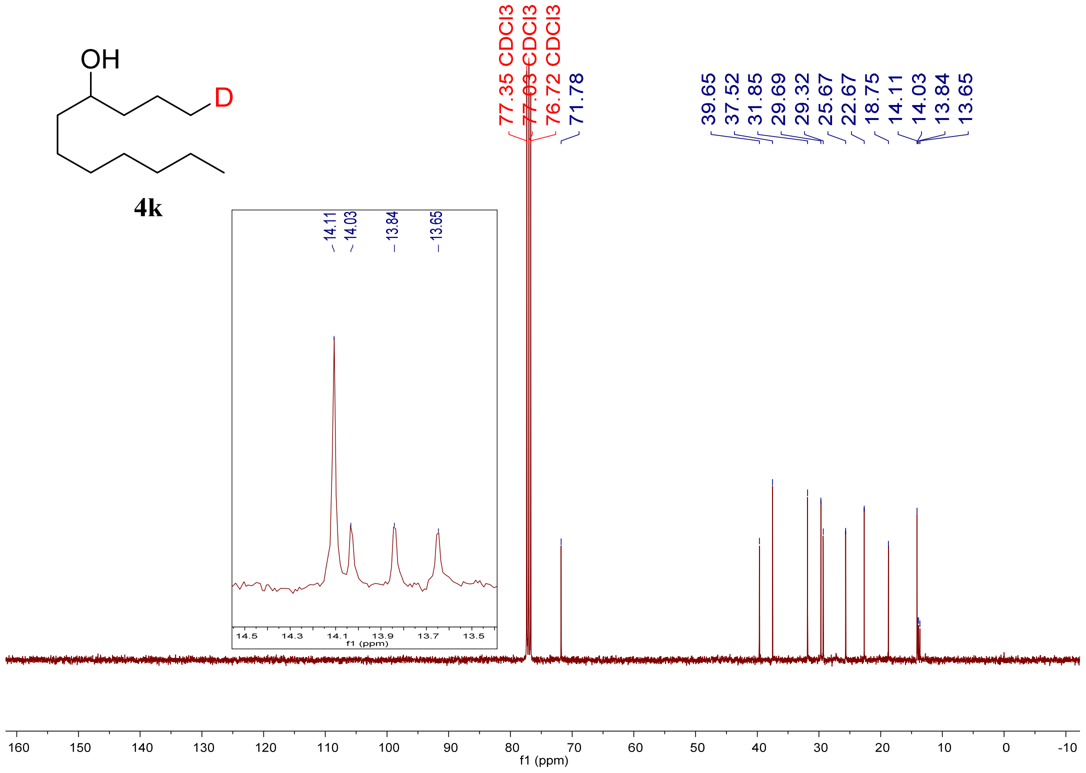

Supplementary Fig. 77. NMR spectra of Undecan-1-d-4-ol (4k). The splitting of C-D coupling in ^{13}C-NMR was shown with an enlarged view.

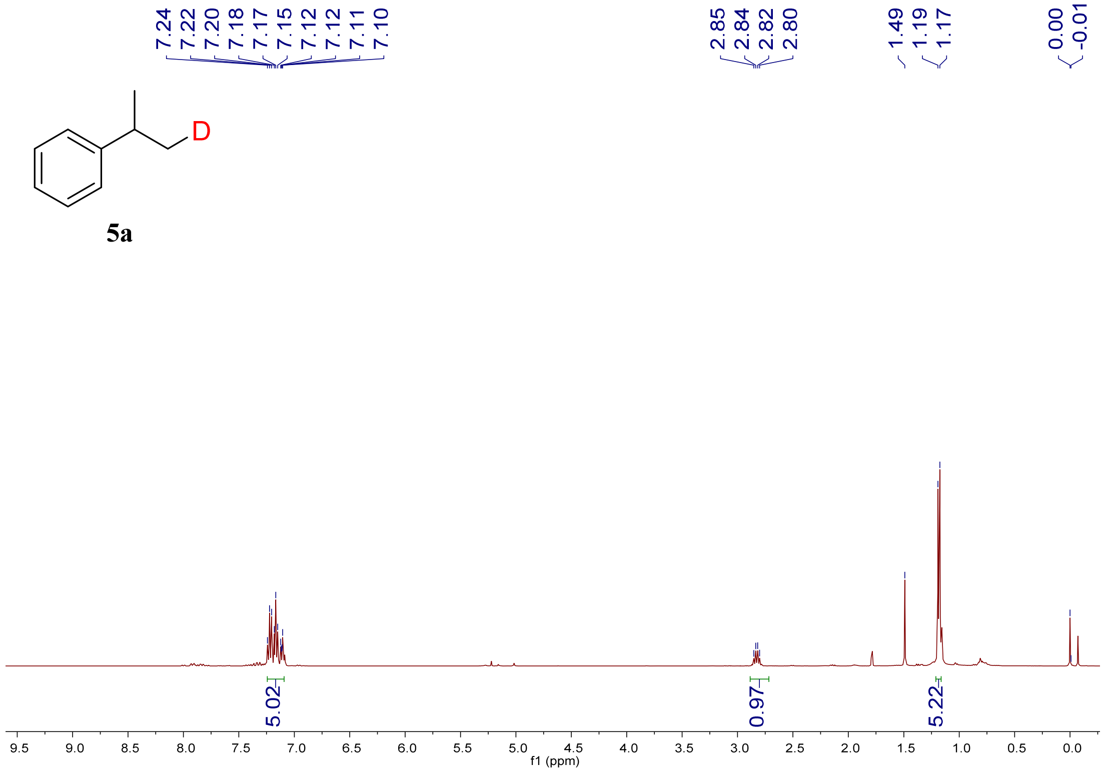
5a
7.24
7.22
7.20
7.18
7.17
7.15
7.12
7.12
7.11
7.10
2.85
2.84
2.82
2.80
1.49
1.19
1.17
0.00
-0.01
5.02
0.97
5.22
f1 (ppm)

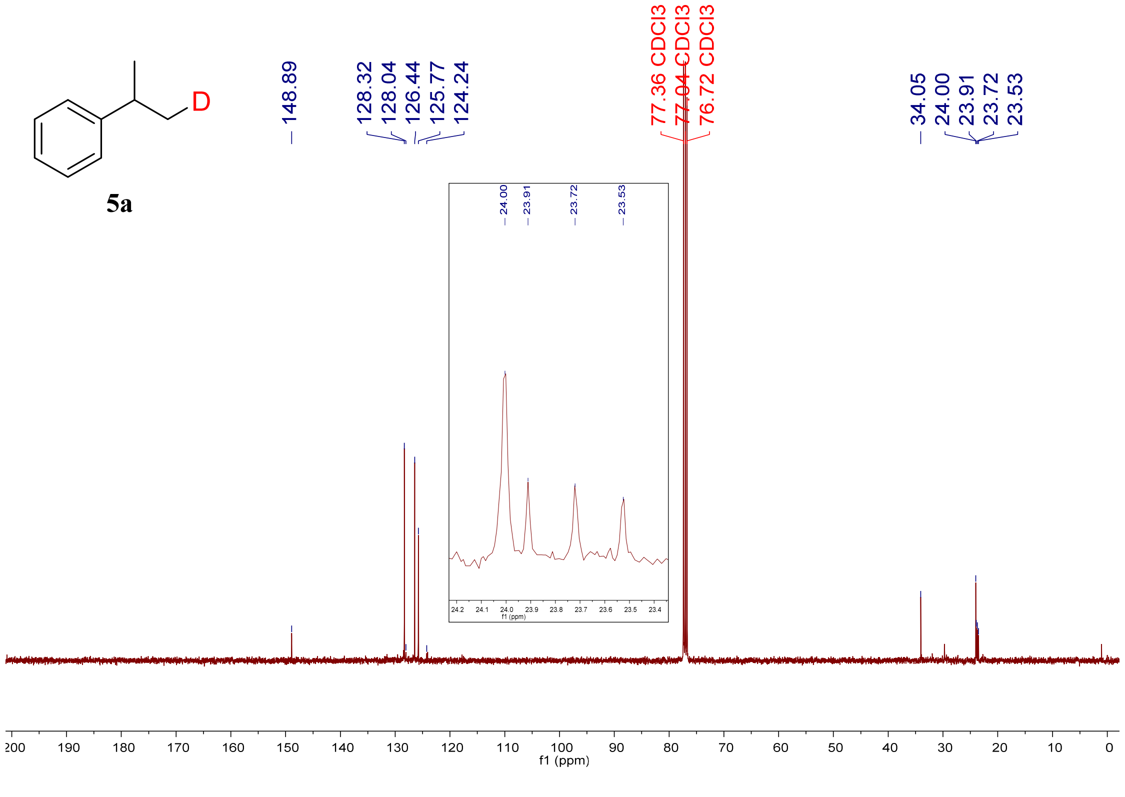
5a
148.89
128.32
128.04
126.44
125.77
124.24
77.36 CDCl3
77.04 CDCl3
76.72 CDCl3
34.05
24.00
23.91
23.72
23.53
f1 (ppm)

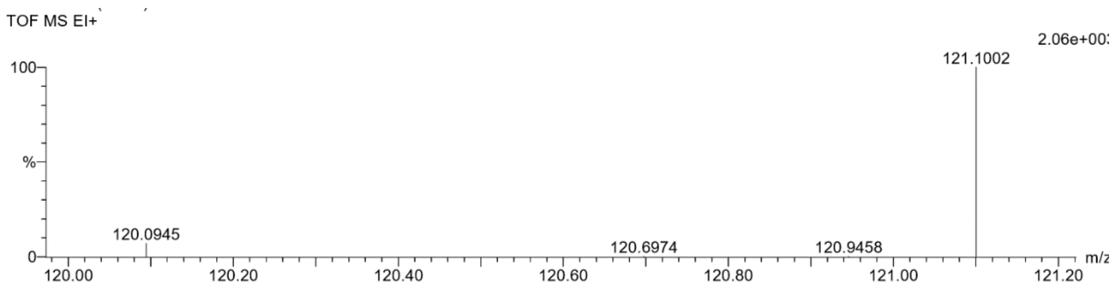

Minimum:	5.00				-1.5		
Maximum:	100.00		1.0	10.0	50.0		
Mass	RA	Calc. Mass	mDa	PPM	DBE	i-FIT	Formula
120.0945	7.00	120.0939	0.6	5.0	4.0	2774028.5	C9 1H12
121.1002	100.00	121.1002	0.0	0.0	4.0	5546843.0	C9 1H11 2H

Supplementary Fig. 78. NMR and HRMS spectra of (Propan-2-yl-1-d)benzene (5a). The splitting of C-D coupling in ^{13}C-NMR was shown with an enlarged view.

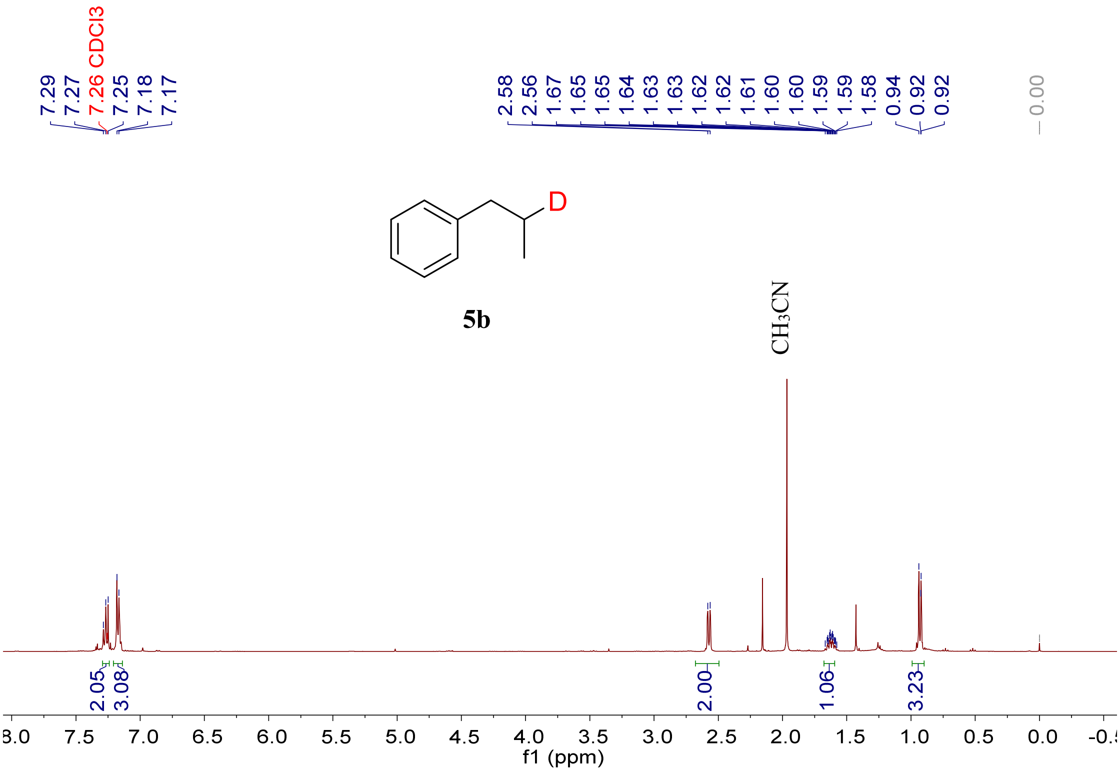

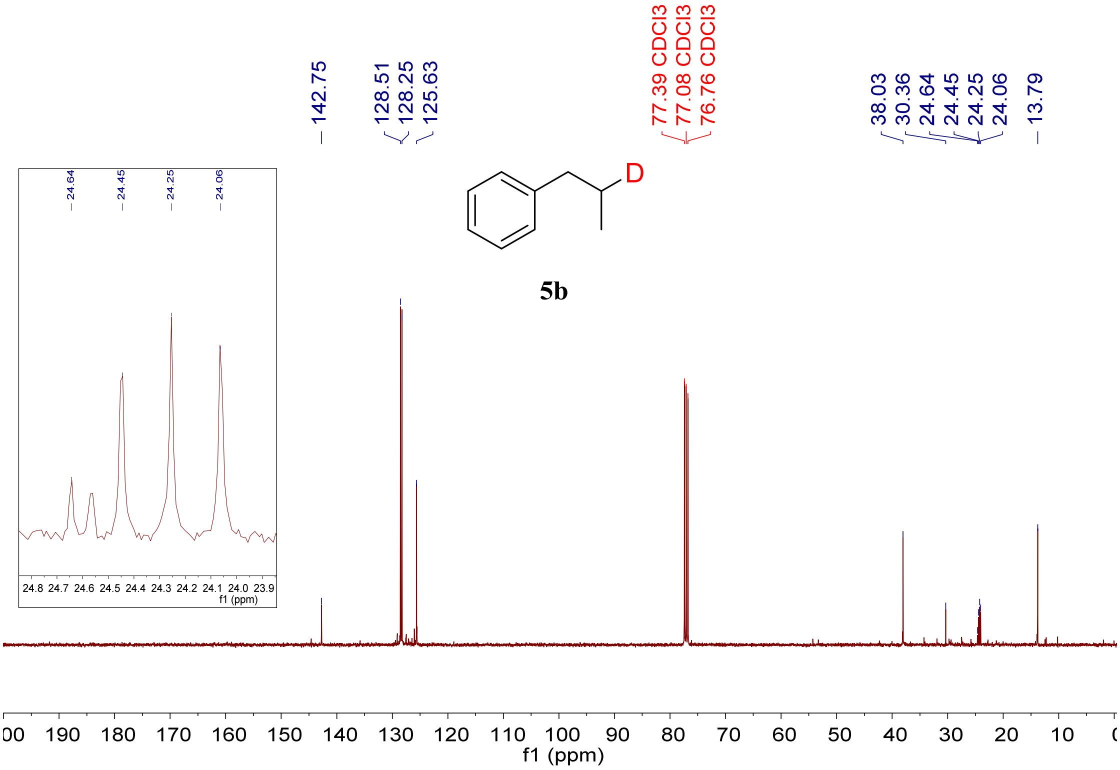

Supplementary Fig. 79. NMR spectra of (Propyl-2-d)benzene (5b). The splitting of C-D coupling in ^{13}C-NMR was shown with an enlarged view.

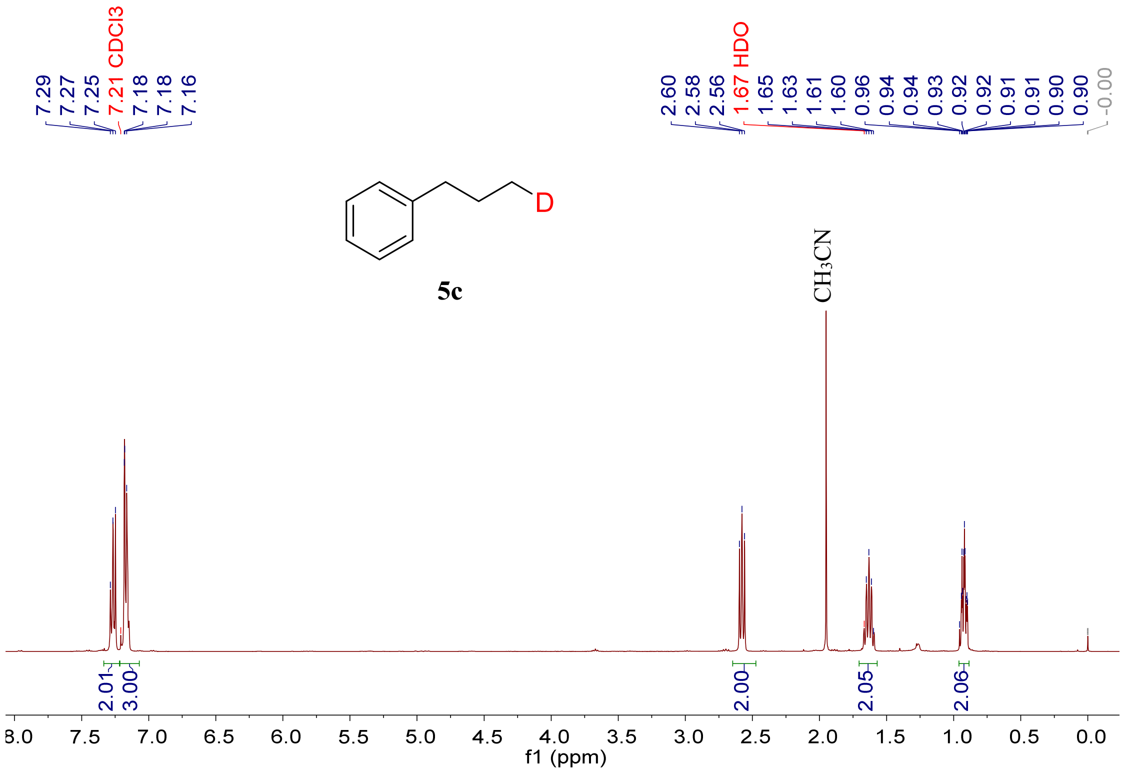

7.29 7.27 7.25 7.21 CDCl3 7.18 7.18 7.16
2.60 2.58 2.56 1.67 HDO 1.65 1.63 1.61 1.60 0.96 0.94 0.94 0.93 0.92 0.92 0.91 0.91 0.90 0.90 -0.00
D
5c
CH3CN
2.01 3.00 2.00 2.05 2.06
f1 (ppm)

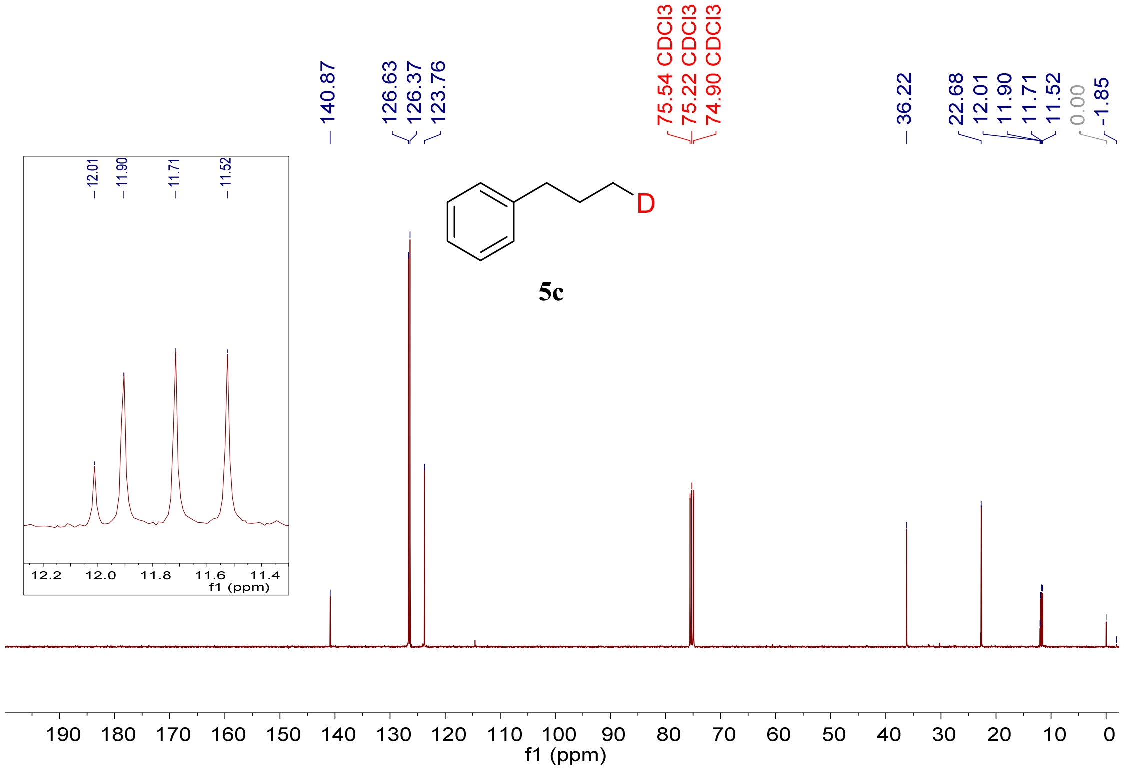

140.87 126.63 126.37 123.76
75.54 CDCl3 75.22 CDCl3 74.90 CDCl3
36.22 22.68 12.01 11.90 11.71 11.52 0.00 -1.85
12.01 11.90 11.71 11.52
f1 (ppm)
D
5c
f1 (ppm)

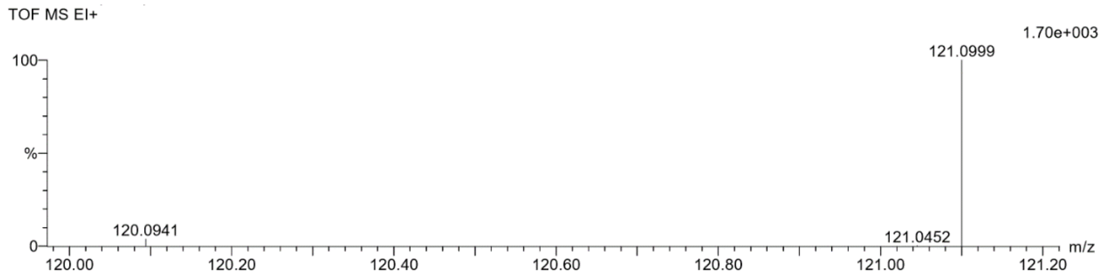

Minimum:	1.00				-1.5		
Maximum:	100.00		1.0	10.0	50.0		
Mass	RA	Calc. Mass	mDa	PPM	DBE	i-FIT	Formula
120.0941	3.81	120.0939	0.2	1.7	4.0	2773859.5	C9 1H12
121.0999	100.00	121.1002	-0.3	-2.5	4.0	5546673.5	C9 1H11 2H

Supplementary Fig. 80. NMR and HRMS spectra of (Propyl-3-d)benzene (5c). The splitting of C-D coupling in ^{13}C-NMR was shown with an enlarged view.

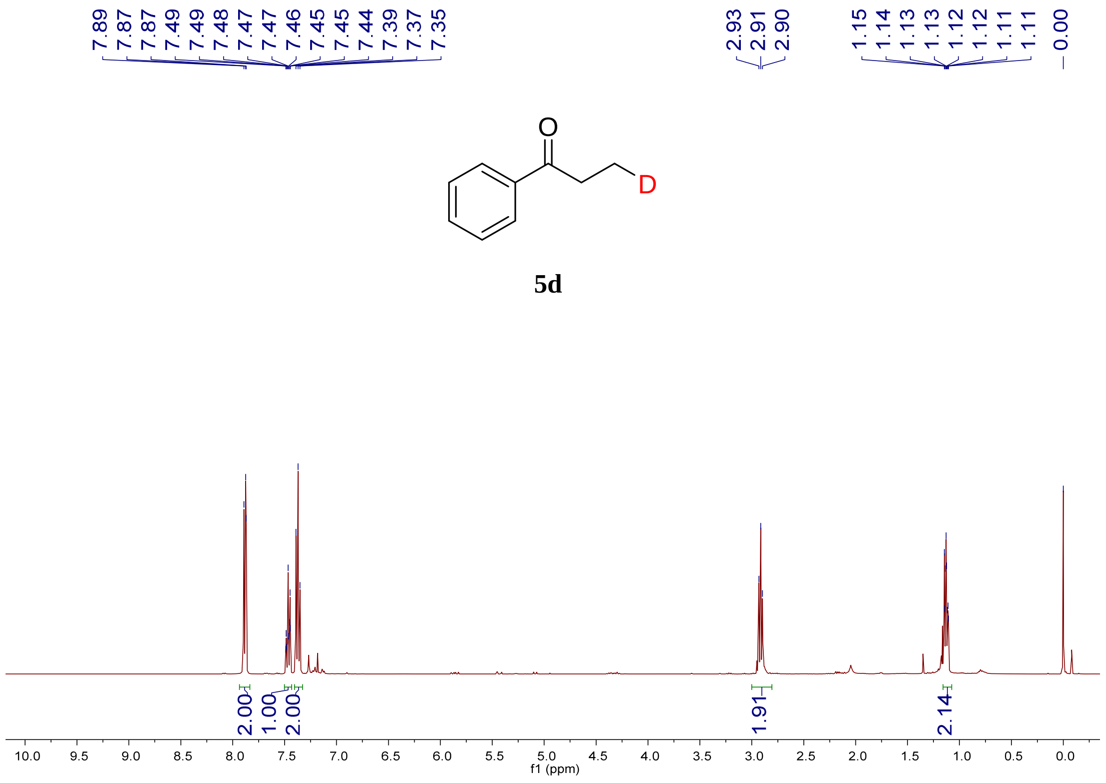

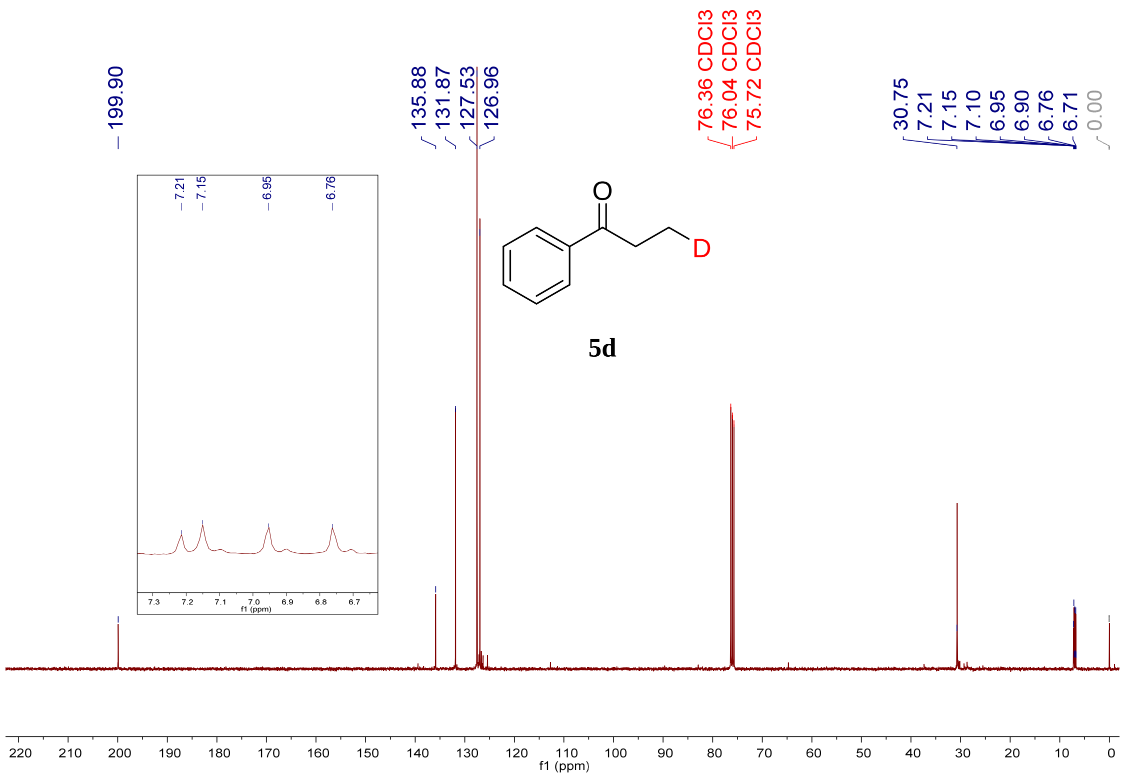

Supplementary Fig. 81. NMR spectra of 1-Phenyl-1-propanone-3-d (5d). The splitting of C-D coupling in ^{13}C-NMR was shown with an enlarged view.

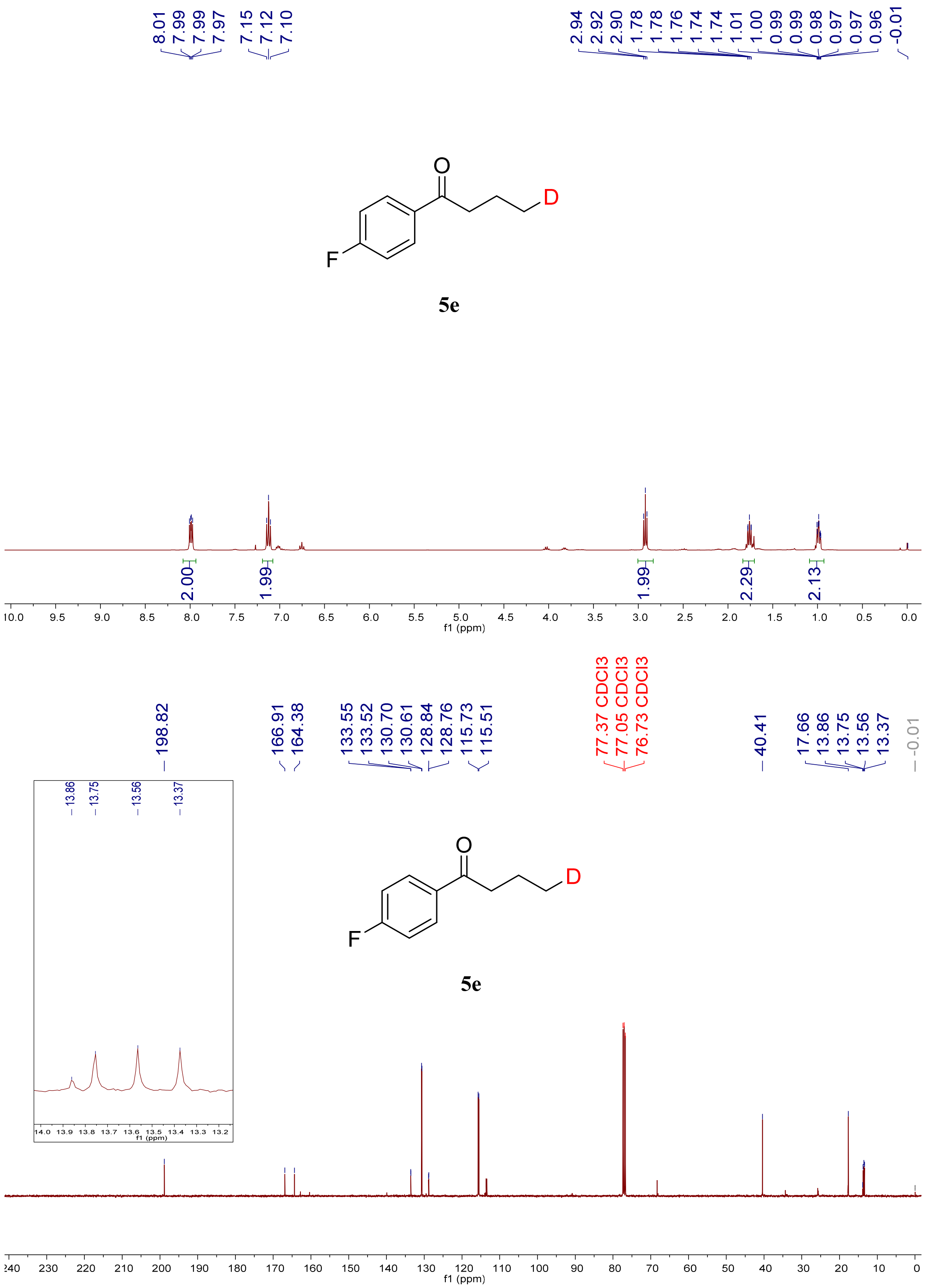

Supplementary Fig. 82. NMR spectra of 1-(4-Fluorophenyl)butan-1-one-4-d (5e). The splitting of C-D coupling in ^{13}C-NMR was shown with an enlarged view.

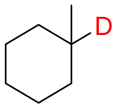

5f

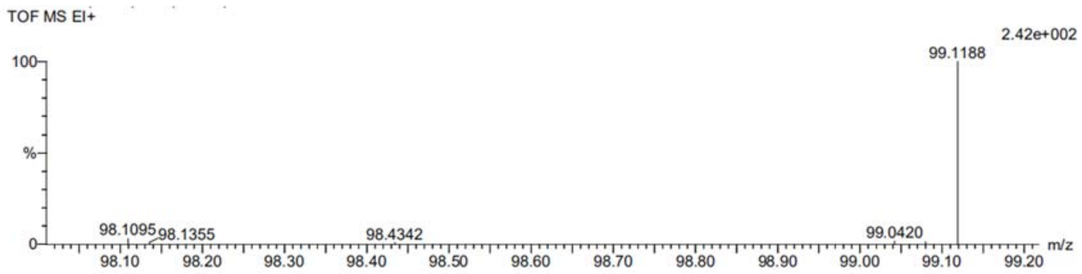

Minimum:	2.00				-1.5		
Maximum:	100.00		4.0	10.0	50.0		
Mass	RA	Calc. Mass	mDa	PPM	DBE	i-FIT	Formula
98.1095	2.93	98.1065	3.0	30.6	2.0	0.2	C7 1H10 2H2
		98.1080	1.5	15.3	1.5	127.9	C7 1H12 2H
		98.1096	-0.1	-1.0	1.0	2773135.8	C7 1H14
99.1188	100.00	99.1158	3.0	30.3	1.0	2773035.8	C7 1H13 2H
		99.1174	1.4	14.1	0.5	5546091.5	C7 1H15

Supplementary Fig. 83. HRMS spectrum of Methylcyclohexane-1-d (5f).

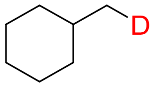

5g

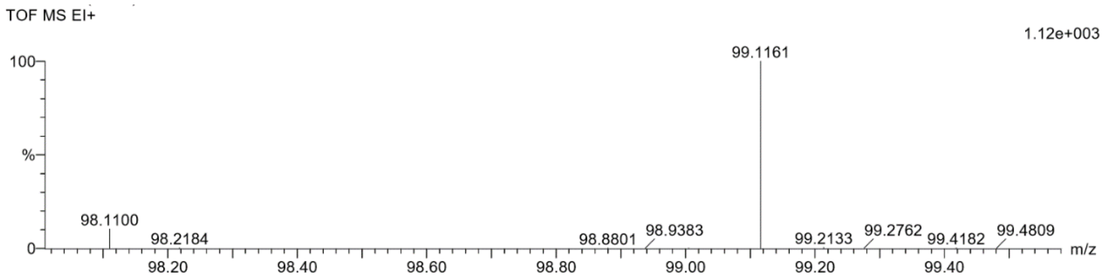

Minimum:	5.00				-1.5		
Maximum:	100.00		1.0	10.0	50.0		
Mass	RA	Calc. Mass	mDa	PPM	DBE	i-FIT	Formula
98.1100	10.39	98.1096	0.4	4.1	1.0	2773563.8	C7 1H14
99.1161	100.00	99.1158	0.3	3.0	1.0	2773317.5	C7 1H13 2H

Supplementary Fig. 84. HRMS spectrum of (Methyl-d)cyclohexane (5g).

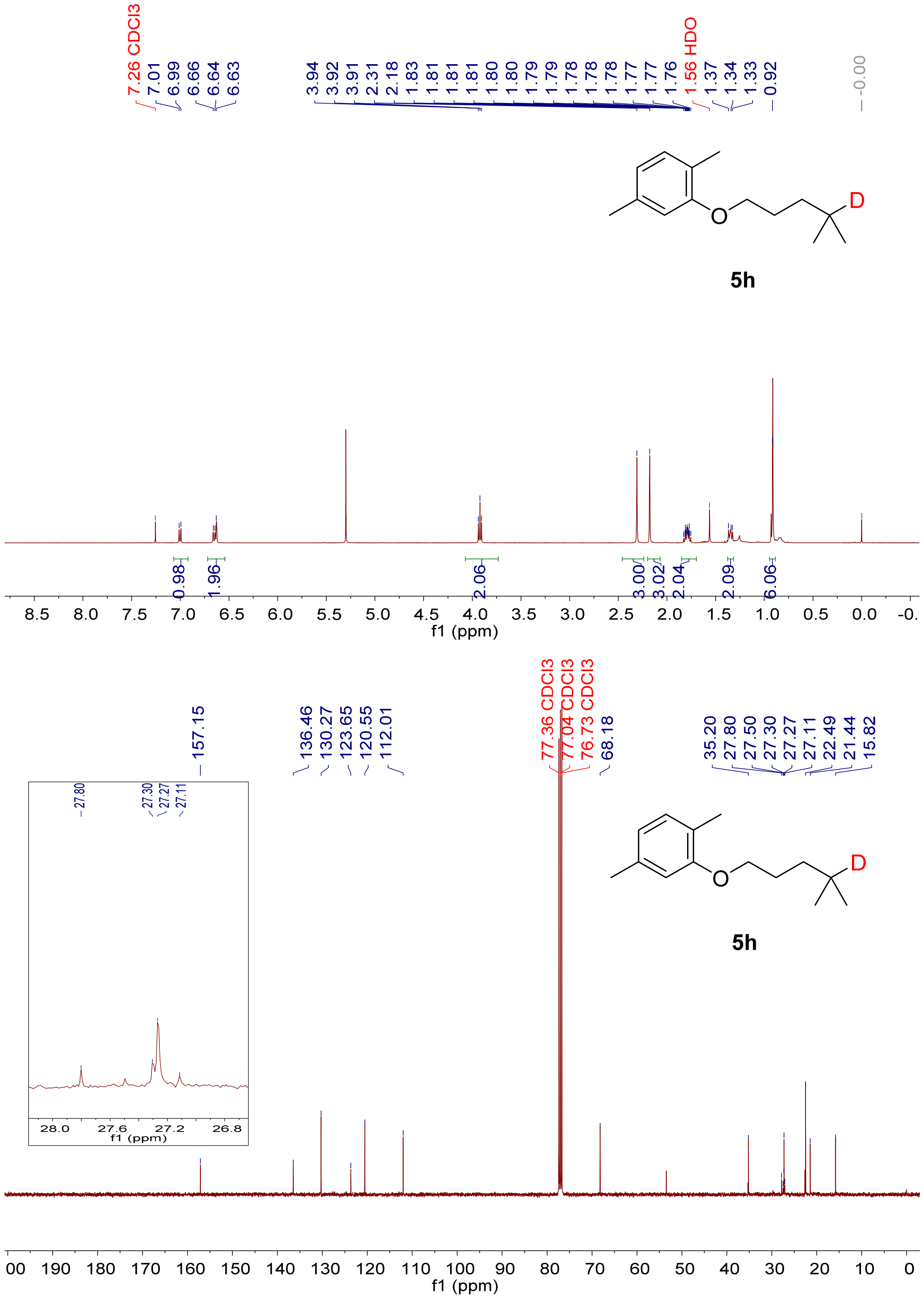

Supplementary Fig. 85. NMR spectra of 1,4-dimethyl-2-((4-methylpentyl-4-d)oxy)benzene (5h). The splitting of C-D coupling in ^{13}C-NMR was shown with an enlarged view.

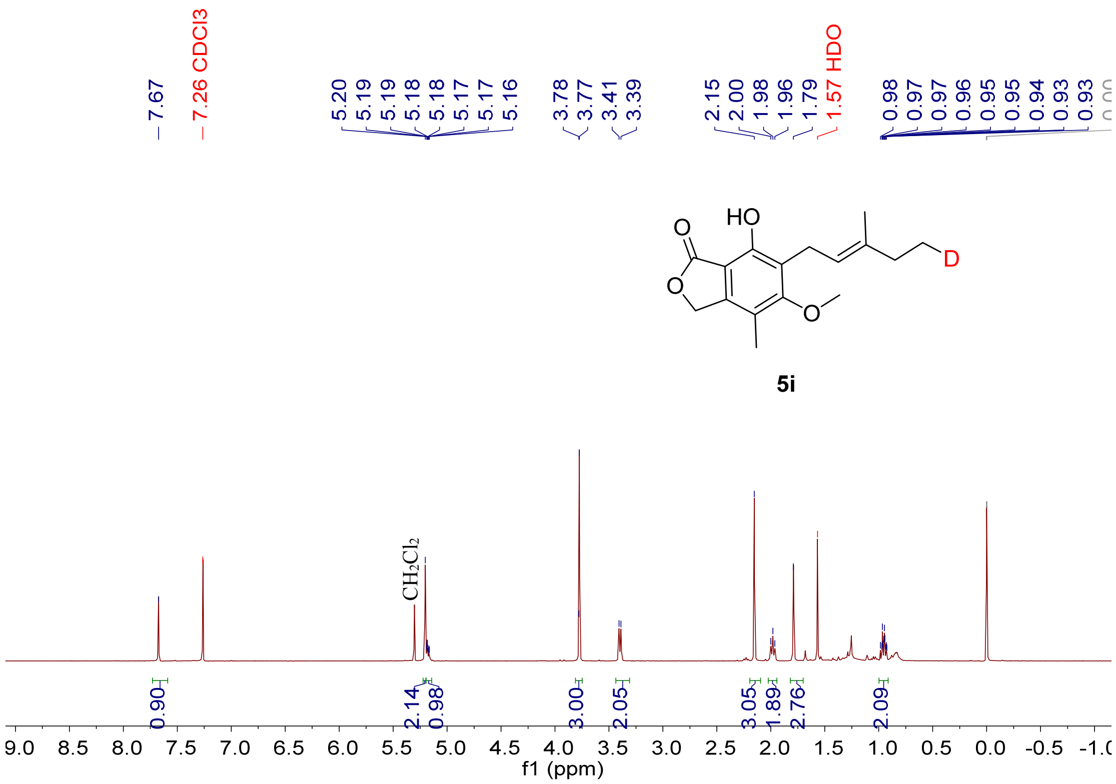

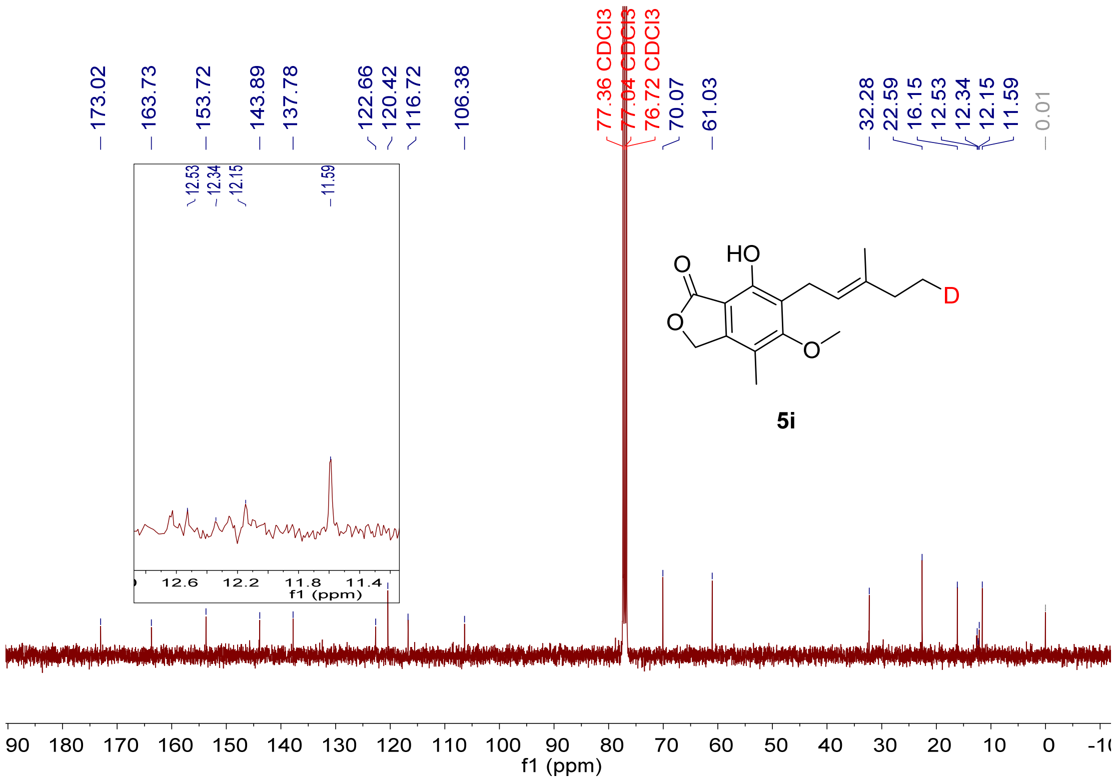

Supplementary Fig. 86. NMR spectra of (E)-7-hydroxy-5-methoxy-4-methyl-6-(3-methylpent-2-en-1-yl-5-d)isobenzofuran-1(3H)-one (5i). The splitting of C-D coupling in ^{13}C-NMR was shown with an enlarged view.

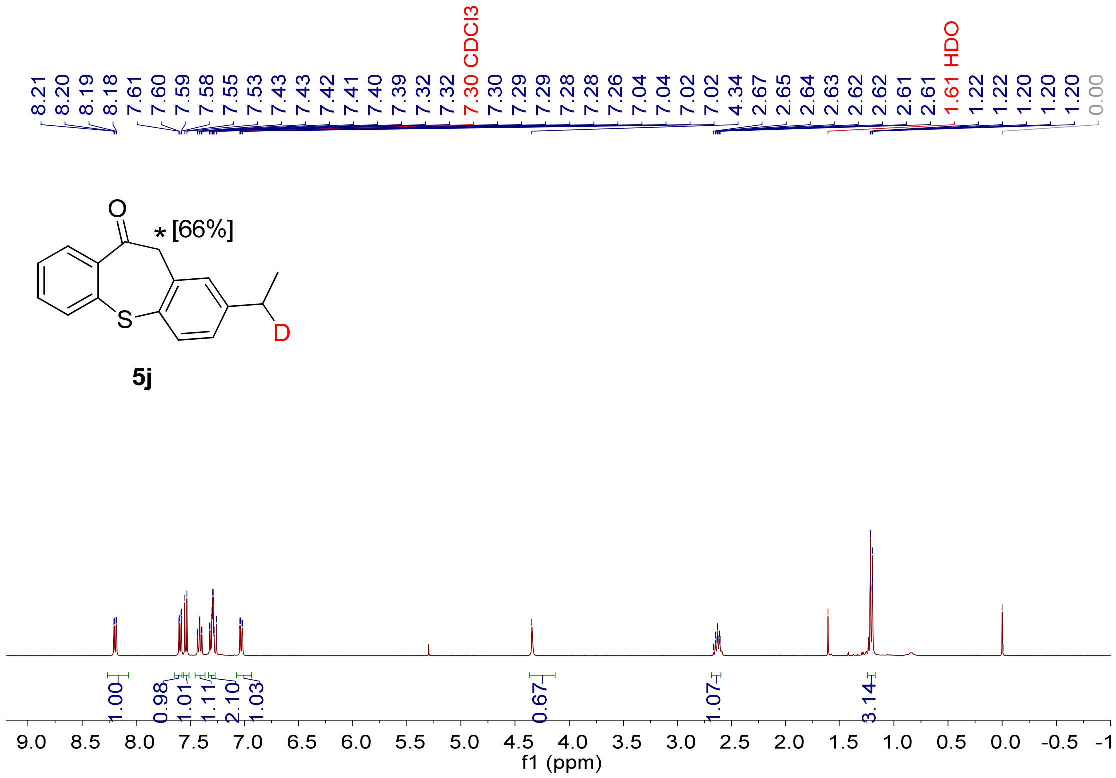

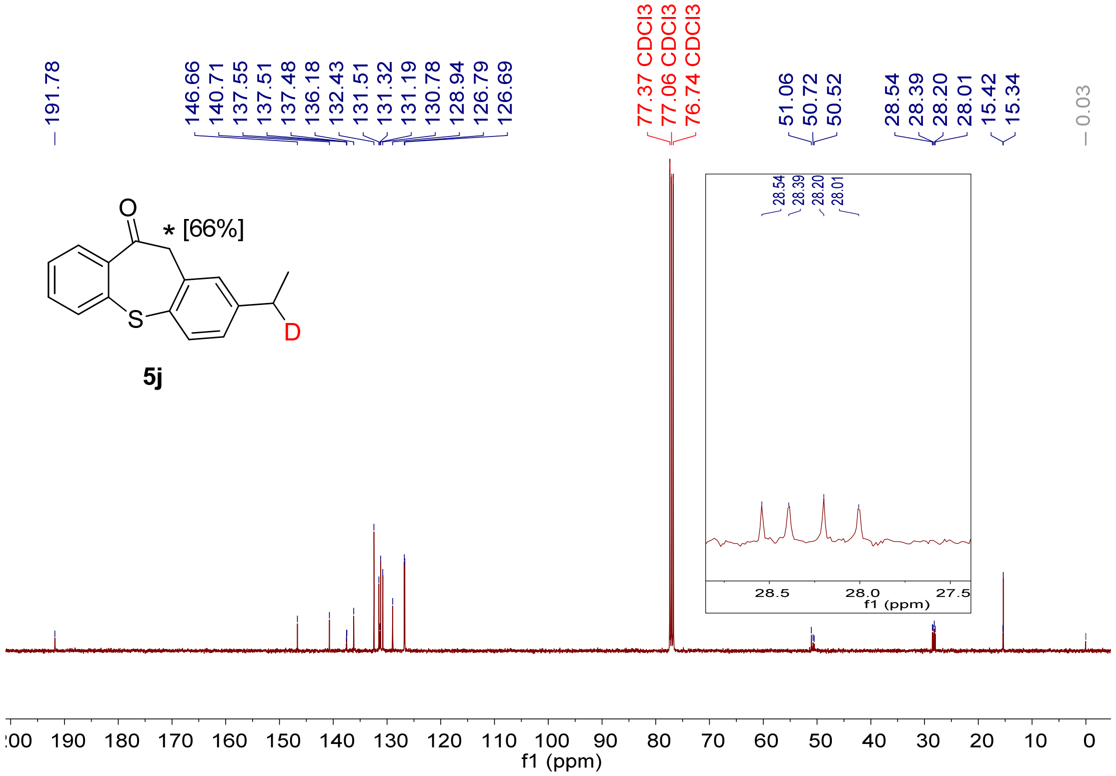

Supplementary Fig. 87. NMR spectra of 2-(ethyl-1-d)dibenzo[b,f]thiepin-10(11H)-one (5j). The splitting of C-D coupling in ^{13}C-NMR was shown with an enlarged view. The signal at δ 4.34 for two protons on C11 (labeled with star) shows reduced intensity, indicating some deuteration also occurs at this position due to tautomeric exchange of the substrate with the solvent. The D-inc. on C11 of **5j** was 66% according to the signal intensity at δ 4.34.

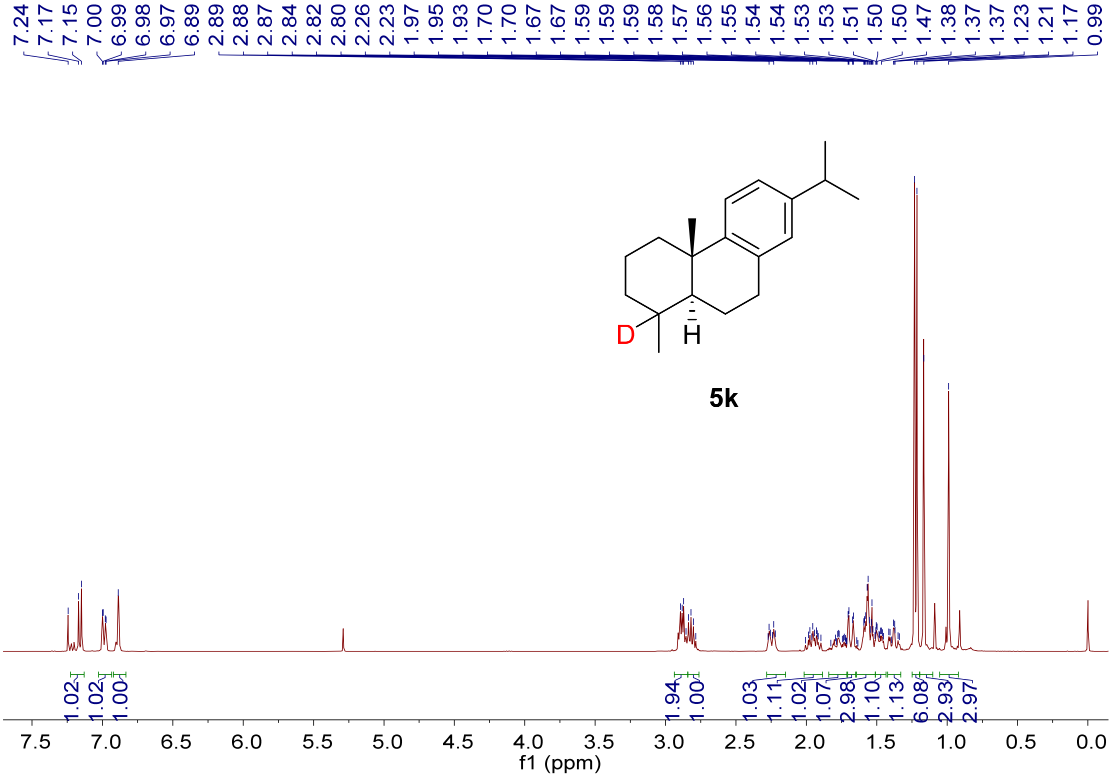

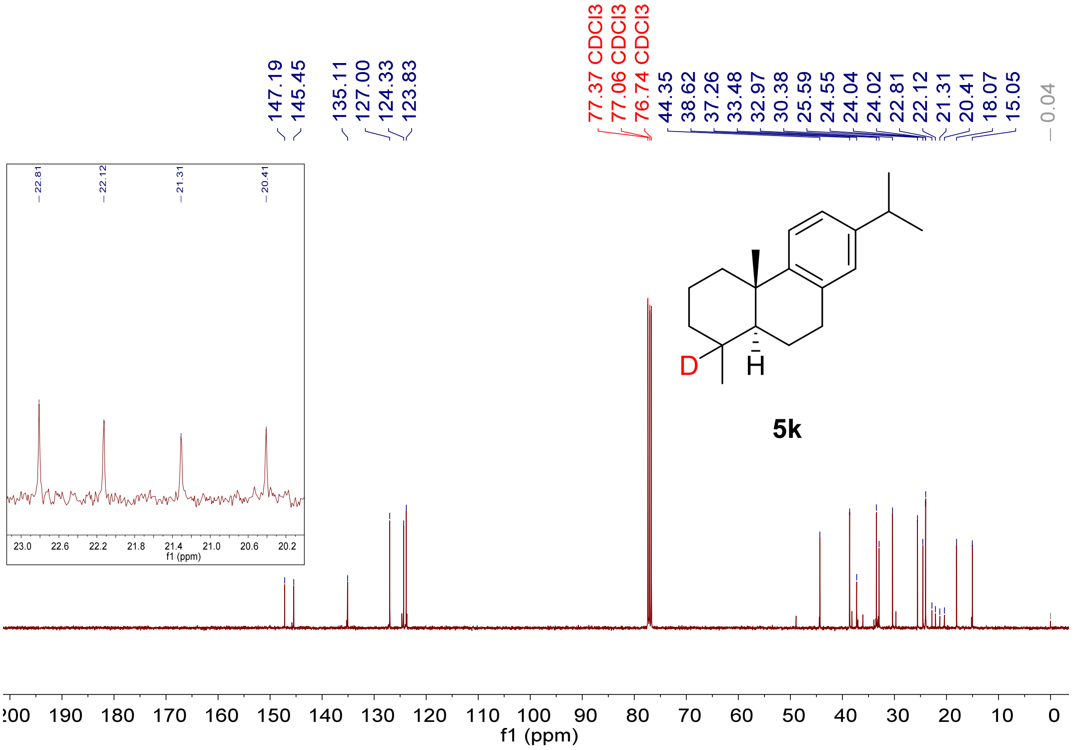

Supplementary Fig. 88. NMR spectra of (4aS,10aS)-7-isopropyl-1,4a-dimethyl-1,2,3,4,4a,9,10,10a-octahydrophenanthrene-1-d (5k). The splitting of C-D coupling in ^{13}C-NMR was shown with an enlarged view.

Supplementary Tables

Supplementary Table 1 List of primers.

Mutant	Sequence
A128L	GTTAAAATTCCGCTGGCAATTACCCG
A128F	GTTAAAATTCCGTTTGCAATTACCCG
A171L	TAGCAGCGCAACCAATCTGACCCTGTATCATCG
A171F	TAGCAGCG CAACCAATTTTACCCTGTATCATCG
T172A	GCAACCAATGCAGCGCTGTATCATCG
T172L	GCAACCAATGCACTGCTGTATCATCG
T172F	GCAACCAATGCATTTCTGTATCATCG
I398A	AAATATGATGGTGCCGCCATCAGCGATCAC
I398L	AAATATGATGGTCTGGCCATCAGCGATCAC
I398F	AAATATGATGGTTTTGCCATCAGCGATCAC
I398R	AAATATGATGGTCGCGCCATCAGCGATCAC
A399L	TATGATGGTATTCTGATCAGCGATCACATT
A399F	TATGATGGTATTTTTATCAGCGATCACATT
G431A	CTGACCAGTACCGCGTGTGATCGTGG
G431L	CTGACCAGTACCCTGTGTGATCGTGG
G431F	CTGACCAGTACCTTTTGTGATCGTG
G462A	CTGGATCCGGATGCGGTTAGCACCTATG
G462L	CTGGATCCGGATCTGGTTAGCACCTATG
G462F	CTGGATCCGGATTTTGTTAGCACCTATG
Y466A	GATGGTGTTAGCACCGCGGTTCGTTTTGCAAAA
Y466L	GATGGTGTTAGCACCCTGGTTCGTTTTGCAAAA
Y466F	GATGGTGTTAGCACCTTTGTTCGTTTTGCAAAA
Q486A	GTATTACCATGGCGCTGATTGCATG
Q486L	GTATTACCATGCTGCTGATTGCATG
Q486F	GTATTACCATGTTTCTGATTGCATG
silent reverse primer	GATGCCGGGAGCAGACAAGCCCGTCAGGGCGC

Supplementary Table 2 The ratio of M-1 peak to M peak in HRMS of various hydrocarbons with low boiling points.

**Substrate**	**HRMS result**	
	**Ratio $_{M}$ (%)**	**Ratio $_{M-1}$ (%)**
Ethane	100	66
Propane	100	80
Pentane	100	12
Hexane	100	2
Heptane	100	<1
Octane	100	<1
Decane	100	<1
Undecane	100	<1

Supplementary Table 3 Evaluation of the difference of D-incorporation determined by NMR and HRMS.

Substrate	D-incorporation (%)	
	Determined by HRMS	Determined by NMR
**1a**	91±1	93±2
**1b**	94±2	95±2
**1c**	95±1	96±1

Data are presented as mean value ± SD (standard deviations) of three replicates.

Supplementary Table 4 Protein sequence of *Cv*FAP.

**Protein sequence of *Cv*FAP**
MGSSHHHHHH SSGLVPRGSH MASMTGGQQM GRGSEFMASI TSRASARASC SQANTRAGRV ALSGGALLRP ARPARSFVPA RKQQQGAVRR GGALSARASA VEDIRKVLSD SSSPVAGQKY DYILVGGGTA ACVLANRLSA DGSKRVLVLE AGPDNTSRDV KIPAAITRLF RSPLDWNLFS ELQEQLAERQ IYMARGRLLG GSSATNATLY HRGAAGDYDA WGVEGWSSED VLSWFVQAET NADFGPGAYH GSGGPMRVEN PRYTNKQLHT AFFKAAEEVG LTPNSDFNDW SHDHAGYGTF QVMQDKGTRA DMYRQYLKPV LGRRNLQVLT GAAVTKVNID QAAGKAQALG VEFSTDGPTG ERLSAELAPG GEVIMCAGAV HTPFLLKHSG VGPSAELKEF GIPVVSNLAG VGQNLQDQPA CLTAAPVKEK YDGIAISDHI YNEKGQIRKR AIASYLLGGR GGLTSTGCDR GAFVRTAGQA LPDLQVRFVP GMALDPDGVS TYVRFAKFQS QGLKWPSGIT MQLIACRPQS TGSVGLKSAD PFAPPKLSPG YLTDKDGADL ATLRKGIHWA RDVARSSALS EYLDGELFPG SGVVSDDQID EYIRRSIHSS NAITGTCKMG NAGDSSSVVD NQLRVHGVEG LRVVDASVVP KIPGGQTGAP VVMIAERAAA LLTGKATIGA SAAAPATVAA

Supplementary Table 5 DNA sequence of *Cv*FAP.

	**DNA sequence of *Cv*FAP**
1	ATGGGCAGCA GCCATCATCA TCATCATCAC AGCAGCGGCC TGGTGCCGCG CGGCAGCCAT
61	ATGGCTAGCA TGACTGGTGG ACAGCAAATG GGTCGCGGAT CCGAATTCAT GGCAAGCATT
121	ACCAGCCGTG CAAGCGCACG TGCAAGCTGT AGCCAGGCAA ATACCCGTGC AGGTCGTGTT
181	GCACTGAGCG GTGGTGCACT GCTGCGTCCG GCACGTCCTG CACGTAGCTT TGTTCCGGCA
241	CGTAAACAGC AGCAGGGTGC AGTTCGTCGT GGTGGTGCCC TGAGCGCACG TGCCAGCGCA
301	GTTGAAGATA TTCGTAAAGT TCTGAGCGAT AGCAGCAGTC CGGTTGCAGG TCAGAAATAT
361	GATTATATTC TGGTTGGTGG TGGCACCGCA GCATGTGTTC TGGCAAATCG TCTGAGCGCA
421	GATGGTAGCA AACGTGTTCT GGTTCTGGAA GCAGGTCCGG ATAATACCAG CCGTGATGTT
481	AAAATTCCGG CAGCAATTAC CCGTCTGTTT CGTAGTCCGC TGGATTGGAA CCTGTTTAGC
541	GAACTGCAAG AACAGCTGGC AGAACGTCAG ATTTATATGG CACGTGGTCG TCTGCTGGGT
601	GGTAGCAGCG CAACCAATGC AACCCTGTAT CATCGTGGTG CAGCCGGTGA TTATGATGCA
661	TGGGGTGTTG AAGGTTGGAG CAGCGAAGAT GTTCTGAGCT GGTTTGTTCA GGCAGAAACC
721	AATGCAGATT TTGGTCCGGG TGCATATCAT GGTAGCGGTG GTCCGATGCG TGTTGAAAAT
781	CCGCGTTATA CCAATAAACA GCTGCATACC GCATTTTTCA AAGCAGCAGA AGAAGTTGGT
841	CTGACCCCGA ATAGCGATTT TAATGATTGG AGCCATGATC ATGCAGGTTA TGGCACCTTT
901	CAGGTTATGC AGGATAAAGG CACCCGTGCA GATATGTATC GTCAGTATCT GAAACCGGTT
961	CTGGGTCGTC GTAATCTGCA GGTTCTGACC GGTGCAGCAG TTACCAAAGT TAATATTGAT
1021	CAGGCAGCAG GTAAAGCACA GGCACTGGGT GTTGAATTTT CAACCGATGG TCCGACCGGT
1081	GAACGTCTGA GTGCAGAACT GGCACCGGGT GGTGAAGTTA TTATGTGTGC CGGTGCAGTT
1141	CATACCCCGT TTCTGCTGAA ACATAGCGGT GTTGGTCCGA GCGCAGAACT GAAAGAATTT
1201	GGTATTCCGG TTGTTAGCAA TCTGGCAGGC GTTGGTCAGA ATCTGCAGGA TCAGCCTGCA
1261	TGTCTGACCG CAGCACCGGT TAAAGAAAAA TATGATGGTA TTGCCATCAG CGATCACATT
1321	TATAACGAAA AAGGTCAGAT TCGCAAACGT GCAATTGCAA GCTATCTGCT GGGAGGTCGT
1381	GGTGGTCTGA CCAGTACCGG TTGTGATCGT GGTGCATTTG TTCGTACCGC AGGTCAGGCA
1441	CTGCCGGATC TGCAGGTACG TTTTGTTCCG GGTATGGCAC TGGATCCGGA TGGTGTTAGC
1501	ACCTATGTTC GTTTTGCAAA ATTTCAGAGC CAGGGTCTGA AATGGCCGAG CGGTATTACC
1561	ATGCAGCTGA TTGCATGTCG TCCGCAGAGC ACCGGTAGCG TTGGTCTGAA AAGCGCAGAT
1621	CCGTTTGCAC CGCCTAAACT GAGTCCGGGT TATCTGACCG ATAAAGATGG TGCAGATCTG
1681	GCAACCCTGC GTAAAGGTAT TCATTGGGCA CGTGATGTTG CACGTAGCAG CGCACTGAGC
1741	GAATATCTGG ATGGTGAACT GTTTCCGGGT AGCGGTGTTG TTAGTGATGA TCAGATTGAT
1801	GAATATATCC GTCGCAGCAT TCATAGCAGC AATGCAATTA CCGGCACCTG TAAAATGGGT
1861	AATGCCGGTG ATAGCAGCAG CGTTGTTGAT AATCAGCTGC GTGTTCATGG TGTGGAAGGT
1921	CTGCGTGTTG TTGATGCAAG CGTTGTTCCG AAAATTCCGG GTGGTCAGAC AGGTGCACCG
1981	GTTGTTATGA TTGCAGAACG TGCAGCAGCA CTGCTGACCG GTAAAGCAAC CATTGGTGCA
2041	AGCGCAGCAG CACCGGCAAC CGTTGCAGCA TGA

Supplementary Table 6 Characterization data of prepared substrates and products.

**Compounds**	**Characterization data**
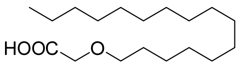<br>**2-(Hexadecyloxy)acetic acid (1I)**	^{1}H NMR (400 MHz, $CDCl_3$) δ 4.09 (s, 2H), 3.57 (t, *J* = 6.7 Hz, 2H), 1.67 – 1.60 (m, 2H), 1.28 (s, 26H), 0.88 (t, 3H).<br>^{13}C NMR (100 MHz, $CDCl_3$) δ 172.64, 72.23, 67.74, 31.95, 29.71, 29.67, 29.60, 29.56, 29.42, 29.38, 25.91, 22.71, 14.14.<br>HRMS (EI-TOF) m/z: [M]+ Calcd for $C_{18}H_{36}O_3$ 300.2665; Found 300.2669.
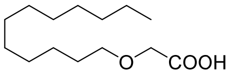<br>**2-(Dodecyloxy)acetic acid (2K)**	^{1}H NMR (400 MHz, $CDCl_3$) δ 4.12 (s, 2H), 3.55 (t, *J* = 6.7 Hz, 2H), 1.72 – 1.57 (m, 2H), 1.26 (m, 18H), 0.88 (t, *J* = 6.7 Hz, 3H).<br>^{13}C NMR (100 MHz, $CDCl_3$) δ 174.78, 72.20, 67.73, 31.93, 29.66, 29.64, 29.59, 29.56, 29.43, 29.40, 29.36, 25.91, 22.70, 14.12.<br>HRMS (EI-TOF) m/z: [M]+ Calcd for $C_{14}H_{28}O_3$ 244.2038; Found 244.2039.
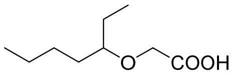<br>**2-(Heptan-3-yloxy)acetic acid (4A)**	^{1}H NMR (400 MHz, $CDCl_3$) δ 4.04 (s, 2H), 3.29 (m, 1H), 1.55 – 1.36 (m, 4H), 1.35 – 1.16 (m, 4H), 0.89 – 0.80 (m, 6H).<br>^{13}C NMR (100 MHz, $CDCl_3$) δ 173.68, 82.51, 65.85, 32.70, 27.41, 26.02, 22.80, 14.02, 9.36.<br>HRMS (EI-TOF) m/z: [M]+ Calcd for $C_9H_{18}O_3$ 174.1256; Found 174.1257.
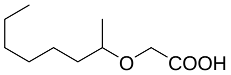<br>**2-(Octan-2-yloxy)acetic acid (4B)**	^{1}H NMR (400 MHz, $CDCl_3$) δ 4.25 – 4.03 (m, 2H), 3.55 (m, 1H), 1.68 – 1.39 (m, 2H), 1.39 – 1.21 (m, 8H), 1.19 (d, *J* = 6.1 Hz, 3H), 0.94 – 0.83 (m, 3H).<br>^{13}C NMR (100 MHz, $CDCl_3$) δ 174.27, 77.29, 65.48, 36.21, 31.78, 29.32, 25.36, 22.61, 19.32, 14.09.<br>HRMS (EI-TOF) m/z: [M]+ Calcd for $C_{10}H_{20}O_3$ 188.1412; Found 188.1411.
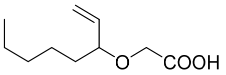<br>**2-(Oct-1-en-3-yloxy)acetic acid (4C)**	^{1}H NMR (400 MHz, $CDCl_3$) δ 5.65 (ddd, *J* = 17.1, 10.2, 8.1 Hz, 1H), 5.26 (dd, *J* = 10.3, 1.6 Hz, 1H), 5.27 – 5.17 (m, 1H), 4.14 (q, *J* = 16.9 Hz, 2H), 3.76 (m, 1H), 1.77 – 1.48 (m, 2H), 1.41 – 1.29 (m, 6H), 0.92 – 0.85 (m, 3H).<br>^{13}C NMR (100 MHz, $CDCl_3$) δ 176.07, 137.63, 118.52, 82.56, 64.79, 35.00, 31.65, 24.80, 22.49, 13.93.<br>HRMS (EI-TOF) m/z: [M]+ Calcd for $C_{10}H_{18}O_3$ 186.1256; Found 186.1253.
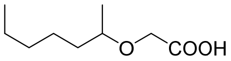<br>**2-(Heptan-2-yloxy)acetic acid (4D)**	^{1}H NMR (400 MHz, $CDCl_3$) δ 3.97 (m, 2H), 3.37 (m, 1H), 1.45 – 1.15 (m, 2H), 1.15 – 1.09 (m, 6H), 1.02 (d, *J* = 6.2 Hz, 3H), 0.72 (t, *J* = 6.9 Hz, 3H).<br>^{13}C NMR (100 MHz, $CDCl_3$) δ 174.25, 77.29, 65.48, 36.17, 31.83, 25.06, 22.59, 1.32, 14.02.<br>HRMS (EI-TOF) m/z: [M]+ Calcd for $C_9H_{18}O_3$ 174.1256; Found 174.1259.
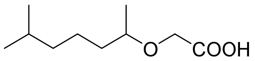<br>**2-((6-Methylheptan-2-yl)oxy)acetic acid (4E)**	^{1}H NMR (400 MHz, $CDCl_3$) δ 10.65 (s, 1H), 4.25 – 4.04 (m, 2H), 3.54 (m, 1H), 1.57 (ddt, *J* = 26.3, 13.3, 6.6 Hz, 2H), 1.48 – 1.25 (m, 3H), 1.18 (m, 5H), 0.87 (d, *J* = 6.7 Hz, 6H).<br>^{13}C NMR (100 MHz, $CDCl_3$) δ 175.73, 76.96, 65.28, 38.89, 36.33, 27.83, 23.07, 22.51, 22.47, 19.12.<br>HRMS (EI-TOF) m/z: [M]+ Calcd for $C_{10}H_{20}O_3$ 188.1412; Found 188.1409.
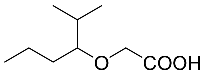	^{1}H NMR (400 MHz, $CDCl_3$) δ 10.41 (s, 1H), 4.07 (d, *J* = 2.4 Hz, 2H), 3.08 (m, 1H), 1.89 – 1.72 (m, 1H), 1.51 – 1.16 (m, 4H), 0.84 (m, 9H).

**2-((2-Methylhexan-3-yl)oxy)acetic acid (4F)**	^{13}C NMR (100 MHz, $CDCl_3$) δ 175.43, 86.21, 66.76, 32.21, 30.37, 18.82, 18.12, 17.70, 14.16.<br>HRMS (EI-TOF) m/z: [M]+ Calcd for $C_9H_{18}O_3$ 174.1256; Found 174.1255.
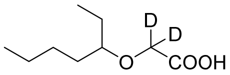<br>**2-(Heptan-3-yloxy)acetic-2,2-d2 acid (4G)**	^{1}H NMR (400 MHz, $CDCl_3$) δ 4.10 (m, 0.25H), 3.36 (m, 1H), 1.65 – 1.43 (m, 4H), 1.42 – 1.26 (m, 4H), 0.91 (m, 6H).<br>^{13}C NMR (100 MHz, $CDCl_3$) δ 174.00, 82.39, 65.53 (t, C-D), 32.70, 27.40, 26.02, 22.81, 14.04, 9.37.<br>HRMS (EI-TOF) m/z: [M]+ Calcd for $C_9H_{16}D_2O_3$ 176.1382; Found 176.1385.
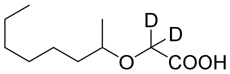<br>**2-(Octan-2-yloxy)acetic-2,2-d2 acid (4H)**	^{1}H NMR (400 MHz, $CDCl_3$) δ 6.91 (s, 1H), 4.00 (m, 0.33H), 3.50 (p, 1H), 1.45 – 1.27 (m, 2H), 1.27 – 1.16 (m, 8H), 1.13 (d, J = 5.1 Hz, 3H), 0.89 – 0.76 (m, 3H).<br>^{13}C NMR (100 MHz, $CDCl_3$) δ 173.03, 76.16, 63.71, 35.23, 30.77, 28.30, 24.34, 21.59, 18.31, 13.06.<br>HRMS (EI-TOF) m/z: [M]+ Calcd for $C_{10}H_{18}D_2O_3$ 190.1538; Found 190.1540.
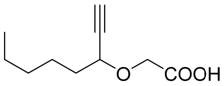<br>**2-(Oct-1-yn-3-yloxy)acetic acid (4I)**	^{1}H NMR (400 MHz, $CDCl_3$) δ 9.76 (s, 1), 4.50 – 4.01 (m, 3H), 2.43 (d, *J* = 2.1 Hz, 1H), 1.84 – 1.59 (m, 2H), 1.49 – 1.34 (m, 2H), 1.33 – 1.15 (m, 4H), 0.88 – 0.72 (m, 3H).<br>^{13}C NMR (100 MHz, $CDCl_3$) δ 175.75, 81.40, 75.11, 70.03, 64.91, 35.24, 31.38, 24.66, 22.48, 13.96.<br>HRMS (EI-TOF) m/z: [M]+ Calcd for $C_{10}H_{16}O_3$ 184.1099; Found 184.1101.
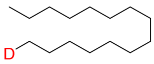<br>**Pentadecane-1-d (1a)**	^{1}H NMR (400 MHz, $CDCl_3$) δ 1.26 (m, 26H), 0.88 (m, 5H).<br>93% D-inc. calculated from ^{1}H NMR. 91% D-inc. calculated from HRMS.<br>^{13}C NMR (100 MHz, $CDCl_3$) δ 31.95, 31.92, 29.73, 29.68, 29.39, 22.72, 22.63, 14.14-13.65(-CH_2–D, -CH_3).<br>HRMS (EI-TOF) m/z: [M]+ Calcd for $C_{15}H_{31}D$ 213.2567; Found 213.2593.
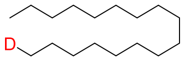<br>**Heptadecane-1-d (1b)**	^{1}H NMR (400 MHz, $CDCl_3$) δ 1.26 (m, 30H), 0.92 – 0.86 (m, 5H).<br>95% D-inc. calculated from ^{1}H NMR. 94% D-inc. calculated from HRMS.<br>^{13}C NMR (100 MHz, $CDCl_3$) δ 31.95, 31.92, 29.72, 29.68, 29.39, 22.72, 22.68, 22.63, 14.14-13.65(-CH_2–D, -CH_3).<br>HRMS (EI-TOF) m/z: [M]+ Calcd for $C_{17}H_{35}D$ 241.2880; Found 241.2880.
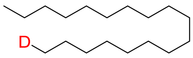<br>**Octadecane-1-d (1c)**	^{1}H NMR (400 MHz, $CDCl_3$) δ 1.29 – 1.26 (m, 32H), 0.90-0.86 (m, 5H).<br>96% D-inc. calculated from ^{1}H NMR. 95% D-inc. calculated from HRMS.<br>^{13}C NMR (100 MHz, CDCl3) δ 31.96, 31.93, 29.74, 29.70, 29.40, 22.72, 22.63, 14.11-13.62 (-CH_2–D, -CH_3).<br>HRMS (EI-TOF) m/z: [M]+ Calcd for $C_{18}H_{37}D$ 255.3036; Found 255.3036.
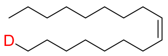<br>**(Z)-Heptadec-8-ene-1-d (1d)**	^{1}H NMR (400 MHz, $CDCl_3$) δ 5.28 (m, 2H), 1.99 – 1.90 (m, 4H), 1.20 (m, 22H), 0.81 (m, 5H).<br>91% D-inc. calculated from ^{1}H NMR.<br>^{13}C NMR (100MHz, $CDCl_3$) δ 128.90, 30.91, 30.86, 28.78, 28.53, 28.32, 28.28, 28.24, 26.20, 21.68, 21.59, 13.10-12.61 (-CH_2–D, -CH_3).<br>HRMS (EI-TOF) m/z: [M]+ Calcd for $C_{17}H_{33}D$ 239.2723; Found 239.2719.
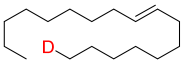<br>**(E)-Heptadec-8-ene-1-d (1e)**	^{1}H NMR (400 MHz, $CDCl_3$) δ 5.38 (m, 2H), 1.96 (q, *J* = 6.7 Hz, 4H), 1.26 (s, 22H), 0.90 – 0.86 (m, 5H).<br>^{13}C NMR (100 MHz, $CDCl_3$) δ 130.38, 32.63, 31.92, 31.87, 31.61, 29.68, 29.52, 29.34, 29.23, 29.19, 29.15, 22.70, 22.68, 22.61, 14.14-13.64 (-CH_2–D, -CH_3).

	HRMS (EI-TOF) m/z: [M]+ Calcd for $C_{17}H_{33}D$ 239.2723; Found 239.2729.<br>96% D-inc. calculated from HRMS.
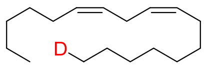<br>**(6Z,9Z)-Heptadeca-6,9-diene-17-d (1f)**	^{1}H NMR (400 MHz, $CDCl_3$) δ 5.35 (m, 4H), 2.78 (t, *J* = 6.5 Hz, 2H), 2.05 (q, *J* = 6.9 Hz, 4H), 1.43 – 1.18 (m, 16H), 0.88 (m, 5H).<br>^{13}C NMR (100 MHz, $CDCl_3$) δ 130.21, 127.97, 31.87, 31.56, 29.71, 29.38, 29.31, 29.25, 27.26, 27.22, 25.64, 22.60, 14.13-13.63 (-$\underline{C}H_2$–D, -$\underline{C}H_3$).<br>HRMS (EI-TOF) m/z: [M]+ Calcd for $C_{17}H_{31}D$ 237.2567; Found 237.2567.<br>93% D-inc. calculated from HRMS.
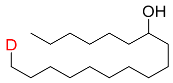<br>**Heptadecan-17-d-7-ol (1g)**	^{1}H NMR (400 MHz, $CDCl_3$) δ 3.52 (m, 1H), 1.36 (m, 8H), 1.20 (m, 20H), 0.82 (m, 5H).<br>87% D-inc. calculated from ^{1}H NMR.<br>^{13}C NMR (100 MHz, $CDCl_3$) δ 72.05, 37.51, 31.89, 29.73, 29.64, 29.39, 29.35, 25.67, 25.63, 22.62, 14.09-13.63 (-$\underline{C}H_2$–D, -$\underline{C}H_3$).<br>HRMS (EI-TOF) m/z: [M]+ Calcd for $C_{17}H_{35}DO$ 257.2829; Found 257.2514.
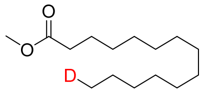<br>**Methyl tetradecanoate-14-d (1h)**	^{1}H NMR (400 MHz, $CDCl_3$) δ 3.67 (s, 3H), 2.30 (t, *J* = 7.6 Hz, 2H), 1.62 (m, 2H), 1.25 (m, 20H), 0.90 – 0.83 (m, 2H).<br>^{13}C NMR (100 MHz, $CDCl_3$) δ 174.41, 51.47, 34.14, 31.91, 29.69, 29.66, 29.62, 29.47, 29.38, 29.28, 29.17, 24.98, 22.62, 14.03-13.65 (-$\underline{C}H_2$–D).<br>HRMS (EI-TOF) m/z: [M]+ Calcd for $C_{15}H_{29}DO_2$ 243.2309; Found 243.2311.<br>96% D-inc. calculated from HRMS.
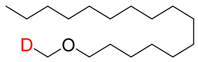<br>**1-(Methoxy-d)hexadecane (1i)**	^{1}H NMR (400 MHz, $CDCl_3$) δ 3.36 (t, *J* = 6.6 Hz, 2H), 3.34 – 3.30 (m, 2H), 1.60 – 1.50 (m, 2H), 1.25 (m, 26H), 0.88 (t, *J* = 6.8 Hz, 3H).<br>96% D-inc. calculated from ^{1}H NMR.<br>^{13}C NMR (100 MHz, $CDCl_3$) δ 72.97, 58.55-58.04 (-O$\underline{C}H_2$–D), 31.94, 29.71, 29.67, 29.62, 29.53, 29.38, 26.16, 22.71, 14.13.<br>HRMS (EI-TOF) m/z: [M]+ Calcd for $C_{17}H_{35}DO$ 257.2829; Found 257.2849.
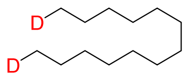<br>**Tridecane-1,13-d2 (1j)**	^{1}H NMR (400 MHz, $CDCl_3$) δ 1.26 (m, 22H), 0.90 – 0.82 (m, 4H).<br>^{13}C NMR (100 MHz, $CDCl_3$) δ 31.92, 29.73, 29.69, 29.40, 22.63, 14.13-13.64 (-$\underline{C}H_2$–D).<br>HRMS (EI-TOF) m/z: [M]+ Calcd for $C_{13}H_{27}D$ 185.2254; Found 185.2250.<br>HRMS (EI-TOF) m/z: [M]+ Calcd for $C_{13}H_{26}D_2$ 186.2317; Found 186.2319.<br>91% 2D-inc. calculated from HRMS.
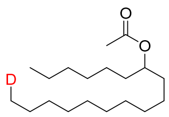<br>**Heptadecan-7-yl-17-d acetate (1k)**	^{1}H NMR (400 MHz, $CDCl_3$) δ 4.79 (m, 1H), 1.97 (s, 3H), 1.43 (m, 4H), 1.19 (s, 24H), 0.81 (t, *J* = 6.7 Hz, 5H).<br>83% D-inc. calculated from ^{1}H NMR.<br>^{13}C NMR (100 MHz, $CDCl_3$) δ 170.97, 74.48, 34.13, 31.89, 31.76, 29.61, 29.59, 29.55, 29.34, 29.22, 25.32, 25.28, 22.59, 21.30, 14.06-13.62 (-$\underline{C}H_2$–D, -$\underline{C}H_3$).<br>HRMS (EI-TOF) m/z: [M]+ Calcd for $C_{19}H_{37}DO_2$ 299.2935; Found 299.2944.
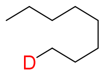<br>**Octane-1-d (2a)**	HRMS (EI-TOF) m/z: [M]+ Calcd for $C_8H_{17}D$ 115.1471; Found 115.1473.<br>98% D-inc. calculated from HRMS.
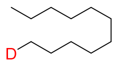	^{1}H NMR (400 MHz, $CDCl_3$) δ 1.26 (m, 18H), 0.88 (m, 5H).<br>^{13}C NMR (100 MHz, $CDCl_3$) δ 31.95, 31.92, 29.73, 29.68, 29.39, 22.71, 22.63, 14.12-13.64 (-$\underline{C}H_2$–D, -$\underline{C}H_3$).

**Undecane-1-d (2b)**	HRMS (EI-TOF) m/z: [M]+ Calcd for $C_{11}H_{23}D$ 157.1941; Found 157.1944.<br>95% D-inc. calculated from HRMS.
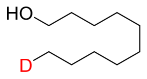<br>**Decan-10-d-1-ol (2c)**	^{1}H NMR (400 MHz, $CDCl_3$) δ 3.62 (t, J = 6.7 Hz, 2H), 1.96 (m, 1H), 1.55 (m, 2H), 1.27 (m, 14H), 0.92 – 0.81 (m, 2H).<br>90% D-inc. calculated from ^{1}H NMR.<br>^{13}C NMR (100 MHz, $CDCl_3$) δ 62.94, 32.77, 31.90, 31.87, 29.63, 29.57, 29.46, 29.34, 25.76, 22.67, 22.59, 14.08-13.59 (-$\underline{C}H_2$–D).<br>HRMS (EI-TOF) m/z: [M]+ Calcd for $C_{10}H_{21}DO$ 159.1733; Found 159.1724.
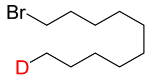<br>**1-Bromodecane-10-d (2d)**	^{1}H NMR (400 MHz, $CDCl_3$) δ 3.40 (t, *J* = 6.9 Hz, 2H), 1.85 (m, 2H), 1.42 (m, 2H), 1.27 (s, 12H), 0.88 (t, *J* = 6.8 Hz, 2H).<br>^{13}C NMR (100 MHz, $CDCl_3$) δ 34.01, 32.87, 31.90, 31.87, 29.53, 29.47, 29.32, 28.80, 28.21, 22.69, 22.61, 14.12-13.63 (-$\underline{C}H_2$–D).<br>HRMS (EI-TOF) m/z: [M]+ Calcd for $C_{10}H_{20}DBr$ 221.0889; Found 221.0898.<br>99% D-inc. calculated from HRMS.
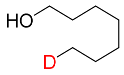<br>**Heptan-7-d-1-ol (2e)**	^{1}H NMR (400 MHz, $CDCl_3$) δ 4.61 (s, 1H), 3.67 (t, *J* = 6.9 Hz, 2H), 1.55 (p, *J* = 6.8 Hz, 2H), 1.23 (m, 8H), 0.87 – 0.75 (m, 2H).<br>87% D-inc. calculated from ^{1}H NMR.<br>^{13}C NMR (100 MHz, $CDCl_3$) δ 64.00, 32.01, 31.77, 31.74, 29.02, 25.52, 22.57, 22.49, 14.02-13.53 (-$\underline{C}H_2$–D).<br>HRMS (EI-TOF) m/z: [M]+ Calcd for $C_7H_{15}DO$ 117.1264; Found 117.1264.
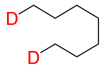<br>**Heptane-1,7-d2 (2f)**	HRMS (EI-TOF) m/z: [M]+ Calcd for $C_7H_{15}D$ 101.1315; Found 101.1301.<br>HRMS (EI-TOF) m/z: [M]+ Calcd for $C_7H_{14}D_2$ 102.1378; Found 102.1384.<br>97% 2D-inc. calculated from HRMS.
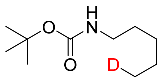<br>**Tert-butyl (pentyl-5-d)carbamate (2g)**	^{1}H NMR (400 MHz, $CDCl_3$) δ 4.68 (s, 1H), 3.11 (m, 2H), 1.48 (m, 2H), 1.44 (s, 9H), 1.30 (m, 4H), 0.95 – 0.84 (m, 2H).<br>82% D-inc. calculated from ^{1}H NMR.<br>^{13}C NMR (100 MHz, $CDCl_3$) δ 155.99, 78.84, 40.55, 29.72, 28.91, 28.38, 22.32, 22.23, 13.95-13.46 (-$\underline{C}H_2$–D).<br>HRMS (EI-TOF) m/z: [M]+ Calcd for $C_{10}H_{20}DNO_2$ 188.1635; Found 188.1643.
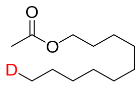<br>**Decyl-10-d acetate (2h)**	^{1}H NMR (400 MHz, $CDCl_3$) δ 4.1 (t, *J* = 6.8 Hz, 2H), 2.0 (s, 3H), 1.6 (t, *J* = 7.1 Hz, 2H), 1.3 (d, *J* = 3.5 Hz, 14H), 0.9 – 0.8 (m, 2H).<br>85% D-inc. calculated from ^{1}H NMR.<br>^{13}C NMR (100 MHz, $CDCl_3$) δ 171.30, 64.69, 31.90, 31.87, 29.54, 29.32, 29.27, 28.61, 25.92, 22.69, 22.60, 21.04, 14.12-13.63 (-$\underline{C}H_2$–D).<br>HRMS (EI-TOF) m/z: [M]+ Calcd for $C_{12}H_{23}DO_2$ 201.1839; Found 201.1841.
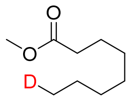<br>**Methyl octanoate-8-d (2i)**	^{1}H NMR (400 MHz, $CDCl_3$) δ 3.60 (s, 3H), 2.23 (t, *J* = 15.1 Hz, 2H), 1.59 – 1.51 (m, 2H), 1.25 – 1.14 (m, 8H), 0.85 – 0.76 (m, 2H).<br>^{13}C NMR (100 MHz, $CDCl_3$) δ 173.38, 50.43, 33.11, 30.64, 30.61, 28.10, 27.91, 23.95, 21.49, 13.10-12.55 (-$\underline{C}H_2$–D).<br>HRMS (EI-TOF) m/z: [M]+ Calcd for $C_9H_{17}DO_2$ 159.1370; Found 159.1375.<br>93% D-inc. calculated from HRMS.
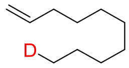	^{1}H NMR (400 MHz, $CDCl_3$) δ 5.82 (m, 1H), 5.10 – 4.87 (m, 2H), 2.12 – 1.97 (m, 2H), 1.37 (m, 2H), 1.27 (m, 10H), 0.88 (m, 2H).

**Dec-1-ene-10-d (2j)**	^{13}C NMR (100 MHz, $CDCl_3$) δ 139.31, 114.09, 33.84, 31.88, 29.49, 29.31, 29.18, 28.97, 22.60, 14.12-13.63 (-CH_2–D).<br>HRMS (EI-TOF) m/z: [M]+ Calcd for $C_{10}H_{19}D$ 141.1628; Found 141.1617.<br>92% D-inc. calculated from HRMS.
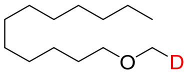<br>**1-(Methoxy-d)dodecane (2k)**	^1H NMR (400 MHz, $CDCl_3$) δ 3.36 (m, 2H), 3.33 – 3.28 (m, 2H), 1.56 (m, 2H), 1.29 (s, 18H), 0.88 (t, J = 6.7 Hz, 3H).<br>95% D-inc. calculated from ^1H NMR.<br>^{13}C NMR (100 MHz, $CDCl_3$) δ 72.97, 58.47-58.04 (-OCH_2–D), 31.94, 29.65, 29.62, 29.53, 29.36, 26.16, 22.70, 14.13.<br>HRMS (EI-TOF) m/z: [M]+ Calcd for $C_{13}H_{27}DO$ 201.2203; Found 201.2219.
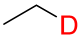<br>**Ethane-d (3a)**	HRMS (EI-TOF) m/z: [M]+ Calcd for C_2H_5D 31.0532; Found 31.0558.<br>98% D-inc. calculated from HRMS.
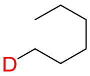<br>**Hexane-1-d (3b)**	HRMS (EI-TOF) m/z: [M]+ Calcd for $C_6H_{13}D$ 87.1158; Found 87.1156.<br>97% D-inc. calculated from HRMS.
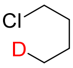<br>**1-Chlorobutane-4-d (3c)**	HRMS (EI-TOF) m/z: [M]+ Calcd for C_4H_8DCl 93.0456; Found 93.0453.<br>83% D-inc. calculated from HRMS.
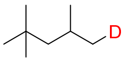<br>**2, 2, 4-Trimethylpentane-5-d (3d)**	HRMS (EI-TOF) m/z: [M]+ Calcd for $C_8H_{17}D$ 115.1471; Found 115.1475.<br>99% D-inc. calculated from HRMS.
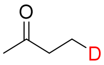<br>**Butan-2-one-4-d (3e)**	HRMS (EI-TOF) m/z: [M]+ Calcd for C_4H_7DO 73.0638; Found 73.0642.<br>92% D-inc. calculated from HRMS.
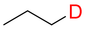<br>**Propane-1-d (3f)**	HRMS (EI-TOF) m/z: [M]+ Calcd for C_3H_7D 45.0689; Found 45.0692.<br>88% D-inc. calculated from HRMS.
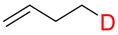<br>**But-1-ene-4-d (3g)**	HRMS (EI-TOF) m/z: [M]+ Calcd for C_4H_7D 57.0689; Found 57.0689.<br>99% D-inc. calculated from HRMS.
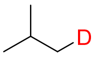<br>**Isobutane-1-d (3h)**	HRMS (EI-TOF) m/z: [M]+ Calcd for C_4H_9D 59.0845; Found 59.0847.<br>99% D-inc. calculated from HRMS.
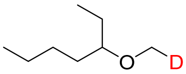<br>**3-(Methoxy-d)heptane (4a)**	^1H NMR (400 MHz, $CDCl_3$) δ 3.35 – 3.28 (m, 2H), 3.08 (m, 1H), 1.56 – 1.40 (m, 4H), 1.40 – 1.26 (m, 4H), 0.90 (q, J = 7.4, 7.0 Hz, 6H).<br>99% D-inc. calculated from ^1H NMR.<br>^{13}C NMR (100 MHz, $CDCl_3$) δ 82.07, 57.41-56.33 (-OCH_2–D), 53.45 (DCM), 32.70, 27.55, 25.82, 22.94, 14.13, 9.37.<br>HRMS (EI-TOF) m/z: [M]+ Calcd for $C_8H_{17}DO$ 131.1420; Found 131.1411.
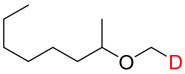<br>**2-(Methoxy-d)octane (4b)**	^1H NMR (400 MHz, $CDCl_3$) δ 3.34 – 3.20 (m, 3H), 1.54 – 1.45 (m, 2H), 1.28 (m, 8H), 1.12 (d, *J* = 6.1 Hz, 3H), 0.92 – 0.84 (t, 3H).<br>99% D-inc. calculated from ^1H NMR.<br>^{13}C NMR (100 MHz, $CDCl_3$) δ 76.87, 55.86-55.44 (-OCH_2–D), 53.44 (DCM), 36.36, 31.88, 29.46, 25.44, 22.64, 19.04, 14.11.<br>HRMS (EI-TOF) m/z: [M]+ Calcd for $C_9H_{19}DO$ 145.1577; Found 145.1581.

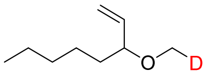<br>**3-(Methoxy-d)oct-1-ene (4c)**	^{1}H NMR (400 MHz, $CDCl_3$) δ 5.64 (ddd, *J* = 16.9, 10.6, 7.8 Hz, 1H), 5.25 – 5.09 (m, 2H), 3.48 (m, 1H), 3.30 – 3.19 (m, 2H), 1.60 – 1.38 (m, 2H), 1.37 – 1.19 (m, 6H), 0.88 (t, J = 6.8 Hz, 3H).<br>93% D-inc. calculated from ^{1}H NMR.<br>^{13}C NMR (100 MHz, $CDCl_3$) δ 138.97, 117.04, 83.13, 56.14-55.64 (-O$\underline{C}H_2$–D), 53.46 (DCM), 35.32, 31.84, 29.72, 24.99, 22.63, 14.08.<br>HRMS (EI-TOF) m/z: [M]+ Calcd for $C_9H_{17}DO$ 143.1420; Found 143.1425.
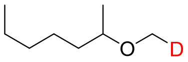<br>**2-(Methoxy-d)heptane (4d)**	^{1}H NMR (400 MHz, $CDCl_3$) δ 3.37 – 3.20 (m, 3H), 1.59 – 1.36 (m, 2H), 1.29 (m, 6H), 1.12 (d, *J* = 6.1 Hz, 3H), 0.89 (t, *J* = 6.8 Hz, 3H).<br>99% D-inc. calculated from ^{1}H NMR.<br>^{13}C NMR (100 MHz, $CDCl_3$) δ 76.87, 55.86-55.43 (-O$\underline{C}H_2$–D), 36.31, 32.01, 25.15, 22.67, 19.04, 14.08.<br>HRMS (EI-TOF) m/z: [M]+ Calcd for $C_8H_{17}DO$ 131.1420; Found 131.1420.
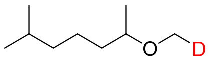<br>**2-(Methoxy-d)-6-methylheptane (4e)**	^{1}H NMR (400 MHz, $CDCl_3$) δ 3.32– 3.29 (m, 3H), 1.57 – 1.48 (m, 2H), 1.26 (m, 5H), 1.12 (d, *J* = 6.1 Hz, 3H), 0.90 – 0.86 (m, 6H).<br>97% D-inc. calculated from ^{1}H NMR.<br>^{13}C NMR (100 MHz, $CDCl_3$) δ 60.45, 55.89-55.45 (-O$\underline{C}H_2$–D), 53.47 (DCM), 39.11, 36.57, 30.99, 27.99, 23.24, 22.62, 19.04.<br>HRMS (EI-TOF) m/z: [M]+ Calcd for $C_9H_{19}DO$ 145.1577; Found 145.1580.
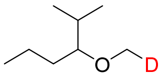<br>**3-(Methoxy-d)-2-methylhexane (4f)**	^{1}H NMR (400 MHz, $CDCl_3$) δ 3.37 – 3.28 (m, 2H), 2.93 – 2.83 (m, 1H), 1.91 – 1.76 (m, 1H), 1.48 – 1.29 (m, 4H), 0.90 (m, 9H).<br>96% D-inc. calculated from ^{1}H NMR.<br>^{13}C NMR (100 MHz, $CDCl_3$) δ 86.10, 57.67-57.15 (-O$\underline{C}H_2$–D), 53.44 (DCM), 40.95(DMSO), 32.47, 30.31, 19.02, 18.17, 17.94, 14.36.<br>HRMS (EI-TOF) m/z: [M]+ Calcd for $C_8H_{17}DO$ 131.1420; Found 131.1429.
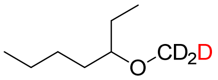<br>**3-(Methoxy-d3)heptane (4g)**	^{1}H NMR (400 MHz, $CDCl_3$) δ 3.67 (m, 0.27H), 3.01 (m, 1H), 1.46 – 1.36 (m, 4H), 1.28 – 1.15 (m, 4H), 0.87 – 0.69 (m, 6H).<br>98% D-inc. calculated from ^{1}H NMR.<br>^{13}C NMR (100 MHz, $CDCl_3$) δ 80.97, 54.68-54.26 (-O$\underline{C}D_3$), 31.70, 26.53, 24.81, 21.93, 13.09, 8.34.<br>HRMS (EI-TOF) m/z: [M]+ Calcd for $C_8H_{15}D_3O$ 133.1546; Found 133.1544.
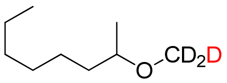<br>**2-(Methoxy-d3)octane (4h)**	^{1}H NMR (400 MHz, $CDCl_3$) δ 3.28 (m, 1H), 1.59 – 1.36 (m, 2H), 1.28 (m, 8H), 1.12 (d, *J* = 6.1 Hz, 3H), 0.94 – 0.84 (m, 3H).<br>94% D-inc. calculated from ^{1}H NMR.<br>^{13}C NMR (100 MHz, $CDCl_3$) δ 76.77, 55.24-54.81 (-O$\underline{C}D_3$), 36.35, 31.87, 29.45, 25.43, 22.63, 19.03, 14.09.<br>HRMS (EI-TOF) m/z: [M]+ Calcd for $C_9H_{17}D_3O$ 147.1702; Found 147.1703.
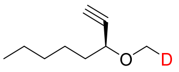<br>**3-(Methoxy-d)oct-1-yne (4i)**	HRMS (EI-TOF) m/z: [M]+ Calcd for $C_9H_{15}DO$ 141.1264; Found 141.1252.<br>97% D-inc. calculated from HRMS.
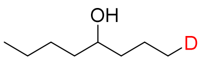<br>**Octan-8-d-4-ol (4j)**	^{1}H NMR (400 MHz, $CDCl_3$) δ 3.59 – 3.48 (m, 1H), 1.41 – 1.19 (m, 10H), 0.90 – 0.80 (m, 5H).<br>92% D-inc. calculated from ^{1}H NMR.<br>^{13}C NMR (100 MHz, $CDCl_3$) δ 71.75, 39.66, 37.21, 27.85, 22.78, 18.75, 14.14-13.66

	(-CH$_2$–D, -CH$_3$).<br>HRMS (EI-TOF) m/z: [M]+ Calcd for $C_8H_{17}DO$ 131.1420; Found 131.1424.
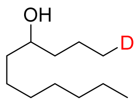<br>**Undecan-1-d-4-ol (4k)**	^{1}H NMR (400 MHz, $CDCl_3$) δ 3.60 (m, 1H), 1.43 (m, 6H), 1.29 (m, 10H), 0.88 (m, 5H).<br>83% D-inc. calculated from ^{1}H NMR.<br>^{13}C NMR (100 MHz, $CDCl_3$) δ 71.78, 39.65, 37.52, 31.85, 29.69, 29.32, 25.67, 22.67, 18.75, 14.11-13.65 (-CH$_2$–D, -CH$_3$).<br>HRMS (EI-TOF) m/z: [M]+ Calcd for $C_{11}H_{23}DO$ 173.1890; Found 173.1899.
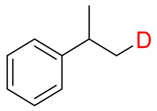<br>**(Propan-2-yl-1-d)benzene (5a)**	^{1}H NMR (400 MHz, $CDCl_3$) δ 7.26 – 7.07 (m, 5H), 2.83 (m, 1H), 1.18 (m, 5H).<br>^{13}C NMR (100 MHz, $CDCl_3$) δ 148.89, 128.32, 126.44, 125.77, 34.05, 24.00-23.53 (-CH$_2$–D, -CH$_3$).<br>HRMS (EI-TOF) m/z: [M]+ Calcd for $C_9H_{11}D$ 121.1002; Found 121.1002.<br>93% D-inc. calculated from HRMS.
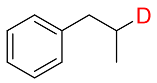<br>**(Propyl-2-d)benzene (5b)**	^{1}H NMR (400 MHz, $CDCl_3$) δ 7.29-7.24 (m, 2H), 7.21-7.14 (m, 3H), 2.62 – 2.54 (d, 2H), 1.69 – 1.55 (m, 1H), 0.98 – 0.83 (d, 3H).<br>94% D-inc. calculated from ^{1}H NMR.<br>^{13}C NMR (100 MHz, $CDCl_3$) δ 142.75, 128.51, 128.25, 125.63, 38.03, 30.36, 24.64-24.06 (-CHD-), 13.79.<br>HRMS (EI-TOF) m/z: [M]+ Calcd for $C_9H_{11}D$ 121.1002; Found 121.0998.
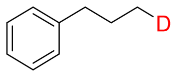<br>**(Propyl-3-d)benzene (5c)**	^{1}H NMR (400 MHz, $CDCl_3$) δ 7.31 – 7.23 (m, 2H), 7.22 – 7.12 (m, 3H), 2.62 – 2.54 (t, 2H), 1.71 – 1.57 (m, 2H), 0.92 (m, 2H).<br>^{13}C NMR (100 MHz, $CDCl_3$) δ 140.87, 126.63, 126.37, 123.76, 36.22, 22.68, 12.01-11.52 (-CH$_2$–D).<br>HRMS (EI-TOF) m/z: [M]+ Calcd for $C_9H_{11}D$ 121.1002; Found 121.0999.<br>96% D-inc. calculated from HRMS.
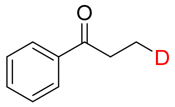<br>**1-Phenyl-1-propanone-3-d (5d)**	^{1}H NMR (400 MHz, $CDCl_3$) δ 7.95 – 7.79 (m, 2H), 7.51 – 7.41 (m, 1H), 7.37 (m, 2H), 3.08 – 2.71 (m, 2H), 1.17 – 1.08 (m, 2H).<br>86% D-inc. calculated from ^{1}H NMR.<br>^{13}C NMR (100 MHz, $CDCl_3$) δ 199.90, 135.88, 131.87, 127.53, 126.96, 30.75, 7.21-6.76 (-CH$_2$–D).<br>HRMS (EI-TOF) m/z: [M]+ Calcd for C_9H_9DO 135.0794; Found 135.0801.
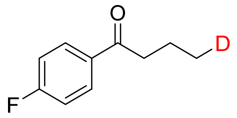<br>**1-(4-Fluorophenyl)butan-1-one-4-d (5e)**	^{1}H NMR (400 MHz, $CDCl_3$) δ 7.99 (dd, J = 8.7, 5.6 Hz, 2H), 7.12 (t, J = 8.6 Hz, 2H), 2.92 (t, J = 7.3 Hz, 2H), 1.84 – 1.69 (m, 2H), 1.09 – 0.93 (m, 2H).<br>87% D-inc. calculated from ^{1}H NMR.<br>^{13}C NMR (100 MHz, $CDCl_3$) δ 198.82, 166.91, 164.38, 133.55, 133.52, 130.70, 130.61, 128.84, 128.76, 115.73, 115.51, 40.41, 17.66, 13.86-13.37 (-CH$_2$–D).<br>HRMS (EI-TOF) m/z: [M]+ Calcd for $C_{10}H_{10}DOF$ 167.0857; Found 167.0863.
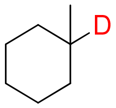<br>**Methylcyclohexane-1-d (5f)**	HRMS (EI-TOF) m/z: [M]+ Calcd for $C_7H_{13}D$ 99.1158; Found 99.1188.<br>97% D-inc. calculated from HRMS.
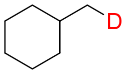<br>**(Methyl-d)cyclohexane (5g)**	HRMS (EI-TOF) m/z: [M]+ Calcd for $C_7H_{13}D$ 99.1158; Found 99.1161.<br>90% D-inc. calculated from HRMS.

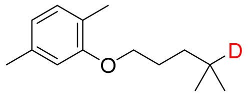<br>**1,4-dimethyl-2-((4-methylpentyl-4-d)oxy)benzene (5h), from Gemfibrozil.**	^{1}H NMR (400 MHz, $CDCl_3$) δ 7.00 (d, J = 7.4 Hz, 1H), 6.69 – 6.60 (m, 2H), 3.92 (t, J = 6.5 Hz, 2H), 2.31 (s, 3H), 2.18 (s, 3H), 1.85 – 1.73 (m, 2H), 1.43 – 1.29 (m, 2H), 0.92 (s, 6H).<br>91% D-inc. calculated from ^{1}H NMR.<br>^{13}C NMR (100 MHz, $CDCl_3$) δ 157.15, 136.46, 130.27, 123.65, 120.55, 112.01, 68.18, 53.46, 35.20, 27.80-27.11 (-C-D, -C-H), 22.62, 22.49, 21.44, 15.82.<br>HRMS (EI-TOF) m/z: [M]+ Calcd for $C_{14}H_{21}DO$ 207.1733; Found 207.1736.
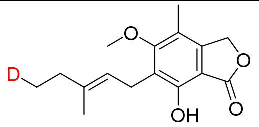<br>**(E)-7-hydroxy-5-methoxy-4-methyl-6-(3-methylpent-2-en-1-yl-5-d)isobenzofuran-1(3H)-one (5i), from Mycophenolic acid.**	^{1}H NMR (400 MHz, $CDCl_3$) δ 7.67 (s, 1H), 5.20 (s, 2H), 5.21 – 5.14 (m, 1H), 3.77 (s, 3H), 3.40 (d, J = 6.9 Hz, 2H), 2.15 (s, 3H), 1.98 (t, J = 7.4 Hz, 2H), 1.79 (s, 3H), 1.01 – 0.90 (m, 2H).<br>91% D-inc. calculated from ^{1}H NMR.<br>^{13}C NMR (100 MHz, $CDCl_3$) δ 173.02, 163.73, 153.72, 143.89, 137.78, 122.66, 120.42, 116.72, 106.38, 70.07, 61.03, 32.28, 22.59, 16.15, 12.50-12.15 (-CH_2-D, -CH_3), 11.59.<br>HRMS (EI-TOF) m/z: [M]+ Calcd for $C_{16}H_{19}DO_4$ 277.1424; Found 277.1423.
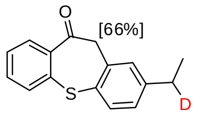<br>**2-(ethyl-1-d)dibenzo[b,f]thiepin-10(11H)-one (5j), from Zaltoprofen.**	^{1}H NMR (400 MHz, $CDCl_3$) δ 8.19 (dd, J = 7.9, 1.7 Hz, 1H), 7.59 (dd, J = 7.9, 1.3 Hz, 1H), 7.54 (d, J = 7.9 Hz, 1H), 7.41 (td, J = 7.6, 1.7 Hz, 1H), 7.35 – 7.24 (m, 2H), 7.03 (dd, J = 7.9, 2.0 Hz, 1H), 4.34 (s, 0.67 H), 2.69 – 2.56 (m, 1H), 1.32 – 1.17 (d, 3H).<br>93% D-inc. calculated from ^{1}H NMR.<br>^{13}C NMR (100 MHz, $CDCl_3$) δ 191.78, 146.66, 140.71, 137.51, 136.18, 132.43, 131.51, 131.32, 131.19, 130.78, 128.94, 126.79, 126.69, 51.06-50.52 (-C(O)-C-D_2, -C(O)-CH-D, -C(O)-CH_2), 28.54 - 28.01 (-CH-D, -CH_2-), 15.42, 15.34.<br>HRMS (EI-TOF) m/z: [M]+ Calcd for $C_{16}H_{11}D_3OS$ 257.0964; Found 257.0916.
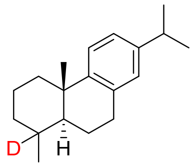<br>**(4aS, 10aS)-7-isopropyl-1,4a-dimethyl-1,2,3,4,4a,9,10,10a-octahydrophenanthrene-1-d (5k), from Dehydroabietic acid.**	^{1}H NMR (400 MHz, $CDCl_3$) δ 7.16 (d, J = 8.1 Hz, 1H), 6.99 (dd, J = 8.2, 2.1 Hz, 1H), 6.89 (s, 1H), 2.88 (td, J = 7.0, 2.1 Hz, 2H), 2.84 – 2.76 (m, 1H), 2.28 – 2.15 (m, 1H), 2.02 – 1.88 (m, 1H), 1.85 – 1.69 (m, 2H), 1.68 (dd, J = 12.8, 2.3 Hz, 1H), 1.56 (tt, J = 10.0, 2.4 Hz, 3H), 1.48 (dtd, J = 15.3, 5.6, 4.7, 2.5 Hz, 1H), 1.38 (td, J = 13.2, 3.6 Hz, 1H), 1.23 (d, J = 7.0, 1.8 Hz, 6H), 1.17 (s, J = 0.8 Hz, 3H), 0.99 (s, 3H).<br>90% D-inc. calculated from ^{1}H NMR.<br>^{13}C NMR (100 MHz, $CDCl_3$) δ 147.19, 145.45, 135.11, 127.00, 124.33, 123.83, 44.35, 38.62, 37.26, 33.48, 32.97, 30.38, 25.59, 24.55, 24.04, 24.02, 22.81-20.41 (-C−D, -C-H), 18.07, 15.05.<br>HRMS (EI-TOF) m/z: [M]+ Calcd for $C_{19}H_{27}D$ 257.2254; Found 257.2258.

Supplementary References

1. Xu, J., Arkin, M., Peng, Y. Z., Xu, W. H., Yu, H. L., Lin, X. F., Wu, Q. Enantiocomplementary decarboxylative hydroxylation combining photocatalysis and whole-cell biocatalysis in a one-pot cascade process. *Green Chem* **21**, 1907-1911 (2019).
2. Bagutski, V., Elford, T.G., Aggarwal, V.K. Synthesis of highly enantioenriched C-tertiary amines from boronic esters: application to the synthesis of Igmesine. *Angew. Chem. Int. Edit.* **50**, 1080-1083 (2011).
