# Supplementary material for: Light-driven decarboxylative deuteration enabled by a divergently engineered photodecarboxylase
Source: Nat Commun. 2021 Jun 25;12:3983. doi: 10.1038/s41467-021-24259-6 (PMC8233396; doi:10.1038/s41467-021-24259-6)
Supplement: Supplementary file 3 — Reporting Summary [file 41467_2021_24259_MOESM3_ESM.pdf]

nature research

Corresponding author(s): J. Xu & Q. Wu

Last updated by author(s): Jun 3, 2021

Reporting Summary

Nature Research wishes to improve the reproducibility of the work that we publish. This form provides structure for consistency and transparency in reporting. For further information on Nature Research policies, see our Editorial Policies and the Editorial Policy Checklist.

Statistics

For all statistical analyses, confirm that the following items are present in the figure legend, table legend, main text, or Methods section.

n/a	Confirmed	
☐	☒	The exact sample size (n) for each experimental group/condition, given as a discrete number and unit of measurement
☐	☒	A statement on whether measurements were taken from distinct samples or whether the same sample was measured repeatedly
☒	☐	The statistical test(s) used AND whether they are one- or two-sided<br>*Only common tests should be described solely by name; describe more complex techniques in the Methods section.*
☒	☐	A description of all covariates tested
☒	☐	A description of any assumptions or corrections, such as tests of normality and adjustment for multiple comparisons
☐	☒	A full description of the statistical parameters including central tendency (e.g. means) or other basic estimates (e.g. regression coefficient) AND variation (e.g. standard deviation) or associated estimates of uncertainty (e.g. confidence intervals)
☒	☐	For null hypothesis testing, the test statistic (e.g. F, t, r) with confidence intervals, effect sizes, degrees of freedom and P value noted<br>*Give P values as exact values whenever suitable.*
☒	☐	For Bayesian analysis, information on the choice of priors and Markov chain Monte Carlo settings
☒	☐	For hierarchical and complex designs, identification of the appropriate level for tests and full reporting of outcomes
☒	☐	Estimates of effect sizes (e.g. Cohen's d, Pearson's r), indicating how they were calculated

Our web collection on statistics for biologists contains articles on many of the points above.

Software and code

Policy information about availability of computer code

Data collection	GC data acquisition was collected by SHIMADZU™ GCsolution software version 3 in a GC-2014 gas chromatography system. Autodock 4.2.6 was used to collect all data of docking experiment. Amber 12 and the embedded algorithms such as SHAKE algorithm, AMBER TLEAP module, NVT ensemble, Langevin thermostat, NPT ensemble, Particle Mesh Ewald (PME) method, Berendsen pressure coupling, were used to collect all data of MD simulations.
Data analysis	GC data was analyzed with SHIMADZU™ GCsolution software version 3 in a GC-2014 gas chromatography system. PyMOL.99rc6 was used to prepare all structural figures of proteins. Autodock 4.2.6 was used to analyze all data of docking experiment. Amber 12 and the embedded SHAKE algorithm, AMBER TLEAP module, NVT ensemble, Langevin thermostat, NPT ensemble, Particle Mesh Ewald (PME) method, Berendsen pressure coupling, were used to analyze the data of MD simulations. OriginPro was used to prepare all supplementary figures for the comparison of the distance between the carboxyl of substrates and the N5 atom of FAD in variants by MD simulation.

For manuscripts utilizing custom algorithms or software that are central to the research but not yet described in published literature, software must be made available to editors and reviewers. We strongly encourage code deposition in a community repository (e.g. GitHub). See the Nature Research guidelines for submitting code & software for further information.

Data

Policy information about availability of data

All manuscripts must include a data availability statement. This statement should provide the following information, where applicable:
- Accession codes, unique identifiers, or web links for publicly available datasets
- A list of figures that have associated raw data
- A description of any restrictions on data availability

The data supporting the findings of this study are available within the paper and its supplementary information files. The source data underlying Fig. 1d-1f and 1h,

and Supplementary Figs. 1-7 and Supplementary Table 3 are provided as a source data file. PDB file used in this study is available in Protein Data Bank (PDB) (ID: 5NCC, https://www1.rcsb.org/structure/5NCC). Source data are provided with this paper.

Field-specific reporting

Please select the one below that is the best fit for your research. If you are not sure, read the appropriate sections before making your selection.

- [x] Life sciences
- [ ] Behavioural & social sciences
- [ ] Ecological, evolutionary & environmental sciences

For a reference copy of the document with all sections, see nature.com/documents/nr-reporting-summary-flat.pdf

Life sciences study design

All studies must disclose on these points even when the disclosure is negative.

Sample size	No statistical methods were used to predetermine sample size. Sample size for all experiments in this study was determined to be adequate based on the magnitude and consistency of measurable differences between groups.
Data exclusions	No data were excluded from the analyses.
Replication	All attempts at replication were successful. We generally performed three independent experiments.
Randomization	Single clones from the LB plates spread with the transformation mixture were randomly picked when necessary. Randomization was not applicable to other experiments done since no statistical comparisons were made.
Blinding	Blinding was not relevant to the study since no statistical comparisons were made.

Reporting for specific materials, systems and methods

We require information from authors about some types of materials, experimental systems and methods used in many studies. Here, indicate whether each material, system or method listed is relevant to your study. If you are not sure if a list item applies to your research, read the appropriate section before selecting a response.

Materials & experimental systems

n/a	Involved in the study	
x		Antibodies
x		Eukaryotic cell lines
x		Palaeontology and archaeology
x		Animals and other organisms
x		Human research participants
x		Clinical data
x		Dual use research of concern

Methods

n/a	Involved in the study	
x		ChIP-seq
x		Flow cytometry
x		MRI-based neuroimaging
